# TARBP2 facilitates renal cell carcinoma immune escape by suppressing 53BP1 phase separation to upregulate B7-H3

**DOI:** 10.1172/JCI199525

**Published:** 2026-11-02

**Authors:** Minyu Chen, Mingjie Lin, Jiangquan Zhu, Kezhi Liu, Guannan Shu, Zhuting Tong, Mi Zhou, Liangmin Fu, Taowei Yang, Yihui Pan, Xinwei Zhou, Jinchen Luo, Wuyuan Liao, Qihao Li, Lican Liao, Hansen Lin, Kangbo Huang, Lizhen Zhang, Wei Chen, Junhang Luo, Jiaxing Zhang

**Affiliations:** 1 Department of Urology, The First Affiliated Hospital of Sun Yat-sen University, Guangzhou, China.; 2 Department of Urology, Sichuan Provincial People’s Hospital, University of Electronic Science and Technology of China, Chengdu, China.; 3 Department of Urology, Guangzhou Women and Children’s Medical Center, Guangzhou Medical University, Guangdong Provincial Clinical Research Center for Child Health, Guangzhou, China.; 4 Department of Radiation Oncology, The First Affiliated Hospital of Anhui Medical University, Hefei, China.; 5 Department of Oncology and Southwest Cancer Center, Southwest Hospital of The Third Military Medical University (Army Medical University), Chongqing, China.; 6 Department of Urology, The Second Xiangya Hospital of Central South University, Changsha, China.; 7 Key Laboratory of Diabetes Immunology (Central South University), Ministry of Education, National Clinical Research Center for Metabolic Disease, Changsha, China.; 8 Uro-Oncology Institute of Central South University, Changsha, China.; 9 Department of Urology, The Third Affiliated Hospital of Soochow University, Changzhou, China.; 10 Department of Urology, Cancer Hospital Chinese Academy of Medical Sciences, Shenzhen, China.; 11 Department of Oncology, The First Affiliated Hospital of Sun Yat-sen University, Guangzhou, China.; 12 Department of Urology and; 13 State Key Laboratory of Oncology in South China, Guangdong Provincial Clinical Research Center for Cancer, Sun Yat-sen University Cancer Center, Guangzhou, China.; 14 Institute of Precision Medicine, The First Affiliated Hospital of Sun Yat-sen University, Guangzhou, China.

**Keywords:** Immunology, Oncology, Cancer immunotherapy, NK cells, Oncogenes

## Abstract

Renal cell carcinoma (RCC) harbors an immunosuppressive tumor microenvironment that facilitates immune evasion and resistance to immune checkpoint blockade (ICB). However, the factors driving immune escape in RCC remain incompletely understood. In this study, transactivation response RNA-binding protein 2 (TARBP2) was identified as a critical regulator of the immune checkpoint molecule B7-H3. Elevated *TARBP2* expression was associated with poor prognosis and ICB resistance in patients with RCC. Functionally, TARBP2 in RCC cells suppressed the intratumoral accumulation and cytotoxicity of T cells and particularly NK cells, thereby promoting immune escape. Mechanistically, nuclear TARBP2 interacted with p53-binding protein 1 (53BP1), inhibiting 53BP1 phase separation and subsequent p53 activation, thereby augmenting *B7-H3* transcription. Furthermore, TARBP2 inhibition significantly enhanced ICB efficacy when combined with adoptive NK cell therapy. Collectively, these findings establish TARBP2 as a key mediator of immune evasion in RCC and a promising therapeutic target for overcoming ICB resistance.

## Abstract 2

A protein called TARBP2 helps renal cell carcinoma (RCC) evade immune attack by increasing B7-H3, suggesting a way to improve immunotherapy.

## Introduction

Renal cell carcinoma (RCC) represents a substantial global health burden, with an estimated 80,980 newly diagnosed cases and 14,510 deaths projected in the United States in 2025 (1). Early-stage RCC has a favorable prognosis, with more than 94% 5-year cancer-specific survival after nephrectomy, yet advanced stages carry a high risk of recurrence and mortality, with 11% presenting at stage IV at diagnosis (2). Immune checkpoint blockade (ICB) immunotherapy has become a cornerstone treatment for advanced RCC, providing durable responses in a subset of patients (3–5). However, most patients do not achieve sustained benefit from ICB, and the mechanisms underlying intrinsic and acquired ICB resistance remain poorly understood (2, 6). Consequently, elucidating key molecular drivers of poor prognosis and ICB resistance in RCC is essential for improving personalized therapeutic strategies and patient outcomes.

Transactivation response RNA-binding protein 2 (TARBP2) is a multifunctional protein that interacts with miRNAs, siRNAs, mRNAs, and proteins to regulate their functions. Its canonical function involves miRNA biogenesis, whereby TARBP2 recruits precursor miRNAs to Dicer for cleavage to generate mature miRNAs and facilitates loading of mature miRNAs onto Argonaute 2 to form the RNA-induced silencing complex (RISC) (7). TARBP2 also associates with siRNAs and assists in their assembly into RISC (7, 8). In addition, TARBP2 directly binds target mRNAs to modulate their stability and degradation, a mechanism critically implicated in cancer pathogenesis (9, 10). Intriguingly, TARBP2 also engages in protein–protein interactions that modulate the activity and stability of interacting proteins independently of RNA binding (11, 12). Despite these established functions, the molecular mechanisms through which TARBP2 drives RCC progression and shapes the tumor immune microenvironment remain poorly understood.

B7-H3 (CD276) is a transmembrane immune checkpoint protein belonging to the B7 family, which also includes B7-1 (CD80), B7-2 (CD86), B7-H1 (PD-L1), B7-DC (PD-L2), B7-H2 (ICOS-L), and B7-H5 (VISTA) (13). B7-H3 shares 20%–27% amino acid identity with other members of the B7 family and was initially identified as a costimulatory molecule (14). B7-H3 is now recognized as an important suppressor of antitumor immunity mediated by T cells and NK cells, though its receptor has not been elucidated (15). In RCC, B7-H3 expression is elevated in clear-cell RCC tissues relative to normal kidney tissues and correlates with poor prognosis (16). Additionally, high B7-H3 expression is associated with inferior survival outcomes in patients with metastatic RCC receiving ICB therapy (17). Although B7-H3 is increasingly recognized as a clinically relevant oncogenic and immunoregulatory factor in RCC, the molecular mechanisms responsible for its upregulation and its precise immunomodulatory functions in RCC remain insufficiently characterized.

In this study, elevated *TARBP2* expression was identified as a predictor of poor prognosis and resistance to ICB therapy in RCC cohorts, with *TARBP2* showing preferential expression in malignant RCC cells. Functionally, TARBP2 in RCC cells promoted tumor immune escape by suppressing the intratumoral accumulation and cytotoxicity of T cells and particularly NK cells. Combined TARBP2 inhibition, adoptive NK cell therapy, and ICB treatment produced the strongest antitumor effects. Mechanistically, nuclear TARBP2 interacted with p53-binding protein 1 (53BP1) and suppressed 53BP1 phase separation, thereby impairing 53BP1-dependent p53 activation and promoting *B7-H3* transcription. These results identify the TARBP2/53BP1/p53/B7-H3 axis as a key regulator of the immunosuppressive tumor microenvironment in RCC and support TARBP2 as a potential therapeutic target for improving the efficacy of ICB-based immunotherapy.

## Results

### Identification of TARBP2 as a biomarker of poor prognosis and ICB resistance in RCC.

Integrated analyses of RNA-seq data from The Cancer Genome Atlas–Kidney Renal Clear Cell Carcinoma (TCGA-KIRC) cohort, the CheckMate ICB treatment cohort, and pretreatment FFPE samples from patients with RCC receiving ICB therapy were conducted to identify genes associated with poor prognosis and ICB resistance. *TARBP2* and *GINS3* emerged as candidate genes exhibiting 3 consistent unfavorable characteristics, including association with poor prognosis in both TCGA-KIRC and CheckMate cohorts based on a Cox proportional hazards model, elevated expression in patients with progressive disease following ICB therapy, and increased expression in pretreatment FFPE samples from ICB nonresponders (Figure 1, A and B, and Supplemental Table 1; supplemental material available online with this article; https://doi.org/10.1172/JCI199525DS1). Among these, *TARBP2* expression was associated with stage III–IV disease in the TCGA-KIRC cohort, whereas *GINS3* did not exhibit a similar association (Figure 1C). In the CheckMate cohort, both genes showed a trend toward higher expression in cases of progressive disease (Figure 1D and Supplemental Figure 1, A–C). However, Kaplan-Meier survival analyses revealed that elevated expression of *TARBP2* was associated with reduced overall survival (OS) in the TCGA-KIRC cohort and reduced OS and progression-free survival in the CheckMate cohort. In contrast, *GINS3* showed a weaker association restricted mainly to progression-free survival in the CheckMate cohort (Supplemental Figure 1, D–I). Furthermore, *TARBP2* but not *GINS3* retained prognostic value across independent RCC (E-MTAB-1980) and melanoma anti–PD-1 cohorts (Figure 1, E and F, and Supplemental Figure 1, J and K) (18). Therefore, *TARBP2* was selected for subsequent analyses, and its prognostic relevance was validated in an independent RCC cohort from Sun Yat-sen University (SYSU) using IHC staining (Figure 1G and Supplemental Table 2).

To further characterize *TARBP2* at single-cell resolution, integrated scRNA-seq datasets (19, 20) comprising 36 tissue samples from 13 patients with advanced RCC were analyzed (Supplemental Figure 1L). Following quality control and data integration, 192,626 high-quality cells were classified into 12 major cell populations based on canonical marker gene expression profiles (Figure 1H and Supplemental Figure 1M). To resolve epithelial and malignant compartments, epithelial and RCC cells were further subclustered into 15 transcriptionally distinct subpopulations (Supplemental Figure 2A). Multimodal analyses incorporating tissue origin, RCC marker expression, copy number variation (CNV) profiles, and pseudotime trajectory inference were used to define cellular states. Clusters 15, 14, 13, and 10 were identified as epithelial cells based on enrichment in adjacent normal tissues, low expression of *CA9* and *NDUFA4L2*, absence of CNV amplification, and early pseudotime localization. Clusters 4, 3, and 1 exhibited elevated CNV burden and late pseudotime positioning, consistent with malignant RCC identity. Other clusters retained epithelial transcriptional features and were therefore designated as epithelial-RCC (E-RCC) cells, consistent with the preservation of epithelial traits during early tumor development (Figure 1I and Supplemental Figure 2, B–I) (21). Trajectory reconstruction demonstrated a continuous transition from epithelial cells to E-RCC cells and subsequently to malignant RCC cells (Figure 1J), accompanied by a progressive increase in *TARBP2* expression (Figure 1K). Specifically, *TARBP2* expression was highest in malignant RCC cells irrespective of ICB treatment status (Figure 1L and Supplemental Figure 2, J and K). Furthermore, RCC cells with elevated *TARBP2* expression displayed reduced immune response activation scores compared with E-RCC cells (Figure 1M), suggesting an association between TARBP2 expression, tumor progression, and the establishment of an immunosuppressive tumor microenvironment.

### TARBP2 inhibits antitumor immunity in RCC.

To determine whether TARBP2 regulates antitumor immune responses in RCC, *Tarbp2* was silenced in the murine RCC cell line Renca using lentiviral shRNA constructs (Supplemental Figure 3A). Results from cell counting kit-8 (CCK-8) and colony formation assays demonstrated that *Tarbp2* knockdown did not affect the in vitro proliferation capacity of Renca cells (Supplemental Figure 3, B and C). In vivo experiments demonstrated that *Tarbp2* knockdown did not affect the growth of Renca-derived subcutaneous tumors in immunodeficient NCG mice (Figure 2, A–D) but significantly suppressed tumor growth in immunocompetent BALB/c mice (Figure 2, E–H). These findings indicate that the tumor-promoting effects of TARBP2 are dependent on interactions with the host immune system rather than the intrinsic proliferative capacity of RCC cells.

To further characterize the immunological alterations associated with *Tarbp2* knockdown, immune cell composition in Renca-derived tumors from BALB/c mice was analyzed by flow cytometry (Supplemental Figure 4A). *Tarbp2* knockdown significantly increased the intratumoral accumulation of CD8^+^ T cells, CD4^+^ T cells, and NK cells (Figure 2I), whereas the abundance of B cells and tumor-associated macrophages remained largely unchanged (Supplemental Figure 4B). Notably, although the proportion of CD4^+^FOXP3^+^ Tregs among CD4^+^ T cells was decreased, no significant alteration was observed in the percentage of Tregs within CD45^+^ cells (Supplemental Figure 4C). Moreover, the effector function of CD8^+^ T cells, CD4^+^ T cells, and NK cells, as indicated by granzyme B and IFN-γ, was enhanced in the *Tarbp2*-knockdown group (Figure 2J and Supplemental Figure 4D). Ki67 and TUNEL staining revealed that *Tarbp2* knockdown exhibited no significant effect on proliferation but markedly increased apoptosis rates in tumor sections from BALB/c mice (Figure 2, K and L). Given that both independent shRNAs targeting *Tarbp2* produced consistent phenotypic effects, sh*Tarbp2*#3, which showed the highest silencing efficiency (Supplemental Figure 3A), was used for subsequent experiments and is hereafter designated as sh*Tarbp2*.

To investigate tumor-immune interactions, we performed cell–cell communication analysis using the integrated scRNA-seq data. The results illustrated that RCC cells with elevated *TARBP2* expression exhibited enhanced communication with T and NK cells compared with E-RCC cells, regardless of ICB treatment status (Figure 2, M and N). Further ligand-receptor analysis revealed enhanced coinhibitory signaling mediated by immune checkpoint ligand-receptor pairs in RCC-T/NK cell interactions relative to E-RCC-T/NK cell interactions. Representative examples included the LGALS9-HAVCR2 pair in RCC-T cell communication and the HLA-E-CD94:NKG2A pair in RCC-NK cell crosstalk (Figure 2O).

Collectively, these results indicate that elevated TARBP2 in RCC cells inhibits antitumor immunity.

### TARBP2 attenuates NK cell cytotoxic activity in RCC.

Having discovered that TARBP2 in RCC cells suppresses T and NK cell function within the tumor microenvironment, we next investigated which immune cell populations are primarily targeted by TARBP2. CD8^+^ T, CD4^+^ T, or NK cells were selectively depleted using specific antibodies in BALB/c mice bearing subcutaneous tumors (Figure 3A). The tumor growth suppression observed in mice implanted with sh*Tarbp2* Renca cells was most strongly reversed by NK cell depletion, followed by partial reversal upon CD8^+^ T cell depletion, whereas CD4^+^ T cell depletion did not significantly affect tumor control in this model (Figure 3, B–D). These results demonstrate that the enhanced antitumor immunity induced by *Tarbp2* knockdown is dependent on CD8^+^ T cells and particularly NK cells, leading us to focus on NK cells.

To characterize NK cell alterations associated with *Tarbp2* knockdown, we employed scRNA-seq on CD3^–^NKp46^+^ cells isolated by FACS from control and sh*Tarbp2* Renca-derived tumors in BALB/c mice. After quality control, 37,915 high-quality cells were retained and classified into 8 clusters (Figure 3E). Cluster composition analysis revealed a reduction of cluster 2 and an increase of cluster 3 in the sh*Tarbp2* group (Figure 3F). We further conducted a systematic examination of specific markers to better depict the transcriptomic profiles in each cell cluster (Figure 3, G and H, and Supplemental Figure 5, A–H). Clusters 1–6 were defined as bona fide NK cells based on expression of *Nkg7*, *Ncr1*, and *Eomes* and negligible expression of *Cd3e*, *Cd8a*, and *Il7r*. Cluster 8 was annotated as an innate lymphoid cell (ILC) cluster due to expression of *Ncr1*, *Il7r*, and *Rora* and negligible expression of *Cd3e*, *Cd8a*, and *Eomes* (22, 23). Expression of *Tbx21* but not *Eomes* in the ILC cluster indicated the predominance of ILC1s (22). ILC cluster identification was further confirmed by utilizing published NK and ILC1-specific signatures (Supplemental Figure 5I) (24, 25). Cluster 7 was identified as cycling cells based on *Mki67* and *Birc5* expression. Subsequent reintegration and label transfer analysis following regression of cell cycle genes revealed a ubiquitous distribution of cycling cells across all NK/ILC clusters, indicating that cycling cells do not represent a distinct phenotype (Supplemental Figure 5J). Of note, cycling cells exhibited a significantly higher proportion of NK cluster 5 and a markedly lower proportion of NK cluster 6 compared with noncycling cells (Supplemental Figure 5K), suggesting that NK cluster 5 possesses greater proliferation potential, whereas NK cluster 6 exhibits opposing functional characteristics. Within NK subsets, cluster 6 exhibited a dysfunctional tissue-resident phenotype characterized by upregulation of *Klrg1*, *Itga1*, and *Havcr2*, alongside downregulation of *Itga2* and *Itgam*. In contrast, clusters 2 and 3 displayed elevated expression of *Itga2* and *Itgam*, corresponding to conventional cytotoxic NK cells (Figure 3G) (26). We further compared the transcriptomic profiles of clusters 2 and 3. Cluster 3 exhibited upregulated expression of *Ifng* and the activation marker *Cd69* (27), along with enhanced *Ccl4* expression, which facilitates DC recruitment to augment antitumor immune responses (28). In addition, the immune checkpoint gene *Tigit* was downregulated in cluster 3 (Figure 3H). Gene Ontology and Kyoto Encyclopedia of Genes and Genomes (KEGG) analyses illustrated enrichment of pathways related to immune activation in cluster 3 (Figure 3I). Moreover, the enrichment scores of the NK activation signature previously reported (29) were higher in cluster 3 compared with cluster 2, while TGF-β signaling, the activation of which suppresses NK cell proliferation and cytotoxicity (30), showed an inverse trend (Figure 3, J and K). Trajectory analysis of NK clusters 1–6 revealed that clusters 2 and 3 had transition potential toward the dysfunctional state characterized by cluster 6 (Supplemental Figure 5L). Gene regulatory network analysis was applied to further investigate the potential drivers of this transition. Our analysis revealed concomitant downregulation of multiple transcription factor regulons associated with NK cell activation and proliferation over pseudotime, including AP-1 complex members (e.g., *Jun*, *Fos*) and their upstream regulator *Ikzf1* (31), along with NF-κB family members (e.g., *Rel*, *Nfkb1*) (29). The heatmap demonstrated that cluster 3, positioned at earlier stages of pseudotime relative to cluster 2, exhibited higher levels of these regulons (Figure 3L). Since *Tarbp2* knockdown in RCC cells promoted the accumulation of cluster 3, these results suggest that TARBP2 in RCC cells may direct NK cell differentiation toward a dysfunctional trajectory.

We next investigated whether TARBP2 in RCC cells drives NK cell dysfunction through secreted factors or direct cell–cell contact. To this end, we used 0.4 μm Transwell inserts, which permit the diffusion of soluble mediators but prevent cell migration, to coculture Renca cells with murine primary NK cells (Supplemental Figure 6A). Under indirect coculture conditions, in which Renca and NK cells were separated by the Transwell inserts, sh*Tarbp2* did not alter Renca cell apoptosis or NK cell activation (e.g., *Cd69*), inhibitory (e.g., *Tigit*), and maturation (e.g., *Itgam*) marker expression. In contrast, under direct coculture conditions allowing physical contact between Renca and NK cells, sh*Tarbp2* significantly increased Renca cell apoptosis, enhanced NK cell activation and maturation marker expression, and reduced inhibitory marker expression, consistent with the scRNA-seq findings (Supplemental Figure 6, B and C). These results suggest that TARBP2 in RCC cells may regulate NK cell function in a contact-dependent manner.

To investigate whether TARBP2 suppresses NK cell cytotoxicity in vitro, primary RCC cells derived from patients were cocultured with NK-92MI cells and FACS-sorted peripheral blood NK cells (Supplemental Figure 6A and Supplemental Figure 7A). *TARBP2* knockdown in primary RCC cells (targets [T]) enhanced the cytotoxicity of NK cells (effectors [E]) at different E/T ratios (Figure 3M). The proportions of CD107a^+^, TNF-α^+^, and IFN-γ^+^ NK cells were increased after coculture with *TARBP2*-knockdown RCC cells (Figure 3N and Supplemental Figure 7, B and C).

In summary, TARBP2 in RCC cells inhibits NK cell cytotoxicity and may exert this effect, at least in part, through direct cell–cell contact with NK cells.

### TARBP2 drives NK cell dysfunction in RCC through the p53/B7-H3 axis.

We next investigated the molecular mechanisms underlying TARBP2-mediated modulation of NK cell function. Tumor cells from sh*Tarbp2* and control Renca-derived tumors in BALB/c mice were isolated using a magnetic-activated cell sorting (MACS) tumor cell isolation kit, and the transcriptomic profiles of these cells were obtained by RNA-seq (Supplemental Figure 8A). Bioinformatics analysis demonstrated substantial transcriptional alterations following *Tarbp2* knockdown, with 877 upregulated and 961 downregulated genes meeting the thresholds of |log_2_(fold change)| > 1 and *P* < 0.05 (Supplemental Figure 8B). Given that TARBP2-mediated regulation of NK cell function is dependent on direct cell–cell contact and that contact-dependent regulation of NK cell function is predominantly mediated by membrane-bound ligand-receptor interactions (32), cell surface molecules were prioritized as candidate mediators of TARBP2-driven modulation of NK cell function. Notably, *B7h3*, an immune checkpoint previously reported to attenuate NK cell–mediated tumor control (15, 33, 34) and whose human homolog *B7-H3* was also highly expressed in pretreatment FFPE samples from ICB nonresponders, emerged as the most significantly downregulated surface molecule following *Tarbp2* knockdown (Figure 4, A and B, and Supplemental Figure 8, C and D). Flow cytometry and immunofluorescence analyses further confirmed decreased plasma membrane–localized B7-H3 levels in MACS-isolated tumor cells from sh*Tarbp2* tumors (Figure 4C and Supplemental Figure 8E). A positive correlation between *TARBP2* and *B7-H3* expression was observed across multiple cancer types in the TCGA database (Figure 4D). This TARBP2 and B7-H3 coexpression pattern was also identified in the CheckMate ICB treatment cohort, the Clinical Proteomic Tumor Analysis Consortium RCC dataset, and Cancer Cell Line Encyclopedia RCC cell dataset (Figure 4E). The correlation was further validated by IHC in RCC samples (Figure 4F). We next performed KEGG pathway enrichment analysis of differentially expressed genes between tumor cells isolated from sh*Tarbp2* and control Renca-derived tumors, and enrichment of the p53 signaling pathway was observed (Supplemental Figure 8F). GSEA further confirmed activation of p53 signaling following *Tarbp2* knockdown (Figure 4G). Given prior evidence that p53 negatively regulates B7-H3 expression (35), we hypothesized that TARBP2 drives B7-H3 expression by suppressing the p53 pathway.

To validate this hypothesis, we conducted in vitro experiments using RCC cell lines with WT *TP53* (769-P, ACHN, Caki-1). Based on endogenous TARBP2 levels (Supplemental Figure 8, G and H), we knocked down *TARBP2* in 769-P and ACHN cells and overexpressed *TARBP2* in Caki-1 cells. *TARBP2* knockdown reduced both total and cell surface B7-H3 protein levels, while increasing p53 and its downstream target p21 (Figure 4, H and I, and Supplemental Figure 8, I and J). In contrast, *TARBP2* overexpression produced the opposite effects (Supplemental Figure 8, K and L). For all subsequent rescue experiments, sh*TARBP2*#2 was used and is hereafter referred to as sh*TARBP2*. Rescue experiments showed that *TP53* knockdown reversed the reduction in B7-H3 protein levels induced by sh*TARBP2* (Figure 4, J and K, and Supplemental Figure 9, A and B). p53 is a transcription factor encoded by *TP53*, one of the most frequently mutated genes in human cancers. Most *TP53* mutations are missense mutations located within the DNA-binding domain and impair the DNA-binding activity of p53 (36). Based on this, WT *TP53* or 3 common DNA-binding–deficient mutants (R175H, R248W, and R273H) were overexpressed in *TARBP2*-overexpressing Caki-1 cells. Only WT *TP53* reversed TARBP2-induced upregulation of B7-H3, whereas mutant constructs failed to do so (Supplemental Figure 9, C and D). Dual-luciferase reporter assays assessing *B7-H3* promoter–driven transcriptional activity showed trends consistent with the changes in B7-H3 protein levels. *TP53* knockdown reversed the decrease in *B7-H3* promoter–driven transcriptional activity induced by sh*TARBP2*, whereas only WT *TP53* overexpression reversed the increase in transcriptional activity induced by *TARBP2* overexpression (Figure 4L and Supplemental Figure 9, E and F). These findings suggest that TARBP2 promotes B7-H3 expression by suppressing the transcriptional regulatory activity of p53.

To further determine whether p53 directly binds to the *B7-H3* promoter region, the human *B7-H3* promoter sequence was analyzed using the JASPAR database. Potential p53-binding sites were identified within the *B7-H3* promoter region (Supplemental Figure 9G). ChIP-qPCR demonstrated significant enrichment of p53 at the *B7-H3* promoter. *TP53* knockdown abolished the increase in p53 enrichment induced by sh*TARBP2*, whereas only WT *TP53* overexpression rescued the decrease caused by *TARBP2* overexpression (Supplemental Figure 9, H–J). These findings indicate that TARBP2 promotes B7-H3 expression by inhibiting p53 binding to the *B7-H3* promoter region, thereby attenuating the transcriptional repression of *B7-H3* by p53.

To investigate the impact of the TARBP2/p53/B7-H3 axis on tumor growth and NK cell function in vivo, we performed genetic interventions targeting *Tarbp2*, *Trp53*, and *B7h3* in Renca cells, followed by subcutaneous implantation in BALB/c mice. Combined knockdown of *Tarbp2* and *Trp53* abrogated *Tarbp2* knockdown–induced tumor growth suppression, NK cell accumulation, and increases in CD107a^+^, TNF-α^+^, and IFN-γ^+^ NK cell proportions. Combined knockdown of *Tarbp2*, *Trp53*, and *B7h3* further reversed these effects observed in the double knockdown setting (Supplemental Figure 10, A–D). To determine the dependency of B7-H3, *B7h3*-knockout Renca cells were generated via CRISPR/Cas9 technology. These genetically modified cells subsequently underwent *Tarbp2* knockdown or overexpression for assessment of phenotypic alterations in BALB/c mice. No significant differences in tumor growth, NK cell accumulation, CD107a degranulation, or cytokine production were observed among *B7h3*-knockout groups (Figure 4, M–O, and Supplemental Figure 10, E and F).

In summary, TARBP2 in RCC cells impairs the antitumor potential of NK cells by relieving p53-mediated transcriptional repression of *B7-H3*.

### TARBP2 impairs 53BP1-p53 complex formation and chromatin accumulation.

Previous studies demonstrated that TARBP2 forms a complex with Dicer to participate in miRNA processing and RISC assembly, thereby facilitating posttranscriptional gene silencing (7, 37). TARBP2 has also been reported to regulate mRNA stability (9, 10). However, *TARBP2* knockdown or overexpression did not alter *TP53* mRNA levels (Supplemental Figure 11, A–C). Moreover, Dicer silencing in *TARBP2*-overexpressing Caki-1 cells did not reverse TARBP2-induced upregulation of B7-H3 or the downregulation of p53 and its downstream target p21 (Supplemental Figure 11, D and E). Notably, RCC samples from the SYSU cohort showed higher overall TARBP2 expression in high-grade (G3-4) tumors than in low-grade (G1-2) tumors, particularly in the nucleus (Figure 5A and Supplemental Figure 12A). Patients with higher nuclear TARBP2 expression exhibited poorer OS (Supplemental Figure 12B). Given that TARBP2 functions as a multifunctional protein capable of interacting with diverse partner proteins and modulating their functional activities (11, 12), nuclear and cytoplasmic fractions of 769-P cells were isolated and subjected to co-IP coupled with mass spectrometry analysis to characterize the TARBP2 protein interactome. Following subtraction of control IgG-bound proteins, nuclear-specific interactors were ranked by descending confidence score. Among the top candidates, 53BP1, a p53-binding regulator of p53 signaling (38), was identified within the top 5 hits (Figure 5B and Supplemental Figure 12C). Co-IP assays using nuclear fractions confirmed the interaction between endogenous TARBP2 and 53BP1 in 769-P and ACHN cells (Figure 5, C and D, and Supplemental Figure 12, D and E). Overexpressed FLAG-tagged TARBP2 or HA-tagged 53BP1 coimmunoprecipitated with endogenous 53BP1 or TARBP2 in Caki-1 cells, respectively (Supplemental Figure 12, F and G). Moreover, FLAG-tagged TARBP2 coimmunoprecipitated with HA-tagged 53BP1 and vice versa when coexpressed in Caki-1 cells (Figure 5E). 3D structured illumination microscopy demonstrated nuclear colocalization of TARBP2 and 53BP1 (Figure 5F and Supplemental Figure 12H).

We further investigated whether TARBP2 modulates the function of 53BP1. Knockdown or overexpression of *TARBP2* did not affect endogenous DNA damage levels (Supplemental Figure 12, I–K) or total 53BP1 protein abundance (Figure 5, G and H, and Supplemental Figure 12L). However, *TARBP2* knockdown significantly enhanced the formation of chromatin-associated 53BP1 nuclear bodies known as 53BP1 foci, while *TARBP2* overexpression exhibited the opposite effect (Figure 5, I and J, and Supplemental Figure 12M). Consistent with prior evidence that 53BP1 chromatin retention independent of extensive DNA damage enhances p53 chromatin binding and augments p53 responses (39), increased p53 chromatin accumulation correlated with 53BP1 enrichment under *TARBP2* knockdown, whereas an inverse relationship was observed under *TARBP2* overexpression (Figure 5, K and L, and Supplemental Figure 12N). Furthermore, the interaction between 53BP1 and p53 is critical for p53-mediated gene regulatory events (38). A proximity ligation assay confirmed that *TARBP2* knockdown disrupted formation of the TARBP2-53BP1 complex while increasing the number of 53BP1-p53 puncta, whereas TARBP2 overexpression exhibited the opposite effect (Figure 5M and Supplemental Figure 12, O and P). Sequential co-IP using nuclear extracts further clarified the interaction hierarchy. Initial TARBP2 immunoprecipitation identified 53BP1 as an interacting partner, while p53 was undetectable in the TARBP2-bound complex. Subsequent p53 immunoprecipitation of the TARBP2-unbound fraction demonstrated an association between 53BP1 and p53, with TARBP2 remaining absent from the precipitated complex (Figure 5N). These results suggest that TARBP2 interferes with 53BP1-p53 complex formation and suppresses chromatin accumulation of both 53BP1 and p53.

### TARBP2 restricts 53BP1 phase separation to attenuate p53 activation and upregulate B7-H3.

To identify the region of 53BP1 required for interaction with TARBP2, we transfected FLAG-tagged *TARBP2*-overexpressing Caki-1 cells with a series of plasmids expressing HA-tagged truncation mutants of 53BP1 (Figure 6A). Co-IP assays revealed that the oligomerization domain (OD), whose protein sequence is conserved across multiple species (Figure 6B), was required for stable interaction between TARBP2 and 53BP1 (Figure 6C). Additionally, TARBP2 did not affect chromatin accumulation of the 53BP1 fragment lacking the OD (Figure 6, D and E, and Supplemental Figure 13A). Previous studies have demonstrated that deletion of the OD disrupts liquid–liquid phase separation (LLPS) of 53BP1 (39–41) and that impaired LLPS compromises 53BP1-mediated p53 activation (39, 40). Therefore, we hypothesized that TARBP2 interacts with the OD of 53BP1 to suppress 53BP1 LLPS, thereby regulating the p53/B7-H3 axis.

To verify this hypothesis, mCherry-labeled *Arabidopsis thaliana* cryptochrome 2–based (Cry2-based) fusion constructs containing 53BP1 with or without the OD were used to evaluate blue light–induced 53BP1 oligomerization and formation of phase-separated condensates, termed optoDroplets (Figure 6F and Supplemental Figure 13B) (40, 42). Formation of 53BP1 optoDroplets strictly required the OD, and *TARBP2* perturbation influenced the assembly of OD-containing Cry2-mCherry-53BP1 into these phase-separated structures (Figure 6G and Supplemental Figure 13, C and D). Furthermore, p53 accumulation within OD-containing Cry2-mCherry-53BP1 optoDroplets increased in *TARBP2*-knockdown cells and decreased in *TARBP2*-overexpressing cells (Figure 6H and Supplemental Figure 13E). Fluorescence recovery after photobleaching analysis was performed to evaluate the LLPS capacity of GFP-tagged 53BP1 under *TARBP2* perturbation in live cells. Remarkably, *TARBP2* knockdown enhanced fluorescence recovery of GFP-53BP1 puncta after photobleaching, whereas its overexpression produced the opposite effect (Figure 6I and Supplemental Figure 13F).

We next assessed the role of LLPS in regulating the p53/B7-H3 axis. *TARBP2*-knockdown and control cells were treated with 1,6-hexanediol, an aliphatic alcohol commonly used to disrupt LLPS. 1,6-Hexanediol treatment restored the reduction in total and cell surface B7-H3 levels induced by *TARBP2* knockdown, accompanied by reversal of upregulation of the p53 downstream target p21 (Supplemental Figure 14, A–D). To determine whether TARBP2-mediated regulation of the p53/B7-H3 axis depends on 53BP1 OD-driven LLPS, Cry2-mCherry-53BP1 constructs with or without the OD were expressed in *TARBP2*-overexpressing Caki-1 cells, followed by blue light stimulation. The OD-containing construct reversed the TARBP2-induced increase in total and cell surface B7-H3 levels while also restoring p21 expression. This rescue effect was further enhanced upon blue light stimulation. In contrast, the OD-deficient construct showed no such effect (Supplemental Figure 14, E and F).

Collectively, these results demonstrate that TARBP2 restricts 53BP1 OD-driven LLPS, thereby impairing 53BP1-mediated p53 activation and ultimately promoting B7-H3 upregulation.

### Targeting TARBP2 sensitizes RCC to adoptive NK cell therapy and enhances ICB efficacy.

To evaluate the clinical relevance of TARBP2 in predicting responses to ICB therapy, we performed multiplex IHC on pretreatment tumor tissues from patients with RCC classified as responders or nonresponders to ICB therapy. TARBP2 expression was predominantly localized in pan-cytokeratin^+^ tumor cells, and significantly higher TARBP2 expression was observed in tumor cells from nonresponders compared with responders (Supplemental Figure 15, A and B). Furthermore, tumors from nonresponders showed elevated nuclear TARBP2 expression and increased B7-H3 expression, accompanied by reduced accumulation of CD3^+^ T cells and particularly CD3^–^CD56^+^ NK cells (Figure 7, A and B).

To investigate whether TARBP2 inhibition enhances NK cell–mediated antitumor activity in a clinically relevant human RCC model, immunodeficient NCG mice were engrafted with RCC patient-derived xenografts (PDXs) and subsequently received adoptive transfer of NK-92MI cells. Localized administration of *TARBP2*-targeting siRNA (si*TARBP2*) was conducted to abrogate *TARBP2* expression in PDX tumors (Figure 7C). Consistent with observations in the NCG Renca-derived tumor model (Figure 2, A–D), si*TARBP2* alone exerted negligible effects on tumor growth in the absence of NK-92MI cell transfer. Although NK-92MI cell monotherapy produced moderate antitumor effects, combination treatment with si*TARBP2* achieved the greatest suppression of PDX tumor growth (Figure 7, D–F). The expression levels of TARBP2 and B7-H3, along with apoptosis levels indicated by Cleaved Caspase-3 staining in PDX tumor tissues, were assessed by IHC (Figure 7G and Supplemental Figure 16, A and B). Additionally, flow cytometry analysis demonstrated that cotreatment with si*TARBP2* significantly increased the proportions of CD107a^+^, TNF-α^+^, and IFN-γ^+^ NK cells within PDX tumor microenvironments compared with NK-92MI monotherapy (Figure 7H and Supplemental Figure 16C).

To further determine whether TARBP2 inhibition synergizes with adoptive NK cell therapy to enhance ICB efficacy, we established orthotopic RCC tumors in immunocompetent BALB/c mice using luciferase-expressing Renca cells transfected with either *Tarbp2* knockdown or control vectors. Tumor-bearing mice were then assigned to treatment groups and received adoptive NK cell transfer alone or in combination with anti–PD-1, anti–B7-H3, or anti–PD-1 plus anti–B7-H3 antibodies. NK cells were labeled with PKH26 prior to transfer to distinguish transferred NK cells from endogenous murine NK cells within tumors (Figure 7I). Consistent with the NCG PDX model, *Tarbp2* knockdown combined with NK cell transfer significantly suppressed tumor growth. The addition of anti–PD-1 or anti–B7-H3 further improved tumor control, with dual blockade achieving the greatest therapeutic efficacy. Notably, *Tarbp2* knockdown significantly enhanced treatment efficacy across all treatment groups (Figure 7, J and K, and Supplemental Figure 17). No evident toxicity was observed, as indicated by body weight monitoring and histological examination of organ sections (Supplemental Figure 18, A and B). Flow cytometry analysis of orthotopic tumors showed that, among all groups receiving adoptive NK cell transfer, no significant differences were observed in the ratio of PKH26^+^ transferred NK cells to PKH26^–^ endogenous NK cells within the intratumoral NK cell population. However, *Tarbp2* knockdown significantly increased the proportion of NK cells among intratumoral immune cells and enhanced NK cell effector functions across all treatment groups. These effects were most pronounced in mice receiving *Tarbp2* knockdown together with adoptive NK cell transfer and dual checkpoint blockade (Figure 7L and Supplemental Figure 19, A and B).

Collectively, these findings demonstrate that TARBP2 inhibition sensitizes RCC to adoptive NK cell therapy, with further therapeutic enhancement achieved by concurrent ICB treatment.

## Discussion

In summary, we identified *TARBP2* through comprehensive bioinformatics analyses. *TARBP2* is highly expressed in malignant RCC cells and is associated with poor prognosis and ICB resistance in patients with RCC. Using a syngeneic murine cell line with *Tarbp2* expression modulation for in vivo experiments, we found that TARBP2 promotes tumor progression by reducing intratumoral accumulation and cytotoxicity of T cells and particularly NK cells. Mechanistically, nuclear TARBP2 in RCC cells binds to 53BP1 and inhibits its phase separation, suppressing 53BP1-dependent p53 activation and thereby promoting immune checkpoint *B7-H3* transcription. This process facilitates RCC immune escape. The biological and therapeutic implications of these findings were further explored.

TARBP2 is an RNA-binding protein with a structural framework that supports both RNA binding and protein–protein interactions. It contains 2 canonical type A double-stranded RNA-binding domains (dsRBDs) that bind dsRNA and 1 noncanonical type B dsRBD that lacks RNA-binding residues and instead mediates interactions with partner proteins (43). Previous studies have shown that TARBP2 binding can alter the function of interacting proteins. For example, TARBP2 physically interacts with MAVS and RIG-I, suppressing virus-induced MAVS-mediated IFN-β production to attenuate innate immune responses (11). Additionally, TARBP2 is broadly distributed in both the cytoplasm and nucleus of human cells (44), where it performs distinct biological functions. For example, in the cytoplasm, TARBP2 binds to Dicer and participates in miRNA processing (7), whereas in the nucleus, TARBP2 recruits the m^6^A methylation machinery to target transcripts, promoting intron retention and transcript degradation (9). In this study, we observed elevated nuclear TARBP2 expression in high-grade RCC tissues and in pretreatment samples from ICB nonresponders. Further mechanistic investigations revealed that TARBP2 regulates B7-H3 expression independently of its RNA-binding function through directly interacting with the OD of 53BP1 in the nucleus and modulating 53BP1 phase separation. These findings provide a better understanding of the oncogenic role of TARBP2. Nevertheless, the specific domain of TARBP2 involved in the interaction with the 53BP1 OD remains unclear. Given that the noncanonical type B dsRBD of TARBP2 primarily mediates protein–protein interactions, it is tempting to speculate that this domain may participate in binding to the 53BP1 OD. Since OD-mediated self-association is essential for higher-order assembly and phase separation of 53BP1, TARBP2 binding may either sterically interfere with 53BP1 oligomerization or induce conformational alterations that impair 53BP1 self-assembly. These possibilities warrant further structural and biochemical investigation. Moreover, the mechanisms responsible for TARBP2 nuclear accumulation in RCC cells remain to be clarified.

The tumor suppressor p53 functions as a stress-responsive transcription factor activated by diverse stimuli such as DNA damage, hypoxia, oncogenic signaling, and ribosomal stress. Upon activation, it orchestrates multiple cellular processes, including cell cycle arrest, DNA damage repair, cell death, and metabolic reprogramming (45). *TP53* is mutated in approximately 50% of human malignancies (46). Most *TP53* mutations are missense mutations that predominantly occur within the DNA-binding domain and impair the DNA-binding capacity of p53, while certain gain-of-function variants even exacerbate protumor inflammation and promote tumorigenesis (36). Despite harboring a WT *TP53* gene, p53 is functionally inactivated in many tumors through the overexpression of negative regulators, including MDM2 and MDM4 (47). Although RCC exhibits a relatively low *TP53* mutation rate, the WT p53 protein frequently exhibits functional impairment (48). This highlights the crucial need to identify additional molecular regulators of p53 activity in RCC to facilitate the development of effective therapeutic strategies. Previous studies have demonstrated that restoration of p53 activity enhances systemic antitumor immunity (49), including NK cell cytotoxicity (50). In this study, we utilized human RCC cell lines with the WT *TP53* gene and murine-derived Renca cells with WT status of p53 protein (51) to conduct in vitro and in vivo experiments. Our results suggest that TARBP2 inhibition in these RCC cells restores the function of p53 and enhances NK cell cytotoxicity. Thus, TARBP2 emerges as a promising target for drug development against RCC via p53 reactivation. Notably, our findings demonstrate that *B7-H3* transcriptional regulation is mediated by WT p53 rather than by common DNA-binding–deficient p53 mutants (R175H, R248W, and R273H). Further systematic investigation of whether distinct p53 mutants participate in TARBP2-mediated regulation of B7-H3 will help clarify the therapeutic potential of targeting TARBP2 across different *TP53* mutational backgrounds. From a clinical perspective, integrating *TP53* mutation status with variant allele frequency analysis may provide a more comprehensive assessment of p53 functional status and its reactivation potential, thereby enabling more personalized therapeutic strategies.

53BP1 was identified as an interactor of p53, situated at the crossroads of DNA double-strand break (DSB) repair and p53 signaling (38). Our study demonstrated that in RCC cells, TARBP2 inhibits 53BP1 phase separation by binding to its OD, without altering endogenous DNA damage levels. This suppression disrupts the assembly of p53 into 53BP1 phase-separated nuclear bodies and consequently attenuates the transcriptional regulatory function of p53. Previous studies have established that 53BP1 in tumor cells undergoes OD-dependent phase separation during DSB repair and heterochromatin binding (40, 41). In addition, 53BP1 phase separation can be influenced by various physical and chemical perturbations like temperature, salt concentration, pH, and osmotic pressure (40). Zhang et al. found that 53BP1 can form phase-separated puncta at heterochromatin independently of DSB repair, although direct 53BP1-p53 binding was not detected in unperturbed U-2 OS cells (41). Considering that tumor cells free from exogenous perturbations like ionizing radiation undergo DSBs due to replication stress, ROS accumulation, cytochrome *c* leakage from the mitochondria, and other factors (52), we propose that while TARBP2 in RCC cells does not cause additional DNA damage, it may impair 53BP1 phase separation and nuclear body formation at the repair sites of spontaneous DSBs, thereby suppressing p53 activation levels. This is supported by the perspective of Kilic et al. that 53BP1 phase separation integrates localized DNA damage recognition with global p53-dependent transcriptional responses (40). Although there is no evidence elucidating how assembly of p53 into 53BP1 phase-separated nuclear bodies facilitates p53-mediated gene regulatory functions, a possible explanation is that 3D genome folding in the nucleus enables interactions between p53 regulatory elements and 53BP1 nuclear bodies enriched with p53 proteins. However, whether TARBP2 influences the aggregation of 53BP1 and p53 at specific nuclear sites, such as DNA damage sites, p53 regulatory elements in the genome, or reservoir locations in the nucleus requires further in-depth investigation.

B7-H3 blockade suppresses tumor growth in various tumor models, although the dominant immune cell population responsible for this effect remains debated, with proposed roles for CD8^+^ T cells (53, 54), CD4^+^ T cells (55), and NK cells (15). Our study demonstrates that TARBP2-mediated upregulation of B7-H3 primarily impairs NK cell antitumor activity within the RCC microenvironment. One possible explanation is that the receptors mediating B7-H3 signaling remain incompletely defined and may exhibit context-dependent expression patterns across distinct tumor microenvironments. Specifically, in the RCC microenvironment, B7-H3 from RCC cells may primarily mediate interactions with NK cells. The limitation lies in the fact that our study did not directly examine how RCC-derived B7-H3 remodels the RCC immune microenvironment. TARBP2 in RCC cells may exhibit B7-H3–independent effects, as *Tarbp2* knockdown or overexpression in *B7h3*-knockout Renca-derived tumors still produced modest antitumor or protumor effects that did not reach statistical significance (Figure 4, M and N, and Supplemental Figure 10E). Moreover, a clinical trial has demonstrated the safety and antitumor efficacy of enoblituzumab (MGA271), a humanized Fc-engineered antibody targeting B7-H3, in various malignancies including RCC (56). Further investigation into the effects of B7-H3 blockade on the RCC immune microenvironment and mechanisms of therapeutic resistance may therefore improve treatment strategies for RCC, particularly in advanced cases.

Adoptive NK cell therapy is an immunotherapy involving the expansion or engineering of NK cells ex vivo, followed by infusion into patients to eliminate cancer cells. Adoptive NK cell therapy offers several advantages over adoptive T cell therapy, including no requirement for antigen-specific priming, efficacy against tumor cells with low mutation load, and absence of graft-versus-host disease (57). However, therapeutic efficacy in solid tumors remains limited due to impaired NK cell infiltration, which is attributed to dysregulated chemokine receptor expression or alterations in infiltration signals driven by the hypoxic and acidic tumor microenvironment (58). Additionally, NK cells rapidly lose effector functions after infiltration (26), partially due to immunosuppressive compounds like TGF-β and activin A (59), and oxidative stress (60) within the tumor microenvironment. Ongoing clinical trials (ClinicalTrials.gov NCT06318871 and NCT05703854) are evaluating adoptive NK cell therapy with cytokine support in advanced RCC. Further efforts are needed to enhance NK cell infiltration and sustain the cytolytic function of tumor-infiltrating NK cells. Our work demonstrates that TARBP2 inhibition enhances NK cell–mediated tumor killing both in vitro and in vivo, inhibits post-infiltration NK cell dysfunction, and improves the efficacy of adoptive NK cell therapy. ICB therapies, such as PD-1/PD-L1 blockade and B7-H3 blockade, can also enhance NK cell–mediated tumor killing (15, 61, 62). Our study demonstrates that both PD-1 blockade and B7-H3 blockade enhance the antitumor efficacy of TARBP2-targeted strategies combined with adoptive NK cell therapy. Other ICB therapies, including anti-TIGIT, anti–CTLA-4, and anti–LAG-3 therapies, may also have the potential to synergize with TARBP2-targeted strategies in combination with adoptive NK cell therapy and therefore warrant further investigation. TIGIT blockade is of particular interest because TIGIT serves as an important inhibitory checkpoint in NK cells (63).

Despite these promising findings, several limitations should be acknowledged. Certain ICB therapies, particularly anti–CTLA-4, are associated with substantial immune-related adverse events (64). Moreover, because TARBP2 modulates 53BP1, a central regulator of the DNA damage response in normal cells, systemic TARBP2 inhibition may cause off-tumor toxicity. Therefore, careful evaluation of the in vivo safety and tolerability of TARBP2 inhibition and TARBP2 inhibition–based combination therapies is required in future studies. In addition, emerging tumor-targeted delivery approaches, including antibody-drug conjugates and precision nanocarriers, may enhance tumor-selective drug delivery and reduce systemic toxicity (65, 66). Accordingly, integrating TARBP2-targeted strategies with such approaches may help minimize potential off-tumor toxicity. It should also be noted that although the murine B7-H3 blocking antibody clone MJ18 used in our Renca-derived orthotopic tumor model has been widely used in preclinical tumor studies (35, 54, 55), its specificity toward murine B7-H3 has recently been questioned (67). Therefore, we cannot exclude the possibility that the antitumor effect observed in this model may be influenced by the unresolved target specificity of MJ18. Future studies using independently validated murine B7-H3 blocking reagents are needed to further confirm the therapeutic contribution of B7-H3 blockade in RCC.

In conclusion, our study identified TARBP2 as a critical regulator of immune escape in RCC through the promotion of *B7-H3* transcription. This effect depends on TARBP2 binding to 53BP1 within the nucleus of RCC cells, thereby disrupting 53BP1 phase separation, inhibiting p53 activation, and ultimately promoting *B7-H3* transcription. Inhibition of TARBP2, in combination with adoptive NK cell transfer and ICB treatment, represents a promising therapeutic strategy for improving immunotherapeutic responses in RCC.

## Methods

Further details are provided in Supplemental Methods.

### Sex as a biological variable.

Our study examined samples from both male and female donors. No significant differences in sex distribution were observed between RCC patients with low and high TARBP2 expression, as shown in Supplemental Table 2. Therefore, sex was not considered as a biological variable. Male mice were used for in vivo experiments to ensure consistency and reduce experimental variability.

### Statistics.

Statistical analyses were performed using GraphPad Prism (version 9.5.1; RRID: SCR_002798) or R (version 4.4.1; RRID: SCR_001905). Statistical significance was determined using the tests specified in the figure legends. *P* < 0.05 was considered statistically significant.

### Study approval.

Human RCC tissue samples were obtained from The First Affiliated Hospital of Sun Yat-sen University. The study was approved by the Ethics Committee of The First Affiliated Hospital of Sun Yat-sen University (approval no. [2025]588), and written informed consent was obtained from all participants. Animal experiments were approved by the Institutional Animal Care and Use Committee of Sun Yat-sen University Cancer Center (approval no. L102012022080B).

### Data availability.

The raw RNA-seq and scRNA-seq data generated in this study have been deposited in the China National GeneBank Sequence Archive (https://db.cngb.org/cnsa/) under accession number CNP0009514. HPLC-MS/MS proteomics data have been deposited in the OMIX, China National Center for Bioinformation/Beijing Institute of Genomics, Chinese Academy of Sciences (https://ngdc.cncb.ac.cn/omix) database under accession number OMIX017103. Values for all data points are available in the Supporting Data Values file. All other data and reagents supporting the findings of this study are available from the corresponding authors upon reasonable request.

## Manuscript information

### Author contributions.

MC, ML, J Zhu, KL, GS, and ZT contributed equally to this work as co–first authors. The order of the co–first authors reflects the relative scope and weight of their contributions to the study. MC and ML performed the core experiments, analyzed the data, and led the preparation of the manuscript. J Zhu and KL made substantial contributions to experimental design and statistical methodology, while also participating in patient follow-up and sample collection. GS and ZT generated critical in vivo animal model data that validated the clinical relevance of the findings and assisted in figure preparation. MZ, LF, XZ, Jinchen Luo, WL, and LL assisted with experiments and data analysis. TY and YP assisted in experimental design and provided essential materials. QL, HL, and KH provided key materials. GS, LF, YP, KH, WC, and J Zhang provided funding support for the study. LZ, WC, Junhang Luo, and J Zhang conceived and designed the study, supervised the project, provided technical support, and critically revised the manuscript.

### Conflict of interest.

The authors have declared that no conflict of interest exists.

### Funding.

This study was supported by the following:

- National Natural Science Foundation of China (82473160 to Jiaxing Zhang, 82272862 and 82473417 to WC, 82503890 to GS, 82503496 to LF, and 82403968 to KH).
- Natural Science Foundation of Hunan Province (2026JJ70012 to LF).
- Natural Science Foundation of Jiangsu Province (BK20240342 to YP).
- Natural Science Foundation of Guangdong Province (2025A1515010988 to KH).
- Medical Scientific Research Foundation of Guangdong Province of China (A2025206 to LF).
- China Postdoctoral Science Foundation (2025M772215 to GS, 2025M782325 to LF, and 2023M730374 to YP).

## Supplementary Material

Supplemental data

Unedited blot and gel images

Supplemental table 1

Supplemental table 2

Supplemental table 3

Supplemental table 4

Supporting data values

## Figures and Tables

**Figure 1:**
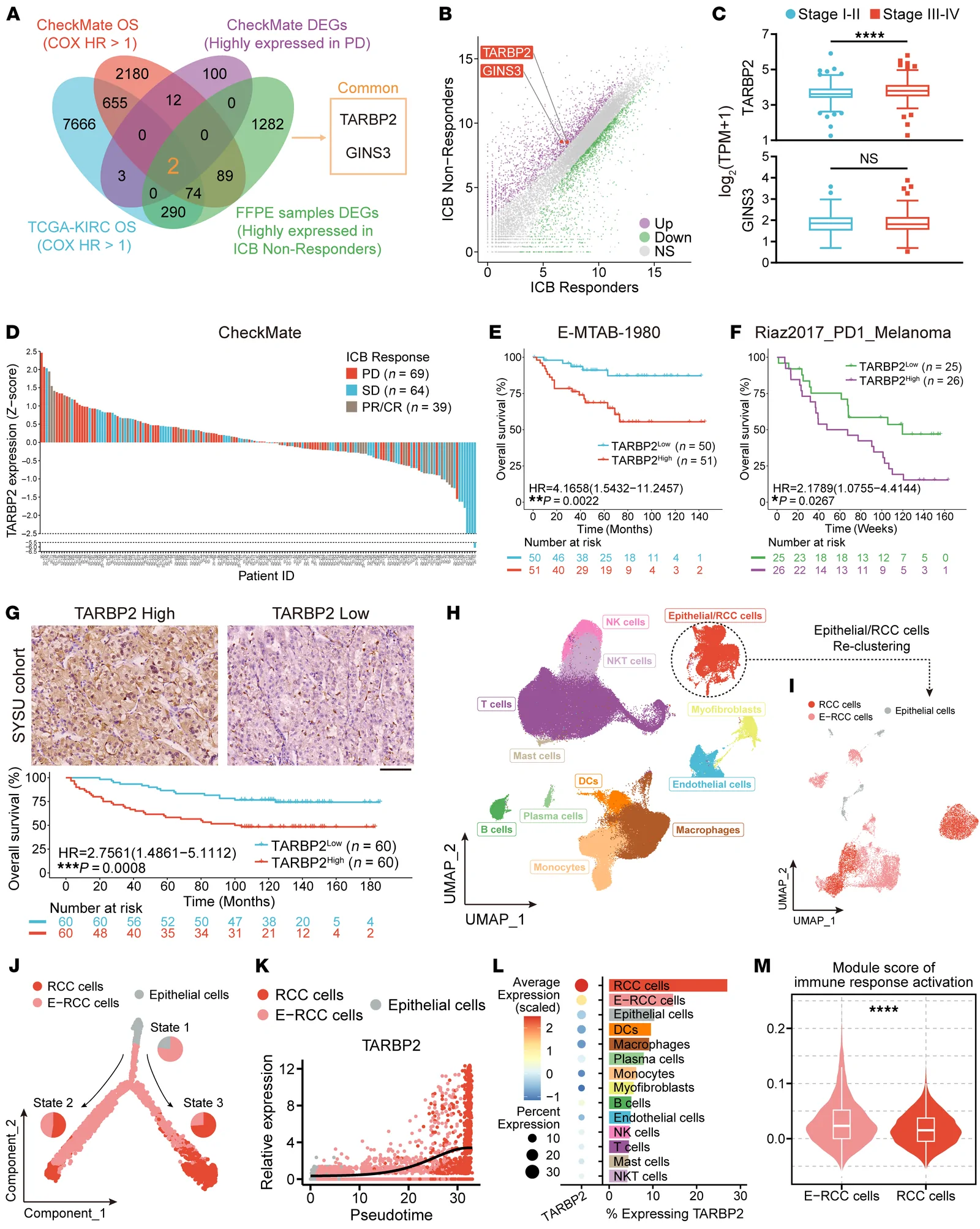
TARBP2 correlates with poor prognosis and ICB resistance in RCC and is predominantly expressed in malignant RCC cells. (**A**) Venn diagram depicting overlapped genes in data mining. DEGs, differentially expressed genes. (**B**) Scatterplot illustrating differentially expressed genes in RCC ICB pretreatment FFPE samples between ICB responders and nonresponders (*n* = 3 samples from different patients per group). (**C**) *TARBP2* (top) and *GINS3* (bottom) expression in low-stage (I–II, *n* = 323) and high-stage (III–IV, *n* = 206) RCC from the TCGA-KIRC cohort. Boxes represent the IQR, with the center line indicating the median. Whiskers extend to values within 1.5-fold of the IQR, and points beyond the whiskers are shown as outliers. (**D**) Waterfall plot of *TARBP2* expression (*z* score) across progressive disease (PD), stable disease (SD), partial response (PR), and complete response (CR) samples in the CheckMate cohort (*n* = 172 samples). (**E** and **F**) OS of RCC patients with low or high *TARBP2* expression in the E-MTAB-1980 cohort (**E**) and Riaz2017_PD1_Melanoma cohort (**F**). (**G**) Top: Representative IHC images of RCC tissues with low or high TARBP2 H-score in the SYSU cohort. Scale bar: 100 μm. Bottom: OS of RCC patients with low (*n* = 60) or high (*n* = 60) TARBP2 H-score in the SYSU cohort. (**H**) Uniform manifold approximation and projection (UMAP) of 192,626 cells classified into 12 cell populations. (**I**) UMAP of reclustered epithelial and RCC cells. (**J**) Differentiation trajectories from epithelial cells to E-RCC cells and subsequently to RCC cells. (**K**) Dynamic *TARBP2* expression over pseudotime. (**L**) *TARBP2* expression levels and proportions of *TARBP2*^+^ cells across cell types in all samples regardless of ICB treatment status. (**M**) Immune response activation scores of E-RCC and RCC cells. Statistics were analyzed by Mann-Whitney test in **C** and **M** and by log-rank test in **E** and **F**. ****P < 0.0001; **P < 0.01; *P < 0.05.

**Figure 2:**
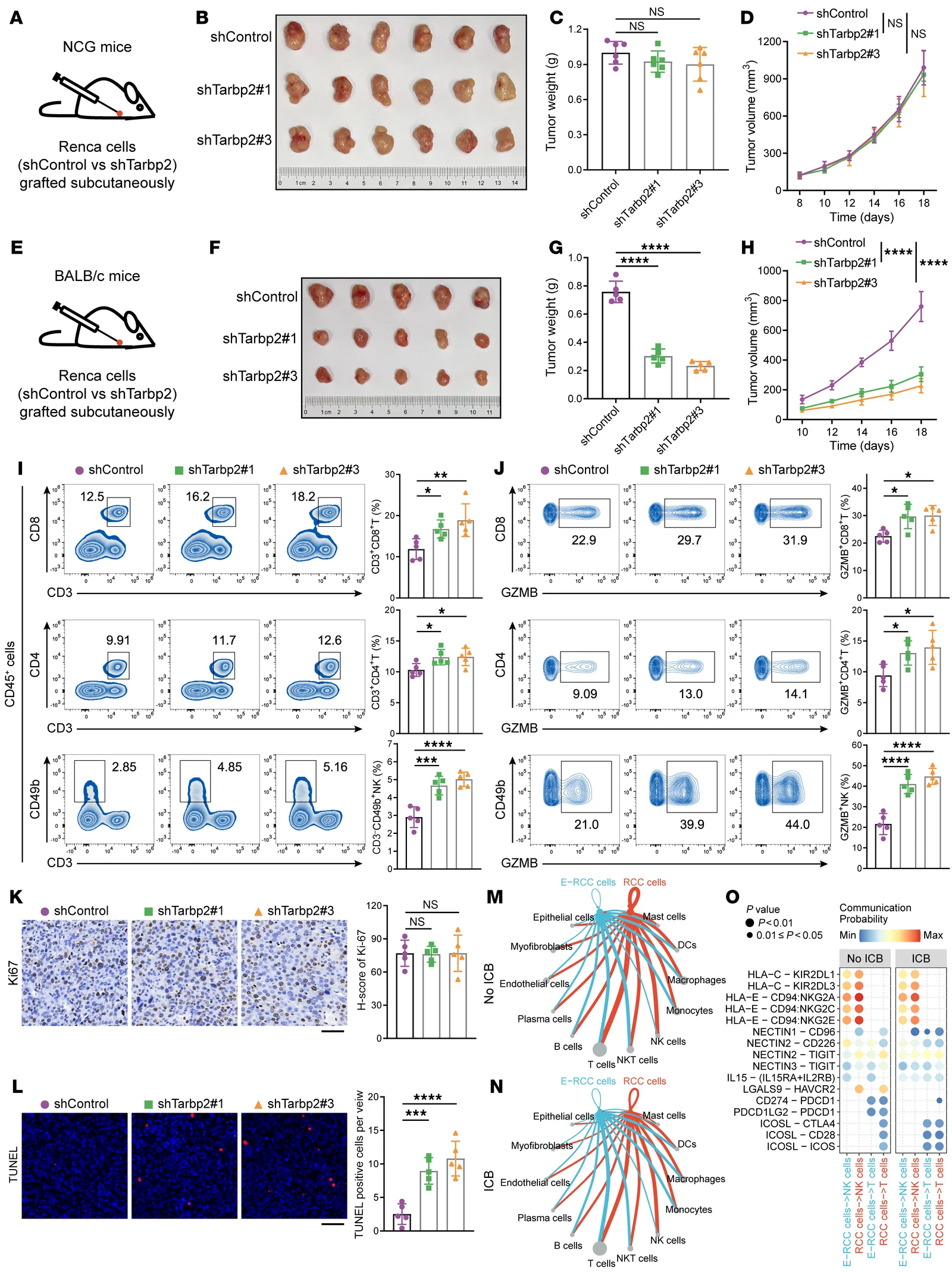
TARBP2 impairs immune-mediated tumor suppression in RCC. (**A**) Schematic of subcutaneous implantation of control or *Tarbp2*-knockdown Renca cells into NCG mice. (**B**–**D**) Tumor images (**B**), weights (**C**), and growth curves (**D**) of the indicated groups in NCG mice (*n* = 6 mice per group). (**E**) Schematic of subcutaneous implantation of control or *Tarbp2*-knockdown Renca cells into BALB/c mice. (**F**–**H**) Tumor images (**F**), weights (**G**), and growth curves (**H**) of the indicated groups in BALB/c mice (*n* = 5 mice per group). (**I**) Proportions of CD8^+^ T cells, CD4^+^ T cells, and CD3^–^CD49b^+^ NK cells in CD45^+^ cells from the indicated Renca-derived tumors in BALB/c mice (*n* = 5 mice per group). (**J**) Proportions of granzyme B^+^ (GZMB) cells in CD8^+^ T cells, CD4^+^ T cells, and NK cells from the indicated Renca-derived tumors in BALB/c mice (*n* = 5 mice per group). (**K** and **L**) Cell proliferation and apoptosis in tumor sections from BALB/c mice, as assessed by Ki67 (**K**) and TUNEL (**L**) staining, respectively (*n* = 5 mice per group). Scale bars: 50 μm. (**M** and **N**) The potential intercellular communication based on the calculation of interactive ligands-receptors between RCC/E-RCC cells and other cells in ICB-untreated (**M**) and ICB-treated (**N**) patient samples. Lines link cell types expressing the cognate receptors or ligands. Line thickness represents the inferred communication strength. (**O**) Dot plot showing immune checkpoint–related ligand-receptor pairs between RCC/E-RCC cells and T/NK cells. All data are presented as mean ± SD. Statistics were analyzed by 1-way ANOVA followed by Dunnett’s multiple-comparison test for comparisons with the control group in **C**, **G**, and **I**–**L** and by 2-way ANOVA followed by Bonferroni’s multiple-comparison test in **D** and **H**. ****P < 0.0001; ***P < 0.001; **P < 0.01; *P < 0.05.

**Figure 3:**
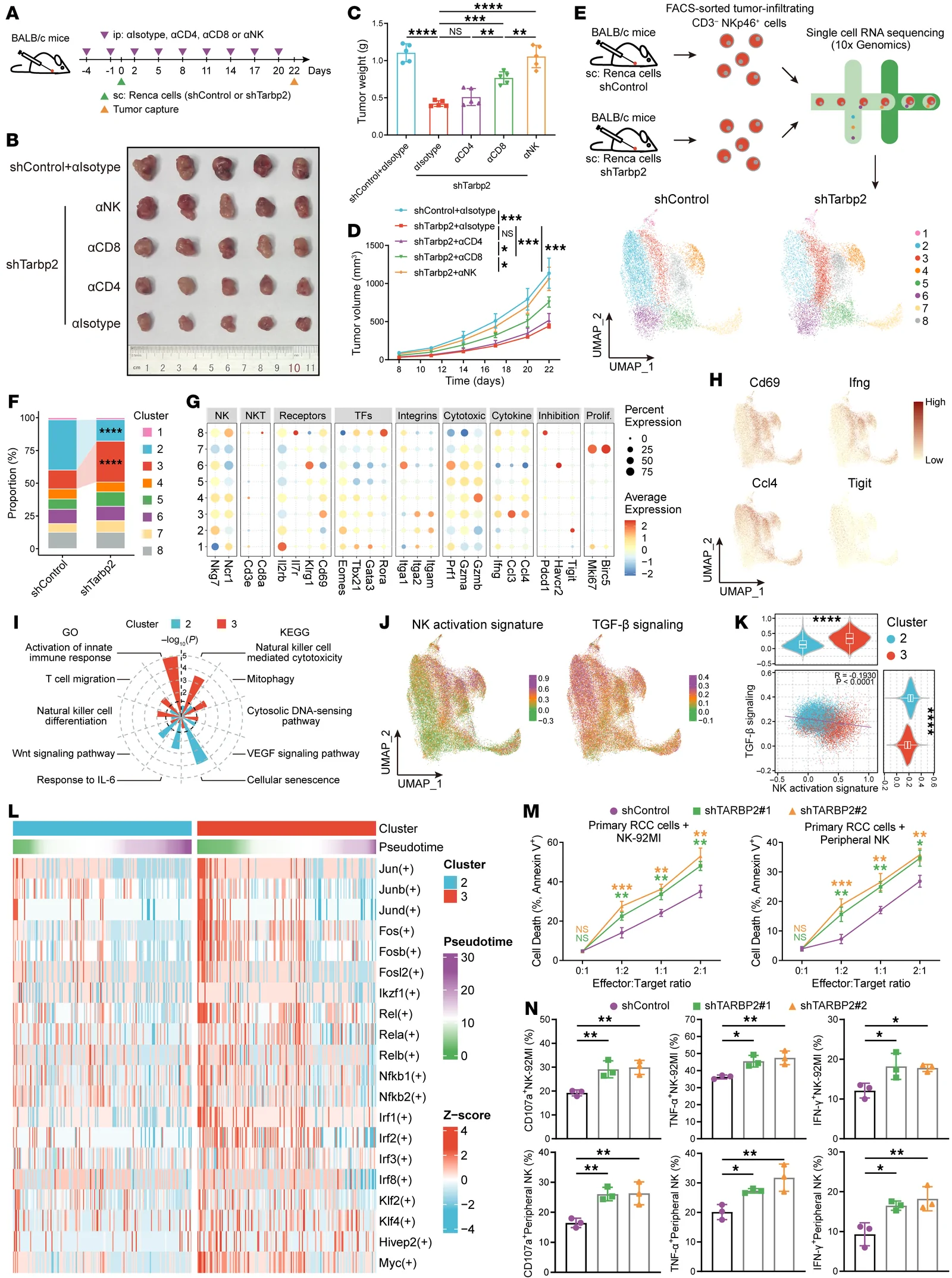
TARBP2 suppresses NK cell–mediated antitumor immunity in RCC. (**A**) Experimental design of Renca-derived tumors with or without *Tarbp2* knockdown in BALB/c mice treated with isotype control, CD4^+^ T cell–depleting antibody (αCD4), CD8^+^ T cell–depleting antibody (αCD8), or NK cell–depleting antibody (αNK). (**B**–**D**) Tumor images (**B**), weights (**C**), and growth curves (**D**) (*n* = 5 mice per group). (**E**) Workflow for isolating CD3^–^NKp46^+^ cells from Renca-derived tumors for scRNA-seq. (**F**) Proportions of clusters in *Tarbp2*-knockdown versus control tumors. (**G**) Dot plots of key marker genes defining clusters from FACS-sorted CD3^–^NKp46^+^ cells, including NK/NKT cell markers, surface receptors, transcription factors (TFs), integrins, cytotoxic molecules, cytokines, inhibitory molecules, and proliferation markers. (**H**) Expression of *Cd69*, *Ifng*, *Ccl4*, and *Tigit*. (**I**) Gene Ontology (GO) and KEGG enrichment analyses in clusters 2 and 3. (**J**) NK activation and TGF-β signaling signature scores across clusters. (**K**) Comparison and correlation of the NK activation and TGF-β signaling signature scores in clusters 2 and 3. (**L**) Heatmap showing transcription factor regulon activity associated with NK cell activation and proliferation over pseudotime. (**M**) Apoptotic rates (%, annexin V^+^) of primary RCC cells after incubation with NK-92MI or peripheral NK cells at different E/T ratios for 8 h (*n* = 3 independent experiments). (**N**) Proportions of CD107a^+^, TNF-α^+^, and IFN-γ^+^ NK cells after incubation with primary RCC cells (E/T = 1:1, 8 h; *n* = 3 independent experiments). All data are presented as mean ± SD. Statistics were analyzed by 1-way ANOVA followed by Tukey’s multiple-comparison test in **C**, by 2-way ANOVA followed by Bonferroni’s multiple-comparison test in **D**, by χ^2^ test in **F**, by Mann-Whitney test for violin plots and Spearman’s correlation for scatterplot in **K**, and by 1-way ANOVA followed by Dunnett’s multiple-comparison test in **M** and **N**. ****P < 0.0001; ***P < 0.001; **P < 0.01; *P < 0.05.

**Figure 4:**
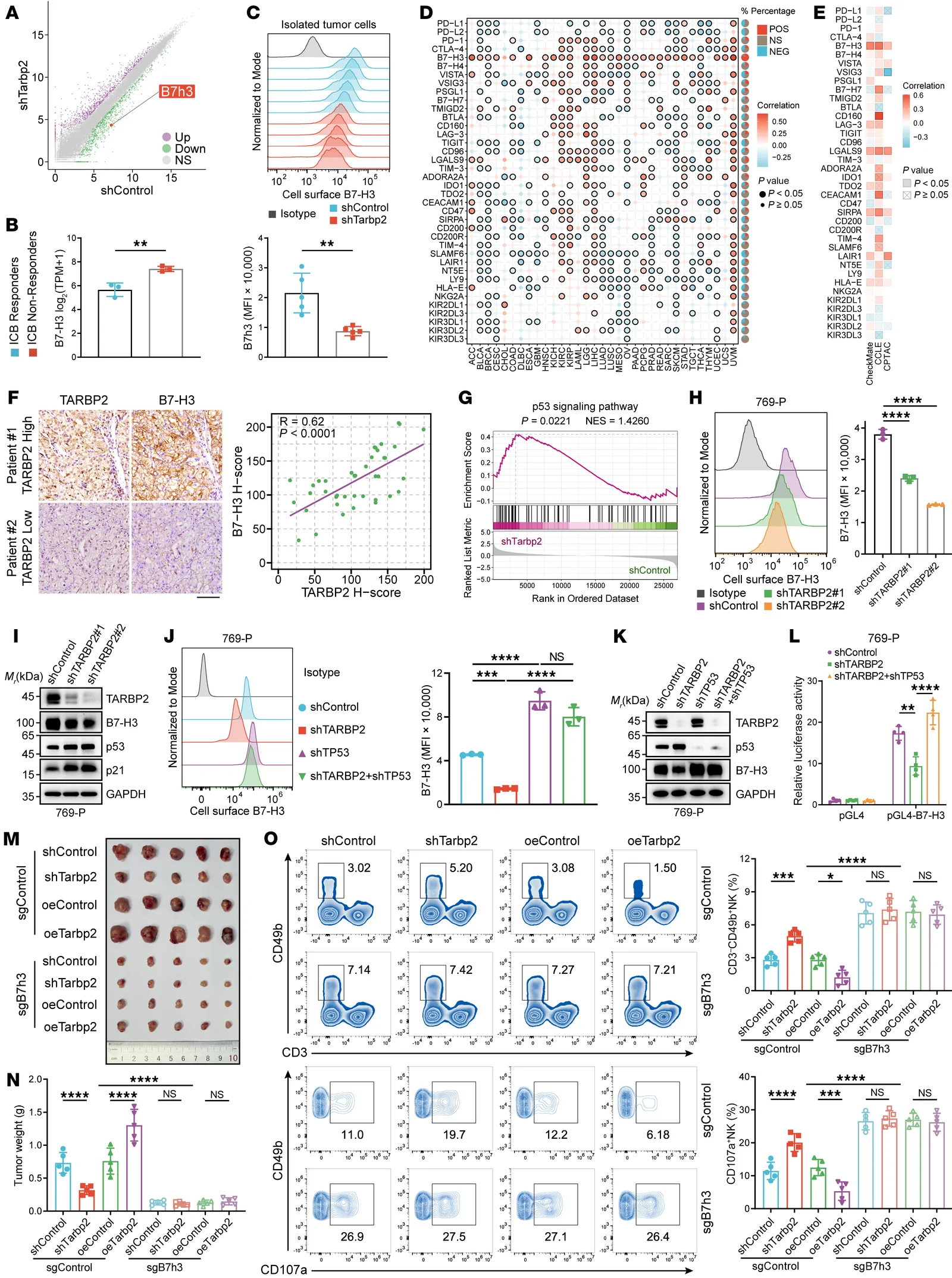
TARBP2/p53/B7-H3 axis regulates NK cell antitumor function in RCC. (**A**) Differentially expressed genes between tumor cells isolated from sh*Tarbp2* and control Renca-derived tumors (*n* = 5 mice/group). (**B**) *B7-H3* expression in RCC ICB pretreatment FFPE samples from responders and nonresponders (*n* = 3 patients/group). (**C**) Cell surface B7-H3 on isolated tumor cells (*n* = 5 mice/group). (**D** and **E**) Spearman’s correlations between TARBP2 and 38 common immune checkpoint genes across 33 TCGA cancer types (**D**) or RCC-related datasets (**E**). POS, positive; NEG, negative. (**F**) Representative IHC images of RCC tissues stained for TARBP2 and B7-H3. Scale bar: 100 μm (left). Spearman’s correlation between TARBP2 and B7-H3 H-scores (right; *n* = 40 samples). (**G**) GSEA of the p53 signaling pathway in isolated tumor cells (*n* = 5 mice/group). (**H**) Cell surface B7-H3 levels in *TARBP2*-knockdown and control 769-P cells (*n* = 3 independent experiments). (**I**) Total B7-H3, p53, and p21 protein levels in *TARBP2*-knockdown and control 769-P cells. GAPDH served as loading control. (**J** and **K**) Cell surface (**J**) and total (**K**) B7-H3 levels in *TP53*-knockdown 769-P cells with *TARBP2* knockdown or control. (**L**) *B7-H3* promoter–driven reporter activity in 769-P cells under control, *TARBP2*-knockdown, or *TARBP2*/*TP53*–co-knockdown conditions (*n* = 4 independent experiments). Relative luciferase activities were normalized to vector control samples. (**M** and **N**) Tumor weights (**M**) and growth curves (**N**) (*n* = 5 mice/group). (**O**) Proportions of CD3^–^CD49b^+^ NK cells within CD45^+^ cells and frequencies of CD107a^+^ cells among NK cells in indicated tumors (*n* = 5 mice/group). All data are presented as mean ± SD. Statistics were analyzed by unpaired 2-tailed Student’s *t* test in **B** and **C**, by 1-way ANOVA followed by Dunnett’s multiple-comparison test in **H**, by 1-way ANOVA followed by Tukey’s multiple-comparison test in **J** and **L**, and by 2-way ANOVA followed by Tukey’s multiple-comparison test in **N** and **O**. ****P < 0.0001; ***P < 0.001; **P < 0.01; *P < 0.05.

**Figure 5:**
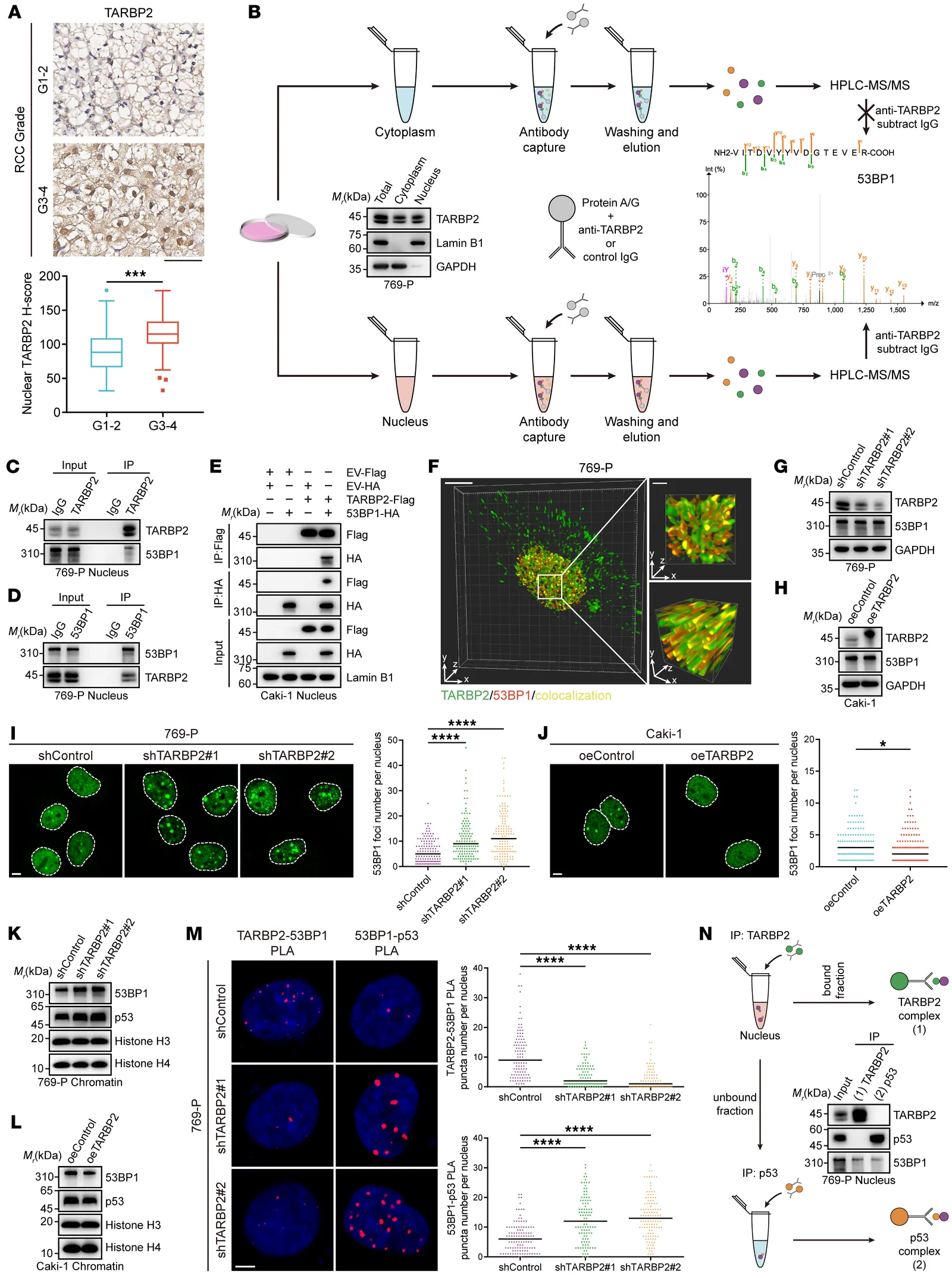
TARBP2 disrupts 53BP1-p53 complex formation and chromatin accumulation. (**A**) Top: Representative IHC staining of TARBP2. Scale bar: 50 μm. Bottom: H-score of nuclear TARBP2 in low- and high-grade RCC tissues from the SYSU cohort (*n* = 120 samples). Boxes represent the IQR, center lines indicate the median, whiskers extend to 1.5-fold of the IQR, and dots indicate outliers. (**B**) Workflow of nucleocytoplasmic separation, co-IP, and mass spectrometry identifying nuclear TARBP2–53BP1 interaction. (**C**–**E**) Co-IP assays showing interaction between endogenous TARBP2 and 53BP1 in 769-P cells (**C** and **D**) and between overexpressed TARBP2-FLAG and 53BP1-HA in Caki-1 cells (**E**). EV, empty vector. (**F**) 3D structured illumination microscopy rendering of 769-P cells stained to indicate TARBP2 (green) and 53BP1 (red) colocalization (yellow). Scale bars: 5 μm and 1 μm (insets). The *x*–*y* plane represents lateral cross-sectional views parallel to the substrate surface, and the *z* axis indicates axial dimension perpendicular to the substrate surface. (**G** and **H**) Total 53BP1 levels in 769-P (**G**) or Caki-1 (**H**) cells. GAPDH served as loading control. (**I** and **J**) Representative confocal images (left) and quantification of 53BP1 foci (right) in 769-P (**I**) or Caki-1 (**J**) cells (*n* > 120 nuclei/group). Dashed lines outline nuclei. Scale bars: 5 μm. (**K** and **L**) 53BP1 and p53 levels in chromatin fractions of 769-P (**K**) or Caki-1 (**L**) cells. Histone H3 and H4 served as chromatin loading controls. (**M**) Representative images (left) and quantification (right) of endogenous TARBP2-53BP1 and 53BP1-p53 interactions in 769-P cells (*n* > 100 nuclei/group). DAPI (blue) marks nuclei, and red dots indicate positive signals. Scale bar: 5 μm. (**N**) Sequential co-IP assays demonstrating distinct endogenous TARBP2-53BP1 and 53BP1-p53 complexes. Solid lines indicate medians in **I** (right), **J** (right), and **M** (right). Statistics were analyzed by unpaired 2-tailed Student’s *t* test in **A**, by Kruskal-Wallis test followed by Dunn’s multiple-comparison test in **I** and **M**, and by Mann-Whitney test in **J**. ****P < 0.0001; ***P < 0.001; *P < 0.05.

**Figure 6:**
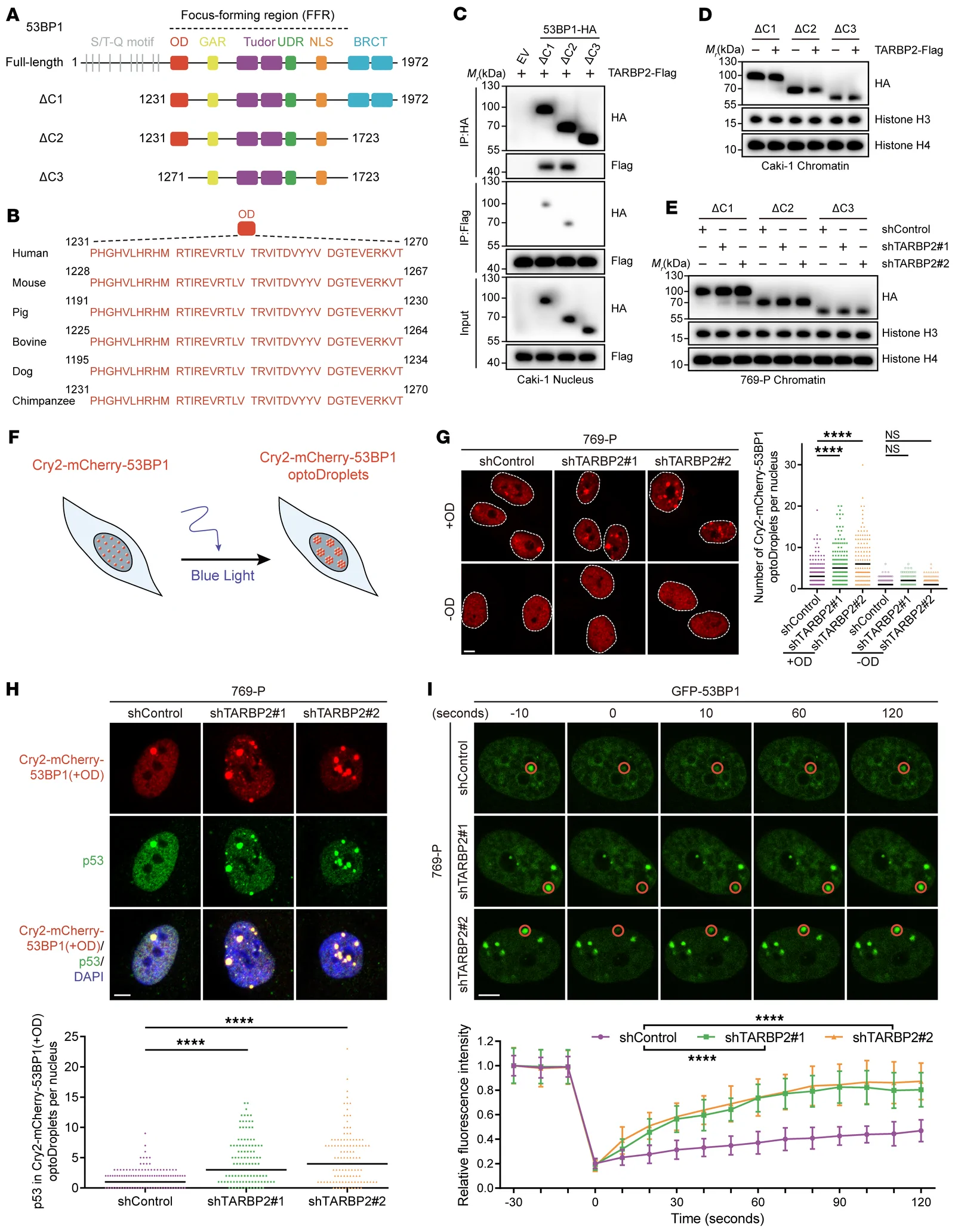
TARBP2 inhibits 53BP1 oligomerization-driven LLPS. (**A**) Schematic of 53BP1 truncation mutants. (**B**) Cross-species conservation of the 53BP1 OD protein sequence. (**C**) Co-IP assays showing the interactions between overexpressed TARBP2-FLAG and indicated 53BP1 truncation mutants in the nuclei of Caki-1 cells. (**D** and **E**) Immunoblot analysis of 53BP1 truncation mutants in chromatin fractions from Caki-1 cells with *TARBP2* overexpression or control (**D**) and 769-P cells with *TARBP2* knockdown or control (**E**). Histone H3 and H4 served as chromatin loading controls. (**F**) Schematic of optoDroplet formation of Cry2-mCherry-53BP1 fusion proteins with or without the OD. (**G**) Representative images (left) and quantification (right) of optoDroplet formation of Cry2-mCherry-53BP1 fusion proteins with or without the OD in 769-P cells with *TARBP2* knockdown or control treatment (*n* > 120 nuclei per group). Dashed lines outline nuclei. Scale bar: 5 μm. (**H**) Cry2-mCherry-53BP1 (containing the OD) optoDroplet induction followed by p53 (green) and DAPI (blue) staining in 769-P cells with *TARBP2* knockdown or control treatment (*n* > 100 nuclei per group). Scale bar: 5 μm. (**I**) Representative images (top) and quantification (bottom) of fluorescence recovery after photobleaching analysis of GFP-53BP1 puncta in 769-P cells with *TARBP2* knockdown or control treatment (*n* = 12 events from independent cells per group). Scale bar: 5 μm. Red circles indicate bleached puncta. Time zero (0 second) on the *x* axis marks the execution of photobleaching. Solid lines indicate medians in **G** (right) and **H** (bottom). Data are presented as mean ± SD in **I** (bottom). Statistics were analyzed by Kruskal-Wallis test followed by Dunn’s multiple-comparison test in **G** and **H** and by 2-way ANOVA followed by Bonferroni’s multiple-comparison test in **I**. ****P < 0.0001.

**Figure 7:**
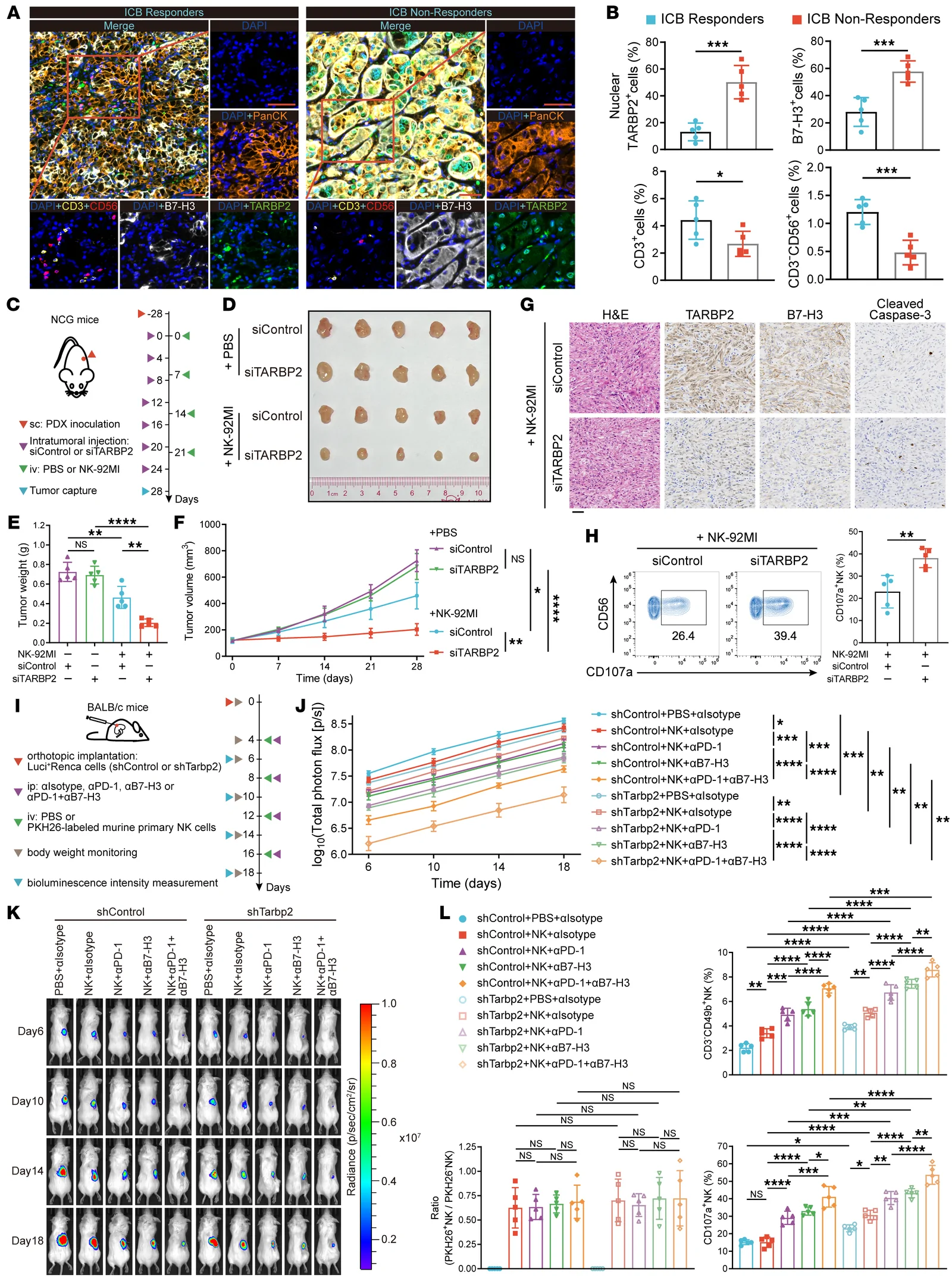
Targeting TARBP2 sensitizes RCC to adoptive NK cell therapy and ICB therapy. (**A**) Representative multiplex IHC staining of pan-cytokeratin (PanCK), TARBP2, B7-H3, CD3, CD56, and DAPI in RCC samples from ICB responders and nonresponders. Scale bars: 50 μm and 50 μm (insets). (**B**) Quantification of nuclear TARBP2^+^, B7-H3^+^, CD3^+^, and CD3^–^CD56^+^ cells in RCC samples from ICB responders and nonresponders (*n* = 5 patients/group). (**C**) Illustration of the RCC PDX model in immunodeficient NCG mice treated with adoptive NK cell transfer and siRNA at the indicated time points. (**D**–**F**) Tumor images (**D**), weights (**E**), and growth curves (**F**) in the indicated PDX groups (*n* = 5 mice/group). (**G**) Representative PDX tumor sections from NCG mice treated with siRNA and adoptive NK-92MI cell transfer stained with H&E, TARBP2, B7-H3, and Cleaved Caspase-3. Scale bar: 50 μm. (**H**) Proportion of CD107a^+^ cells among CD3^–^CD56^+^ NK cells in PDX tumors (*n* = 5 mice/group). (**I**) Schematic of Renca-derived orthotopic tumors in BALB/c mice treated with adoptive NK cell transfer, anti–PD-1 antibody, and anti–B7-H3 antibody at the indicated time points. (**J** and **K**) Quantification of bioluminescence intensity (**J**) and representative bioluminescence images (**K**) of orthotopic tumors from the indicated groups (*n* = 5 mice/group). (**L**) Proportions of CD3^–^CD49b^+^ NK cells among CD45^+^ cells, CD107a^+^ cells among NK cells, and the ratio of PKH26^+^ transferred NK cells to PKH26^−^ endogenous NK cells in orthotopic tumors from the indicated groups (*n* = 5 mice/group). All data are presented as mean ± SD. Statistics were analyzed by unpaired 2-tailed Student’s *t* test in **B** and **H**, by 1-way ANOVA followed by Tukey’s multiple-comparison test in **E** and **L**, and by 2-way ANOVA followed by Bonferroni’s multiple-comparison test in **F** and **J**. ****P < 0.0001; ***P < 0.001; **P < 0.01; *P < 0.05.

## Version 1 11/02/2026

Electronic publication

## Version 2 11/10/2026

Corrected grammatical errors in the abstract, Introduction, and Discussion sections.
