# Supplementary material for: TARBP2 facilitates renal cell carcinoma immune escape by suppressing 53BP1 phase separation to upregulate B7-H3
Source: J Clin Invest. 2026 Nov 2;136(21):e199525. doi: 10.1172/JCI199525 (PMC13722402; doi:10.1172/JCI199525)

Full unedited blot for Figure 4I

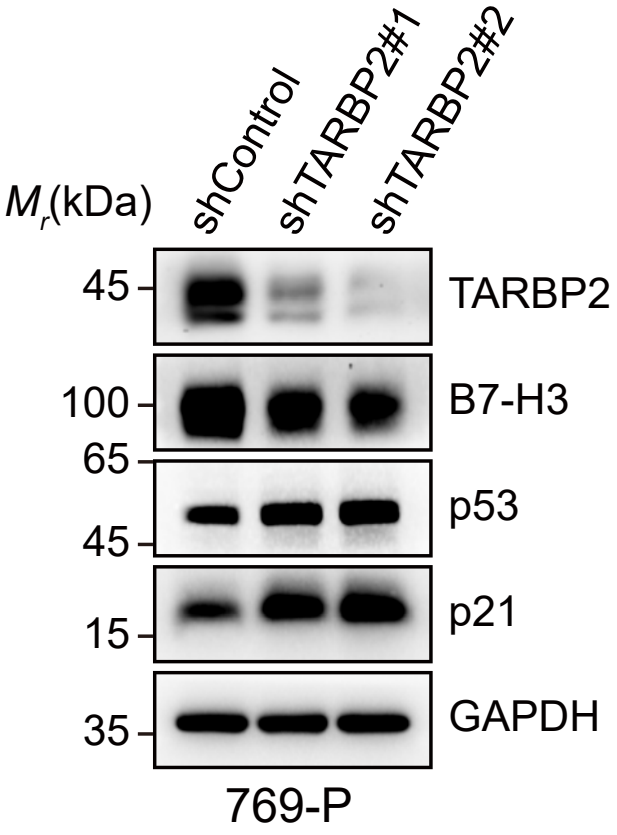

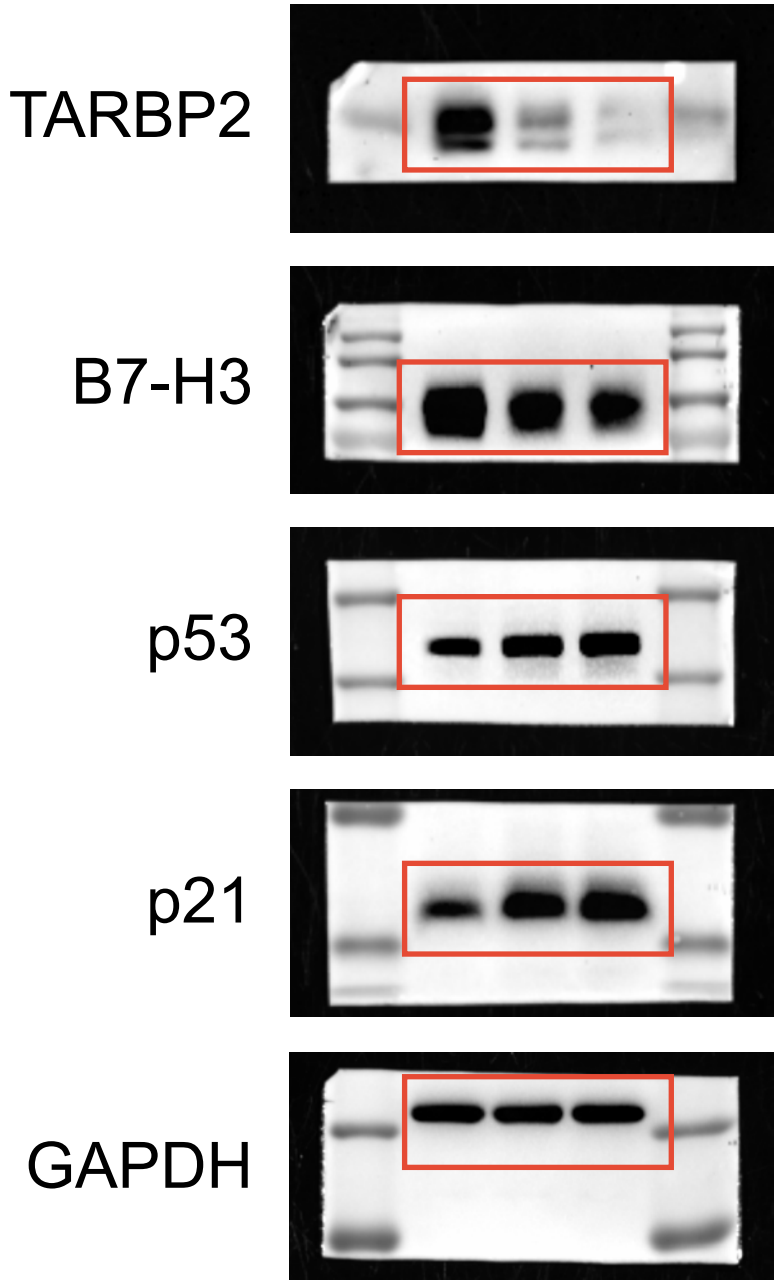

Full unedited blot for Figure 4K

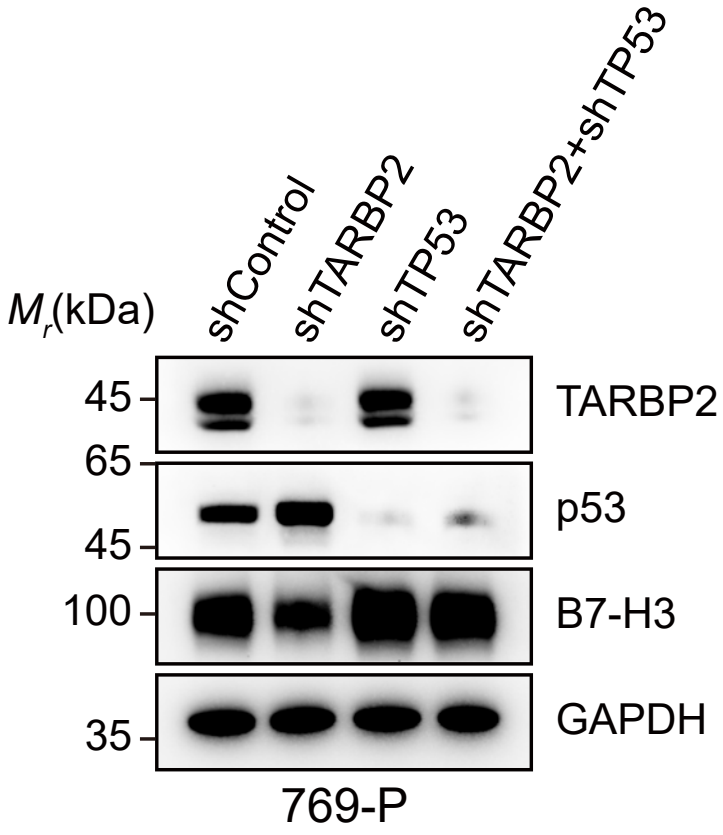

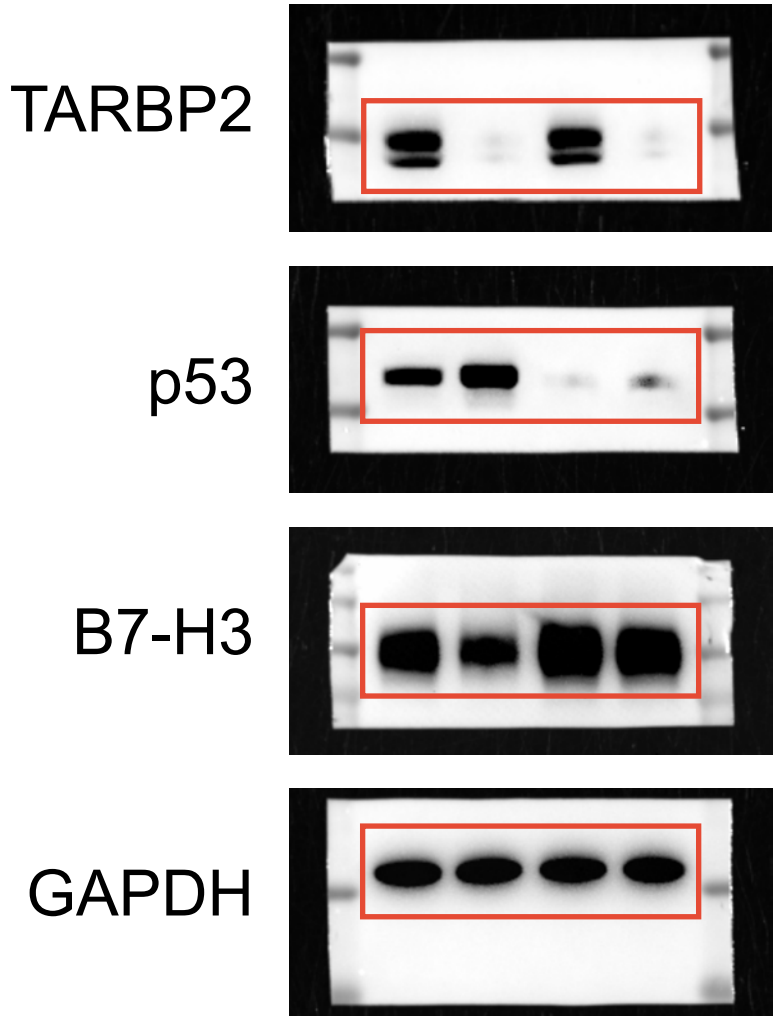

Full unedited blot for Figure 5B

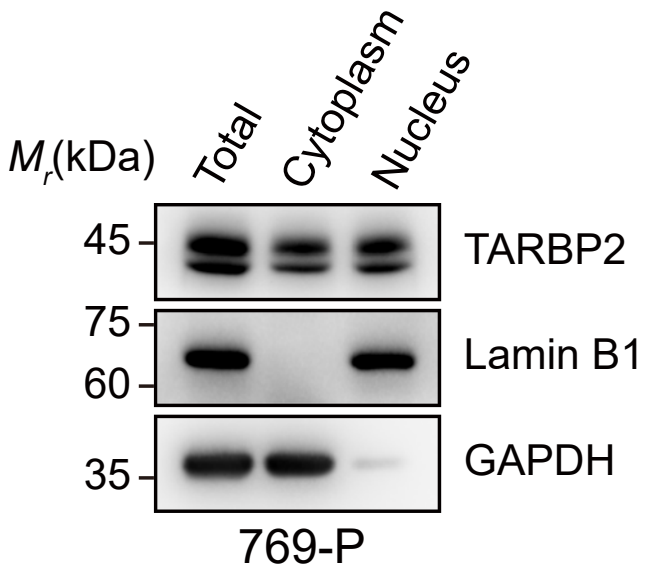

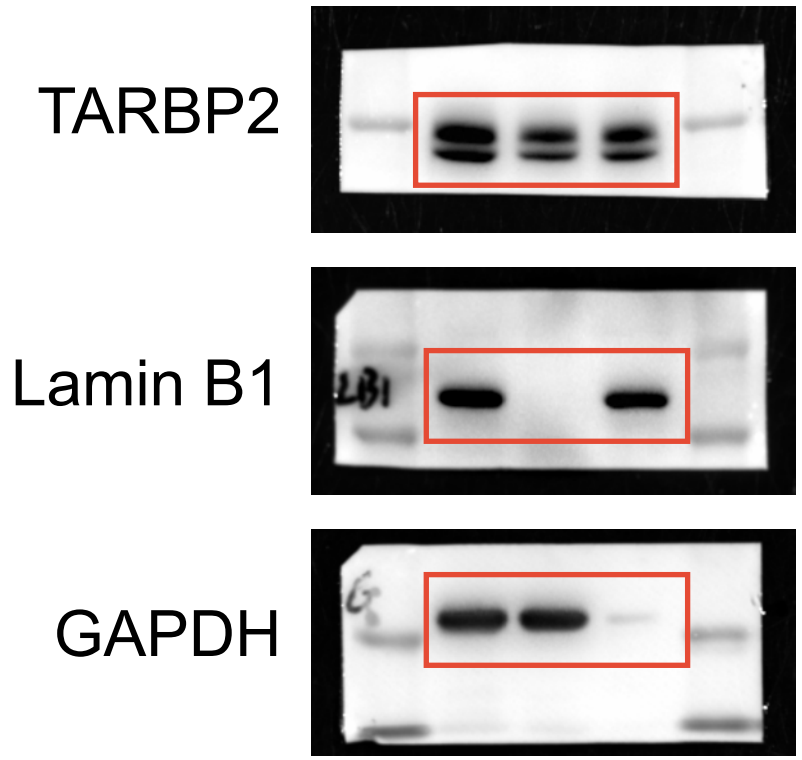

Full unedited blot for Figure 5C

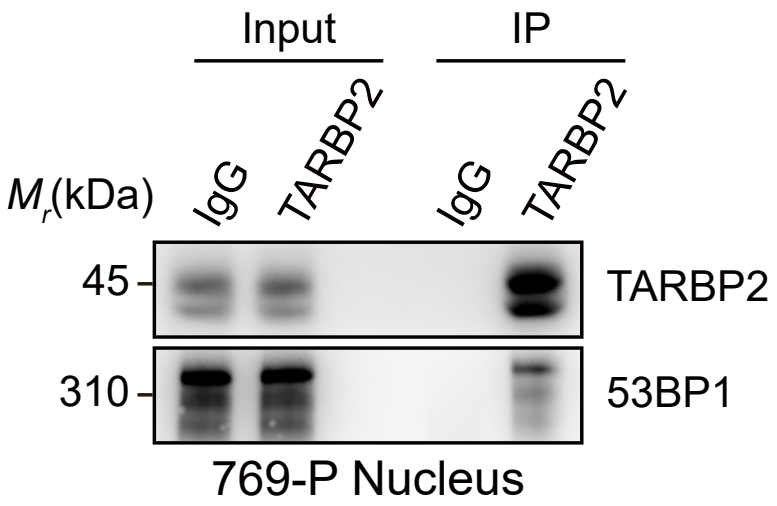

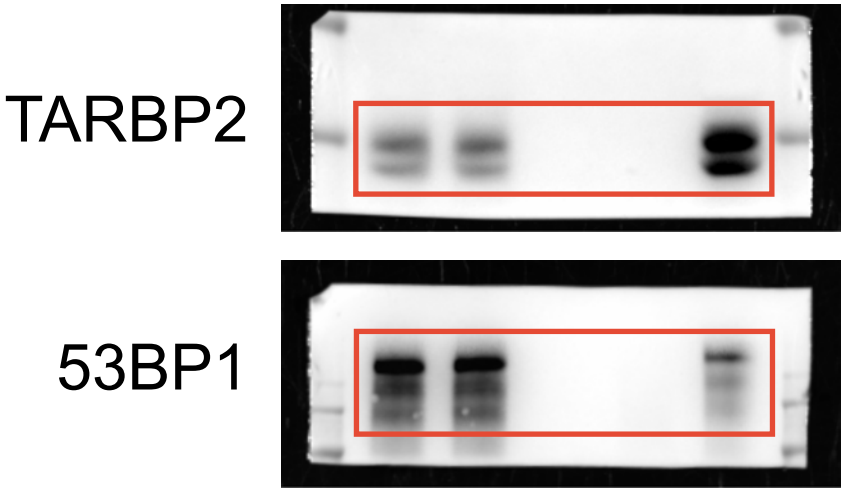

Full unedited blot for Figure 5D

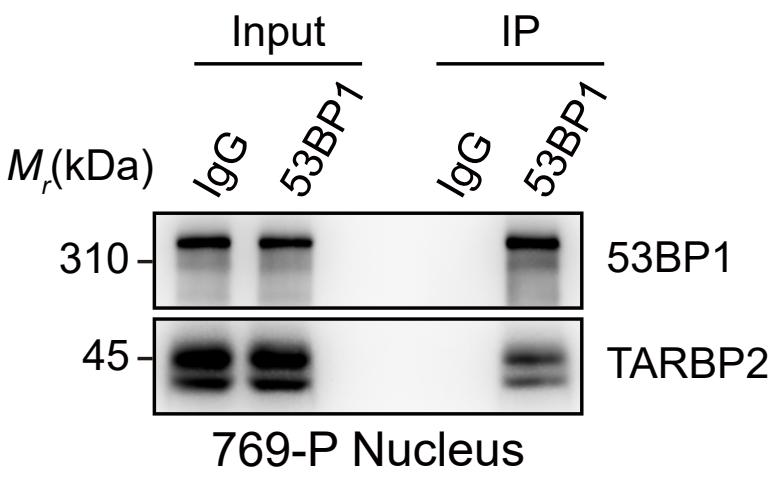

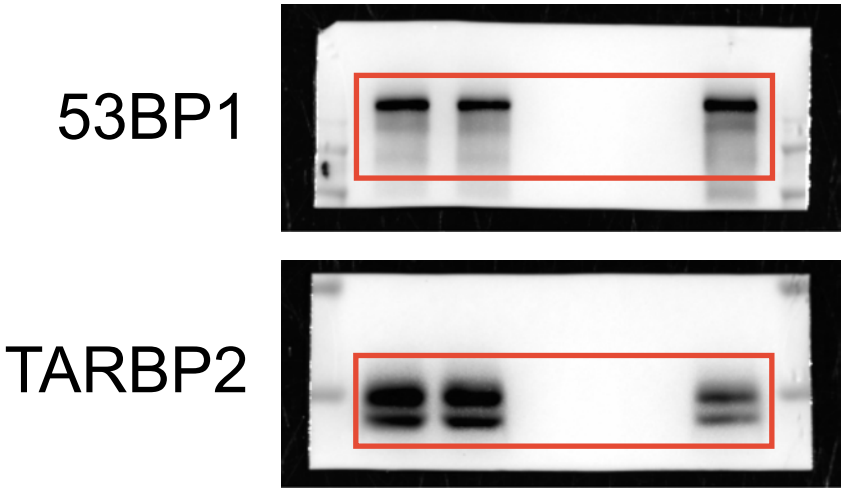

Full unedited blot for Figure 5E

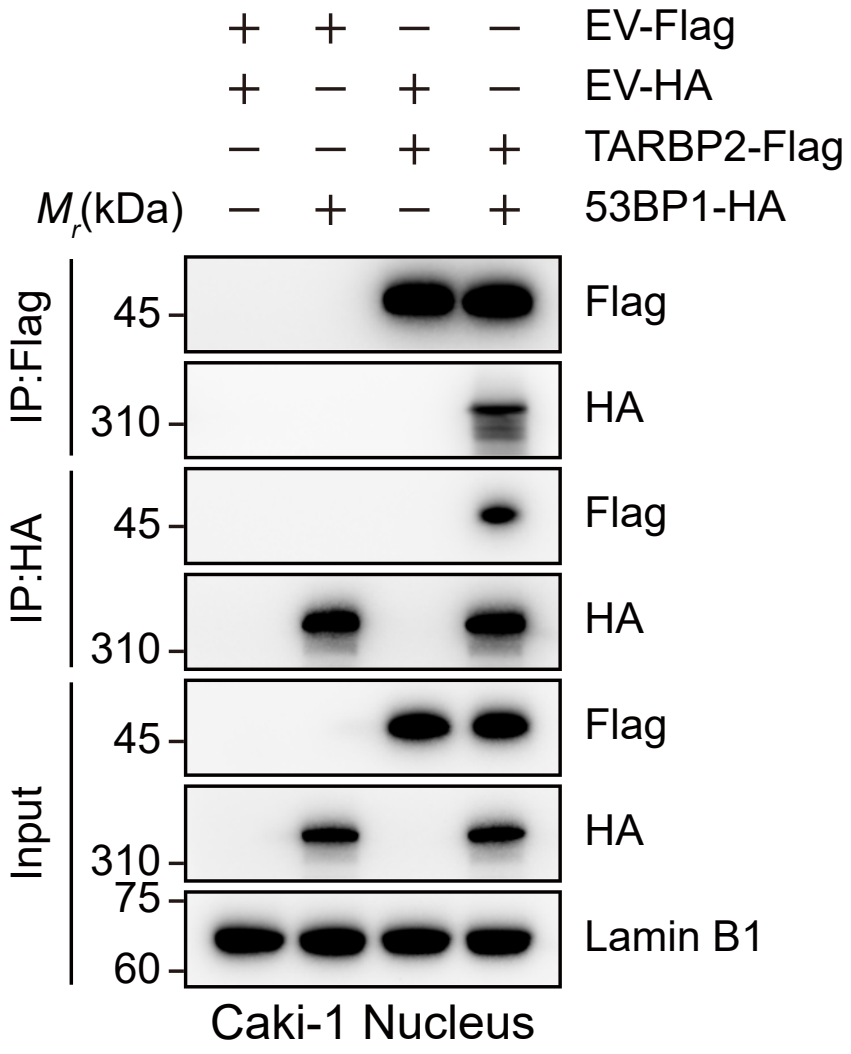

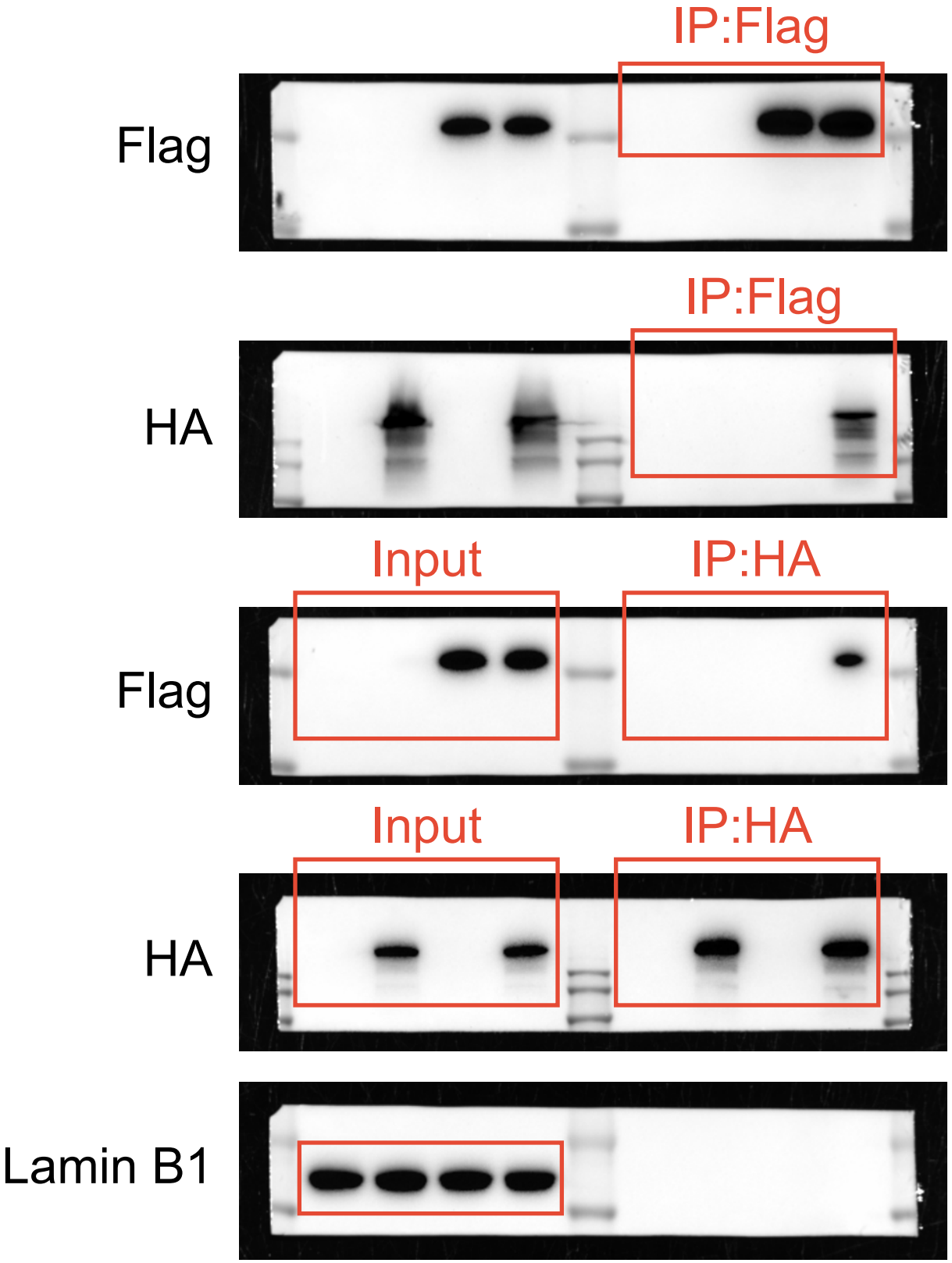

Full unedited blot for Figure 5G

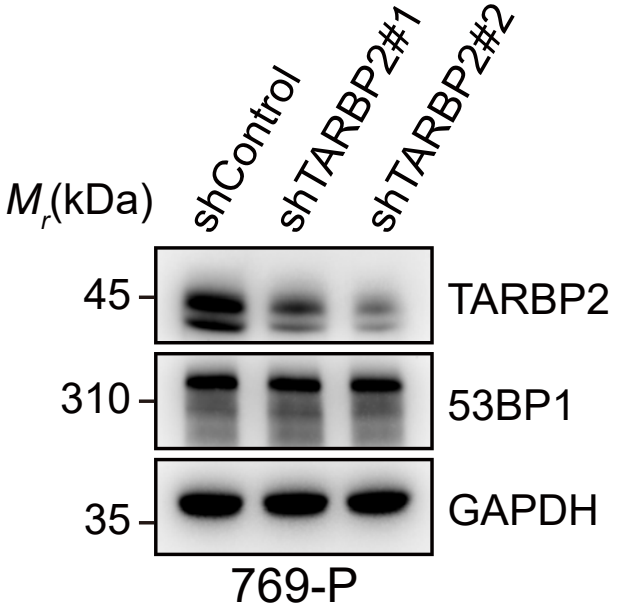

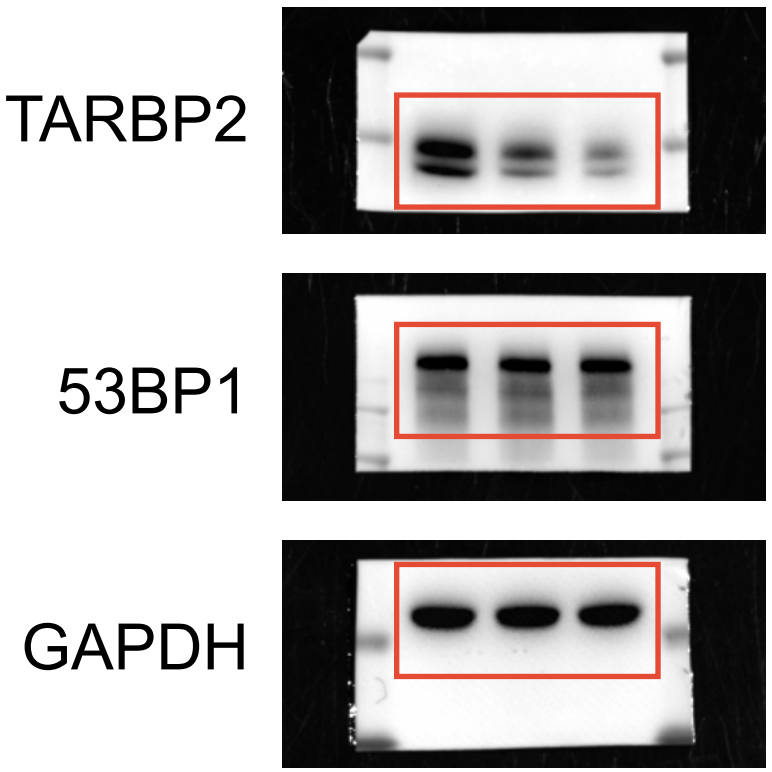

Full unedited blot for Figure 5H

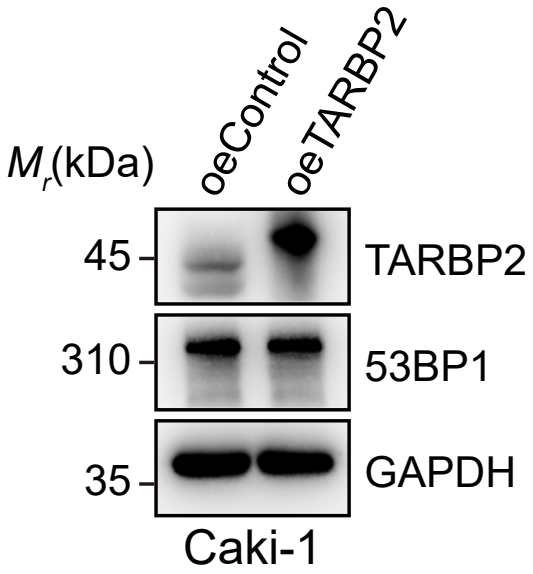

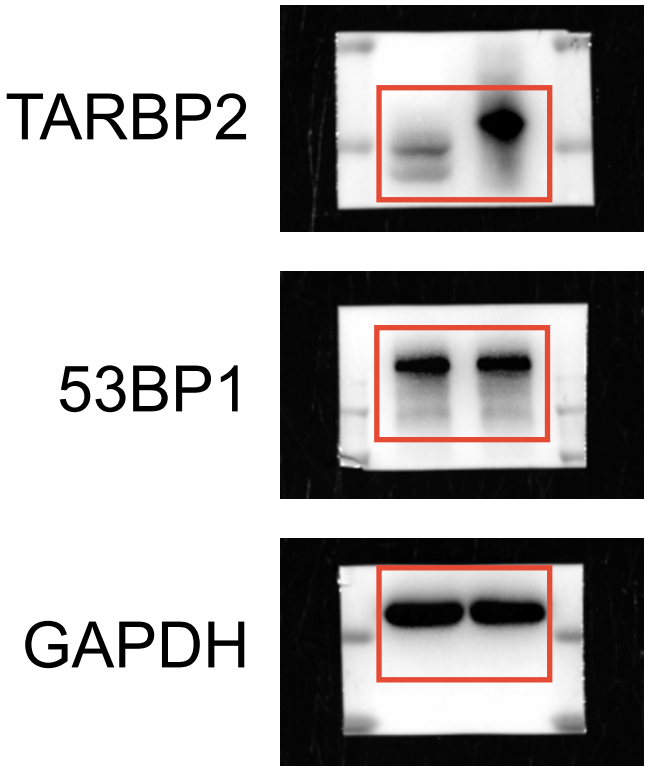

Full unedited blot for Figure 5K

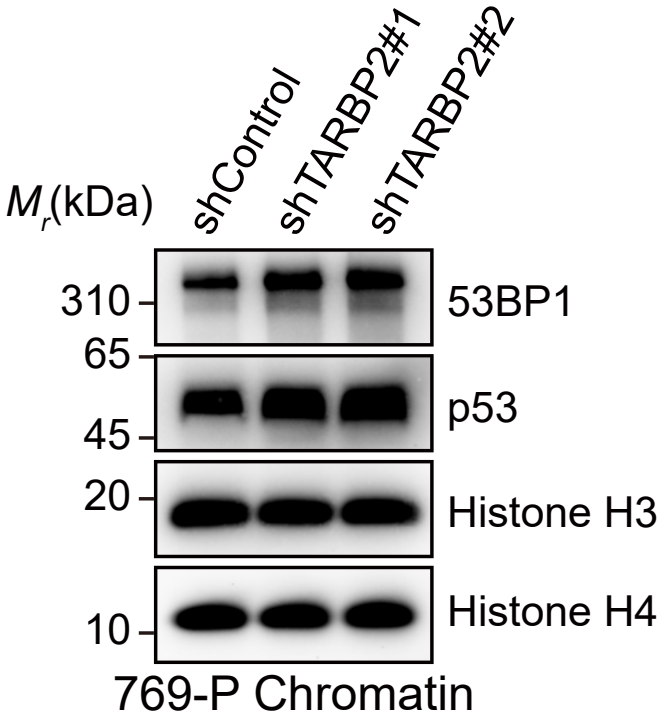

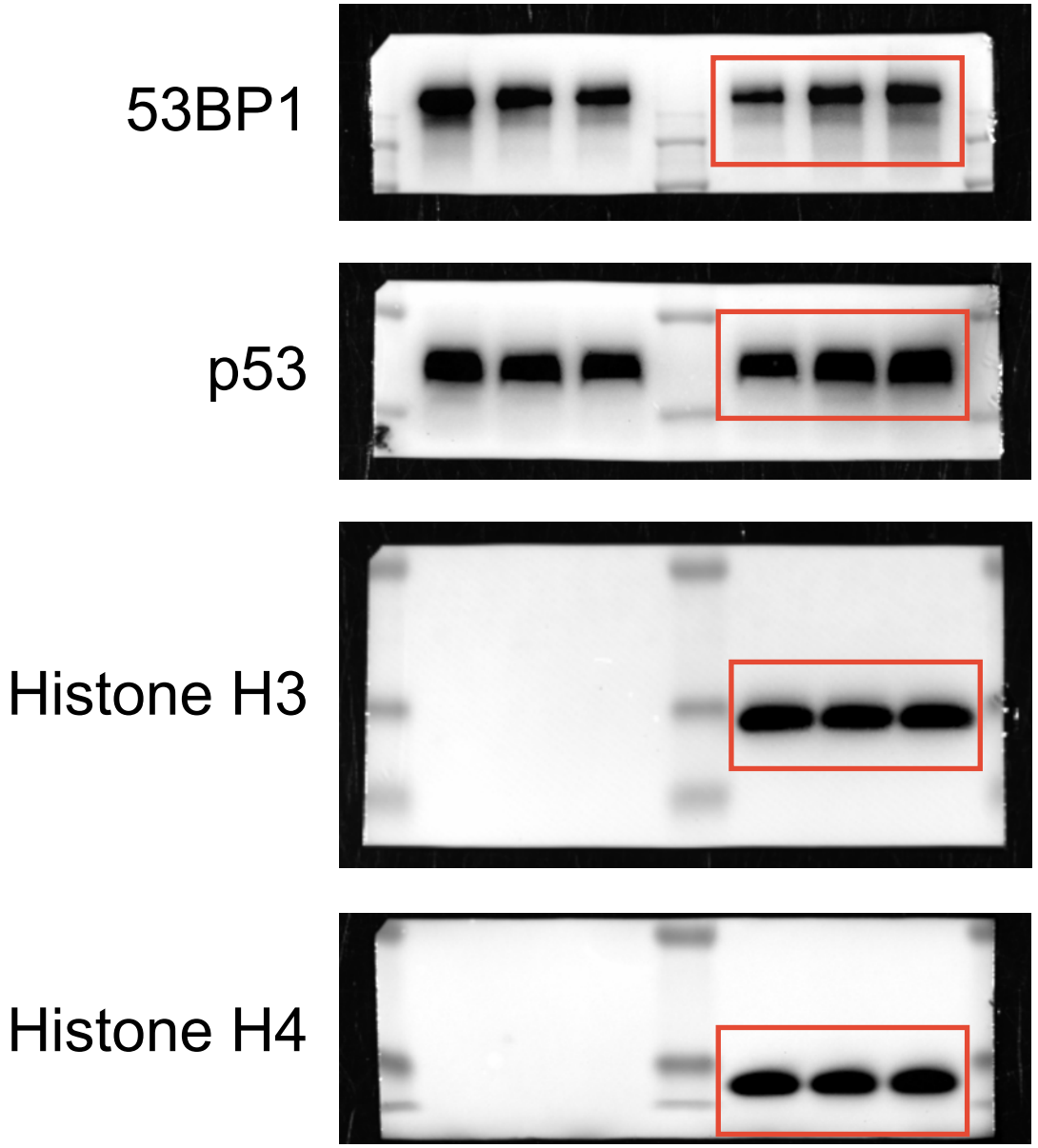

Full unedited blot for Figure 5L

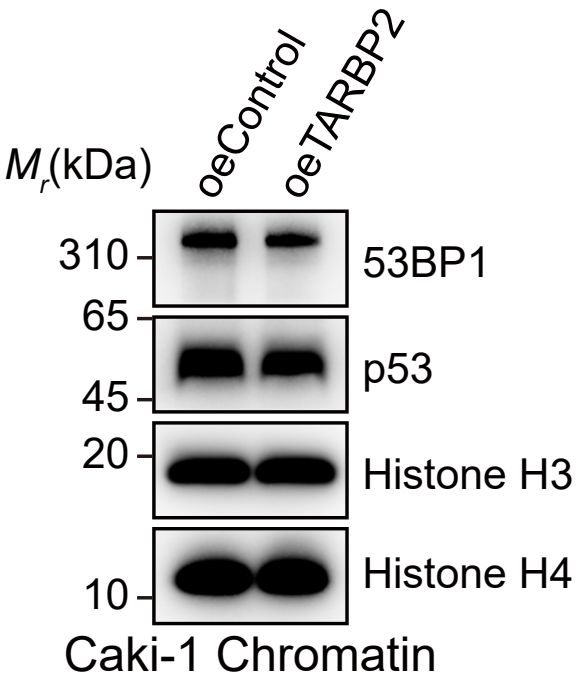

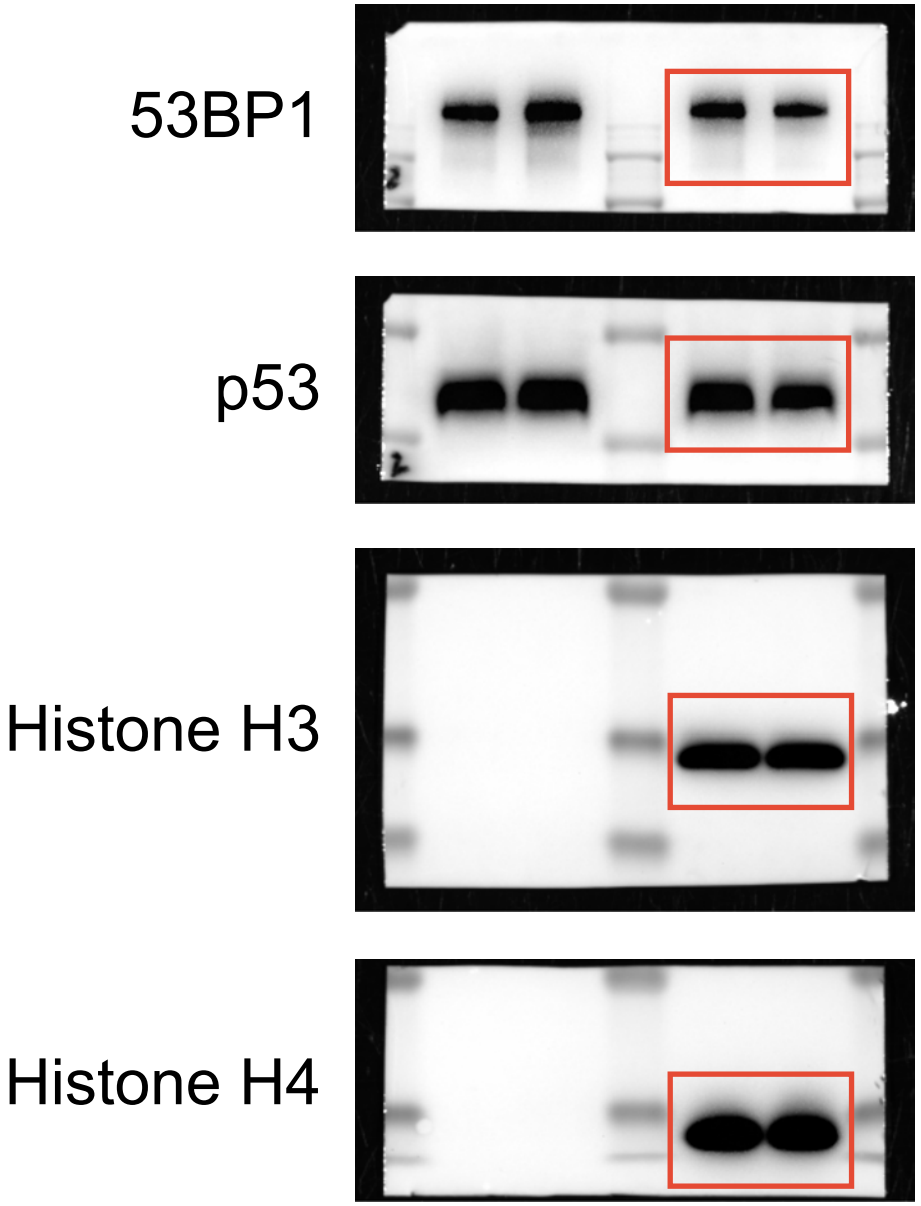

Full unedited blot for Figure 5N

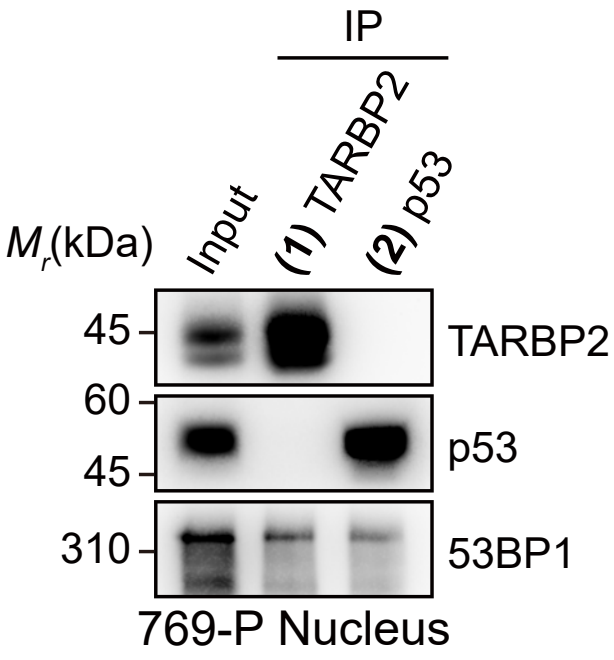

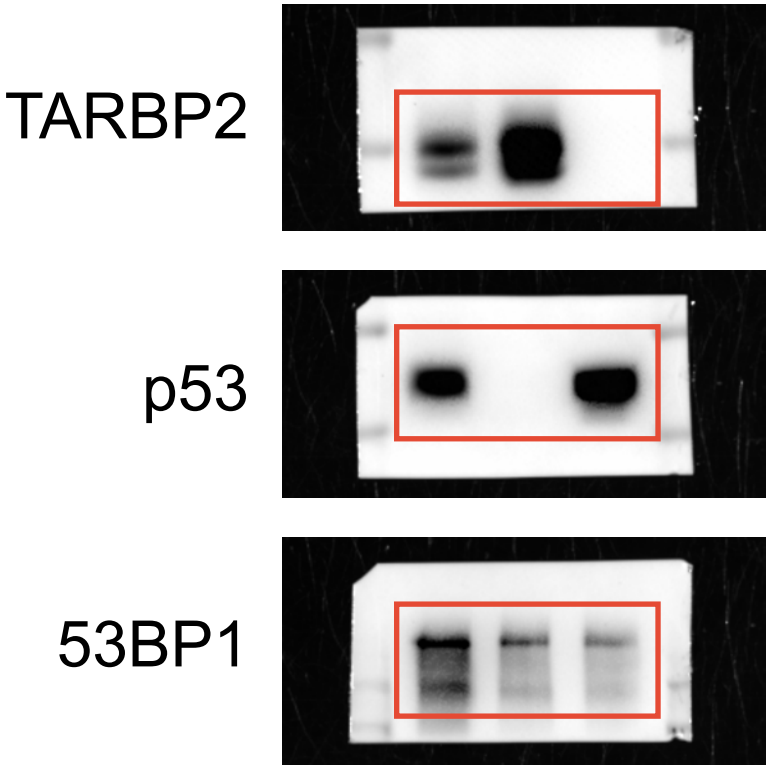

Full unedited blot for Figure 6C

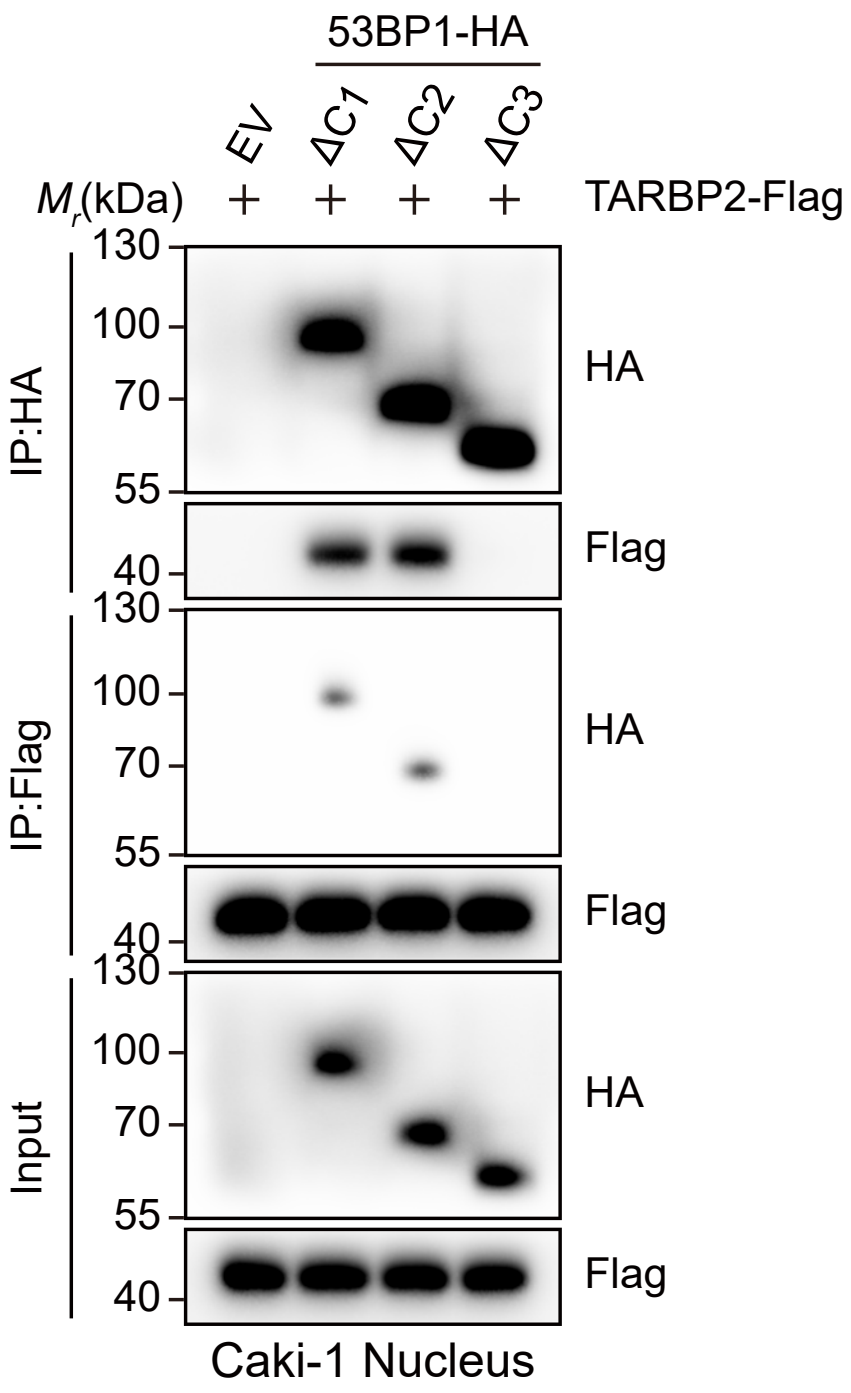

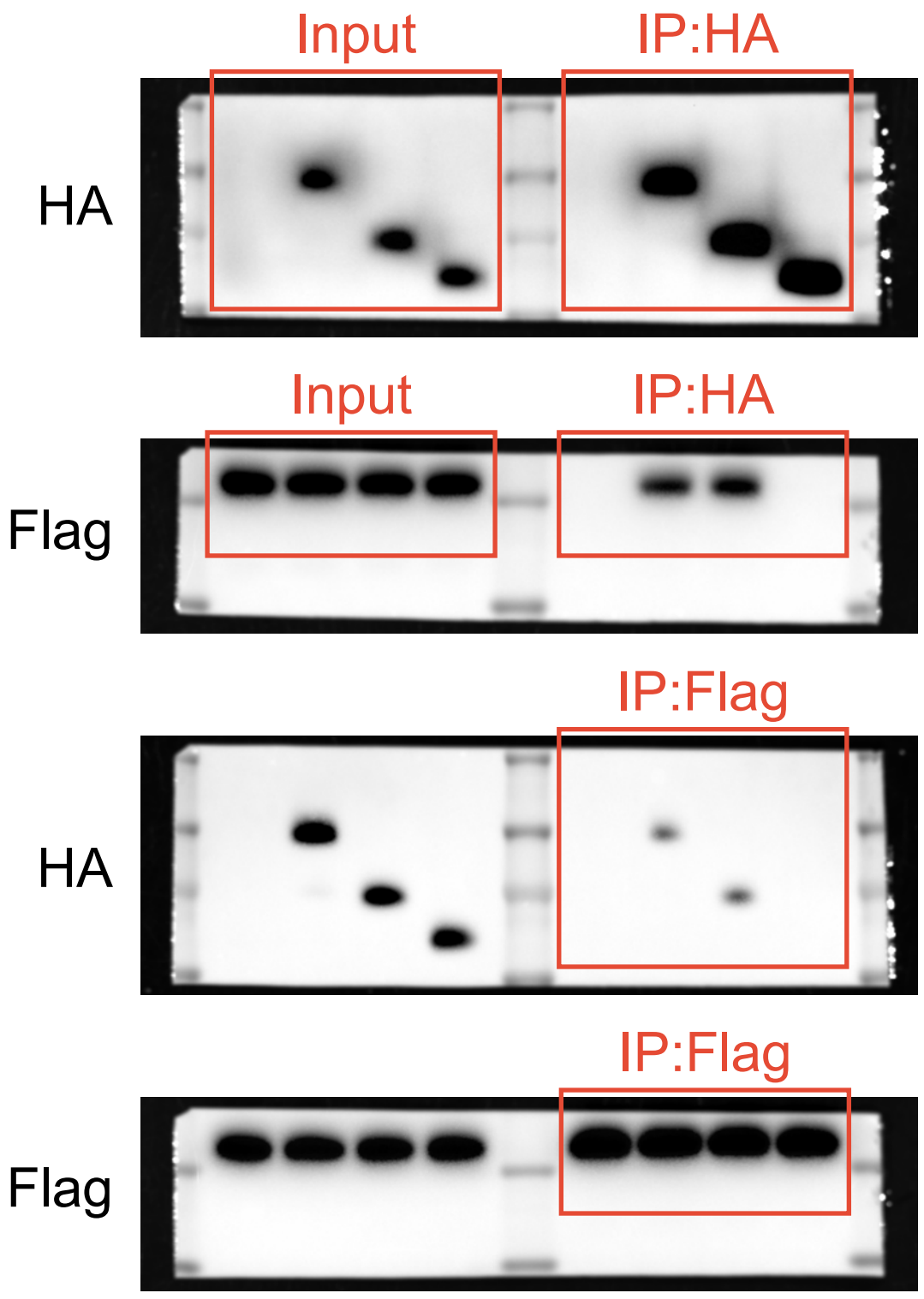

Full unedited blot for Figure 6D

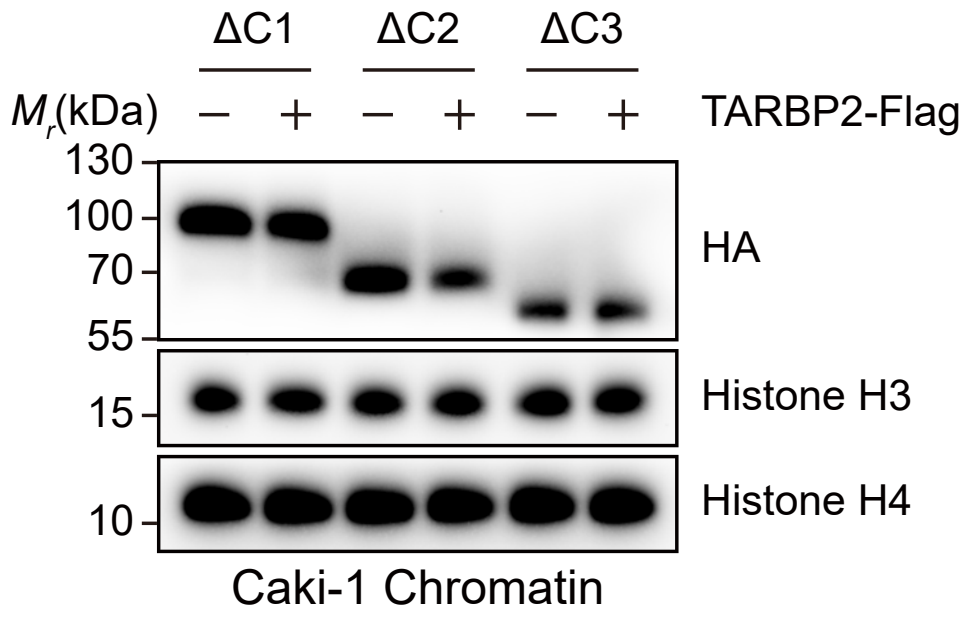

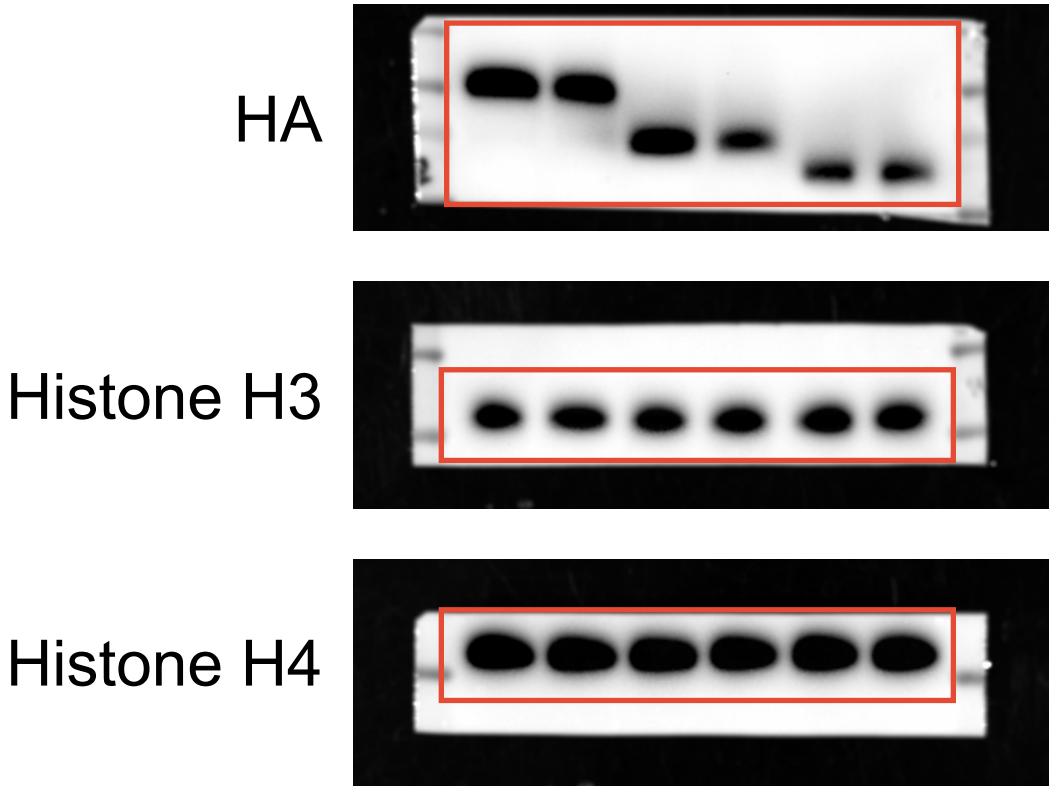

Full unedited blot for Figure 6E

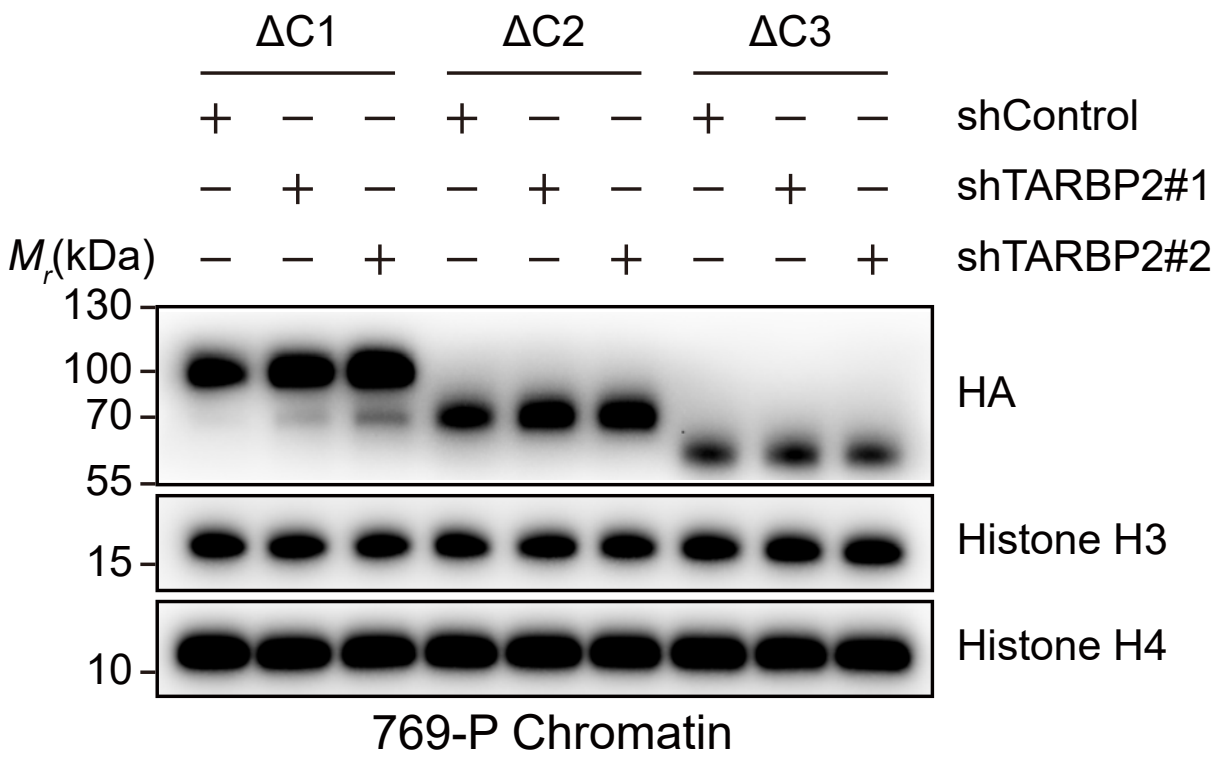

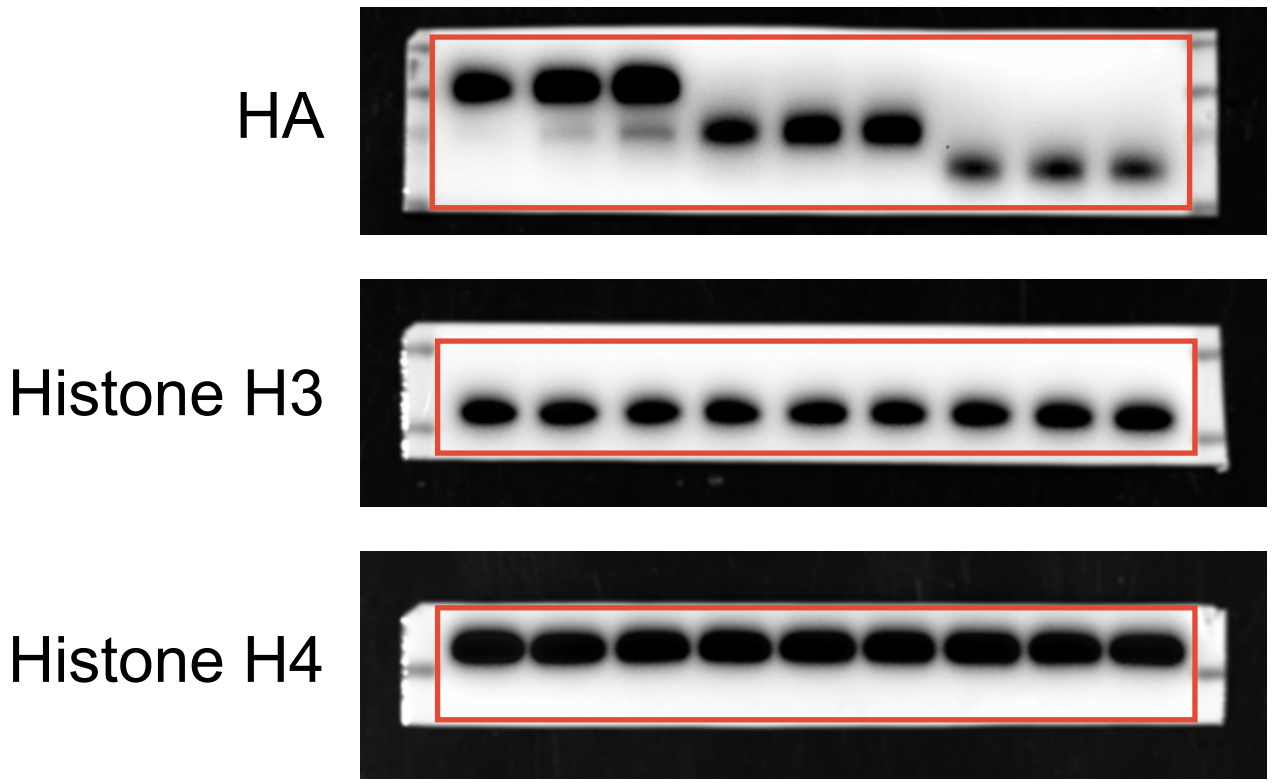

Full unedited blot for Supplemental Figure 3A

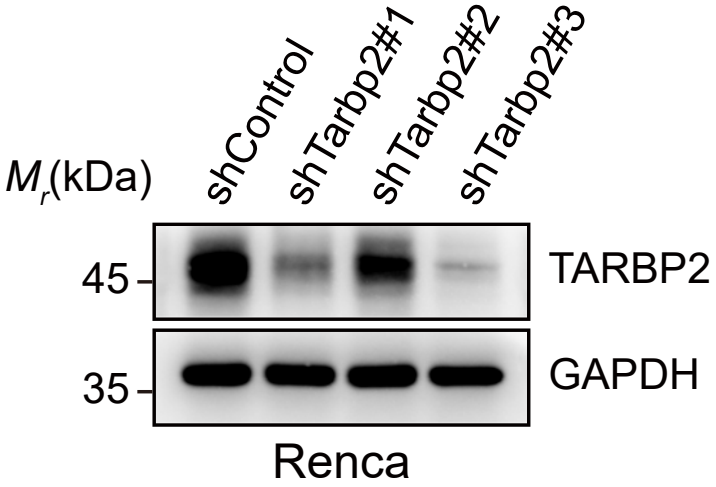

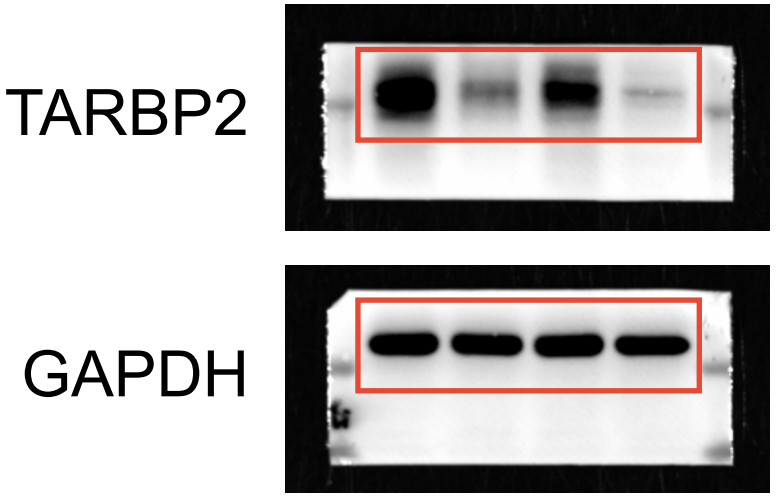

Full unedited blot for Supplemental Figure 8G

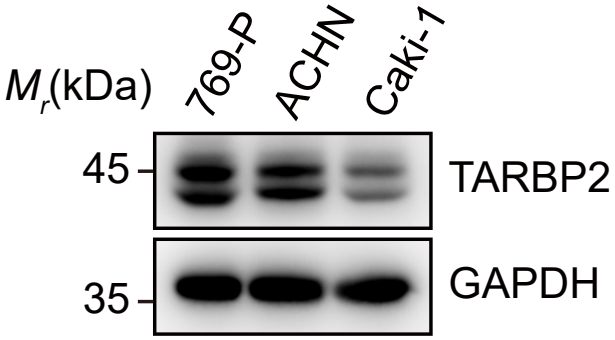

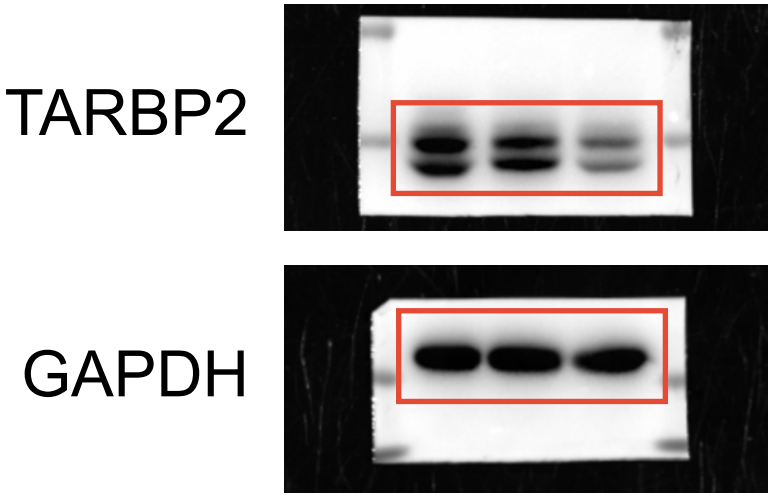

Full unedited blot for Supplemental Figure 8J

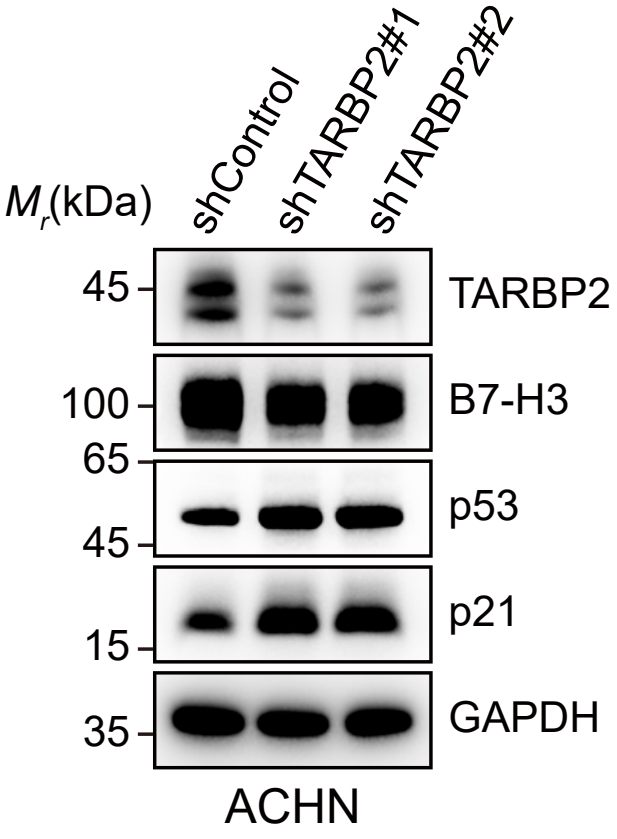

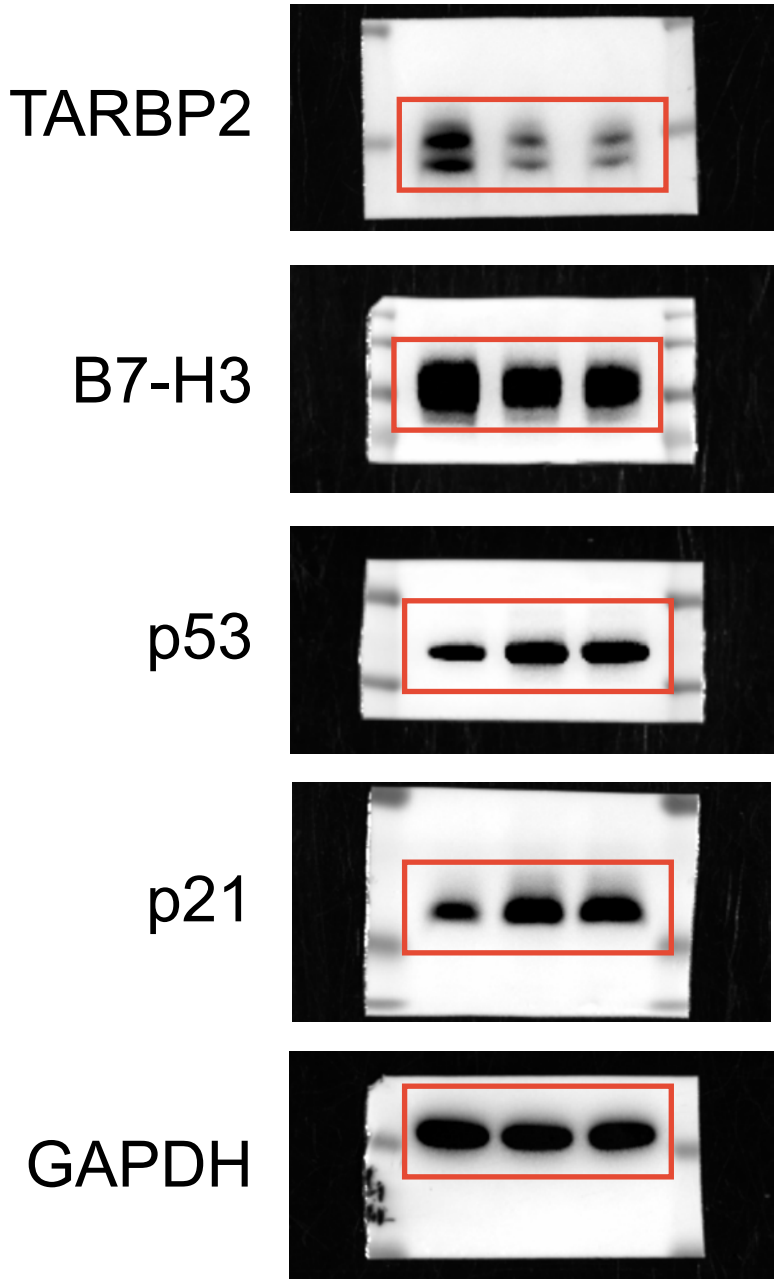

Full unedited blot for Supplemental Figure 8L

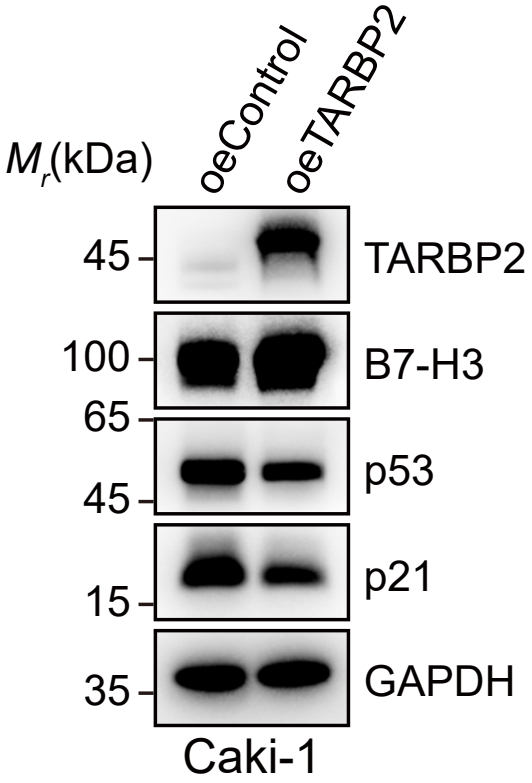

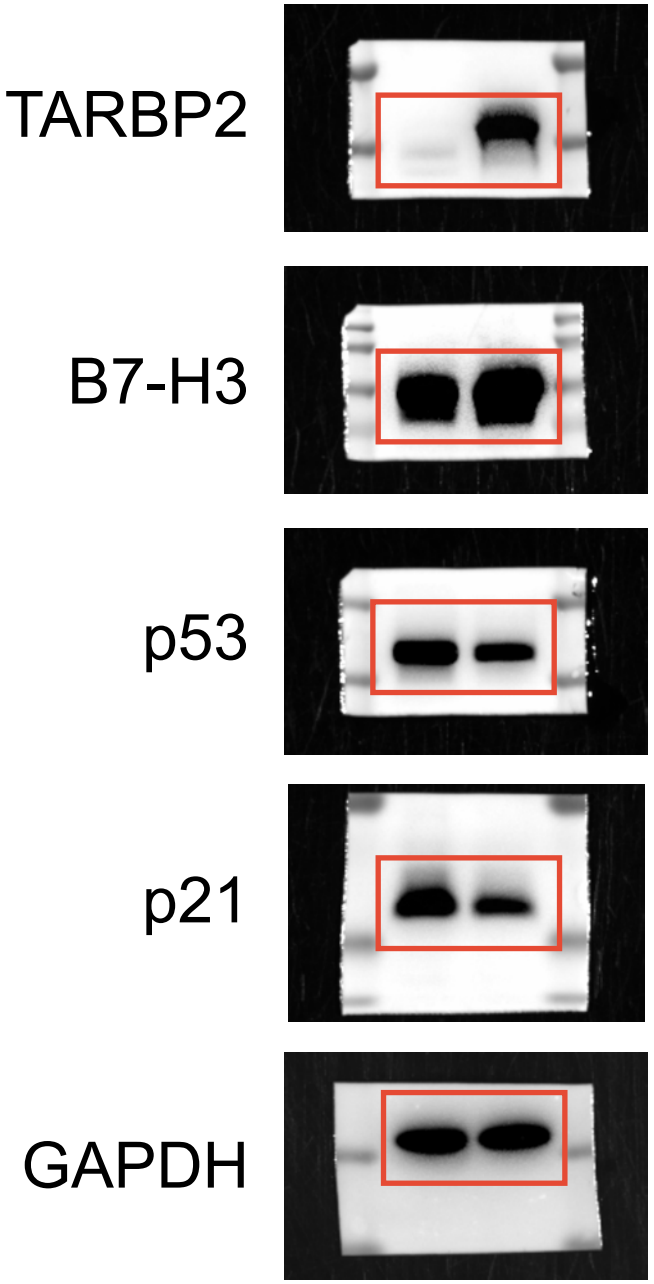

Full unedited blot for Supplemental Figure 9B

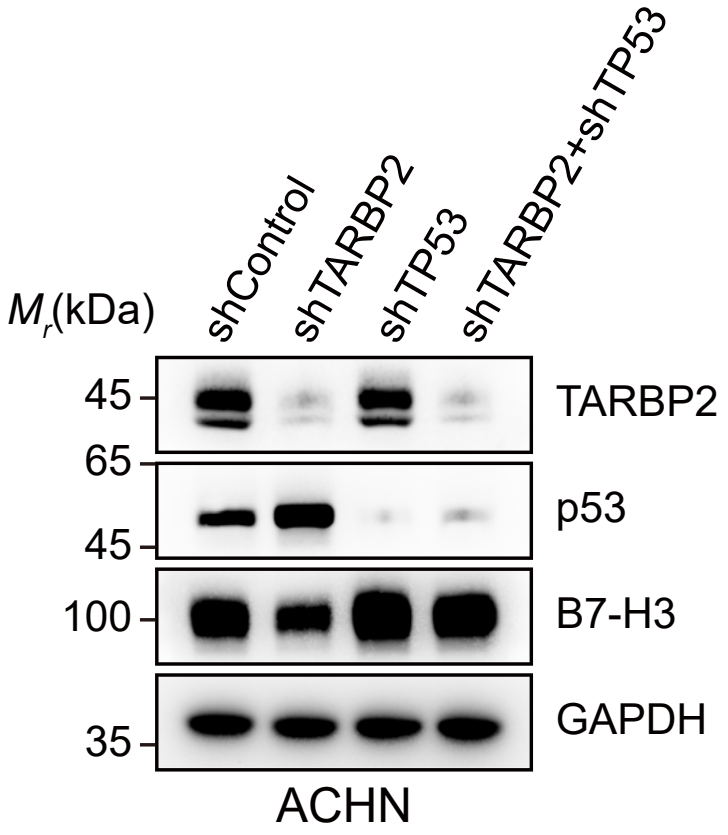

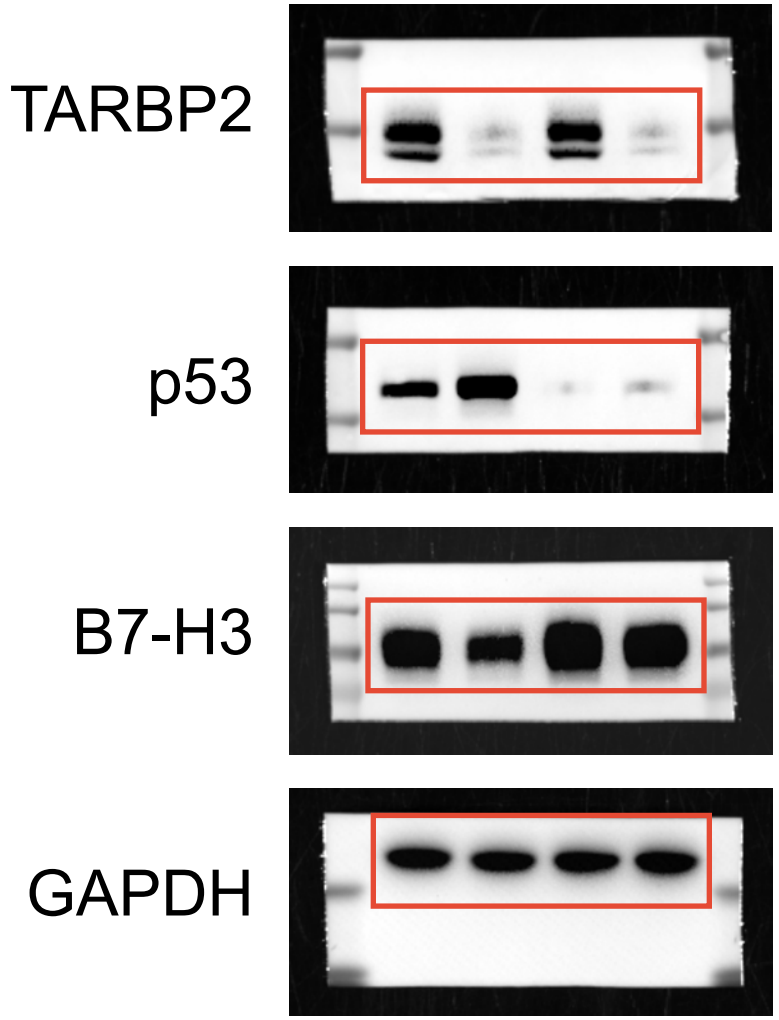

Full unedited blot for Supplemental Figure 9D

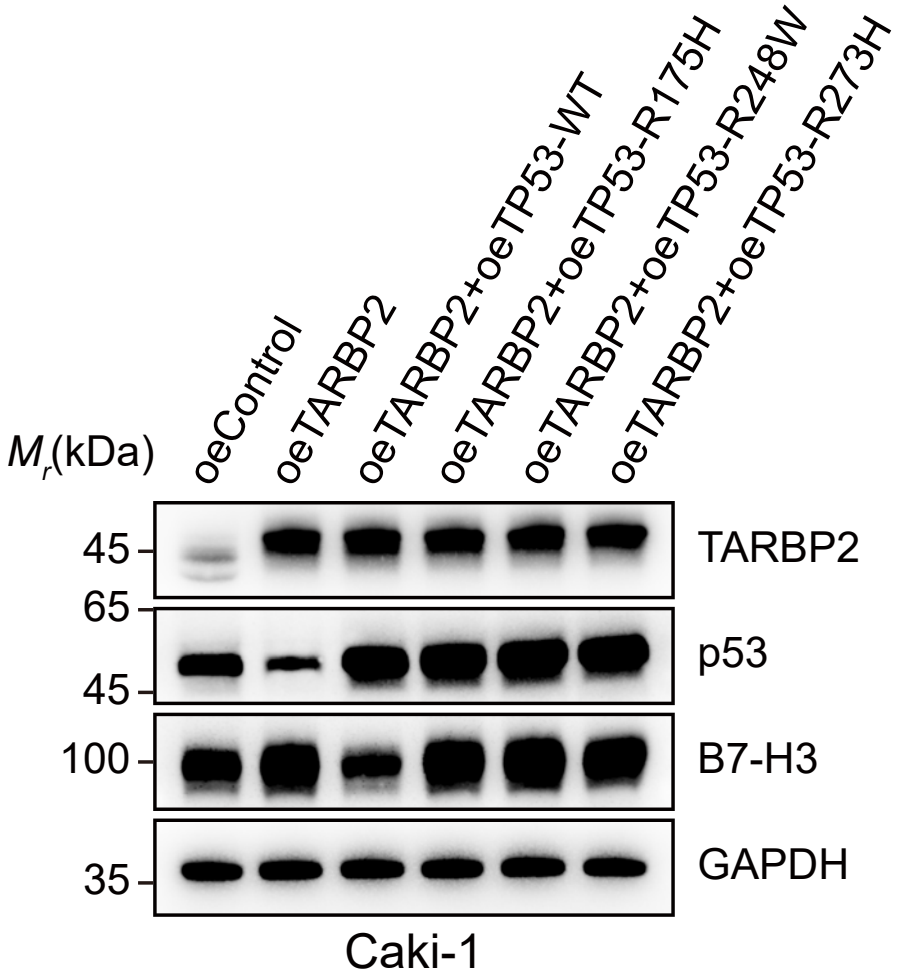

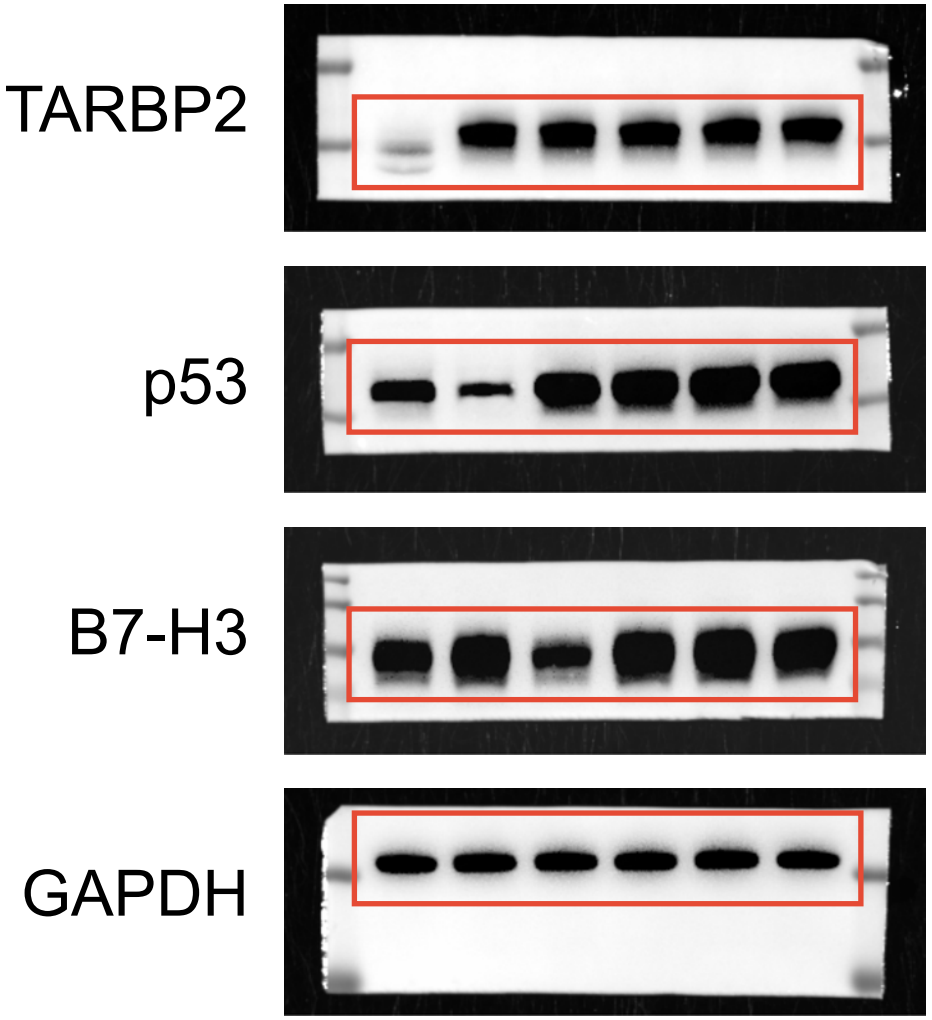

Full unedited blot for Supplemental Figure 11E

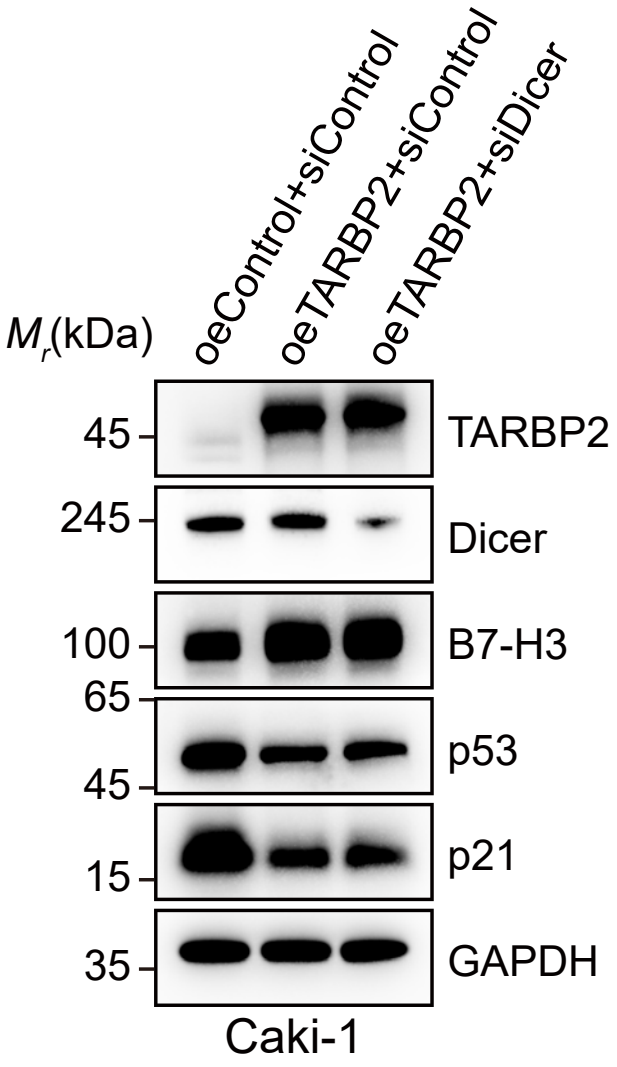

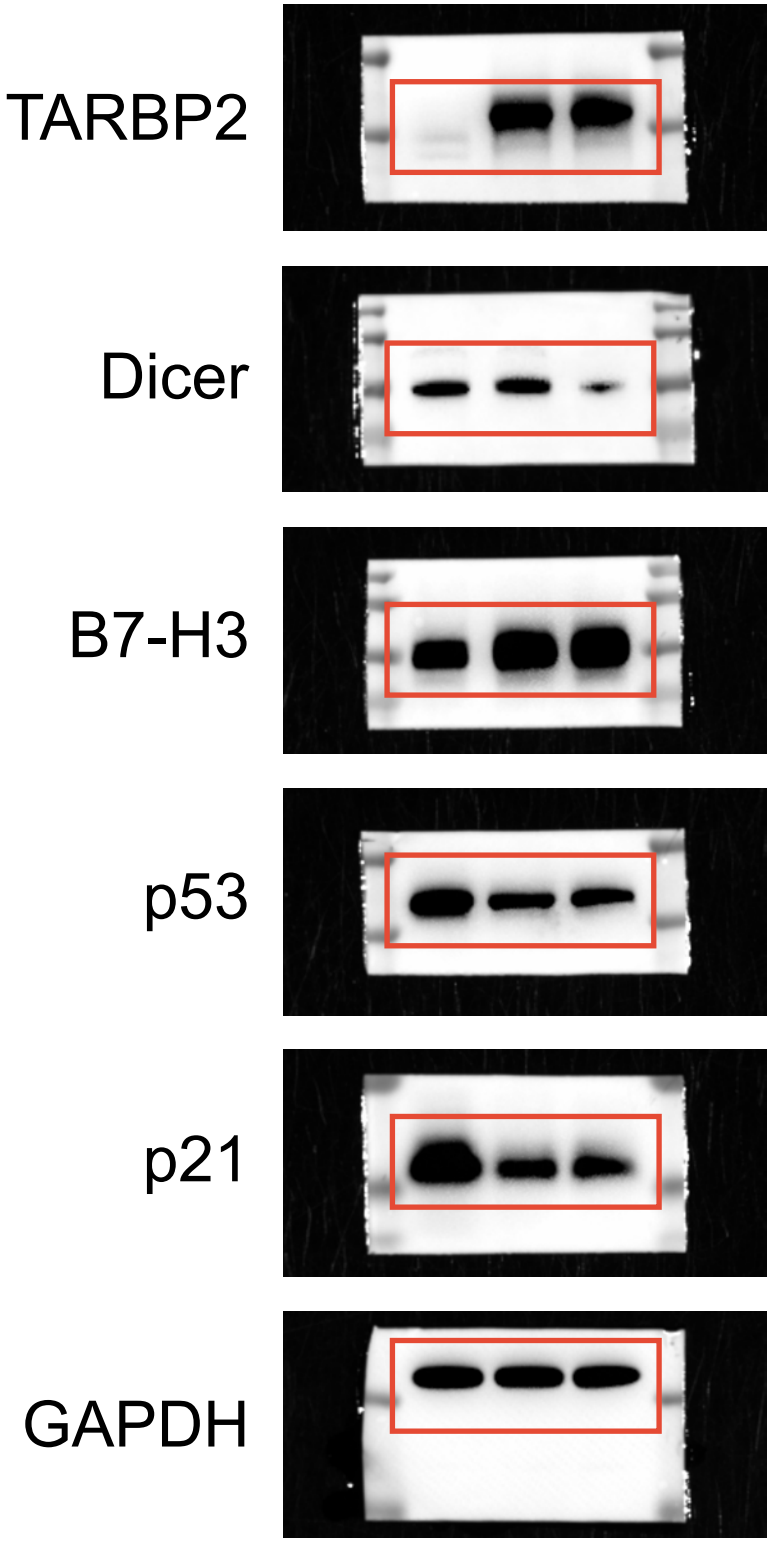

Full unedited blot for Supplemental Figure 12D

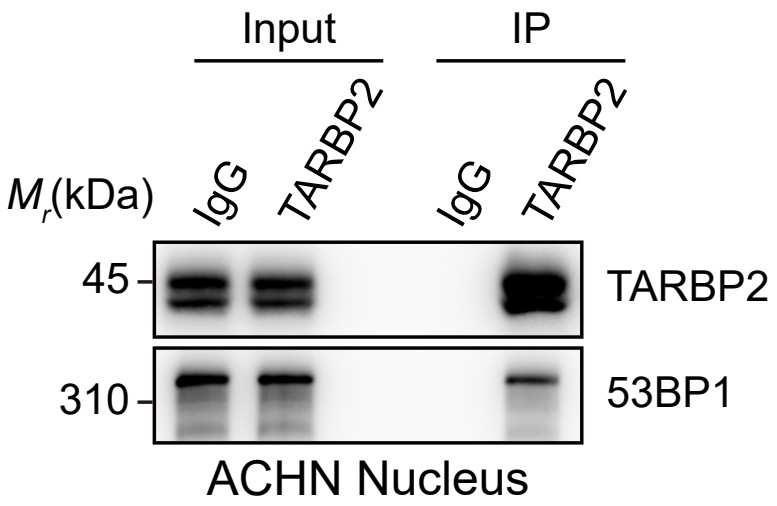

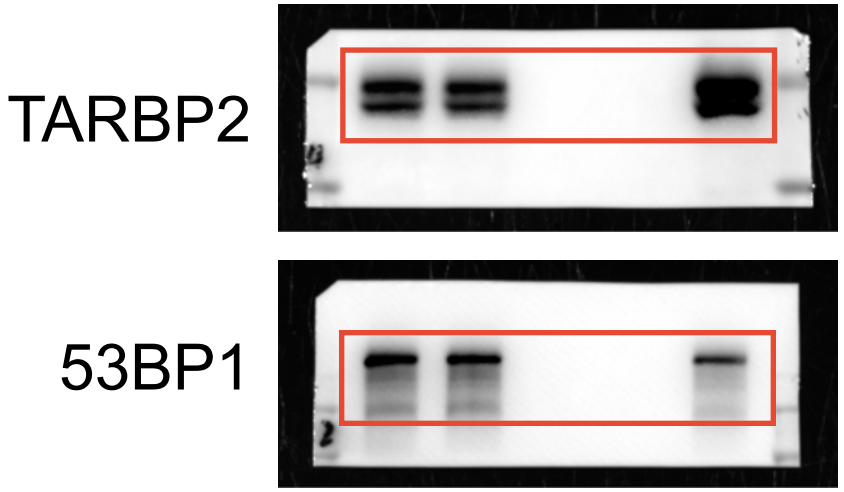

Full unedited blot for Supplemental Figure 12E

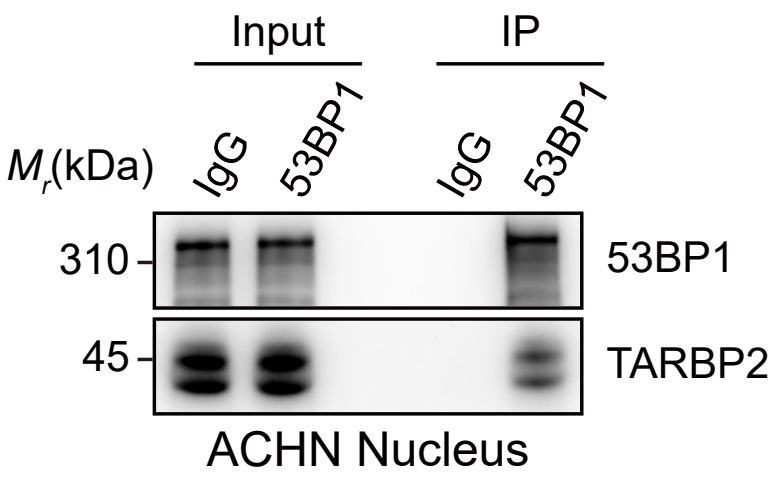

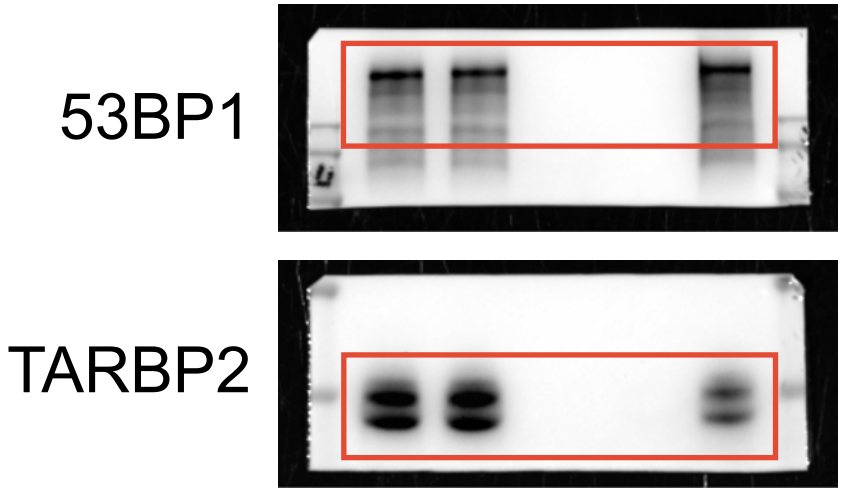

Full unedited blot for Supplemental Figure 12F

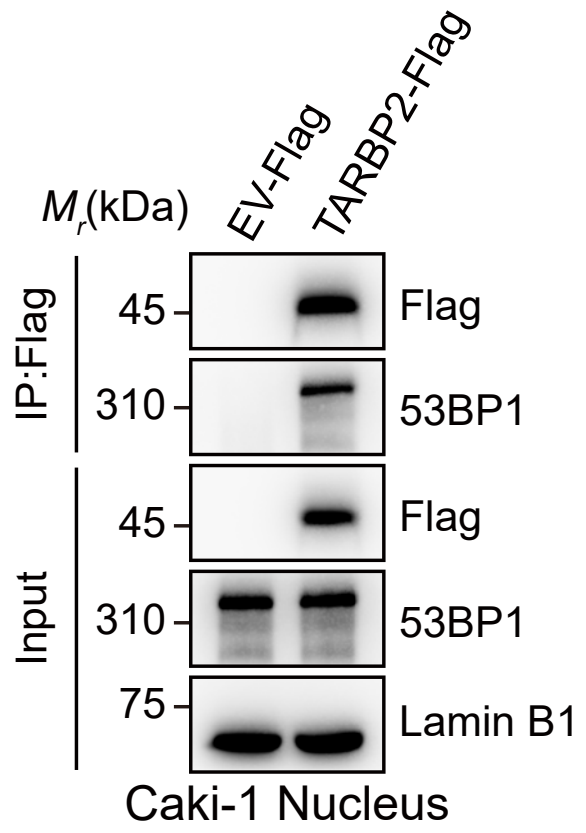

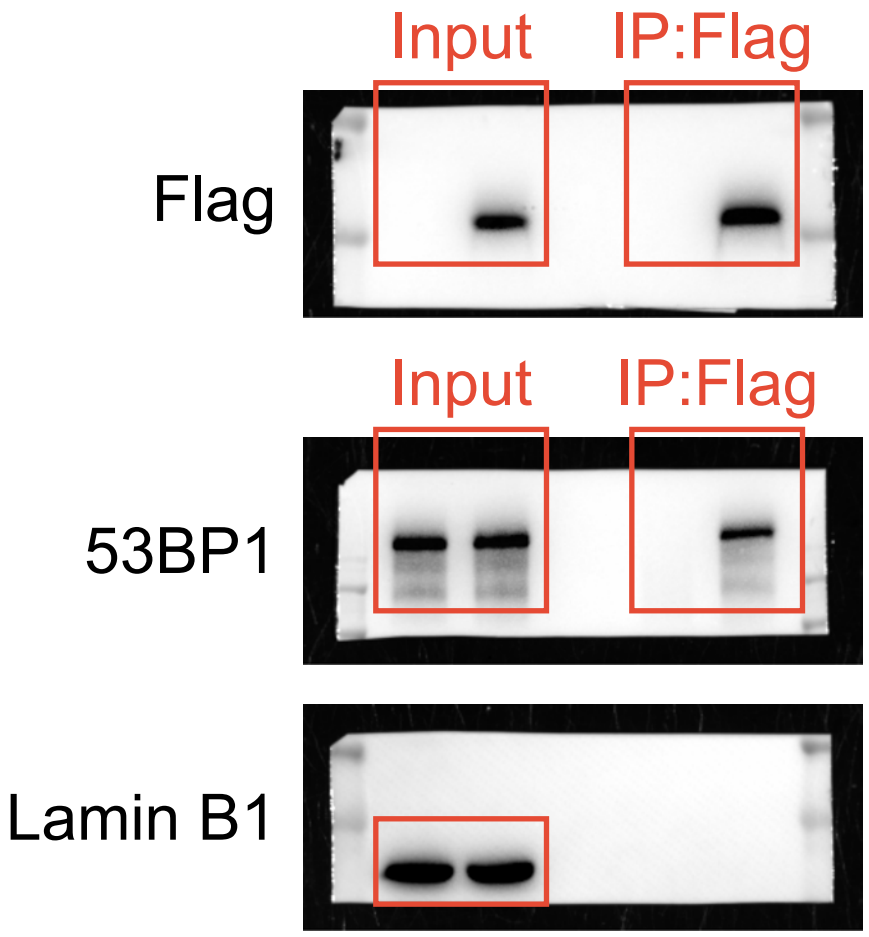

Full unedited blot for Supplemental Figure 12G

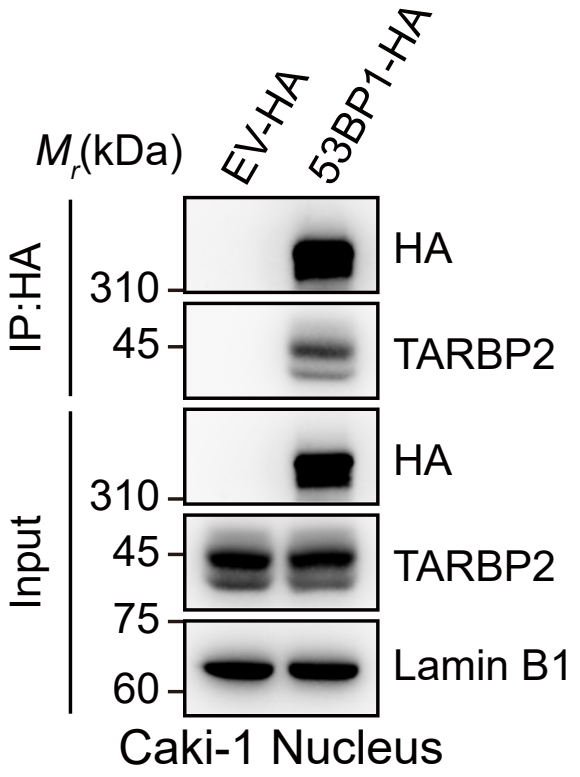

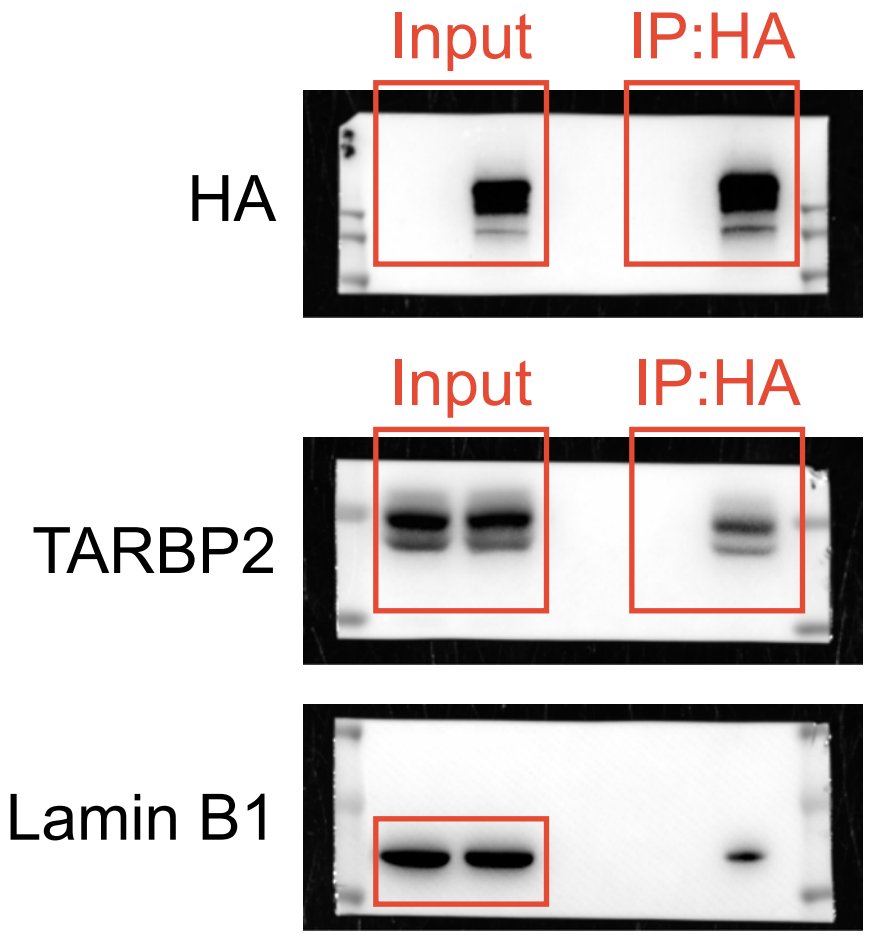

Full unedited blot for Supplemental Figure 12I

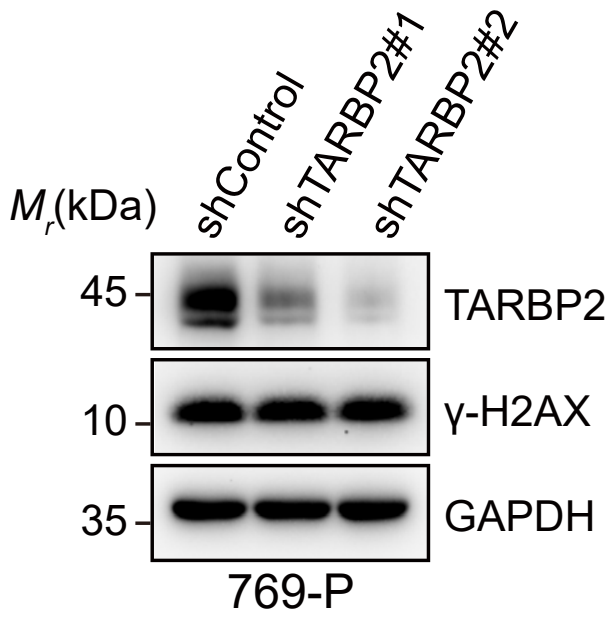

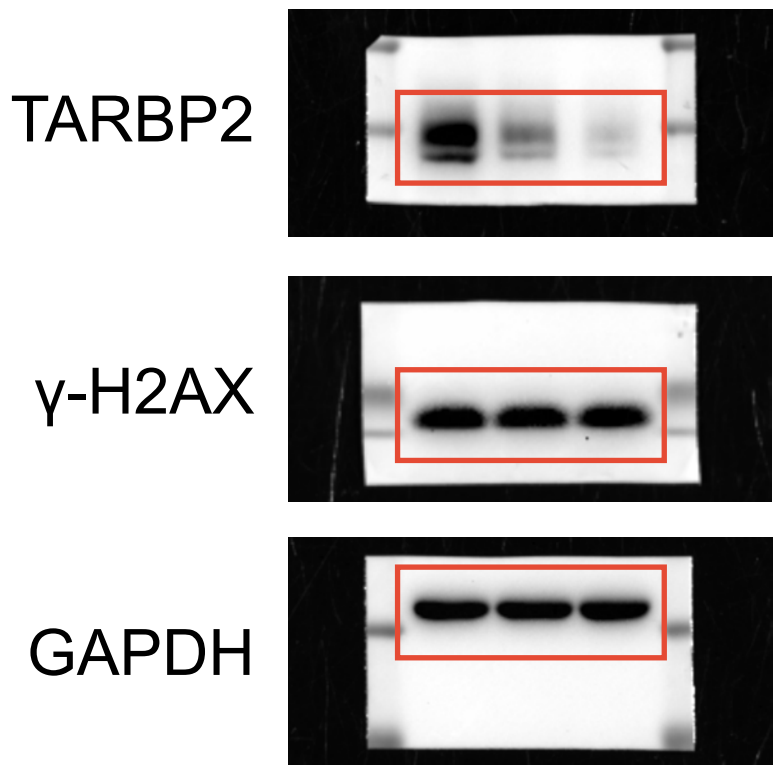

Full unedited blot for Supplemental Figure 12J

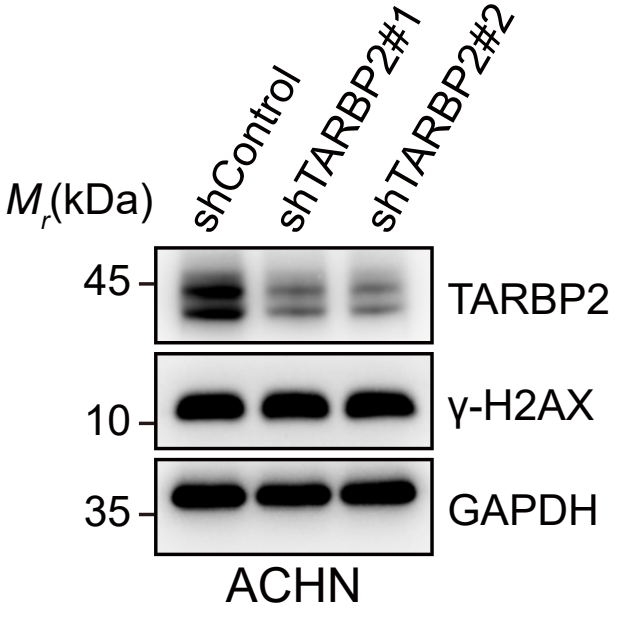

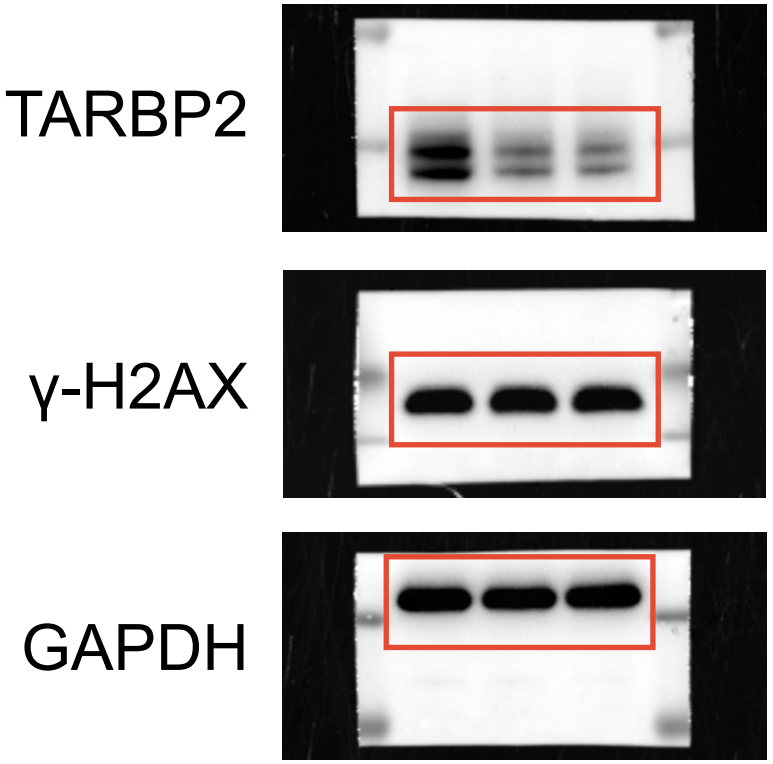

Full unedited blot for Supplemental Figure 12K

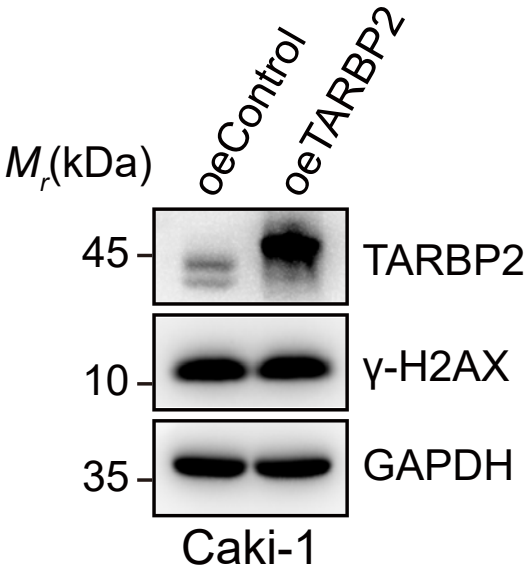

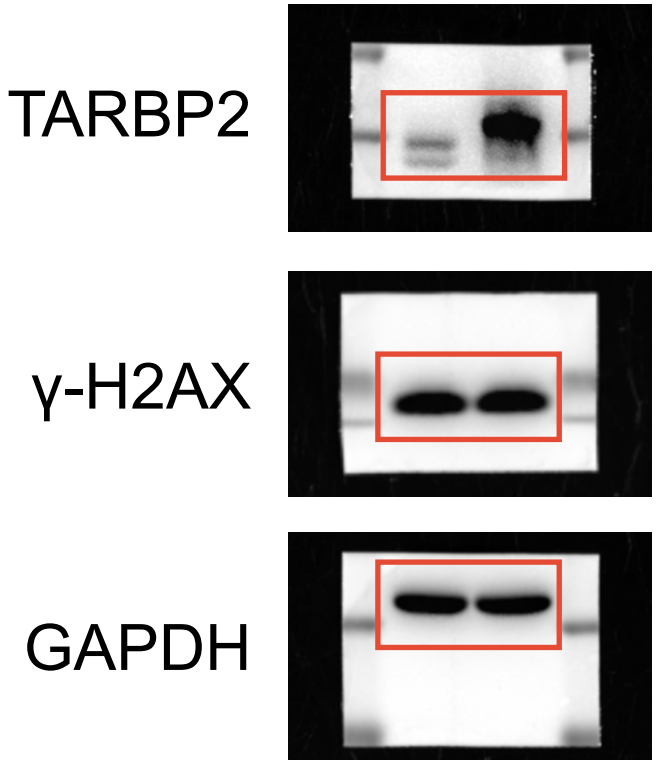

Full unedited blot for Supplemental Figure 12L

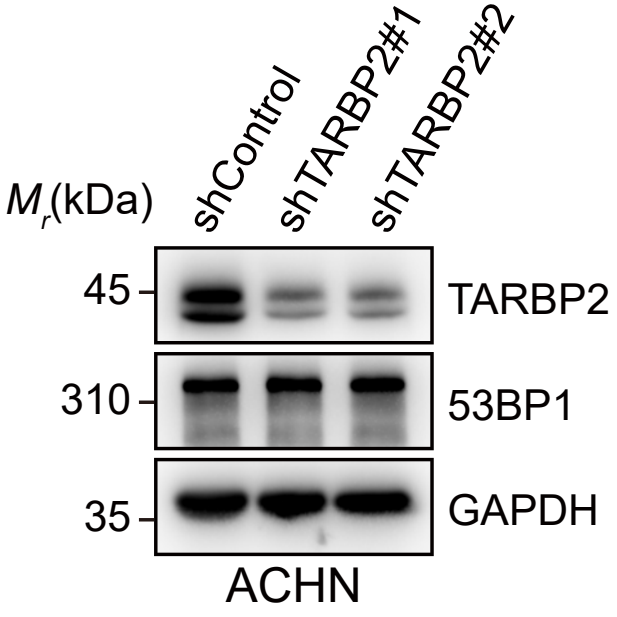

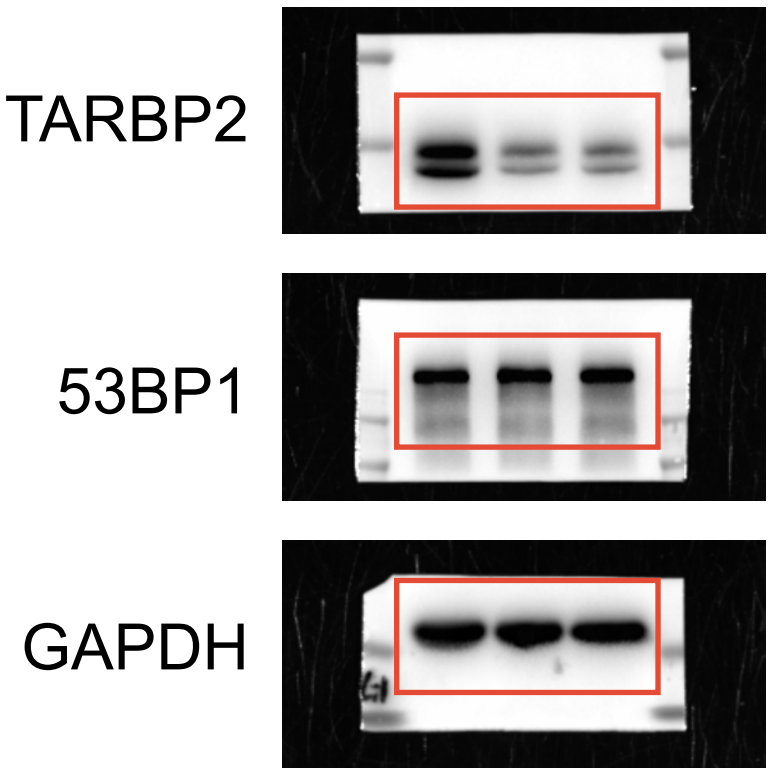

Full unedited blot for Supplemental Figure 12N

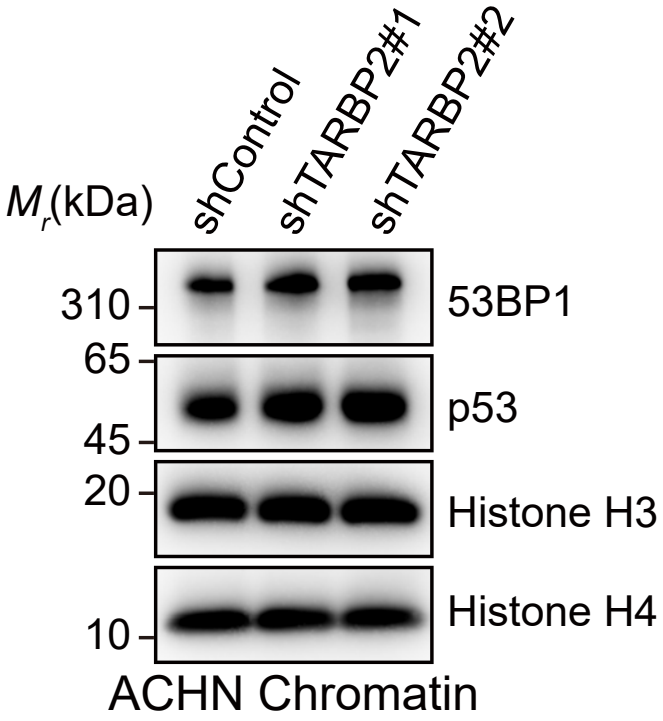

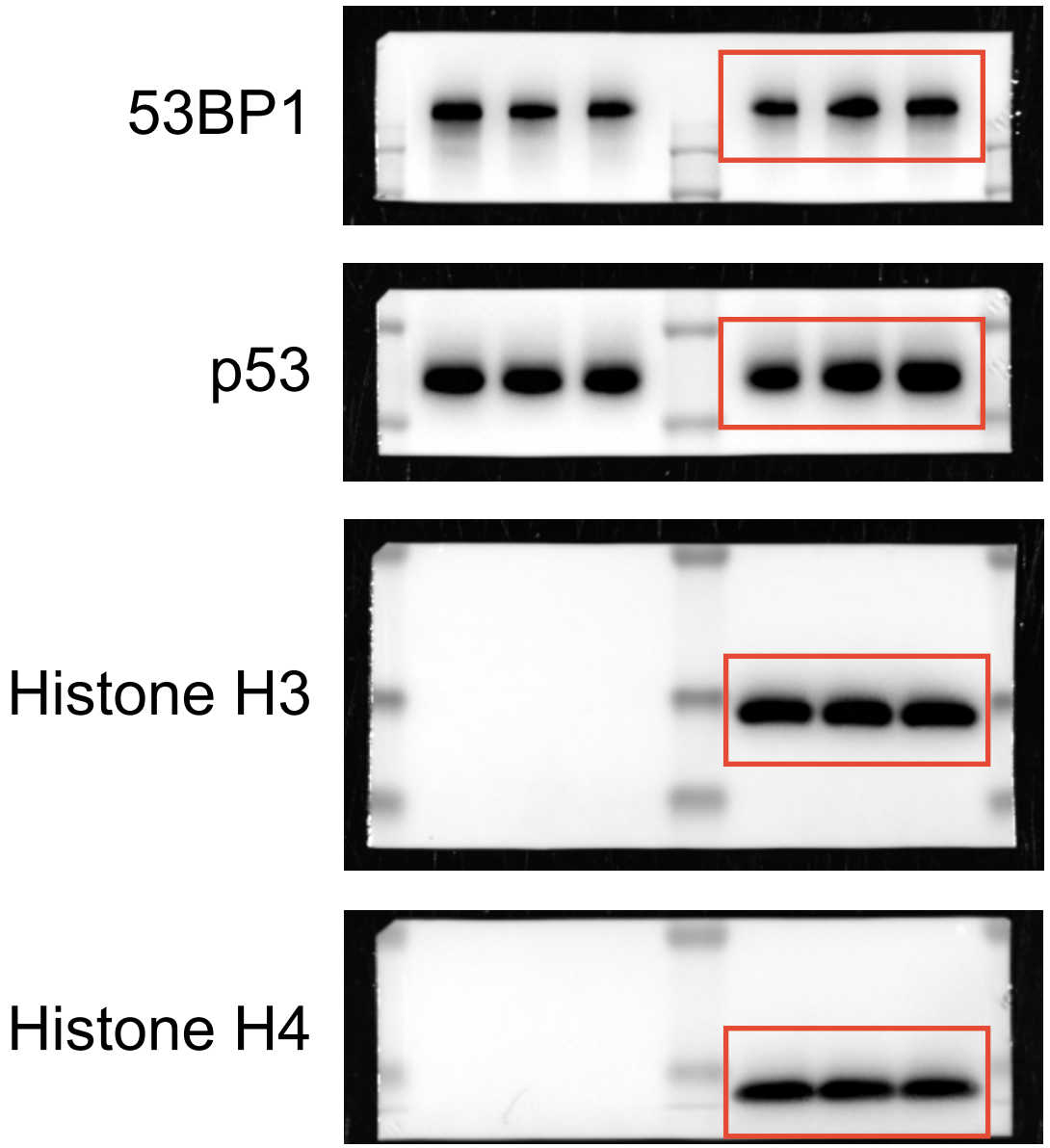

Full unedited blot for Supplemental Figure 13A

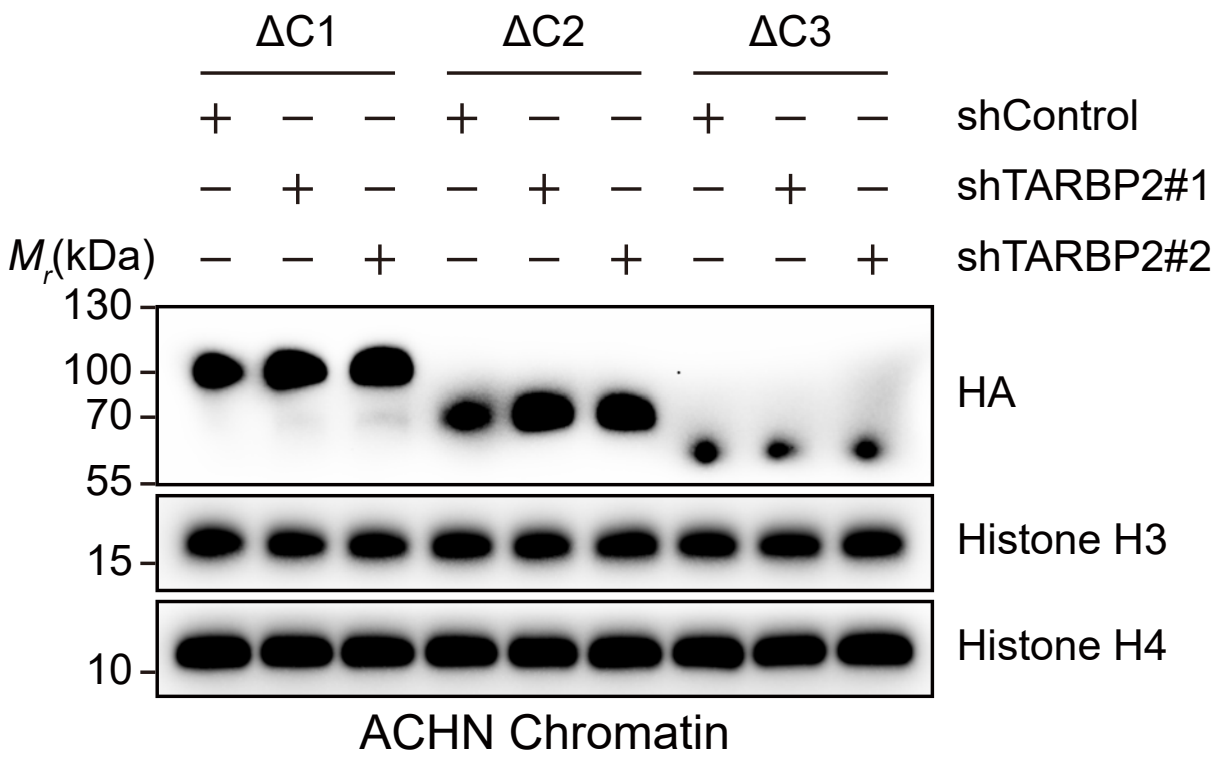

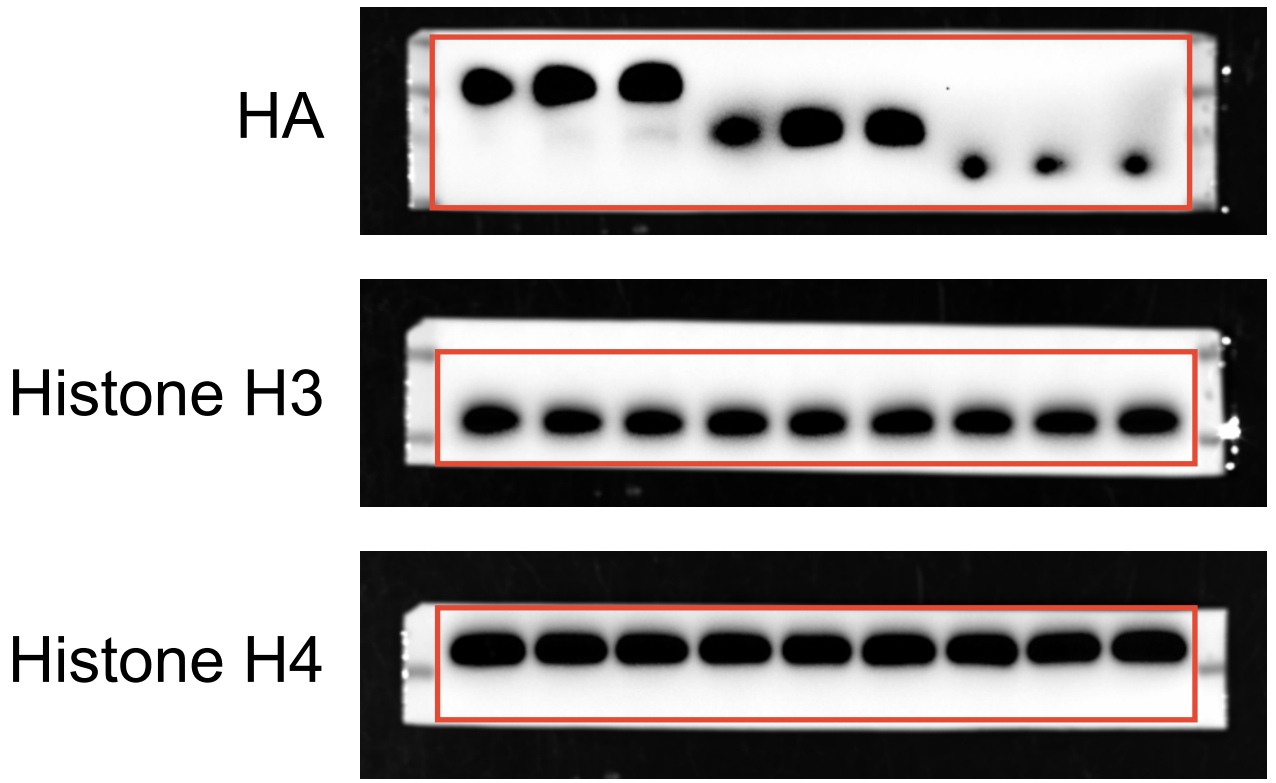

Full unedited blot for Supplemental Figure 14B

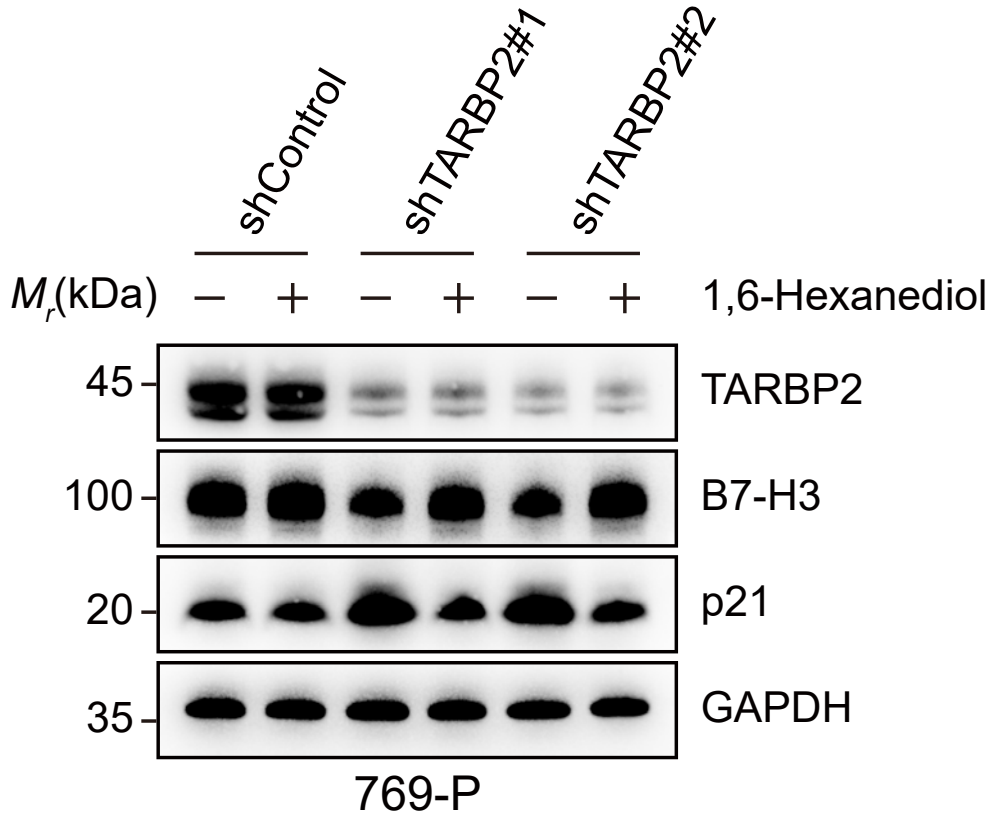

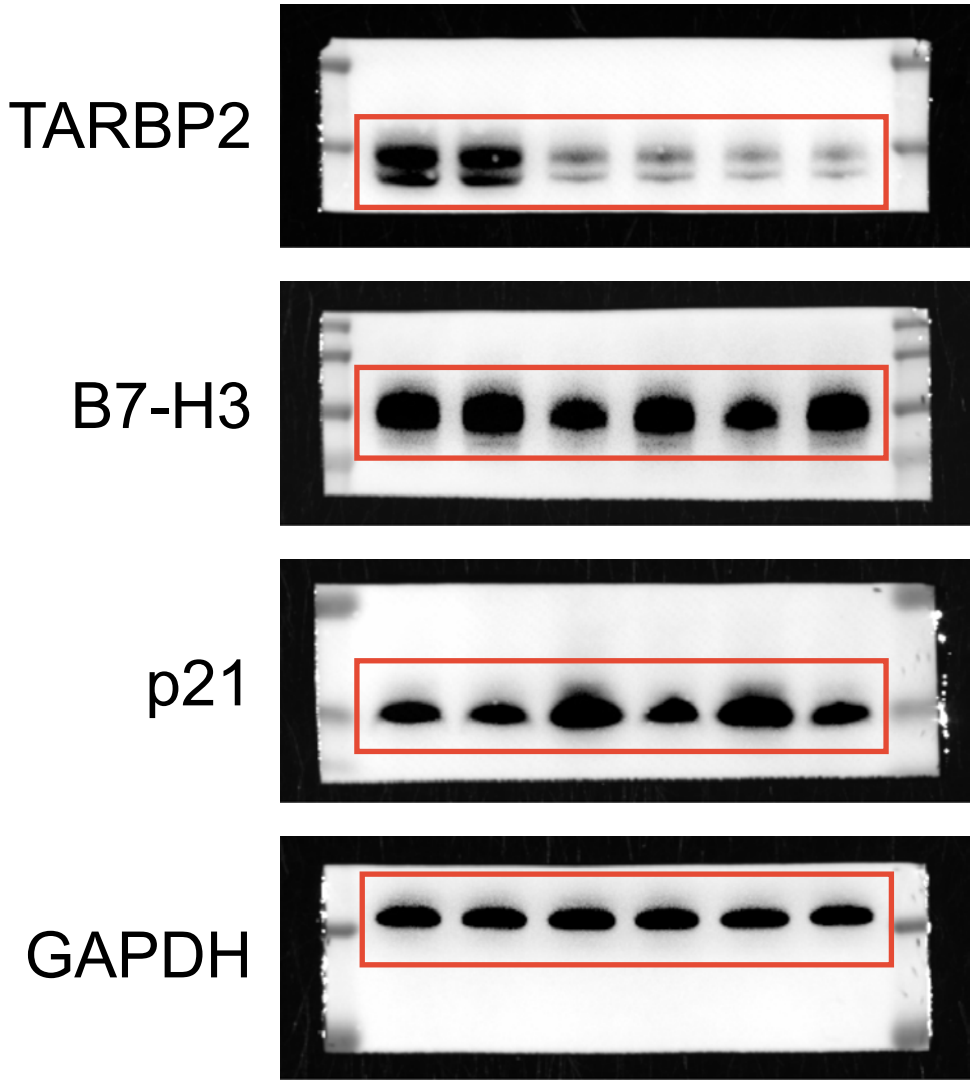

Full unedited blot for Supplemental Figure 14D

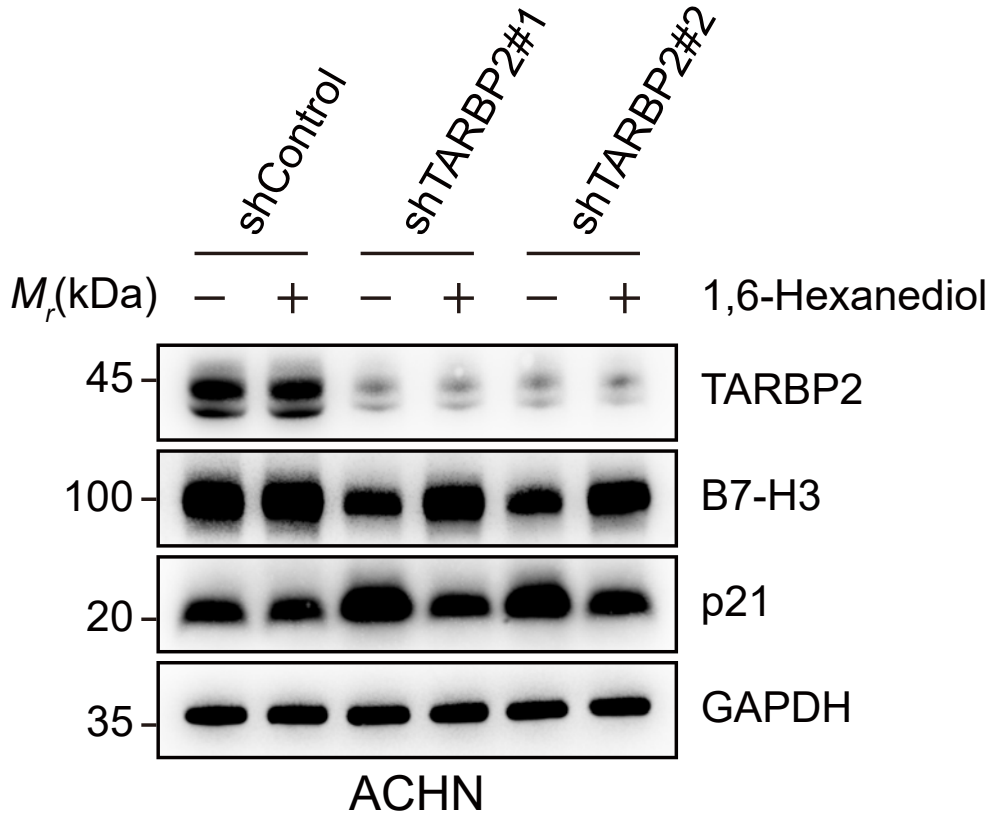

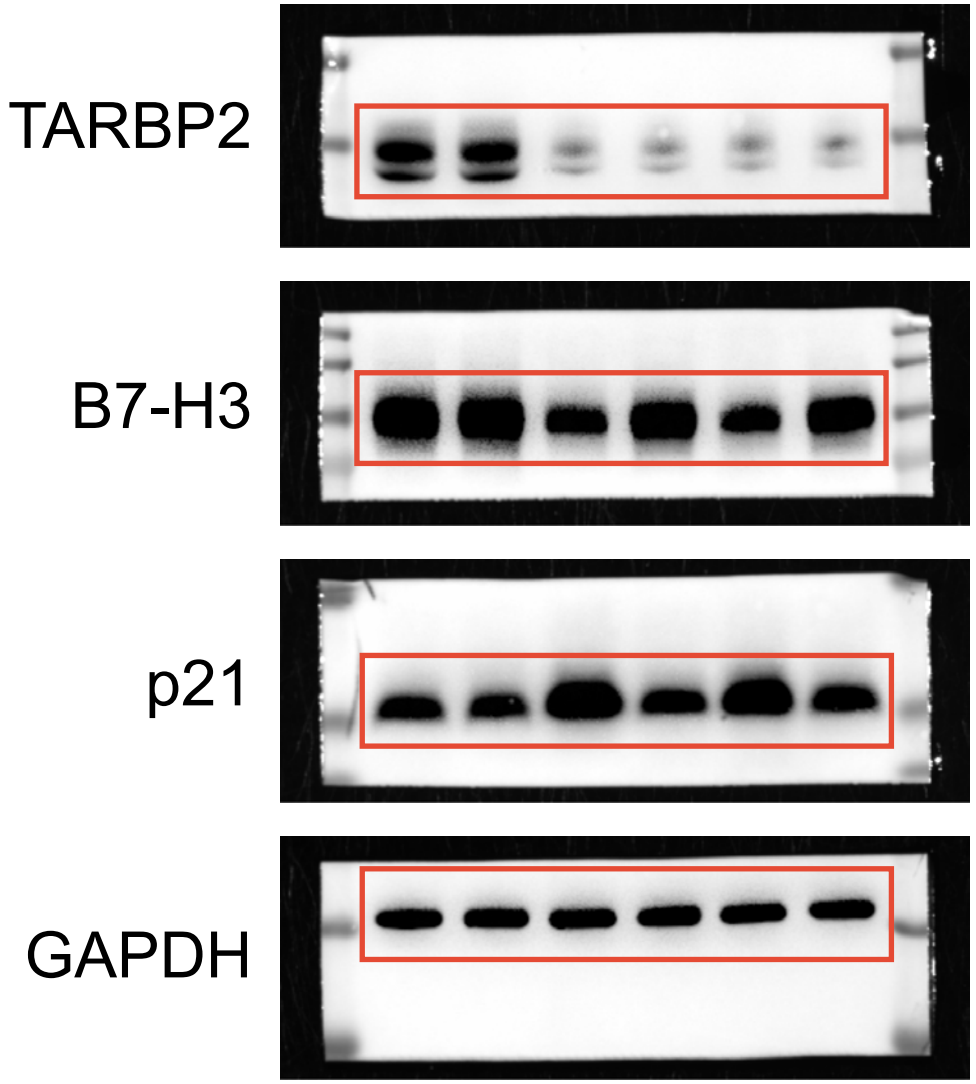

Full unedited blot for Supplemental Figure 14F

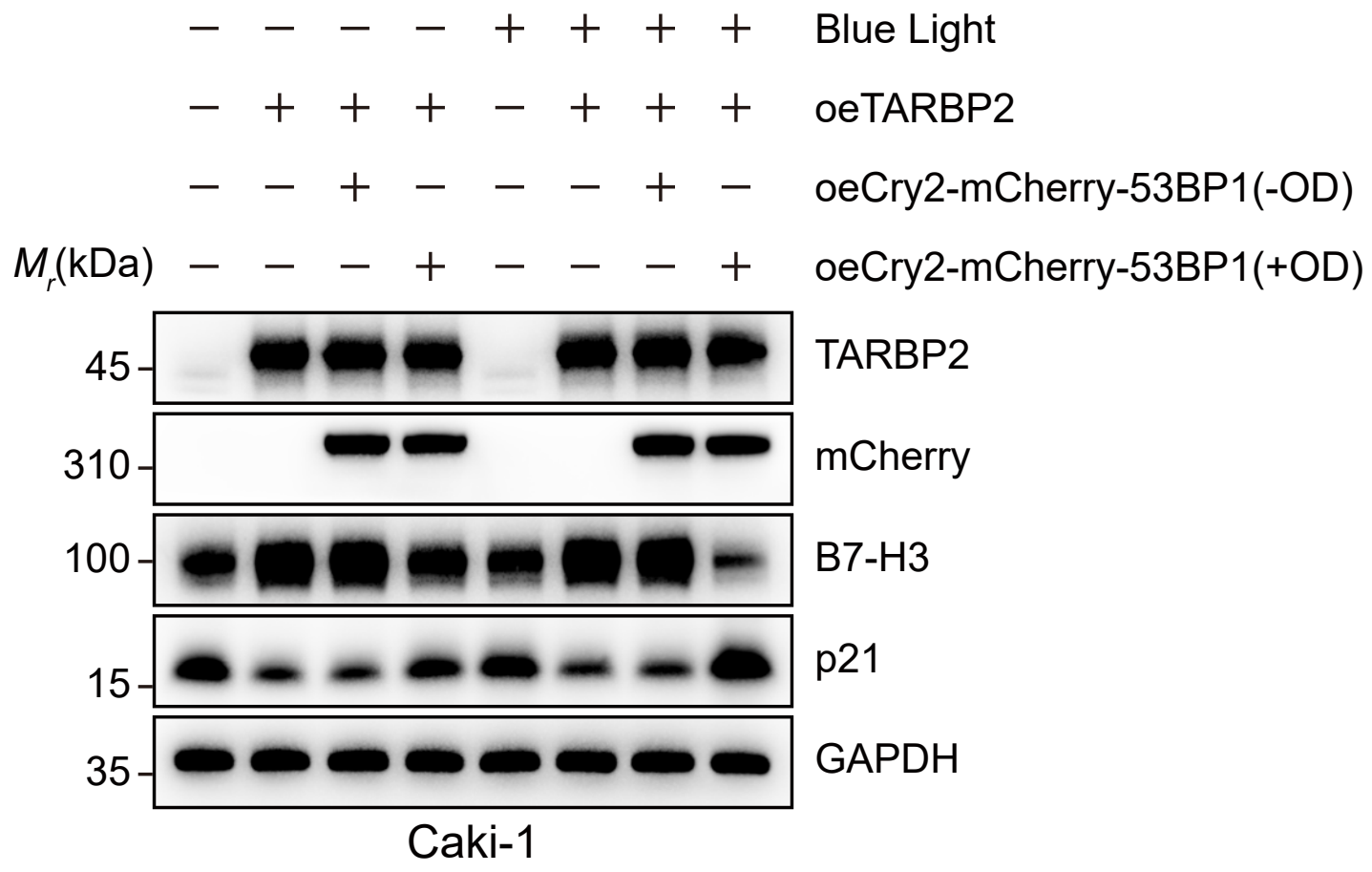

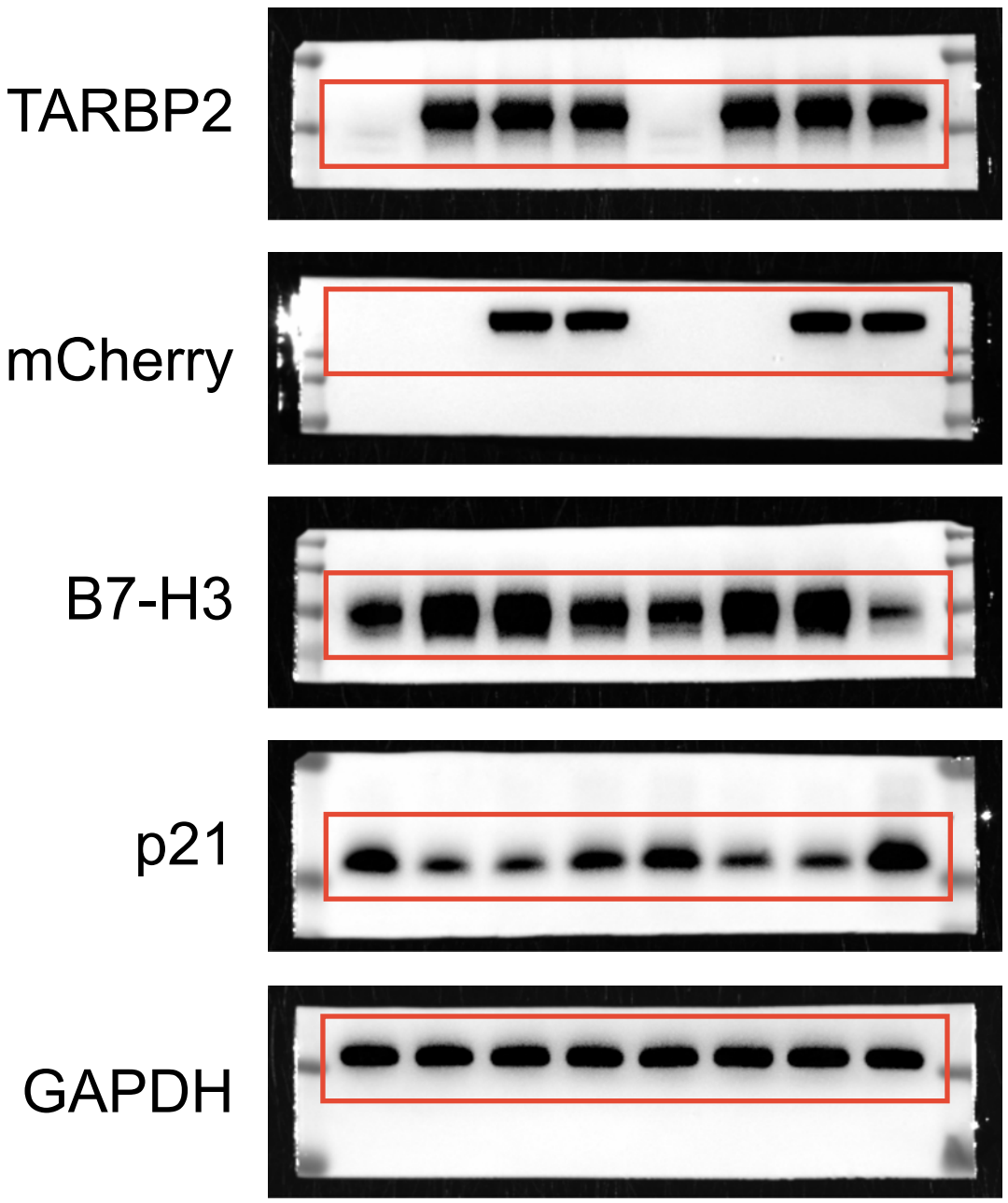

Supplement: Supplementary file 2 [file jci-136-199525-s225.pdf]
