# Supplementary material for: TARBP2 facilitates renal cell carcinoma immune escape by suppressing 53BP1 phase separation to upregulate B7-H3
Source: J Clin Invest. 2026 Nov 2;136(21):e199525. doi: 10.1172/JCI199525 (PMC13722402; doi:10.1172/JCI199525)
Supplement: Supplementary file 3 [file jci-136-199525-s008.pdf]

Supplemental data for

TARBP2 facilitates renal cell carcinoma immune escape by suppressing 53BP1 phase separation to upregulate B7-H3

This file includes:

Supplemental Methods

Supplemental Figures 1-19

Supplemental Methods

Cell lines and cell culture

Human RCC cell lines including 769-P (RRID: CVCL_1050), ACHN (RRID: CVCL_1067), and Caki-1 (RRID: CVCL_0234), along with the murine RCC cell line Renca (RRID: CVCL_2174), were acquired from the American Type Culture Collection (ATCC). Similarly, the human NK-92MI cell line (RRID: CVCL_3755) was also purchased from the ATCC. For cultivation, RPMI 1640 medium (Gibco) was employed for 769-P and Renca cell lines, DMEM medium (Gibco) for ACHN, and McCoy's 5A medium (Gibco) for Caki-1, each medium was supplemented with 10% fetal bovine serum (FBS; BioChannel) and 1% penicillin-streptomycin (Biosharp Biotechnology). NK-92MI cells were maintained in NK complete medium, formulated from alpha minimum essential medium enriched with the following additions: 2 mM L-glutamine, 1.5 g/L sodium bicarbonate, 0.2 mM inositol, 0.1 mM 2-mercaptoethanol, and 0.02 mM folic acid, along with 12.5% horse serum and 12.5% FBS. Ribonucleosides and deoxyribonucleosides were explicitly excluded, in line with earlier documented procedures (1). Vendor-conducted validations encompassed short tandem repeat (STR) profiling and routine Mycoplasma contamination testing for every cell line.

Plasmids and RNAi

Flag-tagged full-length *TARBP2* was cloned into the pCDH-CMV-MCS-EF1-Puro vector. Full-length *Tarbp2* was cloned into the pLVX-IRES-Puro vector. Full-length wild-type *TP53* and its mutants (R175H, R273H, and R248W), along with HA-tagged full-length and truncation mutants of *53BP1*, were cloned into the pcDNA3.1 vector. Cry2-mCherry-*53BP1* fusion constructs were generated by assembly of *53BP1* with or without the OD into the pCMV-CRY2-mCherry vector. To generate GFP-*53BP1*, full-length *53BP1* was cloned into the pEGFP-C1 vector. shRNAs targeting *Tarbp2*, *Trp53*, *B7h3*, *TARBP2*, and *TP53* were cloned into the pLKO.1 vector. All the plasmids were generated by Synbio Technologies (Soochow, China) and verified by sequencing before transfection. siRNAs targeting *Dicer* and *TARBP2* were synthesized by RiboBio (Guangzhou, China). The target sequences of shRNAs and siRNAs are listed in Supplemental Table 3.

B7h3 knockout cell generation by CRISPR-Cas9

Transfection-ready mouse B7-H3 CRISPR/Cas9 KO Plasmid (Santa Cruz, Cat#sc-430440) was transiently transfected into Renca cells. 48 hours after transfection, GFP positive cells were sorted by FACS and diluted for plating to form single-cell clones. Individual clones were subsequently validated by immunoblot analysis to establish stable *B7h3*-deficient Renca cells. The Control CRISPR/Cas9 Plasmid (Santa Cruz, Cat#sc-418922)

served as the negative control.

Analysis of public datasets

TPM normalized RNA-seq data from 33 TCGA cohorts were downloaded from the Genomic Data Commons (GDC) portal (https://portal.gdc.cancer.gov/), with tumor samples exclusively analyzed. Microarray expression data for the RCC E-MTAB-1980 cohort were acquired from ArrayExpress (https://www.ebi.ac.uk/biostudies/arrayexpress). RNA-seq data from the CheckMate cohort, comprising advanced RCC patients receiving ICB therapy, were sourced directly from the research article of Braun et al. (2). RNA-seq data from the Riaz2017_PD1_Melanoma cohort was accessed through the Gene Expression Omnibus (GEO) database (https://www.ncbi.nlm.nih.gov/geo/) under accession code GSE91061. Proteomic data for RCC patients from the CPTAC were retrieved from the Proteomic Data Commons (PDC) portal (https://pdc.cancer.gov/pdc/), with tumor samples selectively included. Gene expression profiles of RCC cell lines were obtained from the CCLE database (https://sites.broadinstitute.org/ccle). Corresponding clinical data with prognostic information for the TCGA-KIRC, E-MTAB-1980, CheckMate, and Riaz2017_PD1_Melanoma cohorts were collected to perform Cox proportional hazards regression and Kaplan-Meier survival analyses.

For integration analysis of publicly available scRNA-seq data, Seurat R package (version 5.1.0; RRID: SCR_016341) was applied. Seurat objects were initially constructed from individual gene expression matrices derived from 36 tissue samples of 13 advanced RCC patients. Quality control filtering retained cells with >200 genes, <7,500 genes, <25% mitochondrial genes, and <1% hemoglobin genes. DoubletFinder R package (version 2.0.4; RRID: SCR_018771) was used to detect and remove predicted doublets. These objects were then integrated into a merged Seurat object, which underwent normalization via NormalizeData function. Variable genes were detected using FindVariableFeatures function, with top 2,000 selected for downstream processing. Data scaling was conducted by ScaleData function, followed by principal component analysis (PCA) with RunPCA function. Subsequent integration and batch effect correction employed RunHarmony function from Harmony R package (version 1.2.0; RRID: SCR_022206). Cellular atlas was visualized through Uniform Manifold Approximation and Projection (UMAP), followed by clustering utilizing FindNeighbors function and FindClusters function with 30 principal components. Differentially expressed genes (DEGs) across clusters were identified using FindAllMarkers function. Cell types were annotated based on canonical marker expression and cluster-specific DEGs. For in-depth analysis, the “Epithelial/RCC cells” cluster was isolated and subjected to the same processing workflow as the full integrated dataset for transcriptomic characterization. CNV analysis of cells within the “Epithelial/RCC cells” cluster across different chromosomal regions was performed using the infercnv R package (version 1.20.0; RRID: SCR_021140). The Monocle R package (version 2.32.0; RRID: SCR_016339) was used

for trajectory analysis. The signature scores of immune response activation, a signature also known as GOBP_ACTIVATION_OF_IMMUNE_RESPONSE (GO:0002253; version 2025.1.Hs) derived from MsigDB (https://www.gsea-msigdb.org/gsea/msigdb), were calculated by AddModuleScore function from Seurat R package. The CellChat R package (version 1.6.1; RRID: SCR_021946) was applied to characterize ligand-receptor interactions between different cell types.

Tumor cell and CD45-positive intratumoral leukocyte isolation

Tumor tissues were harvested from in vivo mouse models and disaggregated via a mouse tumor dissociation kit (Miltenyi Biotec, Cat#130-096-730; RRID: SCR_020285), adhering strictly to manufacturer protocols to generate single-cell suspensions. From these suspensions, malignant tumor cells were subsequently purified with a mouse tumor cell isolation kit (Miltenyi Biotec, Cat#130-110-187) for downstream RNA-seq application. In parallel, CD45-positive intratumoral leukocytes underwent separation employing the CD45 (TIL) MicroBeads (Miltenyi Biotech, Cat#130-110-618) for flow cytometry analysis.

Flow cytometry

Unless otherwise specified, viability staining was performed in phosphate-buffered saline (PBS) for 30 minutes at 4 °C. Following blocking of Fc receptors on human cells using Human BD Fc Block (BD Biosciences, Cat#564219; RRID: AB_2728082) or on mouse cells using Purified Rat Anti-Mouse CD16/CD32 (BD Biosciences, Cat#553141; RRID: AB_394656), surface staining was conducted in FACS buffer (PBS containing 2% FBS and 2 mmol/L EDTA) with fluorochrome-conjugated antibodies for 30 minutes at 4 °C. For intracellular staining of FOXP3, the FOXP3 Fix/Perm Buffer Set (BioLegend, Cat#421403) was applied according to the manufacturer's instructions. For CD107a staining and intracellular staining of GZMB, TNF-α, and IFN-γ, cells were stimulated with 50 ng/mL phorbol 12-myristate 13-acetate (PMA; Sigma-Aldrich) and 1 μg/mL ionomycin (Sigma-Aldrich) for 5 hours at 37 °C in a humidified incubator, with fluorochrome-conjugated CD107a antibody and brefeldin A (BioLegend, Cat#420601) added after the first hour of stimulation. After viability staining, Fc receptor blocking, and surface marker labeling, cells were fixed and permeabilized using the Fixation/Permeabilization Kit (BD Biosciences, Cat#554714). Subsequently, intracellular staining was performed in 1× BD Perm/Wash buffer from the kit for 30 minutes at 4 °C. Upon completion of each step with antibody staining, cells were washed twice with FACS buffer and pelleted by centrifugation. Samples were analyzed using a Beckman CytoFLEX flow cytometer (Beckman Coulter, RRID: SCR_019627), and data analysis was conducted with FlowJo software (version 10.8.1; RRID: SCR_008520).

Tumor tissues from mouse tumor models were dissected and dissociated into single-cell suspensions as described

above and subsequently stained for flow cytometry. The following dyes and antibodies were used for staining cell suspensions from Renca-derived tumors in BALB/c mice: Zombie Aqua™ Fixable Viability Kit (BioLegend, Cat#423101), APC-Cy7 Rat Anti-Mouse CD45 (BD Biosciences, Cat#557659; RRID: AB_396774), PE/Cyanine7 anti-mouse CD3 (BioLegend, Cat#100219; RRID: AB_1732068), FITC Rat Anti-Mouse CD8a (BD Biosciences, Cat#553030; RRID: AB_394568), FITC anti-mouse CD4 (BioLegend, Cat#100509; RRID: AB_312712), FITC anti-mouse CD49b (BioLegend, Cat#108905; RRID: AB_313412), PE anti-mouse CD19 (BioLegend, Cat#152407; RRID: AB_2629816), FITC anti-mouse/human CD11b (BioLegend, Cat#101205; RRID: AB_312788), APC anti-mouse F4/80 (BioLegend, Cat#123115; RRID: AB_893493), Brilliant Violet 421™ anti-mouse FOXP3 (BioLegend, Cat#126419; RRID: AB_2565933), PE anti-mouse CD107a (LAMP-1) (BioLegend, Cat#121611; RRID: AB_1732051), APC anti-mouse CD107a (LAMP-1) (BioLegend, Cat#121613; RRID: AB_2134486), Brilliant Violet 421™ anti-human/mouse Granzyme B Recombinant (BioLegend, Cat#396413; RRID: AB_2810602), APC anti-mouse IFN-γ (BioLegend, Cat#505809; RRID: AB_315403), BV421 Rat Anti-Mouse TNF (BD Biosciences, Cat#566287; RRID: AB_2738173). The following dyes and antibodies were used for staining cell suspensions from PDX tumors in NCG mice: Zombie Aqua™ Fixable Viability Kit (BioLegend, Cat#423101), PE/Cyanine7 anti-human CD45 (BioLegend, Cat#304015; RRID: AB_314403), APC/Cyanine7 anti-human CD3 (BioLegend, Cat#300317; RRID: AB_314053), PE anti-human CD56 (NCAM) (BioLegend, Cat#318305; RRID: AB_604093), Brilliant Violet 421™ anti-human CD107a (LAMP-1) (BioLegend, Cat#328625; RRID: AB_10899581), APC anti-human IFN-γ (BioLegend, Cat#502511; RRID: AB_315236), Brilliant Violet 421™ anti-human TNF-α (BioLegend, Cat#502931; RRID: AB_10898321). For measurement of B7-H3 surface expression, MACS-isolated tumor cells from Renca-derived tumors were stained with Zombie Aqua™ Fixable Viability Kit (BioLegend, Cat#423101), APC-Cy7 Rat Anti-Mouse CD45 (BD Biosciences, Cat#557659; RRID: AB_396774), and PE Rat anti-Mouse CD276 (BD Biosciences, Cat#562357; RRID: AB_11153846), with PE Rat IgG2a (BD Biosciences, Cat#553930; RRID: AB_11153846) used as isotype control. Cultured human RCC cells were stained with Zombie Aqua™ Fixable Viability Kit (BioLegend, Cat#423101) and APC anti-human CD276 (B7-H3) (eBioscience, Cat#17-2769-42; RRID: AB_2573190), with APC Mouse IgG1 (eBioscience, Cat#17-4714-82; RRID: AB_763649) used as isotype control. Fluorochrome-conjugated antibodies and dyes employed in other experiments are described in the corresponding sections of Supplemental Methods.

scRNA-seq from mouse tumor model

Tarbp2 knockdown and control Renca-derived tumors from BALB/c mice were dissected and dissociated into single-cell suspensions as described above. Subsequently, the single-cell suspensions were stained with fluorescent antibodies and dyes, including the Zombie Green™ Fixable Viability Kit (BioLegend, Cat#423111),

APC-Cy7 Rat Anti-Mouse CD45 (BD Biosciences, Cat#557659; RRID: AB_396774), PE/Cyanine7 anti-mouse CD3 (BioLegend, Cat#100219; RRID: AB_1732068), APC anti-mouse CD335 (NKp46) (BioLegend, Cat#137607; RRID: AB_10612749). Live $CD45^{+}CD3^{-}NKp46^{+}$ cells were FACS-sorted using the BD FACSMelody™ Cell Sorter (BD Biosciences; RRID: SCR_023209) for further scRNA-seq.

Prior to cell capture, FACS-sorted cell suspensions were centrifuged and resuspended in PBS to a density of 700-1200 cells/μl. Cell viability assessed via trypan blue staining method exceeded 85%. The resuspended cells were then applied to the 10x Chromium chip following the instructions of the 10x Genomics Chromium Single-Cell 3' kit (V3). cDNA amplification and library construction were executed by LC-Bio Technology (Hangzhou, China), followed by sequencing on an Illumina NovaSeq 6000 sequencing system (paired-end, 150 bp; RRID: SCR_016387) at a minimum depth of 20,000 reads per cell.

Results from Illumina sequencing offline were converted to FASTQ format using bcl2fastq software (version 5.0.1; RRID:SCR_015058). Subsequent alignment to the reference genome, cell barcode identification, and unique molecular identifier (UMI) quantification were performed using Cell Ranger software (version 7.0.0).

Data from the CellRanger output (features, barcodes, and matrices) were analyzed using the Seurat workflow as described for the analysis of publicly available scRNA-seq datasets. Briefly, individual Seurat objects were created for each sample, followed by quality control and doublet removal. These objects were then merged into a single Seurat object. Subsequent processing involved normalization, identification of high-variance genes, data scaling, PCA dimensionality reduction, harmony integration, UMAP dimensionality reduction, and cell clustering. The cluster representing cycling cells (cluster 7) underwent re-integration and label transfer using the tl.ingest function within Scanpy python tool (version 1.10.4; RRID: SCR_018139). Cell cycle regression was conducted on these cycling cells via Scanpy's pp.regress_out function prior to re-integration. Trajectory inference was performed employing the Monocle R package (version 2.32.0; RRID: SCR_016339). For GO and KEGG pathway enrichment analyses, hypergeometric tests were performed using DEGs identified by Seurat's FindAllMarkers function for each cluster relative to all other clusters. Cellular pathway activity was quantified using Seurat's AddModuleScore function for gene set scoring. Relevant gene sets included the TGF-β signaling signature also known as HALLMARK_TGF_BETA_SIGNALING (version 2025.1.Mm) obtained from MsigDB, and gene sets derived from published papers. Regulatory network inference for transcription factors was conducted using pySCENIC (version 0.12.1; RRID: SCR_025802).

Isolation of human primary RCC cells

Histologically confirmed Human RCC tissues were processed under sterile conditions. Tissues were washed with PBS (4°C) supplemented with 2% penicillin-streptomycin and mechanically dissociated into small fragments.

Enzymatic digestion was performed in DMEM containing 0.2% collagenase IV (STEMCELL Technologies), 0.01% hyaluronidase (STEMCELL Technologies), 0.002% DNase I (STEMCELL Technologies), and 3 × 10^{-3} mol/L $CaCl_2$ (Sigma-Aldrich, Cat#21115) for 60 minutes at 37°C with 200 rpm agitation. The resultant suspension was centrifuged at 300 × g and the supernatant was filtered through a 40-μm cell strainer (Corning, Cat#352340). Cells collected from the filtrate were seeded into 6-well culture plates containing DMEM supplemented with 10% FBS and 1% penicillin-streptomycin.

Human peripheral blood NK cell isolation and pre-activation

Human PBMCs were purified by density gradient centrifugation employing Ficoll reagent (Cytiva, Cat#17144002). The isolated PBMCs were then stained with the Zombie Green™ Fixable Viability Kit (BioLegend, Cat#423111), PE/Cyanine7 anti-human CD45 (BioLegend, Cat#304015; RRID: AB_314403), APC/Cyanine7 anti-human CD3 (BioLegend, Cat#300317; RRID: AB_314053), and PE anti-human CD56 (NCAM) (BioLegend, Cat#318305; RRID: AB_604093). Live $CD45^{+}CD3^{-}CD56^{+}$ NK populations were subsequently sorted using the BD FACSMelody™ Cell Sorter (BD Biosciences; RRID: SCR_023209). The FACS-sorted NK cells were activated by culturing in complete RPMI 1640 medium (Gibco) containing IL-2 (100U/ml; Peprotech) and IL-15 (20 ng/ml; Peprotech) for 24 hours as previously described (3), before they were used in NK cell cytotoxicity assays.

NK cell cytotoxicity assay

Adherent RCC cells at exponential growth phase underwent detachment via trypsin and were labeled with CFSE using the CFSE Cell Division Tracker Kit (BioLegend, Cat#423801) in compliance with the manufacturer's instructions. RCC cells were then seeded into flat-bottom 96-well plates at a density of 30,000 cells per well with 100 μL pre-warmed culture medium, followed by a 6-hour incubation at 37°C to facilitate attachment. The medium was aspirated gently and replaced with 100 μL fresh medium containing specified compounds, alongside or without pre-activated human peripheral blood NK cells or NK-92MI cells at designated E:T ratios. Co-culturing proceeded for 8 hours at 37°C. After incubation, supernatants were collected, and adherent cells were detached with trypsin and combined with the corresponding supernatants. Apoptosis was evaluated through staining with Alexa Fluor® 647 Annexin V (BioLegend, Cat#640912) and 5 μg/mL DAPI (Beyotime Biotechnology, Cat#C1002) in Annexin V Binding Buffer (BioLegend, Cat#422201) for 15 minutes at room temperature. Samples were cooled on ice and analyzed immediately via flow cytometry to quantify apoptotic rates in CFSE-positive RCC cells. To detect NK cell function, co-cultured cells were stained with the Zombie Aqua™ Fixable Viability Kit (BioLegend, Cat#423101), Brilliant Violet 421™ anti-human CD107a (LAMP-1) (BioLegend, Cat#328625; RRID: AB_10899581), APC anti-human IFN-γ (BioLegend, Cat#502511; RRID: AB_315236), and Brilliant Violet 421™ anti-human TNF-α

(BioLegend, Cat#502931; RRID: AB_10898321), for flow cytometry analysis of CFSE-negative cells.

The cytotoxicity of murine primary NK cells against Renca cells was assessed as described above using 0.4 μm Transwell inserts (Corning, Cat#3470) with the following modifications. CFSE-labeled Renca cells were plated on the bottom of 24-well plates at a density of 120,000 cells per well. Pre-activated murine primary NK cells were added either to the upper chamber of the Transwell inserts for indirect co-culture or directly to the lower chamber for direct co-culture with Renca cells. Apoptosis of CFSE-positive Renca cells was analyzed by flow cytometry as described above. CFSE-negative NK cells were subsequently FACS-sorted for RNA extraction, followed by downstream RT-qPCR analysis to assess transcriptional changes.

RNA-seq

For RNA-seq of FFPE samples, total RNA was isolated and purified using the PureLink™ FFPE RNA Isolation Kit (Invitrogen, Cat#K156002) following the manufacturer's procedure. Concentration and purity quality control of total RNA was conducted using the NanoDrop ND-1000 (Thermo Scientific; RRID: SCR_016517), while RNA integrity quality control was performed using the Bioanalyzer 2100 (Agilent; RRID: SCR_019715). cDNA preparation, amplification, and library construction were conducted by LC-Bio Technology (Hangzhou, China). Briefly, utilizing the SMARTer® Stranded Total RNA-Seq Kit v2 - Pico Input Mammalian Kit (Takara Bio Inc.), cDNA was generated via universal priming and amplified. Ribosomal RNA-derived cDNA sequences were subsequently depleted using ZapR v2 with mammalian-specific R-Probes v2 to complete cDNA library construction. For RNA-seq of MACS-isolated tumor cells, TRIzol reagent (Invitrogen, Cat#15596018) was employed for total RNA extraction, with RNA quality verified using Bioanalyzer 2100 (Agilent; RRID: SCR_019715) and RNA 6000 Nano LabChip Kit (Agilent, Cat#5067-1511). mRNA enrichment, mRNA reverse transcription into cDNA, and cDNA library construction were conducted using standard protocols by LC-Bio Technology (Hangzhou, China). Both libraries from FFPE samples and tumor cells were sequenced by LC-Bio Technology (Hangzhou, China) on an Illumina NovaSeq 6000 sequencing system (RRID: SCR_016387) using the Illumina paired-end RNA-seq approach.

High-quality sequencing reads were mapped to the reference genomes (GRCh38.p14 for human; GRCm39 for mouse) using the HISAT2 (RRID: SCR_015530) alignment tool. Differential gene expression analysis was performed by DESeq2 R package (RRID: SCR_015687) between two different groups. Genes meeting the criteria of $|\log2(\text{fold change})|>1$ and $P<0.05$ were considered DEGs. KEGG pathway enrichment analysis and GSEA were performed using OmicStudio (4).

Cell proliferation experiments

Cell proliferation was assessed using CCK-8 and colony formation assays. For CCK-8 assay, cells (1,000 cells/well)

were seeded within flat-bottom 96-well plates with 100 μL culture medium in 3-5 replicates. At the indicated time points, the medium was replaced with 100 μL serum-free medium containing 10% CCK-8 reagent (MedChemExpress, Cat#HY-K0301). After incubation for 2 hours, absorbance at 450 nm was measured using a VersaMax microplate reader (Molecular Devices). For colony formation assay, cells (1,000 cells/well) were seeded in flat-bottom 6-well plates and cultured for 7-10 days. Resultant colonies were subsequently fixed in paraformaldehyde and stained with crystal violet for quantification.

HE staining and TUNEL assay

Paraffin-embedded tissue sections underwent baking at 56°C for 30 minutes. Sections were sequentially dewaxed in xylene and rehydrated through graded ethanol solutions. Nuclear staining was performed using hematoxylin (Servicebio, Cat#G1004). Sections were then briefly dipped in acid ethanol (0.5 % hydrochloric acid in 70% ethanol), rinsed in distilled water, and counterstained with eosin (Servicebio, Cat#G1001) for cytoplasmic visualization. TUNEL assay was performed with the TMR (red) Tunel Cell Apoptosis Detection Kit (Servicebio, Cat#G1502) according to manufacturer's instructions.

IHC

Paraffin-embedded tissue sections were baked at 56°C for 30 minutes, followed by sequential dewaxing in xylene and rehydration through an ethanol gradient. Antigen retrieval was conducted under pressurized heating in citrate buffer solution. After cooling to ambient temperature, endogenous peroxidase activity was quenched by 3% hydrogen peroxide treatment for 10 minutes. Sections underwent blocking with normal goat serum blocking buffer (ZSGB-BIO, Cat#ZLI-9056) at 37°C for 60 minutes before overnight incubation with primary antibodies at 4°C. Subsequently, a secondary antibody (ZSGB-BIO, Cat#PV-6000) was used to bind the primary antibody at 37°C for 1 hour. Target proteins were visualized using 3,3′-diaminobenzidine (DAB; ZSGB-BIO, Cat#ZLI-9017), followed by nuclear counterstaining with hematoxylin (Servicebio, Cat#G1004). Primary antibodies used for identifying human antigen included mouse anti-TARBP2 (Proteintech, Cat#66792-1-Ig; RRID: AB_2882136), rabbit anti-B7-H3 (CST, Cat#14058; RRID: AB_2750877), and rabbit anti-Cleaved Caspase-3 (CST, Cat#9664; RRID: AB_2070042). For identifying mouse antigen, rabbit anti-Ki67 (CST, Cat#12202; RRID: AB_2620142) was used.

To assess antigen expression in tissue sections, H-scores were independently assessed by two pathologists based on the proportion of positively stained cells and staining intensity. Proportional score represented the percentage of stained cells, ranging from 0% to 100%. Staining intensity was graded on a 0-3 scale: 0 for negative, 1 for weak, 2 for moderate, and 3 for strong. The final H-score was calculated as: (3 × percentage of strongly stained cells) + (2 × percentage of moderately stained cells) + (1 × percentage of weakly stained cells), producing a score from 0

to 300.

mIHC

For mIHC staining of FFPE sections, a 7-color mIHC PLUS kit (FreeThinking, Nanjing, China; Cat#FH37100-p) based on the tyramide signal amplification (TSA) method was used according to the manufacturer's instructions. Briefly, FFPE sections were baked and dewaxed, followed by antigen retrieval and hydrogen peroxide treatment. Subsequently, sections were blocked and sequentially stained with one of the indicated primary antibodies, a horseradish peroxidase (HRP)-conjugated secondary antibody and one of the TSA dyes. Another round of blocking and staining was performed after microwave treatment to elute the antibodies from the previous round. After completion of all TSA staining procedures, nuclei were counterstained with DAPI. Sections were visualized and scanned using the Vectra Polaris Automated Quantitative Pathology Imaging System (RRID: SCR_025508). Digital images were analyzed with QuPath (RRID: SCR_018257). TSA dyes included Opal 520 in green pseudo-color for TARBP2, Opal 780 in white pseudo-color for B7-H3, Opal 570 in gold pseudo-color for CD3, Opal 690 in red pseudo-color for CD56, and Opal 620 in magenta pseudo-color for PanCK. Primary antibodies included mouse anti-TARBP2 (Proteintech, Cat#66792-1-Ig; RRID: AB_2882136), rabbit anti-B7-H3 (CST, Cat#14058; RRID: AB_2750877), rabbit anti-CD3 (Proteintech, Cat#17617-1-AP; RRID: AB_1939430), rabbit anti-CD56 (Abcam, Cat#ab75813; RRID: AB_2632384), and mouse anti-PanCK (Abcam, Cat#ab7753; RRID: AB_306047).

RT-qPCR

Total RNA was extracted from specified cellular or tissue samples using TRIzol reagent (Invitrogen, Cat#15596018). Subsequent cDNA synthesis was performed on 1 μg of isolated RNA utilizing the PrimeScript™ RT Reagent Kit (Takara Bio Inc., Cat#RR036A). qPCR was then conducted using the 2× Color SYBR Green qPCR Master Mix (ROX2) (EZBioscience, Cat#A0012-R2) and a Roche LightCycler 480 Instrument (RRID: SCR_018626) according to the manufacturer's instructions. mRNA expression levels of target genes were quantified using specific forward and reverse primers listed in Supplemental Table 4, following normalization to that of endogenous GAPDH.

Chromatin immunoprecipitation

Chromatin immunoprecipitation (ChIP) was performed using the SimpleChIP® Enzymatic Chromatin IP Kit (CST, Cat#9003) according to the manufacturer's instructions. Briefly, cells were fixed with formaldehyde to crosslink histone and non-histone proteins to DNA. Chromatin was then enzymatically digested into DNA-protein fragments, followed by immunoprecipitation with antibody-conjugated magnetic beads. The precipitated DNA-protein

fragments were subsequently de-crosslinked, and the DNA was purified for qPCR analysis using primers targeting the *B7-H3* promoter (Supplemental Table 4). Antibodies used for immunoprecipitation included rabbit anti-p53 (CST, Cat#2527; RRID: AB_10695803) and rabbit control IgG (CST, Cat#2729; RRID: AB_1031062).

Dual-luciferase reporter assay

The promoter sequence of *B7-H3* was cloned into the pGL4 luciferase reporter vector by Synbio Technologies (Soochow, China), with construct integrity validated by DNA sequencing. Human RCC cells were co-transfected with either the promoter-containing recombinant pGL4-*B7-H3* plasmid or the empty pGL4 backbone, alongside a Renilla luciferase-expressing pRL vector (Promega) as an internal control. Following a 48-hour incubation period, luciferase activity was quantified using the Dual-Luciferase® Reporter Assay System (Promega, Cat#E1980) according to the manufacturer's specifications, with luminescence signals captured using a Tecan SPARK Multimode Microplate Reader (RRID: SCR_021897). Firefly luciferase activity was normalized to Renilla luciferase activity to control for transfection efficiency.

Subcellular fractionation

Nuclear-cytoplasmic separation was carried out using the ProteinExt® Mammalian Nuclear and Cytoplasmic Protein Extraction Kit (TransGen Biotech, Cat#DE201-01) in compliance with the manufacturer's specifications. Chromatin-bound proteins were isolated from cultured cells by employing the Subcellular Protein Fractionation Kit (Thermo Scientific, Cat#78840) adhering to the manufacturer's protocols. Lysates obtained from different subcellular fractions were subsequently subjected to immunoprecipitation, western blotting, and mass spectrometry.

Immunoprecipitation

Cells were lysed on ice using cell lysis buffer (Beyotime Biotechnology, Cat#P0013) supplemented with protease inhibitor cocktail (CWBIO, Cat#CW2200S). Lysates were centrifuged to collect the supernatants, followed by protein quantification using a bicinchoninic acid (BCA) assay kit (Invitrogen, Cat#23227). A subset of protein lysates was collected as input controls. Then, equilibrated lysates were subjected to incubation with protein A/G magnetic beads (MedChemExpress, Cat#HY-K0202) at 4°C for 1 hour to reduce nonspecific binding. The beads were then discarded, and fresh protein A/G magnetic beads pre-rinsed and coupled with targeted antibodies, were introduced. Alternatively, pre-coated magnetic beads, including anti-Flag (Selleck, Cat#B26101) and anti-HA (Selleck, Cat#B26201) types, were directly added to the lysates. Following overnight incubation with rotation at 4°C, magnetic beads underwent four sequential washes using chilled cell lysis buffer, each lasting 10 minutes at

4°C. Proteins from magnetic beads and input controls were subsequently denatured with SDS buffer for downstream western blotting evaluation. Antibodies used in immunoprecipitation included mouse anti-TARBP2 (Santa Cruz, Cat#sc-514124; RRID: AB_2732792), mouse anti-53BP1 (Santa Cruz, Cat#sc-517281; RRID: AB_2921289), mouse anti-p53 (Santa Cruz, Cat#sc-126; RRID: AB_628082), and mouse control IgG (Proteintech, Cat#B900620; RRID: AB_2883054).

Western Blotting

Protein lysates were prepared and quantified as described above. After denaturation with SDS buffer, denatured protein samples were separated by 7.5-12.5% SDS-PAGE gels and then transferred to polyvinylidene fluoride (PVDF) membranes (Roche, Cat#03010040001). After blocking, membranes were incubated with primary antibodies overnight at 4°C. Subsequent incubation with HRP-conjugated secondary antibodies proceeded for 1 hour at room temperature. Protein bands were detected using the BioRad ChemiDoc Touch Imaging System (RRID:SCR_021693). Primary antibodies used in western blotting included rabbit anti-TARBP2 (MedChemExpress, Cat#HY-P81688; RRID: AB_3103795), mouse anti-TARBP2 (Santa Cruz, Cat#sc-514124; RRID: AB_2732792), rabbit anti-B7-H3 (Abcam, Cat#ab134161; RRID: AB_2687929), rabbit anti-p53 (CST, Cat#2527; RRID: AB_10695803), rabbit anti-p21 (CST, Cat#2947; RRID: AB_823586), rabbit anti-53BP1 (CST, Cat#4937; RRID: AB_10694558), rabbit anti-Dicer (CST, Cat#5362; RRID: AB_10692484), rabbit γ-H2AX (CST, Cat#9718; RRID: AB_2118009), rabbit anti-Flag (CST, Cat#14793; RRID: AB_2572291), rabbit anti-HA (CST, Cat#3724; RRID: AB_1549585), rabbit anti-mCherry (Abcam, Cat#ab167453; RRID: AB_2571870), rabbit anti-Lamin B1 (CST, Cat#12586; RRID: AB_2650517), rabbit anti-Histone H3 (CST, Cat#4499; RRID: AB_10544537), rabbit anti-Histone H4 (Proteintech, Cat#16047-1-AP; RRID: AB_2118625), and mouse anti-GAPDH (Proteintech, Cat#60004-1-Ig; RRID: AB_2107436). HRP-conjugated secondary antibodies included HRP-conjugated goat anti-rabbit IgG (Proteintech, Cat#SA00001-2; RRID: AB_2722564) and HRP-conjugated goat anti-mouse IgG (Proteintech, Cat#SA00001-1; RRID: AB_2722565).

Mass spectrometry

Wild-type 769-P cells were fractionated into nuclear and cytoplasmic components, and each fraction was subjected to immunoprecipitation using either a TARBP2 monoclonal antibody or control IgG as described above. The eluted proteins were then subjected to high-performance liquid chromatography-tandem mass spectrometry (HPLC-MS/MS) analysis by BGI TechSolutions (Shenzhen, China). Protein identification utilized Mascot (version 2.3; RRID: SCR_014322) with database searches based on UniProt (RRID: SCR_002380).

Immunofluorescence (IF)

Adherent RCC cells were washed with PBS, fixed in 4% paraformaldehyde for 15 minutes, and permeabilized using 0.5% Triton X-100 for 30 minutes at room temperature. After blocking with 5% bovine serum albumin (BSA) for 1 hour at room temperature, cells were incubated with indicated primary antibodies overnight at 4 °C. After incubation, cells were washed with PBS, and incubated with fluorochrome-conjugated secondary antibodies for 1 hour at room temperature. DAPI was incorporated to label nuclei. For plasma membrane labeling, live cells were stained using the MemBrite® Fix 488/515 Cell Surface Staining Kit (Biotium, Cat#30093-T) prior to paraformaldehyde fixation. Image acquisition utilized the Zeiss LSM 880 with Airyscan Confocal Laser Scanning Microscope (RRID: SCR_020925), with data analysis executed by Fiji software (RRID: SCR_002285). For three-dimensional image acquisition, super-resolution imaging was conducted using a Nikon structured illumination microscopy (N-SIM; RRID: SCR_023803) equipped with a 100×/1.49 NA oil immersion objective. A z-stack comprising 20 optical sections spaced at 0.2 μm intervals along the z-axis was acquired. Subsequent reconstruction of three-dimensional volumes and analysis of colocalization were executed with Imaris software (RRID: SCR_007370). Primary antibodies used in IF staining included mouse anti-TARBP2 (Santa Cruz, Cat#sc-514124; RRID: AB_2732792), rabbit anti-B7-H3 (Abcam, Cat#ab134161; RRID: AB_2687929), rabbit anti-53BP1 (CST, Cat#4937; RRID: AB_10694558), and mouse anti-p53 (Santa Cruz, Cat#sc-126; RRID: AB_628082). Fluorochrome-conjugated secondary antibodies included CoraLite488-conjugated Goat Anti-Mouse IgG (Proteintech, Cat#SA00013-1; RRID: AB_2810983), CoraLite488-conjugated Goat Anti-Rabbit IgG (Proteintech, Cat#SA00013-2; RRID: AB_2797132), and CoraLite594-conjugated Goat Anti-Rabbit IgG (Proteintech, Cat#SA00013-4; RRID: AB_2810984).

PLA

Adherent RCC cells were washed with PBS, fixed in 4% paraformaldehyde for 15 minutes, and permeabilized with 0.5% Triton X-100 for 30 minutes at room temperature. PLA was executed with the Duolink® In Situ Red Starter Kit Mouse/Rabbit (Sigma-Aldrich, Cat#DUO92101), adhering strictly to manufacturer's instructions, incorporating steps of blocking, primary antibody incubation, probe incubation, ligation reaction, and amplification reaction. Further Image acquisition was performed using Zeiss LSM 880 with Airyscan Confocal Laser Scanning Microscope (RRID: SCR_020925). PLA puncta quantification employed Fiji software (RRID: SCR_002285). Primary antibodies targeting TARBP2, 53BP1 and p53 corresponded to those outlined in IF.

Blue light-induced optoDroplet formation

Prior to photostimulation, RCC cells were seeded into a 1.5H glass-bottom 96-well plate (Cellvis, Cat#P96-1.5H-

N). Subsequently, cells were transfected with constructed plasmids encoding Cry2-mCherry-53BP1 fusion proteins with or without the OD at about 50% confluency. Following a 36-hour incubation post-transfection, optoDroplet induction was initiated using the Cytiva IN Cell 2500HS (RRID: SCR_026891) as previously described (5). Briefly, cells were exposed to blue light pulses (473 nm excitation wavelength, 28 nm bandpass) with a pulse duration of 25 milliseconds delivered every 15 seconds for a total of 10 minutes. Immediately after optoDroplet induction, cells underwent sequential PBS washing, fixation in 4% formaldehyde, permeabilization, and anti-p53 immunostaining for confocal imaging, as standardized above. Alternatively, cells were further cultured for 12 hours following optoDroplet induction and then harvested for Western blotting or flow cytometry, as described above.

FRAP

Before conducting FRAP, RCC cells were plated into 35-mm glass-bottom culture dishes. Upon reaching about 50% confluency, cells were transiently transfected with GFP-53BP1 expression plasmids. FRAP experiments commenced after a 36-hour incubation using a Nikon AXR NSPARC super resolution inverted microscope (RRID: SCR_026538) with a 100× oil immersion objective. Throughout measurements, live cells were sustained at 37°C under 5% CO_2. An outlined spot for bleaching was first scanned under low laser power (3% maximum power) at 488 nm for 30 seconds at 10-second intervals to ensure pre-bleach stability of its fluorescence intensity. Subsequently, the outlined spot was photobleached using a 488-nm laser at 40% of maximum power with a single exposure duration of 2.69 seconds. Fluorescence recovery of the spot was monitored using low laser power (3% maximum power) at 488 nm every 10 seconds over 120 seconds to approach fluorescence intensity stabilization. For quantification, fluorescence intensity in an outlined spot was adjusted by subtracting average background intensity in three unbleached areas in the same image. This adjusted fluorescence intensity was normalized to the mean adjusted pre-bleach fluorescence intensity of multiple spots from independent cells and plotted as a function of time (in seconds).

Murine primary NK cell isolation and pre-activation

Murine primary NK cells were isolated from spleens of BALB/c mice. Spleens were minced using a syringe plunger, washed in PBS, and centrifuged to acquire cell pellets. NK cells were subsequently purified via negative selection employing the MojoSort™ Mouse NK Cell Isolation Kit (Biolegend, Cat#480050) adhering to the manufacturer's instructions. Murine primary NK cells were cultured in RPMI 1640 medium (Gibco) supplemented with 10% FBS and IL-15 (10 ng/ml; Peprotech), and were activated with IL-12 (25 ng/ml; R&D Systems), IL-15 (50 ng/ml; Peprotech) and IL-18 (5 ng/ml; R&D Systems) for 18 hours as previously described (6) for further tail vein injections.

Animal experiments

Male NCG mice (NOD/ShiLtJGpt-Prkdc^{em26Cd52}Il2rg^{em26Cd22}/Gpt; RRID: IMSR_GPT:T001475) and BALB/c mice (BALB/cJGpt; RRID: IMSR_GPT:N000020), aged 6-8 weeks, were sourced from GemPharmatech and housed under pathogen-free conditions.

For Renca-derived subcutaneous tumor models, NCG and BALB/c mice were subcutaneously injected with stably transfected Renca cells ($5x10^5$ cells/mouse in 100 μL PBS). To deplete $CD8^+$ T cells, $CD4^+$ T cells, or NK cells in BALB/c mice, InVivoPlus anti-mouse CD8α (200 μg/mouse in 100 μL PBS; BioXCell, Cat#BP0117; RRID: AB_10950145), InVivoPlus anti-mouse CD4 (200 μg/mouse in 100 μL PBS; BioXCell, Cat#BP0003-1; RRID: AB_1107636), or Ultra-LEAF™ Purified anti-Asialo-GM1 Antibody (50 μL/mouse; Biolegend, Cat#146002; RRID: AB_2562206) were intraperitoneally injected 4 days and 1 day before Renca cell injection, followed by consecutive injections every 3 days. InVivoPlus rat IgG2b isotype control (BioXCell, Cat#BP0090; RRID: AB_1107780) and Purified Rabbit Polyclonal Isotype Ctrl Antibody (Biolegend, Cat#910801; RRID: AB_2722735) were used as isotype controls.

For the PDX model, fresh human RCC tumor fragments were implanted subcutaneously in NCG mice. When tumors reached approximately 100 mm^3, mice were euthanized, and tumors were dissociated and cut into 1 mm^3 fragments. These fragments were then engrafted into the next generation NCG mice. A stable RCC PDX model was achieved after three serial transplant passages. Based on this model, at the 100 mm^3 tumor volume milestone, intravenous tail vein injections of either PBS or $5x10^6$ NK-92MI cells in PBS (100 μL/mouse) were given every 7 days. Simultaneously, intratumoral injections of control or *TARBP2*-targeting siRNA (5 nmol/mouse) were administered every 4 days.

A standard formula, $\pi/6 \times \text{length} \times \text{width}^2$, was used to estimate subcutaneous tumor volume. Measurements started when the largest tumor reached approximately 100 mm^3, and were subsequently performed every 2, 3, or 7 days based on growth kinetics. Mice were sacrificed before tumors reached 2,000 mm^3. Tumors were surgically excised for photographic documentation and subsequent experiments.

For the Renca-derived orthotopic tumor model, BALB/c mice were anesthetized with pentobarbital sodium (50 mg/kg) by intraperitoneal injection. Then, stably transfected Renca cells ($2.5x10^5$ cells/mouse in 25 μL PBS) were orthotopically injected into the renal subcapsular space of mice. Four days after inoculation, intravenous tail vein injections of either PBS or $5x10^6$ activated murine primary NK cells labeled with PKH26 (Sigma-Aldrich, Cat#MINI26) in PBS (100 μL/mouse) were given every 4 days. Mice also received intraperitoneal injections of InVivoMAb anti-mouse PD-1 (200 μg/mouse; BioXCell, Cat#BE0146; RRID: AB_10949053) or InVivoMAb anti-mouse CD276 (B7-H3) (300 μg/mouse; BioXCell, Cat#BE0124; RRID: AB_10950149) at the same days as the tail vein injections of PBS or NK cells. Corresponding isotype controls were administered using InVivoMAb rat IgG2a

isotype control (200 μg/mouse; BioXCell, Cat#BE0089; RRID: AB_1107769) or InVivoMAb rat IgG1 isotype control (300 μg/mouse; BioXCell, Cat#BE0088; RRID: AB_1107775). For tumor growth monitoring, mice were injected with D-luciferin sodium salt (Yeasen), and bioluminescence signals were analyzed using Perkin Elmer IVIS Spectrum In Vivo Imaging System (RRID: SCR_018621). Mice were euthanized on day 18, and kidneys bearing orthotopic tumors were harvested for subsequent experiments.

References

1. Bugide S, et al. Inhibition of Enhancer of zeste homolog 2 (EZH2) induces natural killer cell-mediated eradication of hepatocellular carcinoma cells. *Proc Natl Acad Sci U S A.* 2018;115(15):E3509-E3518.
2. Braun DA, et al. Interplay of somatic alterations and immune infiltration modulates response to PD-1 blockade in advanced clear cell renal cell carcinoma. *Nat Med.* 2020;26(6):909-918.
3. Zheng X, et al. Mitochondrial fragmentation limits NK cell-based tumor immunosurveillance. *Nat Immunol.* 2019;20(12):1656-1667.
4. Lyu F, et al. OmicStudio: A composable bioinformatics cloud platform with real-time feedback that can generate high-quality graphs for publication. *Imeta.* 2023;2(1):e85.
5. Kilic S, et al. Phase separation of 53BP1 determines liquid-like behavior of DNA repair compartments. *EMBO J.* 2019;38(16):e101379.
6. Michelet X, et al. Metabolic reprogramming of natural killer cells in obesity limits antitumor responses. *Nat Immunol.* 2018;19(12):1330-1340.

Supplemental Figure 1

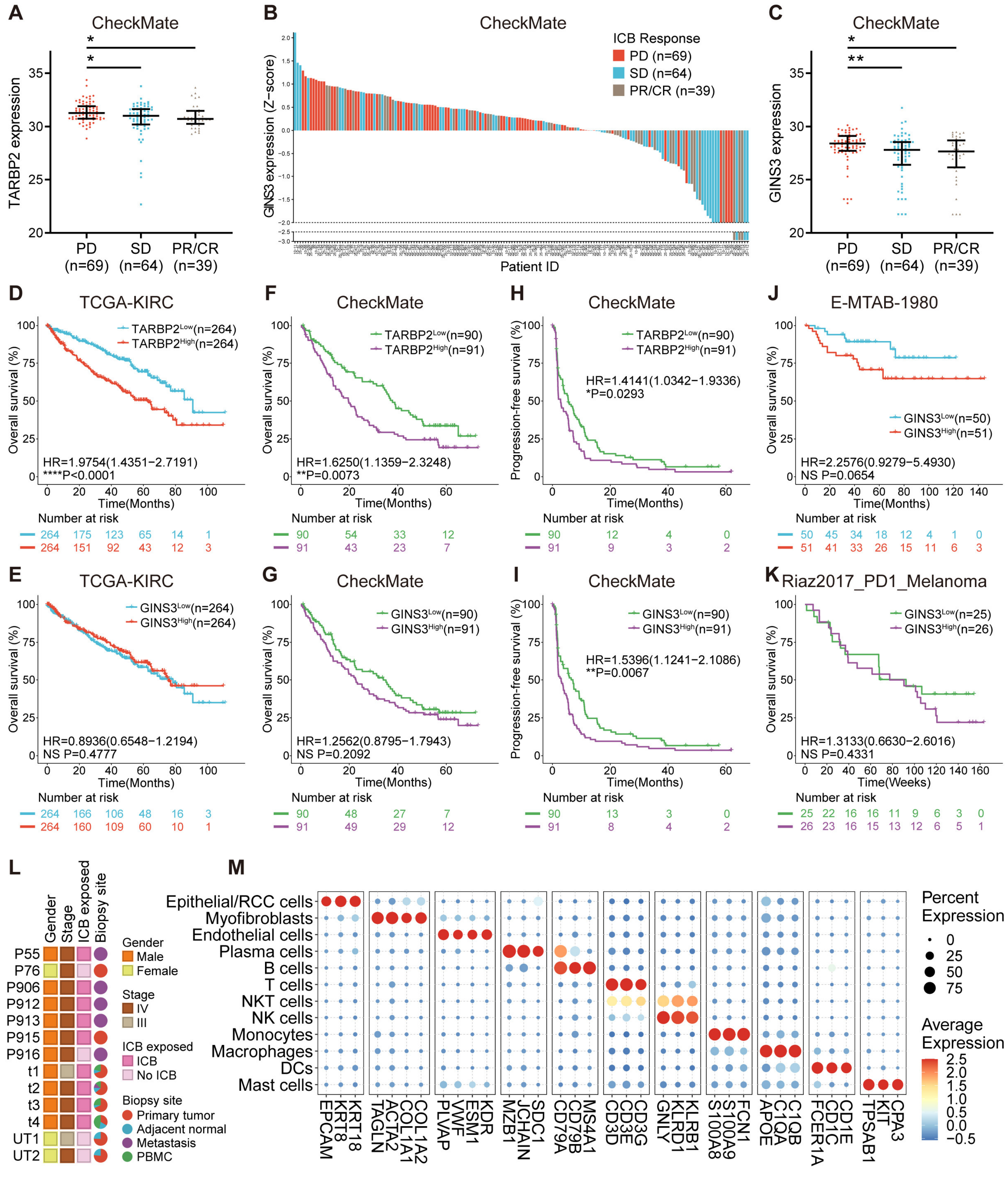

Supplemental Figure 2

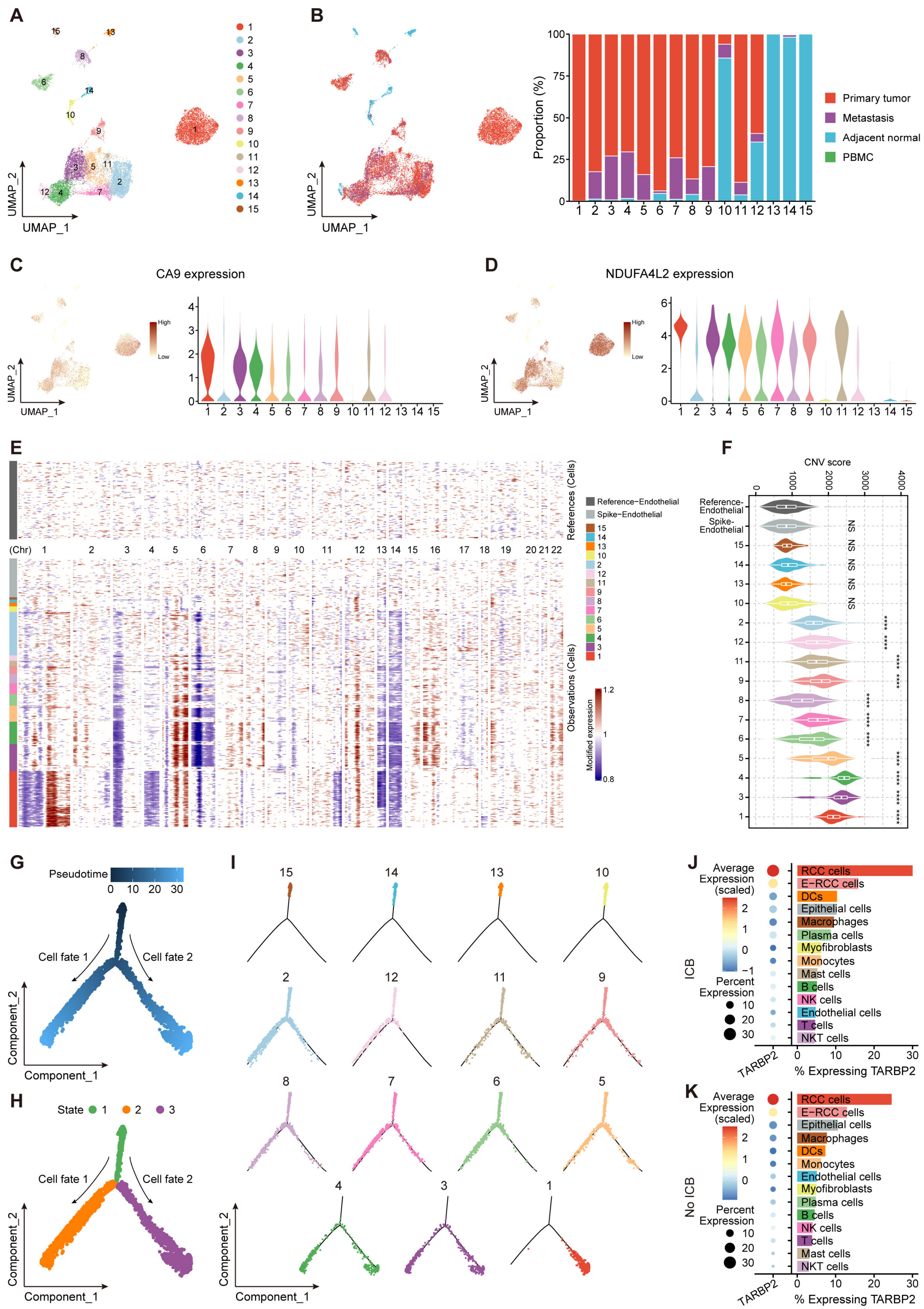

Supplemental Figure 3

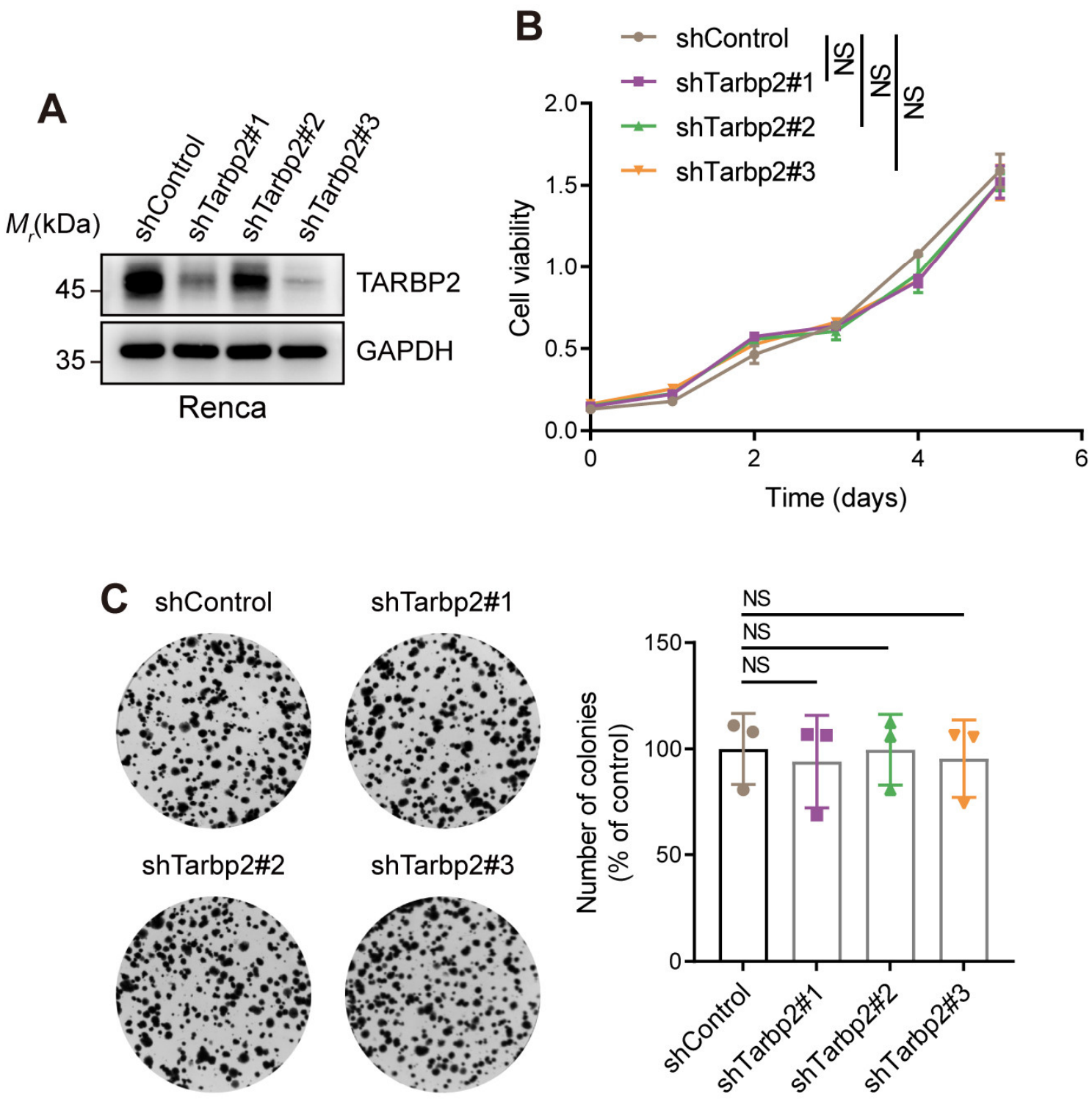

Supplemental Figure 4

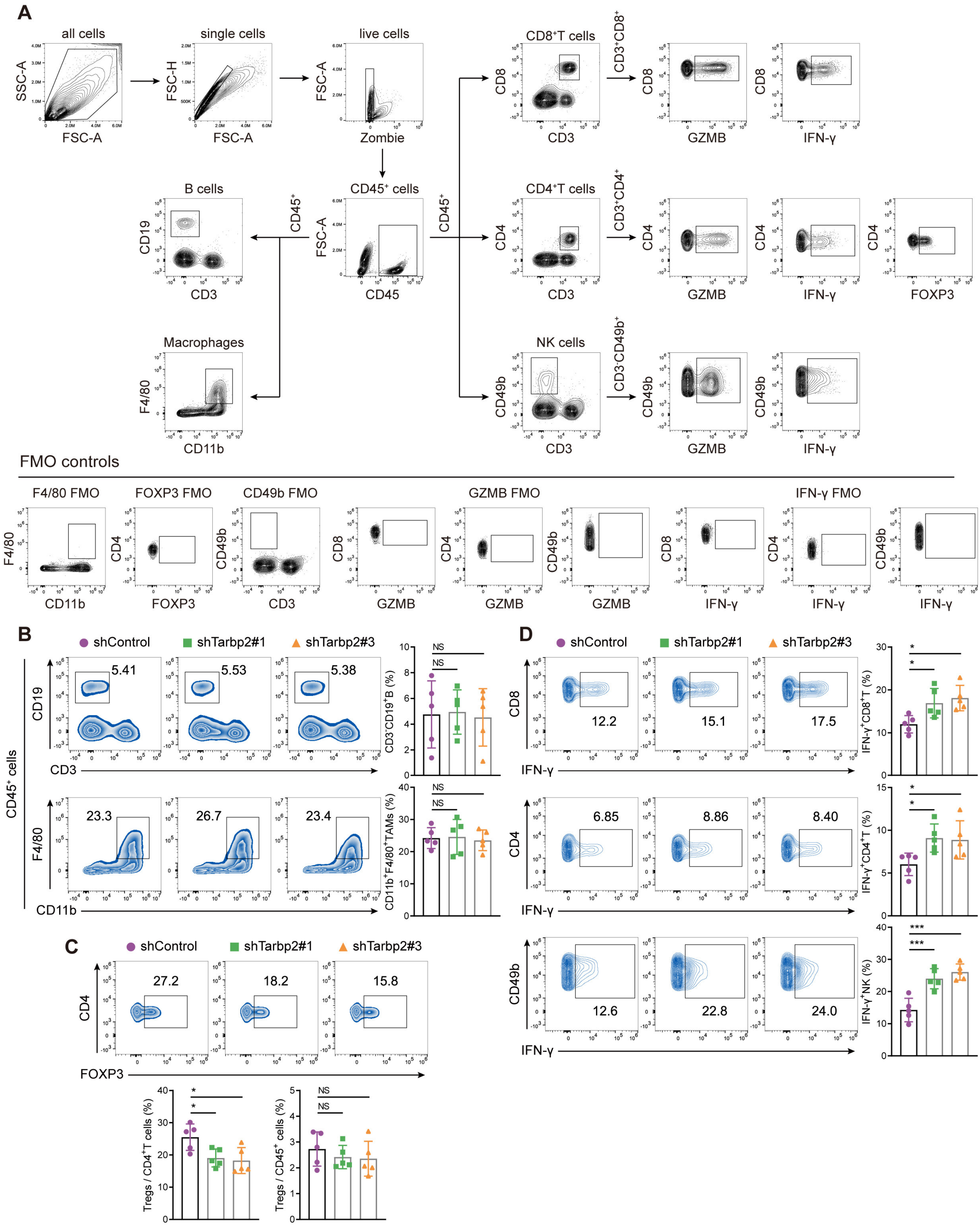

Supplemental Figure 5

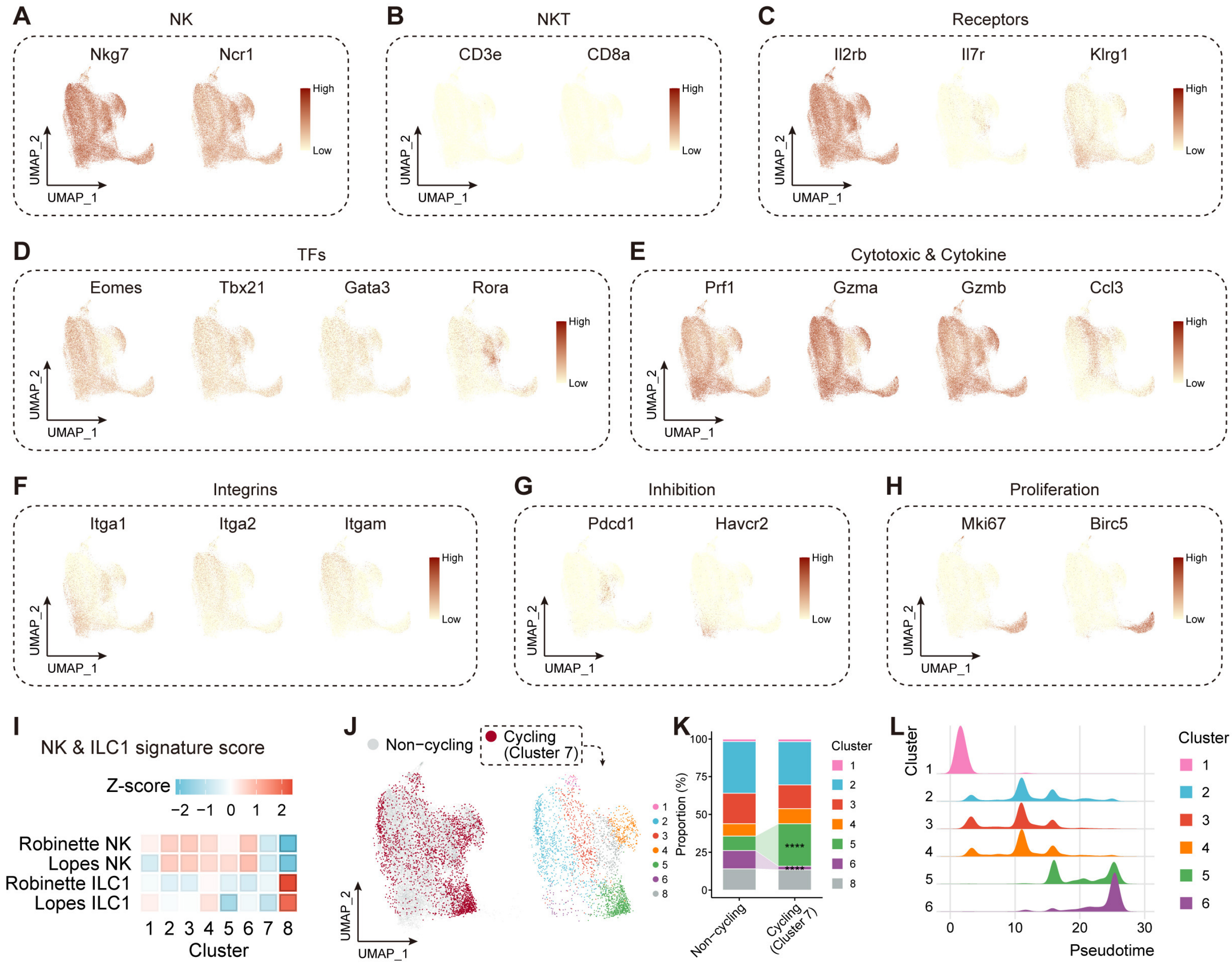

Supplemental Figure 6

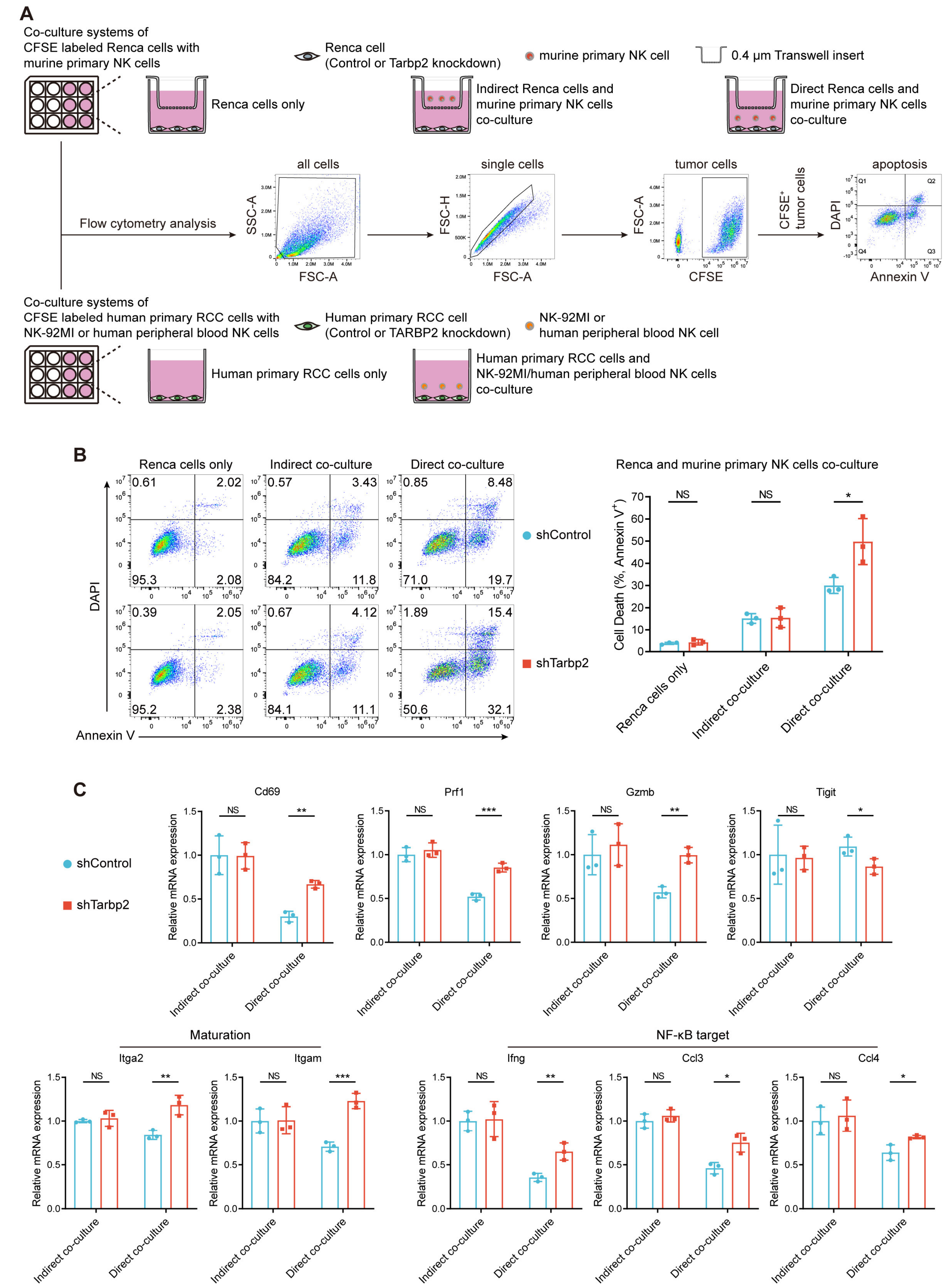

Supplemental Figure 7

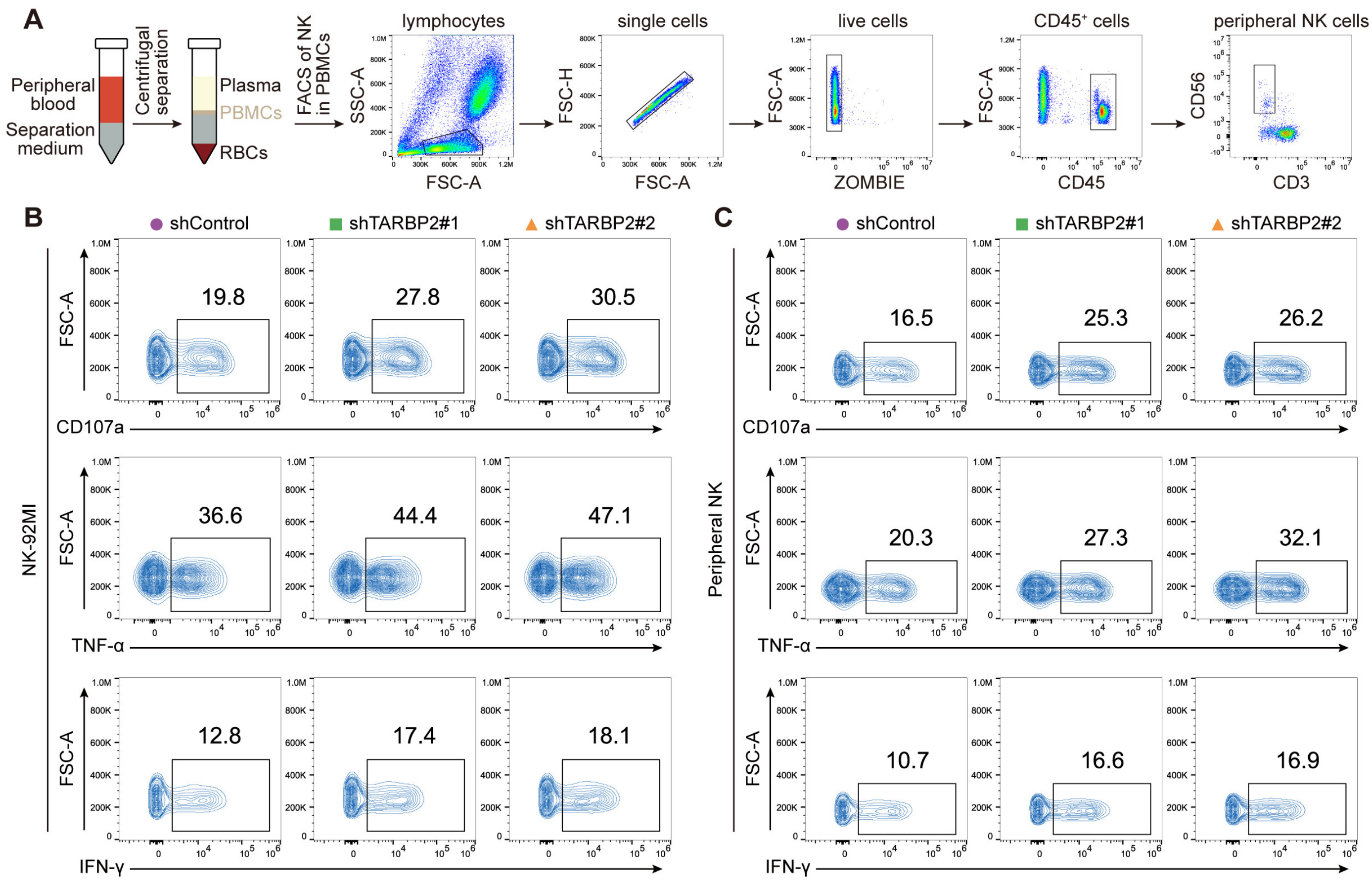

Supplemental Figure 8

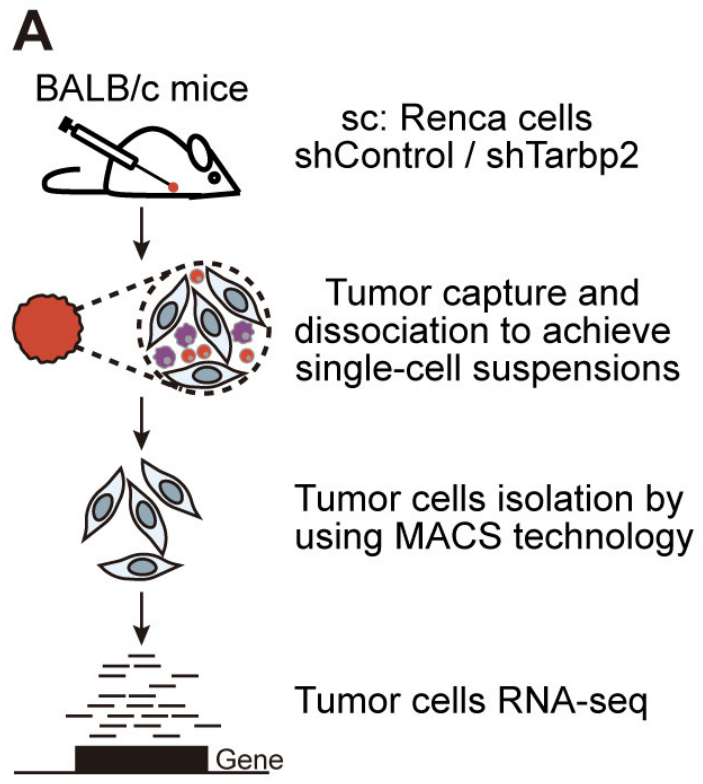

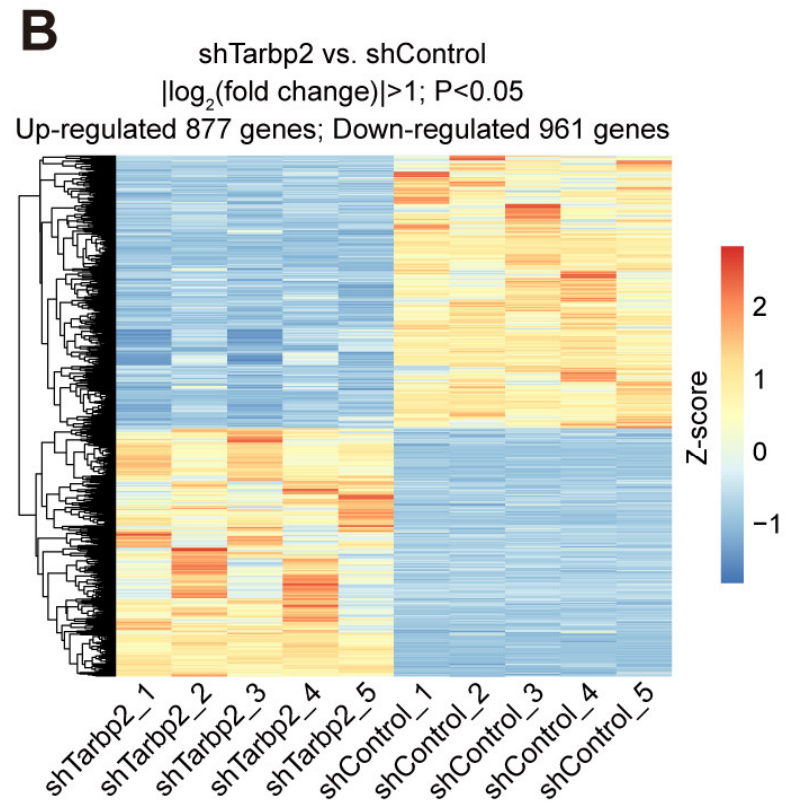

C

877 genes up-regulated in shTarbp2 → 68 genes in GO:0009986 GOCC_CELL_SURFACE → 8 genes down-regulated in FFPE samples from ICB Non-Responders

shTarbp2 vs. shControl			ICB Non-Responders vs. Responders		
mouse gene	$\log_2$(fold change)	P	human gene	$\log_2$(fold change)	P
Lifr	3.7995	<0.0001	LIFR	-1.0741	0.0181
Csf1r	2.6626	<0.0001	CSF1R	-5.0272	0.0167
Adamts9	2.0606	<0.0001	ADAMTS9	-1.1635	0.0166
Trpv4	1.3682	<0.0001	TRPV4	-6.3951	0.0032
Fzd4	1.3307	<0.0001	FZD4	-1.2328	0.0141
Ctsk	1.3193	<0.0001	CTSK	-1.5250	0.0017
Adgrg6	1.2899	<0.0001	ADGRG6	-2.9610	<0.0001
Lmo7	1.1406	<0.0001	LMO7	-1.0357	0.0051

D

961 genes down-regulated in shTarbp2 → 62 genes in GO:0009986 GOCC_CELL_SURFACE → 7 genes up-regulated in FFPE samples from ICB Non-Responders

shTarbp2 vs. shControl			ICB Non-Responders vs. Responders		
mouse gene	$\log_2$(fold change)	P	human gene	$\log_2$(fold change)	P
Cd276 (B7h3)	-3.0206	<0.0001	CD276 (B7-H3)	1.6148	<0.0001
Adam8	-2.2319	<0.0001	ADAM8	1.7343	0.0142
Aoc3	-1.9933	<0.0001	AOC3	2.3340	0.0002
Pxdn	-1.5032	0.0004	PXDN	6.3539	<0.0001
Krt18	-1.3727	<0.0001	KRT18	1.1826	0.0007
Slc7a11	-1.2547	<0.0001	SLC7A11	1.8578	0.0332
Fcrlb	-1.0443	0.0118	FCRLB	1.5941	0.0126

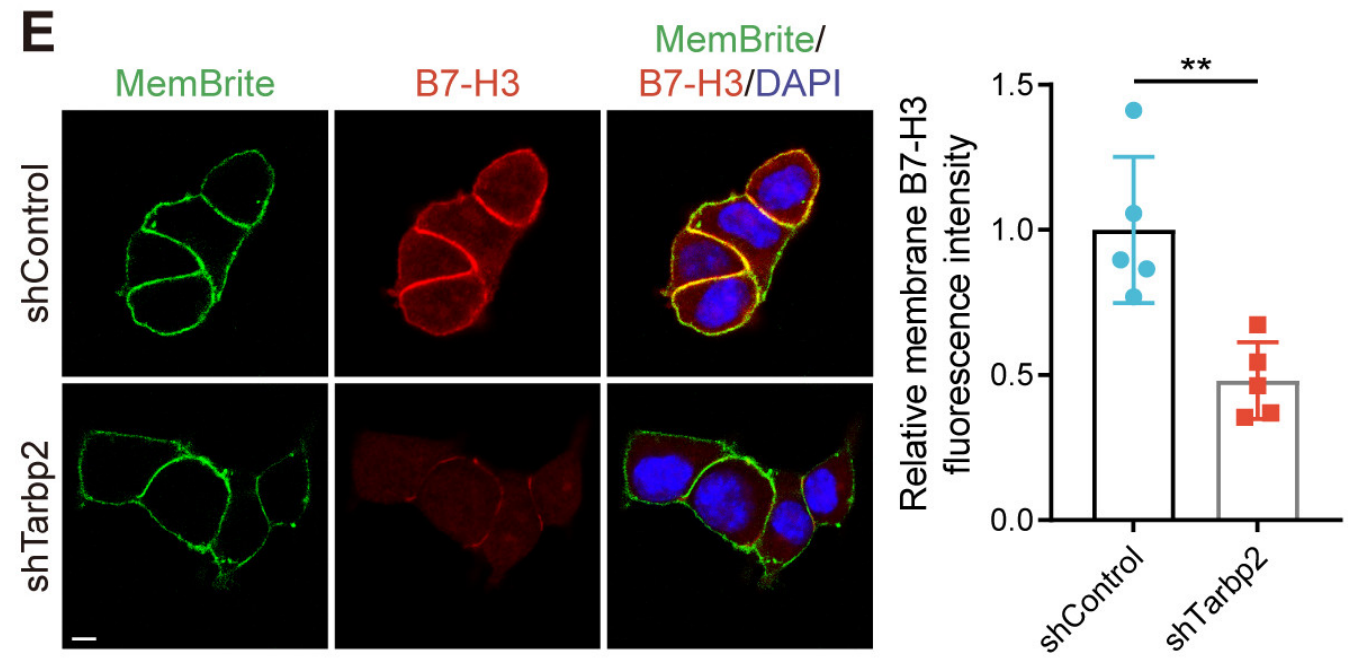

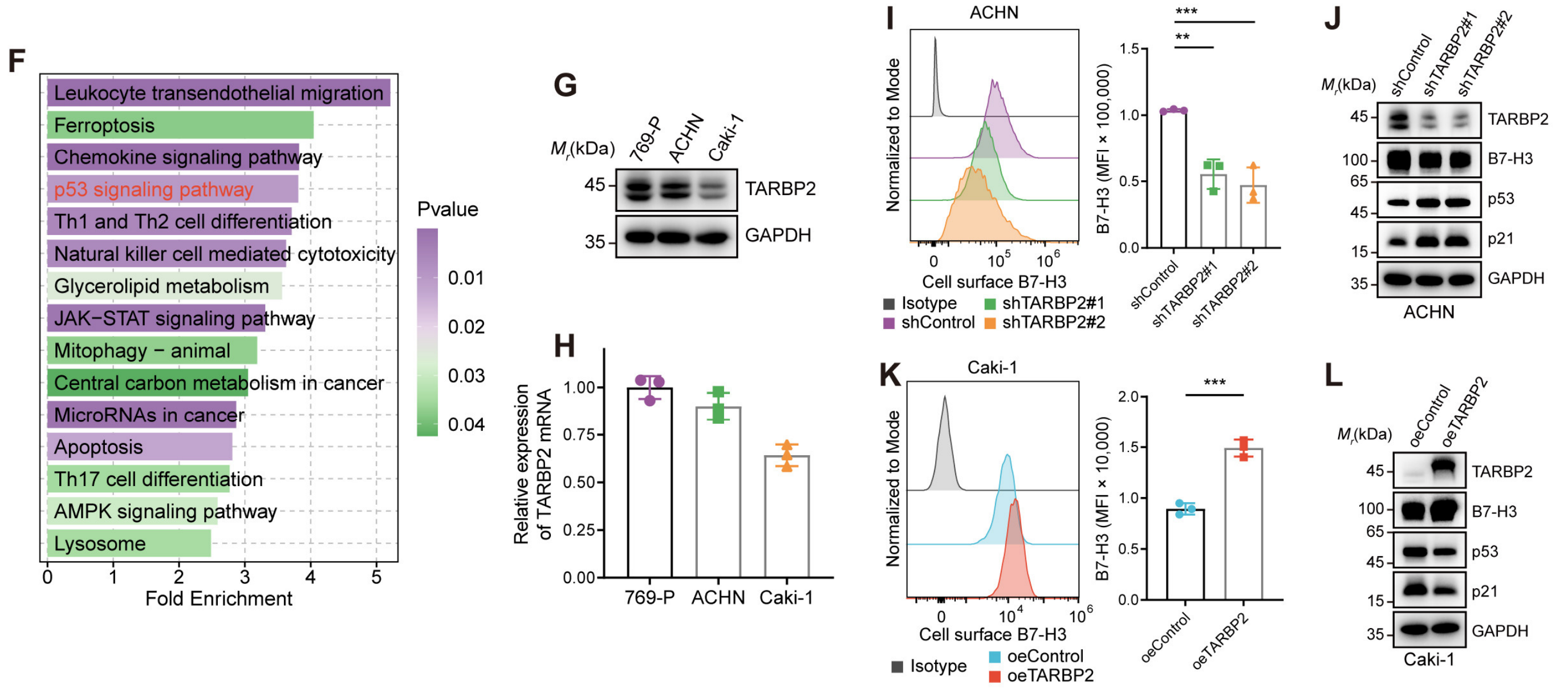

Supplemental Figure 9

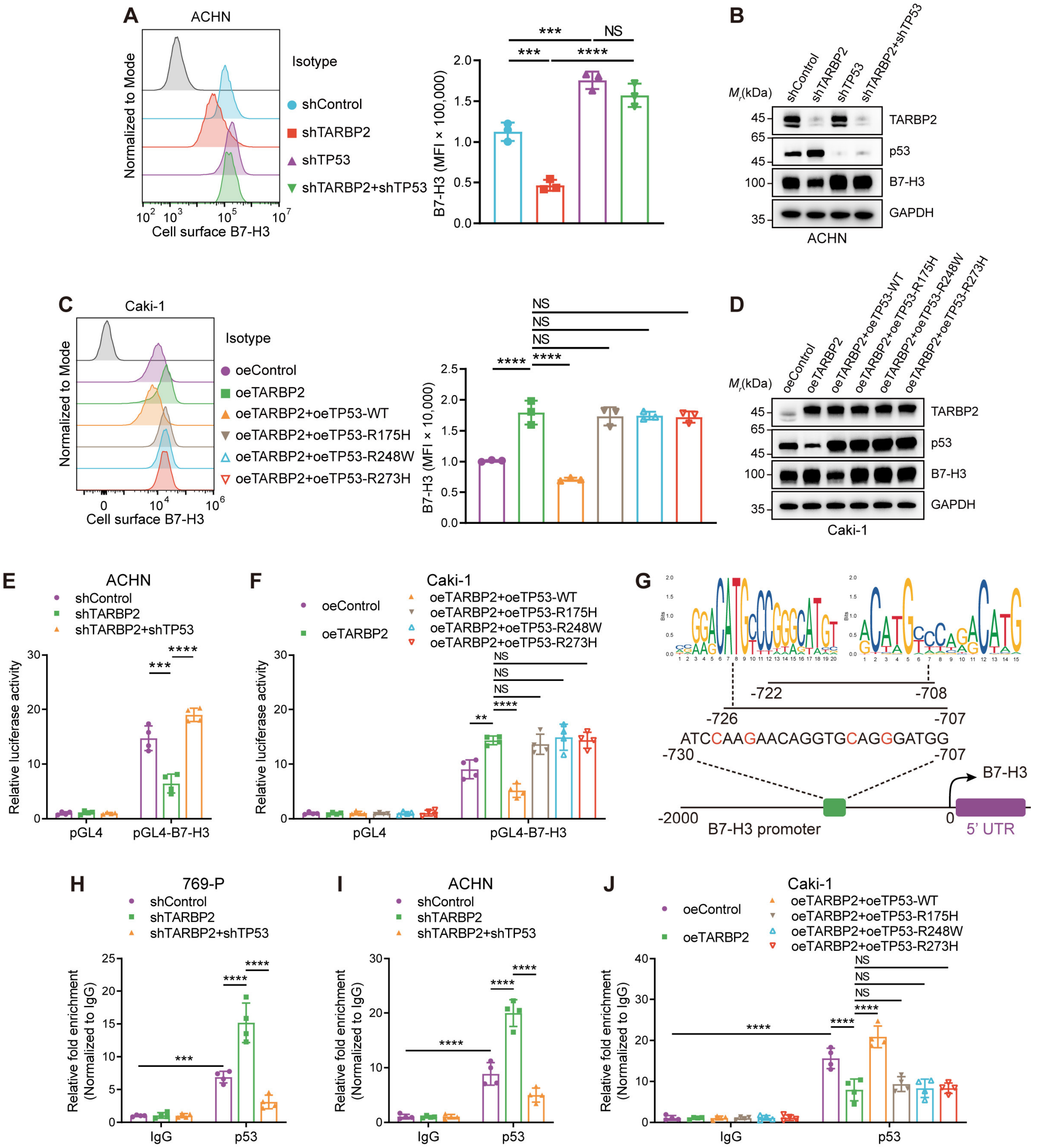

Supplemental Figure 10

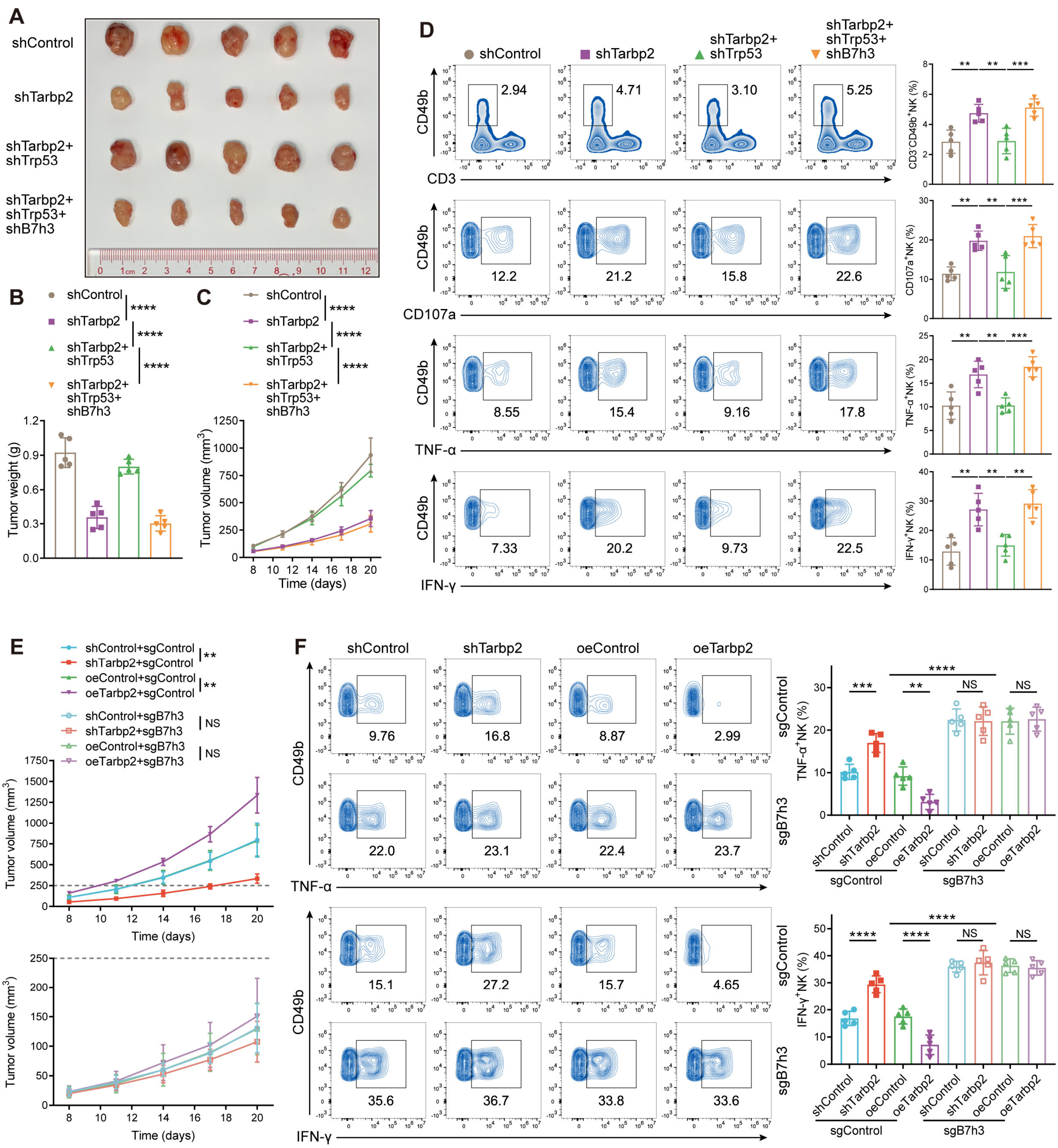

Supplemental Figure 11

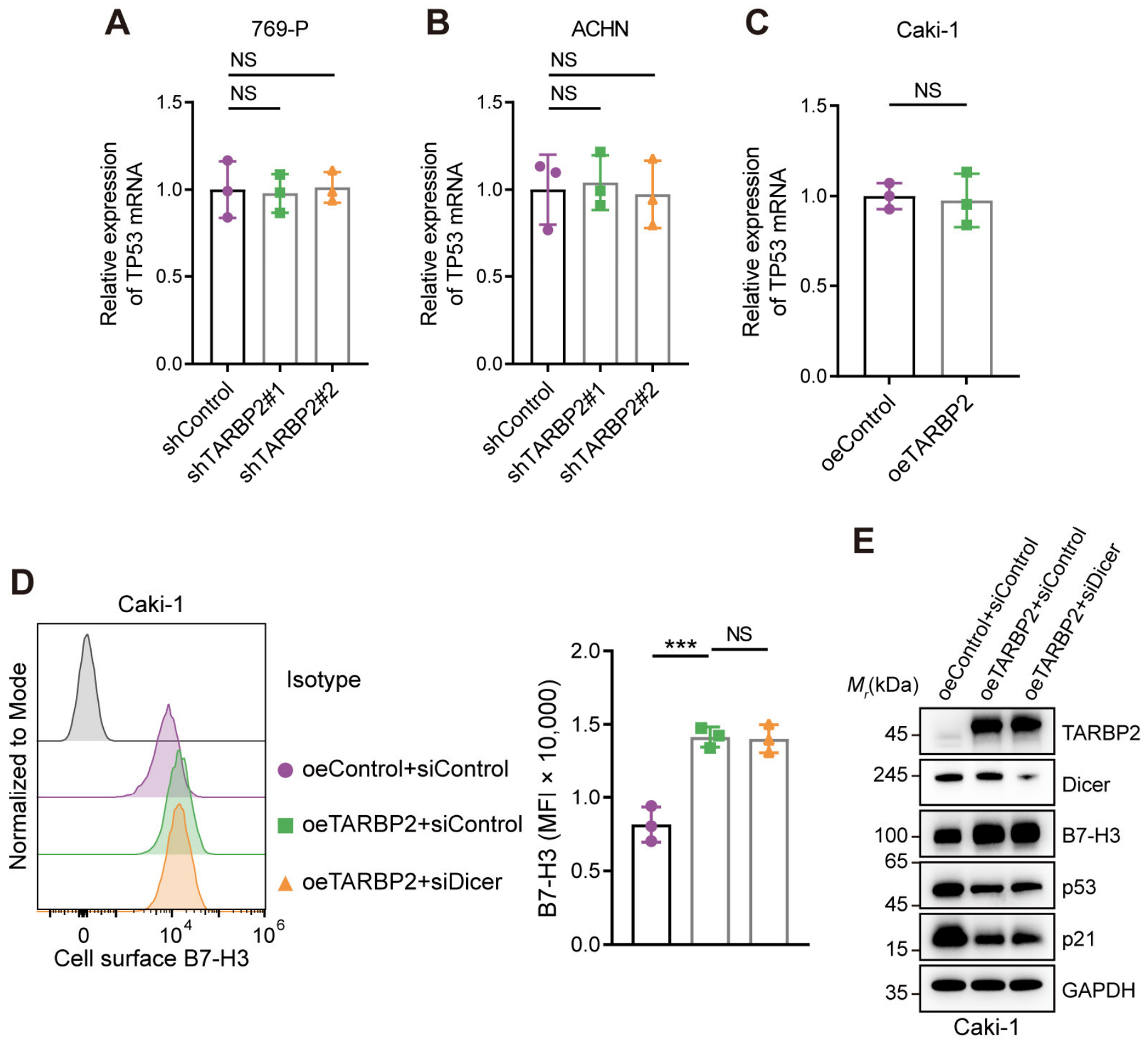

Supplemental Figure 12

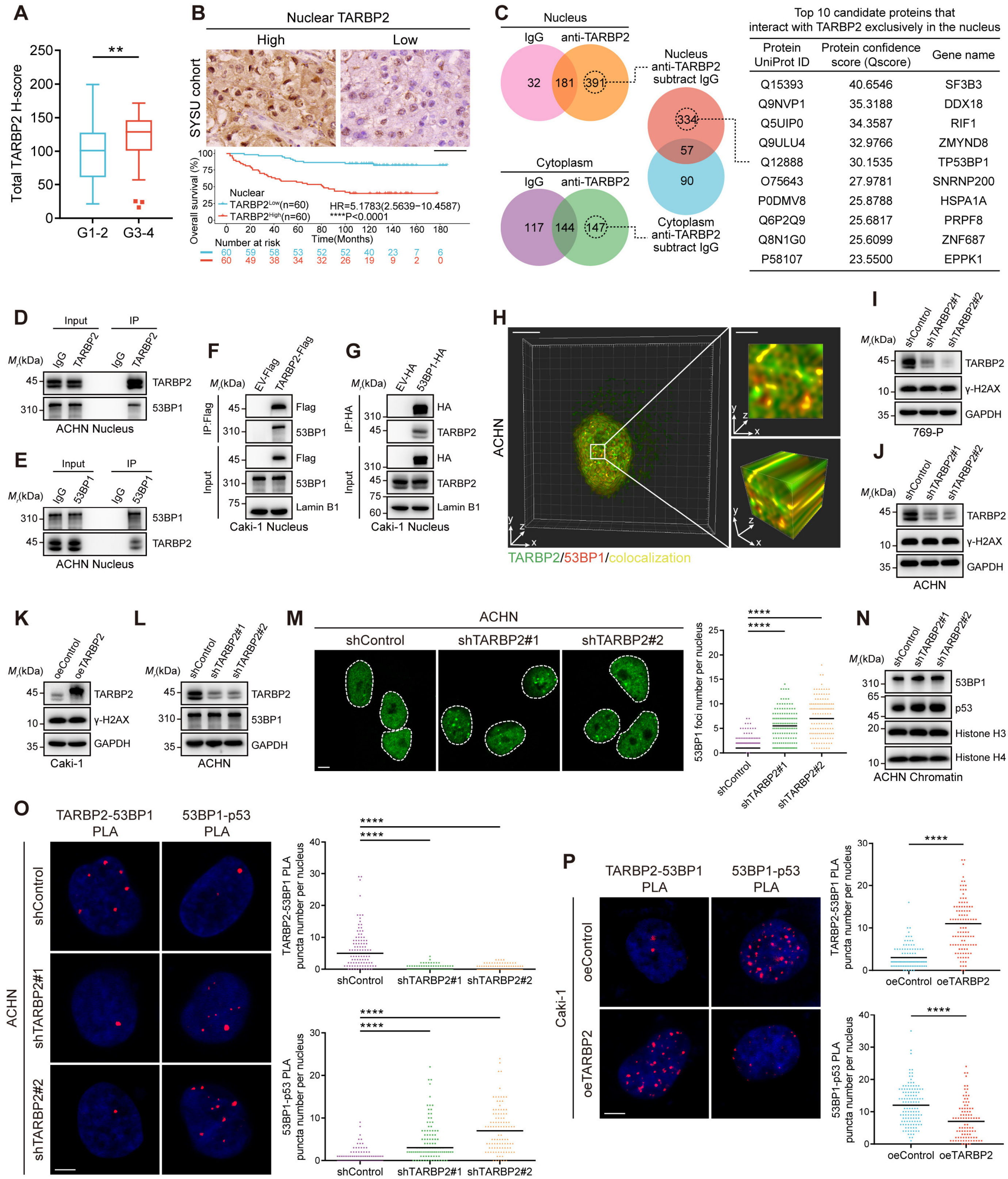

Protein UniProt ID	Protein confidence score (Qscore)	Gene name
Q15393	40.6546	SF3B3
Q9NVP1	35.3188	DDX18
Q5UIP0	34.3587	RIF1
Q9ULU4	32.9766	ZMYND8
Q12888	30.1535	TP53BP1
O75643	27.9781	SNRNP200
P0DMV8	25.8788	HSPA1A
Q6P2Q9	25.6817	PRPF8
Q8N1G0	25.6099	ZNF687
P58107	23.5500	EPPK1

Supplemental Figure 13

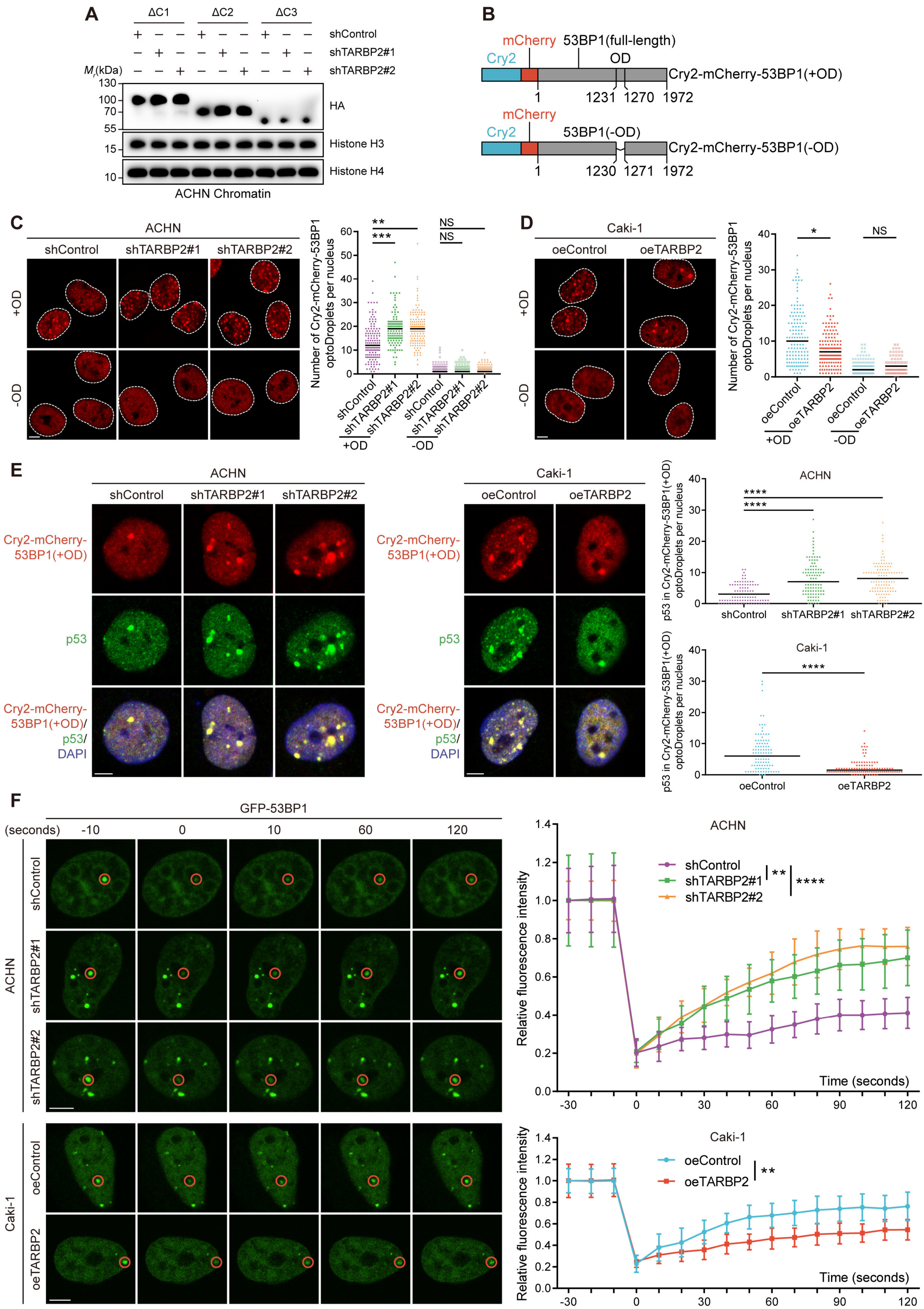

Supplemental Figure 14

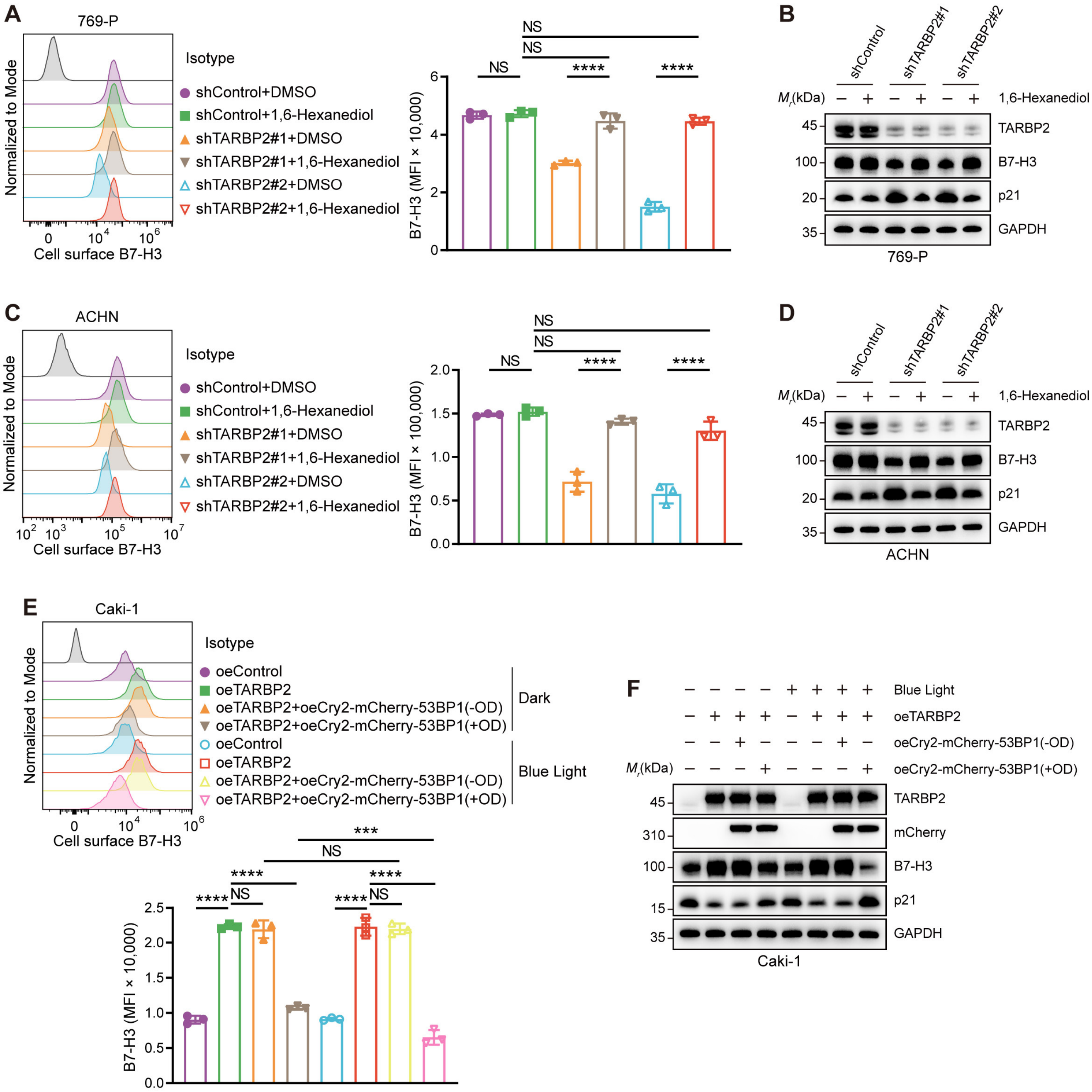

Supplemental Figure 15

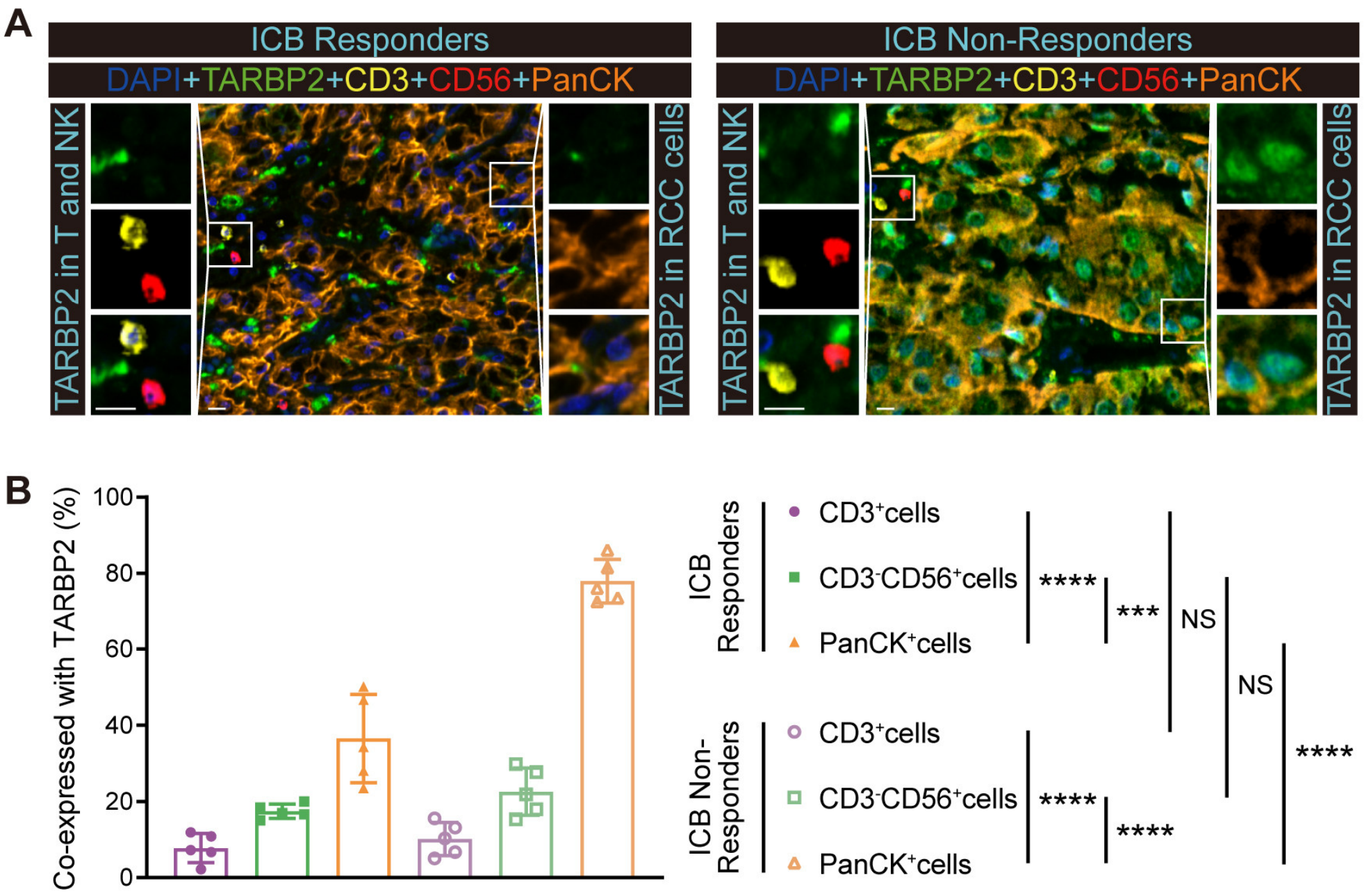

Supplemental Figure 16

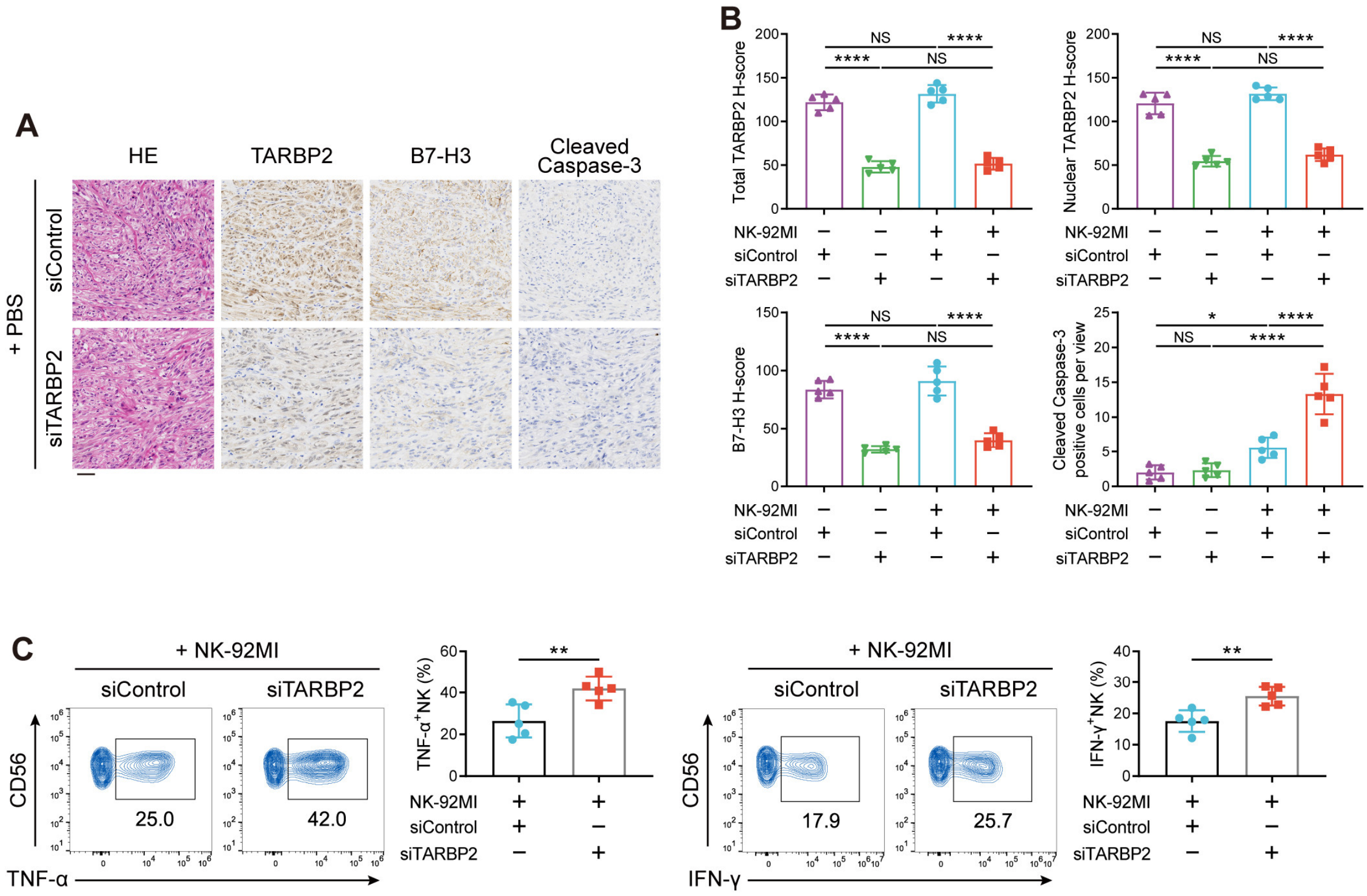

Supplemental Figure 17

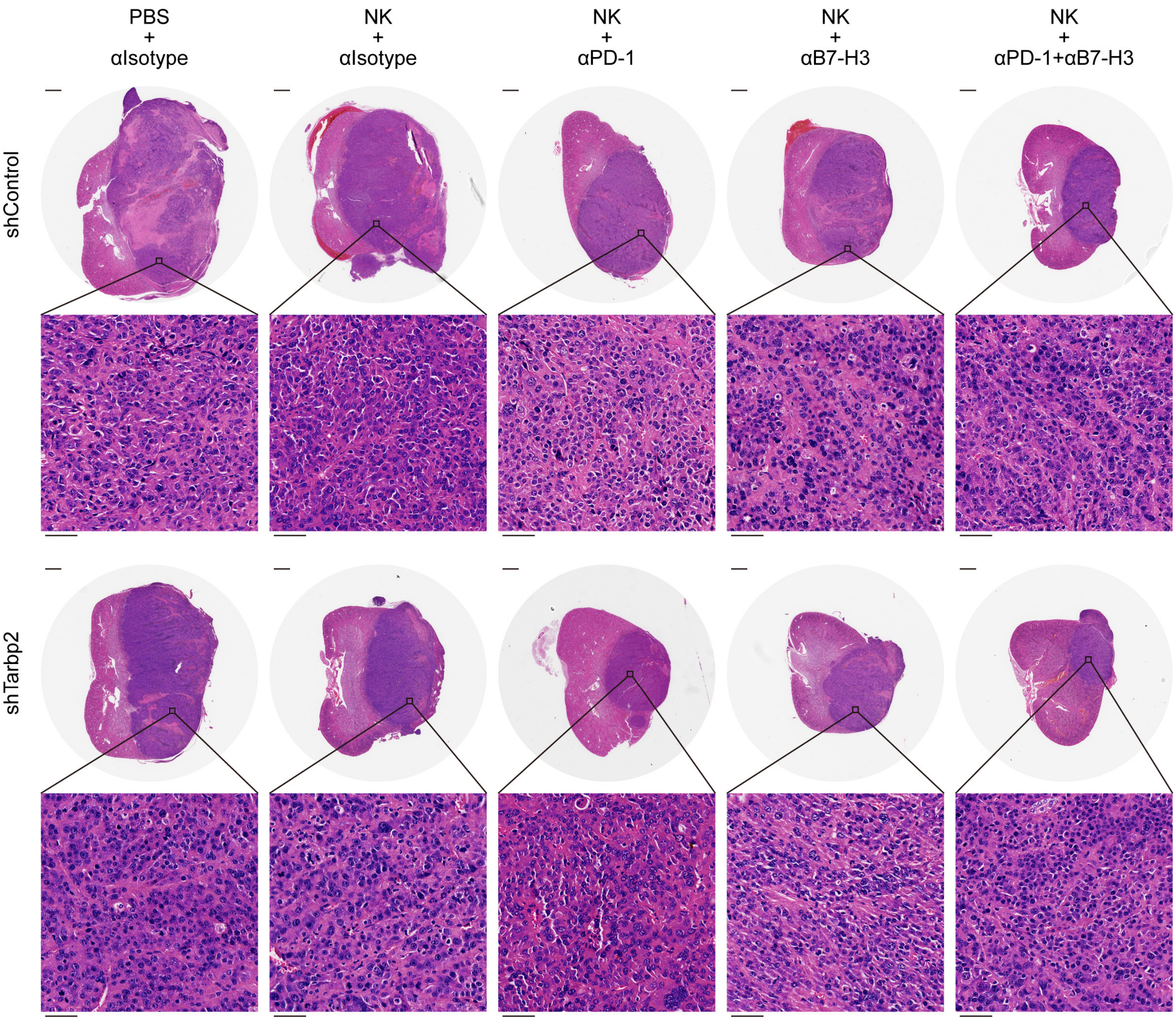

Supplemental Figure 18

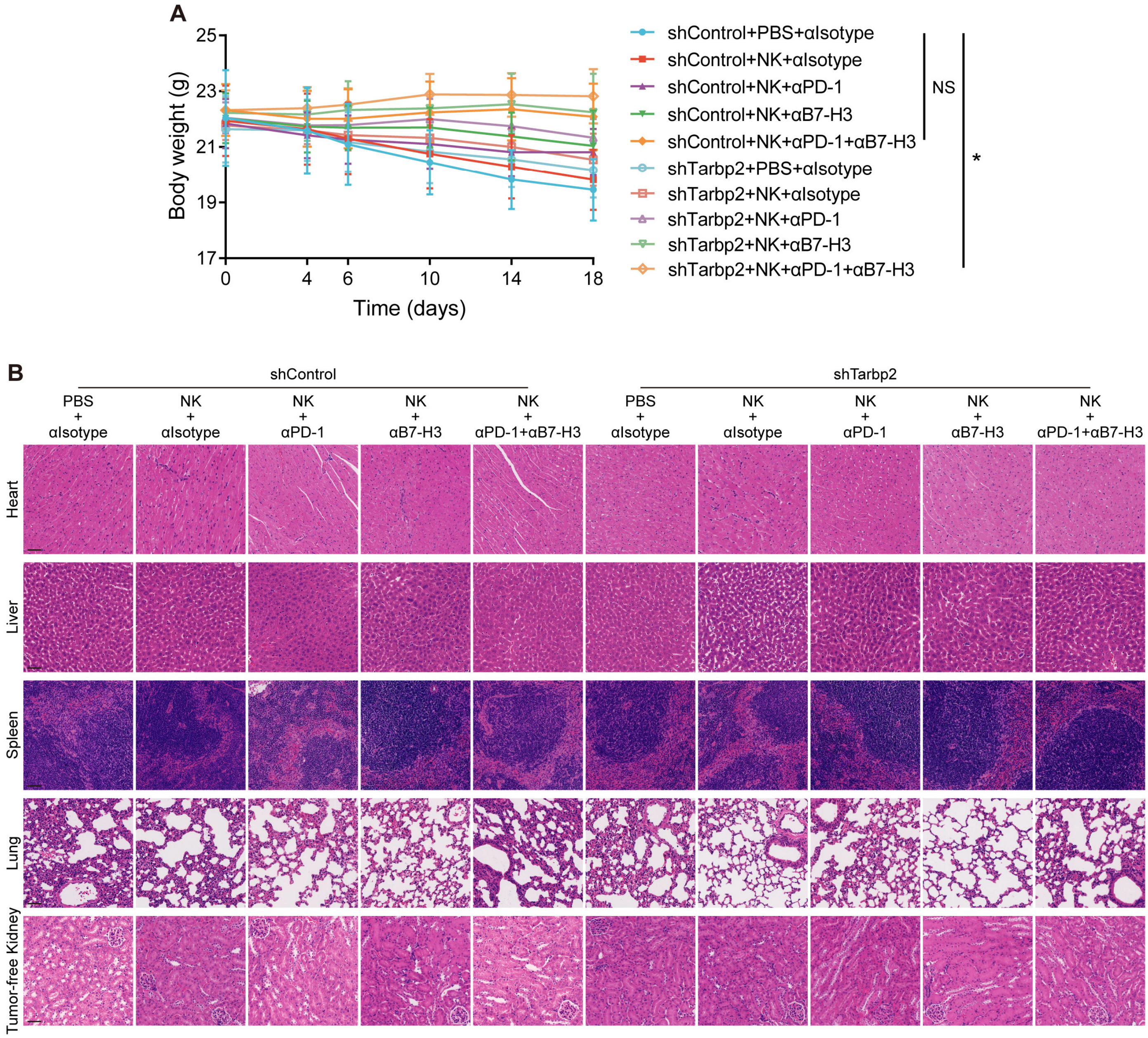

Supplemental Figure 19

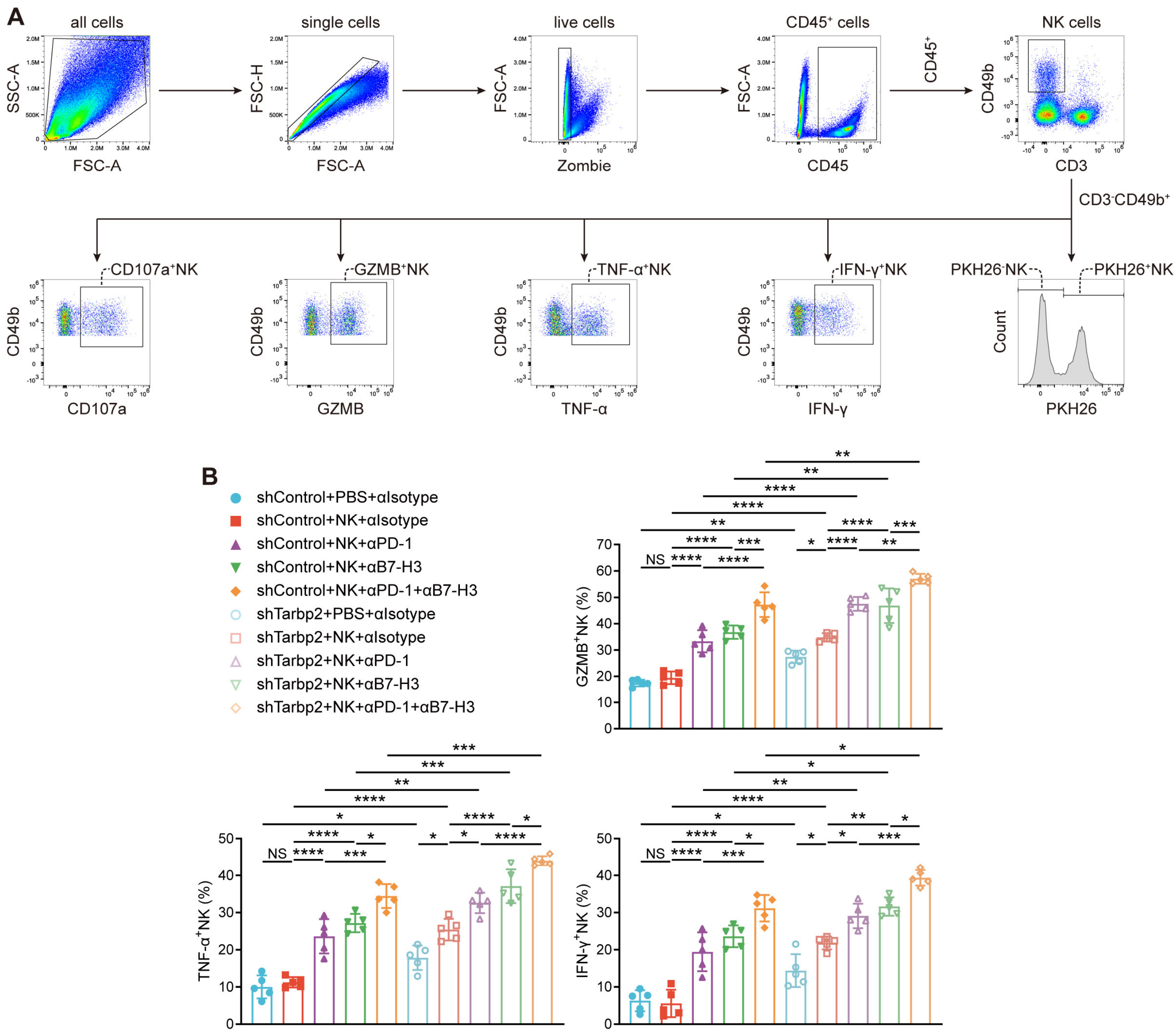

Figure Legends

Supplemental Figure 1. Data mining identifies TARBP2 as a predictor of poor prognosis and ICB resistance in RCC. (**A**) *TARBP2* expression in progressive disease (PD), stable disease (SD), partial response (PR), and complete response (CR) samples from the CheckMate cohort (n=172 samples). (**B**) waterfall plot of *GINS3* expression (Z-score) across PD, SD, and PR/CR samples in the CheckMate cohort (n=172 samples). (**C**) *GINS3* expression in PD, SD, and PR/CR samples from the CheckMate cohort (n=172 samples). (**D** and **E**) Overall survival (OS) of RCC patients with low (n=264) or high (n=264) *TARBP2* (**D**) or *GINS3* (**E**) expression in the TCGA-KIRC cohort. (**F** and **G**) OS of RCC patients with low (n=90) or high (n=91) *TARBP2* (**F**) or *GINS3* (**G**) expression in the CheckMate cohort. (**H** and **I**) Progression-free survival (PFS) of RCC patients with low (n=90) or high (n=91) *TARBP2* (**H**) or *GINS3* (**I**) expression in the CheckMate cohort. (**J**) OS of RCC patients with low (n=50) or high (n=51) *GINS3* expression in the E-MTAB-1980 cohort. (**K**) OS of melanoma patients with low (n=25) or high (n=26) *GINS3* expression in the Riaz2017_PD1_Melanoma cohort. (**L**) Overview of 13 advanced RCC patients included in integrated single-cell RNA sequencing (scRNA-seq) analysis. (**M**) Dot plots showing expression of selected marker genes for cell type annotation. Data are presented as scatter dot plots with error bars indicating the median and interquartile range in **A** and **C**. Statistics were acquired by Kruskal-Wallis test followed by Dunn's multiple comparisons in **A** and **C**, by log-rank test in **D**-**K**. ****, $P<0.0001$; **, $P<0.01$; *, $P<0.05$; NS, not significant. HR, hazard ratio.

Supplemental Figure 2. TARBP2 is highly expressed in malignant RCC cells. (**A**) UMAP plot showing subpopulation clustering of Epithelial/RCC cells. (**B**) UMAP plot (left) and stacked bar chart (right) showing biopsy site composition across Epithelial/RCC subpopulations. (**C** and **D**) Expression of canonical markers *CA9* (**C**) and *NDUFA4L2* (**D**) in Epithelial/RCC subpopulations. (**E**) Heatmap showing large-scale copy number variation (CNV) profiles across Epithelial/RCC subpopulations. Red and blue indicate CNV amplification and deletion, respectively, with color intensity representing magnitude of CNV variation. Endothelial cells served as reference cells. (**F**) Violin plot illustrating CNV levels in endothelial cells and Epithelial/RCC subpopulations. Statistical significance was determined by Kruskal-Wallis test followed by Dunn's multiple comparisons for pairwise comparisons against the Endothelial-Reference group. ****, $P<0.0001$; NS, not significant. (**G**) Pseudotime trajectory analysis with colors from dark to light indicating pseudotime progression. (**H**) Differentiation trajectories showing cell states. (**I**) Distribution of Epithelial/RCC subpopulations along differentiation trajectories. (**J** and **K**) *TARBP2* expression levels and proportions of *TARBP2*-positive cells across cell types in samples from ICB-treated (**J**) or ICB-untreated (**K**) patients.

Supplemental Figure 3. TARBP2 is dispensable for RCC cell proliferation in vitro. (**A**) Western blot analysis of TARBP2 protein levels in Renca cells with control or *Tarbp2* knockdown. GAPDH served as a loading control. (**B**) Cell counting kit-8 (CCK-8) assay assessing the proliferative capacity of Renca cells with control or *Tarbp2* knockdown (n=3 independent experiments). (**C**) Representative images (left) and quantification (right) of colony formation assay evaluating the proliferative capacity of Renca cells with control or *Tarbp2* knockdown (n=3 independent experiments). All data with error bars are presented as mean ± SD. Statistics were acquired by two-way ANOVA followed by Dunnett's multiple comparisons in **B**, by Kruskal-Wallis test followed by Dunn's multiple comparisons in **C**. NS, not significant.

Supplemental Figure 4. TARBP2 reshapes the tumor immune microenvironment in RCC. (**A**) Gating strategy for profiling the tumor immune microenvironment of Renca-derived subcutaneous tumors in BALB/c mice. Fluorescence minus one (FMO) controls are fully stained samples in which the antibody for the marker of interest is omitted. (**B**) Proportions of $CD3^-CD19^+$ B cells and $CD11b^+F4/80^+$ tumor-associated macrophages (TAMs) among $CD45^+$ cells from the indicated Renca-derived tumors in BALB/c mice (n=5 mice per group). (**C**) Proportions of $CD4^+FOXP3^+$ regulatory T cells (Tregs) within $CD4^+$ T cells or $CD45^+$ cells from the indicated Renca-derived tumors in BALB/c mice (n=5 mice per group). (**D**) Proportions of IFN-γ^+ cells within $CD8^+$ T cells, $CD4^+$ T cells, and $CD3^-CD49b^+$ natural killer (NK) cells from the indicated Renca-derived tumors in BALB/c mice (n=5 mice per group). All data with error bars are presented as mean ± SD. Statistics were acquired by one-way ANOVA followed by Dunnett's multiple comparisons test for comparisons with the control group in **B**-**D**. ***, $P<0.001$; *, $P<0.05$; NS, not significant.

Supplemental Figure 5. Transcriptomic profiles of $CD3^-NKp46^+$ cell clusters in scRNA-seq analysis. (**A**-**H**) UMAP visualization of key gene expression patterns in $CD3^-NKp46^+$ cell clusters, including genes annotated as NK cell markers (**A**), natural killer T (NKT) cell markers (**B**), surface receptors (**C**), transcription factors (TFs) (**D**), cytotoxic molecules and cytokines (**E**), integrins (**F**), inhibitory molecules (**G**), and proliferation markers (**H**). (**I**) Enrichment scores of NK and type 1 innate lymphoid cell (ILC1) signatures across cell clusters. (**J**) UMAP plots showing the distribution of cycling cells across all non-cycling NK/ILC clusters after re-integration and label transfer of cycling cells with cell cycle gene regression. (**K**) Proportions of cluster labels from non-cycling NK/ILC cells assigned to cycling cells. (**L**) Distribution of NK cell clusters (clusters 1-6) along pseudotime. Statistics were acquired by Chi-square test in **K**. ****, $P<0.0001$.

Supplemental Figure 6. TARBP2 regulates NK cell function in a contact-dependent manner between RCC and NK cells. (**A**) Schematic of the different co-culture systems and the gating strategy for assessing apoptosis in CFSE-labeled RCC cells. (**B**) Representative flow cytometry plots (left) and quantification (right) of apoptosis in CFSE-labeled Renca cells in the co-culture systems. Renca cells were co-cultured with murine primary NK cells at a 1:1 ratio or cultured alone for 8 h (n=3 independent experiments). (**C**) RT-qPCR analysis of transcriptional changes in murine primary NK cells in the co-culture systems. Renca cells were co-cultured with murine primary NK cells at a 1:1 ratio for 8 h. Relative mRNA levels were normalized to those of the control group under indirect co-culture conditions (n=3 independent experiments). All data with error bars are presented as mean ± SD. Statistics were acquired by one-way ANOVA followed by Tukey's multiple comparisons in **B** and **C**. ***, *P*<0.001; **, *P*<0.01; *, *P*<0.05; NS, not significant.

Supplemental Figure 7. TARBP2 inhibits the effector function of human NK cells in vitro. (**A**) Schematic of primary NK cell isolation from peripheral blood. (**B** and **C**) Representative flow cytometry plots showing $CD107a^+$, TNF-α^+, and IFN-γ^+ cells in NK-92MI cells (**B**) or peripheral NK cells (**C**) after incubation with *TARBP2*-knockdown or control primary RCC cells (E:T=1:1, 8h). RBCs, red blood cells.

Supplemental Figure 8. TARBP2 suppresses p53 signaling and upregulates B7-H3. (**A**) Schematic illustration of tumor cell isolation from Renca-derived tumors in BALB/c mice followed by RNA sequencing (RNA-seq) analysis. (**B**) Heatmap showing differentially expressed genes between *Tarbp2*-knockdown and control groups (n=5 mice per group). (**C**) Identification of cell surface protein-coding genes upregulated following *Tarbp2* knockdown and whose upregulation is potentially associated with improved ICB response. (**D**) Identification of cell surface protein-coding genes downregulated following *Tarbp2* knockdown and whose downregulation is potentially associated with improved ICB response. (**E**) Left: Representative images of plasma membrane-labeled tumor cells isolated from control or *Tarbp2*-knockdown Renca-derived tumors in BALB/c mice. Plasma membranes were labeled with MemBrite Fix Dye (green) prior to immunofluorescence staining for B7-H3 (red). Nuclei were counterstained with DAPI (blue). Scale bar, 5 μm. Right: Quantification of plasma membrane-associated B7-H3 fluorescence intensity. One hundred cells were randomly selected from each mouse, and fluorescence intensities were averaged to generate one biological replicate (n=5 mice per group). Relative fluorescence intensity was normalized to the mean value of the control group. (**F**) KEGG analysis showing significantly altered signaling pathways between *Tarbp2*-knockdown and control groups (n=5 mice per group). (**G**) Western blot of TARBP2 protein expression in RCC cell lines. GAPDH served as a loading control. (**H**) *TARBP2* mRNA levels in RCC cell lines (n=3 independent experiments). Relative mRNA levels were normalized to levels in 769-P cells. (**I**) Flow cytometry analysis of cell-

surface B7-H3 levels in *TARBP2*-knockdown and control ACHN cells (n=3 independent experiments). (**J**) Western blot analysis of total B7-H3, p53, and p21 protein levels in *TARBP2*-knockdown and control ACHN cells. GAPDH served as a loading control. (**K**) Flow cytometry analysis of cell-surface B7-H3 levels in *TARBP2*-overexpressing and control Caki-1 cells (n=3 independent experiments). (**L**) Western blot analysis of total B7-H3, p53, and p21 protein levels in *TARBP2*-overexpressing and control Caki-1 cells. GAPDH served as a loading control. All data with error bars are presented as mean ± SD. Statistics were acquired by unpaired two-tailed Student's t-test in **E** and **K**, by one-way ANOVA followed by Dunnett's multiple comparisons for pairwise comparisons against the control group in **I**. ***, *P*<0.001; **, *P*<0.01. sc, subcutaneous injection; MFI, median fluorescence intensity.

Supplemental Figure 9. TARBP2 attenuates p53-mediated transcriptional repression of *B7-H3*. (**A**) Flow cytometry analysis of cell-surface B7-H3 levels in *TP53*-knockdown ACHN cells with *TARBP2* knockdown or control (n=3 independent experiments). (**B**) Western blot analysis of total B7-H3 protein levels in *TP53*-knockdown ACHN cells with *TARBP2* knockdown or control. GAPDH served as a loading control. (**C**) Flow cytometry analysis showing the effect of wild-type or mutant *TP53* overexpression on cell-surface B7-H3 levels in *TARBP2*-overexpressing Caki-1 cells (n=3 independent experiments). (**D**) Western blot analysis showing the effect of wild-type or mutant *TP53* overexpression on total B7-H3 protein levels in *TARBP2*-overexpressing Caki-1 cells. GAPDH served as a loading control. (**E** and **F**) Dual-luciferase reporter assays evaluating vector or *B7-H3* promoter-driven reporter activity in ACHN cells (**E**) under control, *TARBP2* knockdown, or *TARBP2*/*TP53* co-knockdown conditions; and in Caki-1 cells (**F**) under control, *TARBP2* overexpression, *TARBP2*/wild-type *TP53* co-overexpression, or *TARBP2*/mutant *TP53* co-overexpression conditions (n=4 independent experiments). Relative luciferase activities were normalized to vector control samples. (**G**) Putative p53-binding sites within the human *B7-H3* promoter predicted using the JASPAR database. (**H**-**J**) ChIP-qPCR analysis of p53 enrichment at the *B7-H3* promoter in 769-P cells (**H**) and ACHN cells (**I**) under control, *TARBP2* knockdown, or *TARBP2*/*TP53* co-knockdown conditions; and in Caki-1 cells (**J**) under control, *TARBP2* overexpression, *TARBP2*/wild-type *TP53* co-overexpression, or *TARBP2*/mutant *TP53* co-overexpression conditions (n=4 independent experiments). Enrichment levels were normalized to IgG control. All data with error bars are presented as mean ± SD. Statistics were acquired by one-way ANOVA followed by Tukey's multiple comparisons in **A**, **C**, **E**, **F**, and **H**-**J**. ****, *P*<0.0001; ***, *P*<0.001; **, *P*<0.01; NS, not significant. UTR, untranslated region.

Supplemental Figure 10. TARBP2 suppresses NK cell anti-tumor activity via the p53/B7-H3 axis. (**A**-**C**) Tumor images (**A**), tumor weights (**B**), and tumor growth curves (**C**) of Renca-derived tumors in BALB/c mice from the indicated groups (n=5 mice per group). (**D**) Proportions of $CD3^-CD49b^+$ NK cells within $CD45^+$ cells, as well as

CD107a$^+$, TNF-α$^+$, and IFN-γ$^+$ NK cell subsets from indicated Renca-derived tumors in BALB/c mice (n=5 mice per group). (**E**) Tumor growth curves of Renca-derived tumors in BALB/c mice from the indicated groups (n=5 mice per group). (**F**) Proportions of TNF-α$^+$ and IFN-γ$^+$ cells within NK cells from indicated Renca-derived tumors in BALB/c mice (n=5 mice per group). All data with error bars are presented as mean ± SD. Statistics were acquired by one-way ANOVA followed by Tukey's multiple comparisons in **B** and **D**, by two-way ANOVA followed by Bonferroni multiple comparisons in **C** and **E**, by two-way ANOVA followed by Tukey's multiple comparisons in **F**. ****, P<0.0001; ***, P<0.001; **, P<0.01; NS, not significant.

Supplemental Figure 11. TARBP2 regulates the p53/B7-H3 axis independently of Dicer. (**A**-**C**) *TP53* mRNA levels in *TARBP2*-knockdown and control 769-P cells (**A**), *TARBP2*-knockdown and control ACHN cells (**B**), and *TARBP2*-overexpressing and control Caki-1 cells (**C**) (n=3 independent experiments). Relative mRNA levels were normalized to the corresponding control groups. (**D**) Flow cytometry analysis showing the effect of *Dicer* silencing on cell-surface B7-H3 levels in *TARBP2*-overexpressing Caki-1 cells (n=3 independent experiments). (**E**) Western blot analysis showing the effect of *Dicer* silencing on total B7-H3, p53, and p21 protein levels in *TARBP2*-overexpressing Caki-1 cells. GAPDH served as a loading control. All data with error bars are presented as mean ± SD. Statistics were acquired by one-way ANOVA followed by Dunnett's multiple comparisons test for comparisons with the control group in **A** and **B**, by unpaired two-tailed Student's t-test in **C**, by one-way ANOVA followed by Tukey's multiple comparisons in **D**. ***, P<0.001; NS, not significant.

Supplemental Figure 12. TARBP2 interacts with 53BP1 to disrupt 53BP1-p53 complex formation and chromatin accumulation. (**A**) Box and whisker plot showing H-score of total TARBP2 in low- and high-grade RCC tissues from the SYSU cohort (n=120 samples). Boxes represent the interquartile range (IQR), with the center line indicating the median. Whiskers extend to values within 1.5-fold of the IQR, and dots indicate outliers. (**B**) Top: Representative IHC images of RCC tissues with low or high nuclear TARBP2 H-score in the SYSU cohort. Scale bar, 50 μm. Bottom: OS of RCC patients with low (n=60) or high (n=60) nuclear TARBP2 H-score in the SYSU cohort. (**C**) Workflow for identifying candidate proteins that interact with TARBP2 exclusively in the nucleus. The top 10 candidates ranked by confidence score are shown. (**D** and **E**) Co-IP assays showing interaction between endogenous TARBP2 and 53BP1 in ACHN cells. (**F** and **G**) Co-IP assays showing interaction between endogenous 53BP1 and overexpressed TARBP2-Flag (**F**), or endogenous TARBP2 and overexpressed 53BP1-HA (**G**) in Caki-1 cells. (**H**) 3D-SIM rendering of ACHN cells stained to indicate TARBP2 (green) and 53BP1 (red) colocalization (yellow). Scale bars, 5 μm and 1 μm (insets). The x-y plane represents lateral cross-sectional views parallel to the substrate surface, and the z-axis indicates axial dimension perpendicular to the substrate surface.

(**I**-**K**) Immunoblot analysis of DNA damage marker γ-H2AX in 769-P cells (**I**), ACHN cells (**J**), and Caki-1 cells (**K**). GAPDH served as a loading control. (**L**) Immunoblot analysis of 53BP1 in whole cell lysates of *TARBP2*-knockdown and control ACHN cells. GAPDH served as a loading control. (**M**) Representative confocal images (left) and quantification of 53BP1 foci (right) in *TARBP2*-knockdown and control ACHN cells (n>120 nuclei per group). Dashed lines outline nuclei. Scale bar, 5 μm. (**N**) Immunoblot analysis of 53BP1 and p53 in chromatin fractions of *TARBP2*-knockdown and control ACHN cells. Histone H3 and H4 served as chromatin loading controls. (**O** and **P**) Representative images (left) and quantification (right) of endogenous TARBP2-53BP1 and 53BP1-p53 interactions in ACHN cells (**O**) or Caki-1 cells (**P**) with indicated *TARBP2* modifications (n>100 nuclei per group). Nuclei were counterstained with DAPI (blue). Positive signals are indicated as red dots. Scale bars, 5 μm. Solid lines indicate medians in **M** (right), **O** (right), and **P** (right). Statistics were acquired by unpaired two-tailed Student's t-test in **A**, by log-rank test in **B**, by Kruskal-Wallis test followed by Dunn's multiple comparisons in **M** and **O**, by Mann-Whitney test in **P**. ****, $P<0.0001$; **, $P<0.01$. EV, empty vector.

Supplemental Figure 13. TARBP2 disrupts oligomerization-driven phase separation of 53BP1. (**A**) Immunoblot analysis of 53BP1 truncation mutants in chromatin fractions from *TARBP2*-knockdown and control ACHN cells. Histone H3 and H4 served as chromatin loading controls. (**B**) Schematic diagram showing the structures of Cry2-mCherry-53BP1 fusion proteins with or without the oligomerization domain (OD). (**C** and **D**) Representative images (left) and quantification (right) of optoDroplet formation of Cry2-mCherry-53BP1 fusion proteins with or without the OD in ACHN cells (**C**) or Caki-1 cells (**D**) with indicated *TARBP2* modulation (n>120 nuclei per group). Dashed lines outline nuclei. Scale bars, 5 μm. (**E**) Cry2-mCherry-53BP1 (containing the OD) optoDroplet inducing followed by p53 (green) and DAPI (blue) staining in ACHN cells or Caki-1 cells with indicated *TARBP2* modulation (n>100 nuclei per group). Scale bars, 5 μm. (**F**) Representative images (left) and quantification (right) of fluorescence recovery after photobleaching (FRAP) analysis of GFP-53BP1 puncta in ACHN cells or Caki-1 cells with indicated *TARBP2* modulation (n=12 events from independent cells per group). Scale bars, 5 μm. Red circles indicate bleached puncta. Time zero (0 second) on the x-axis marks the execution of photobleaching. Solid lines indicate medians in **C** (right), **D** (right), and **E** (right). Data are presented as mean ± SD in **F** (right). Statistics were acquired by Kruskal-Wallis test followed by Dunn's multiple comparisons in **C**, **D**, and **E** (ACHN cells), by Mann-Whitney test in **E** (Caki-1 cells), by two-way ANOVA with (ACHN cells) or without (Caki-1 cells) Bonferroni multiple comparisons in **F**. ****, $P<0.0001$; ***, $P<0.001$; **, $P<0.01$; *, $P<0.05$; NS, not significant.

Supplemental Figure 14. TARBP2 modulates the p53/B7-H3 axis via 53BP1 phase separation. (**A**) Flow

cytometry analysis of cell-surface B7-H3 levels in *TARBP2*-knockdown and control 769-P cells treated with DMSO or 1,6-HD (1%, 4 h) (n=3 independent experiments). (**B**) Western blot analysis of total B7-H3 and p21 protein levels in *TARBP2*-knockdown and control 769-P cells treated with DMSO or 1,6-HD (1%, 4 h). GAPDH served as a loading control. (**C**) Flow cytometry analysis of cell-surface B7-H3 levels in *TARBP2*-knockdown and control ACHN cells treated with DMSO or 1,6-HD (1%, 4 h) (n=3 independent experiments). (**D**) Western blot analysis of total B7-H3 and p21 protein levels in *TARBP2*-knockdown and control ACHN cells treated with DMSO or 1,6-HD (1%, 4 h). GAPDH served as a loading control. (**E**) Flow cytometry analysis showing the effect of Cry2-mCherry-53BP1 fusion proteins with or without the OD, under blue light stimulation or in the dark, on cell-surface B7-H3 levels in *TARBP2*-overexpressing Caki-1 cells (n=3 independent experiments). (**F**) Western blot analysis showing the effect of Cry2-mCherry-53BP1 fusion proteins with or without the OD, under blue light stimulation or in the dark, on total B7-H3 and p21 protein levels in *TARBP2*-overexpressing Caki-1 cells. GAPDH served as a loading control. All data with error bars are presented as mean ± SD. Statistics were acquired by one-way ANOVA followed by Tukey's multiple comparisons in **A**, **C**, and **E**. ****, *P*<0.0001; ***, *P*<0.001; NS, not significant.

Supplemental Figure 15. Multiplex immunohistochemical analysis of TARBP2 expression. (**A** and **B**) Representative images (**A**) and quantification (**B**) of TARBP2 expression in $CD3^+$ cells, $CD3^-CD56^+$ cells, and $PanCK^+$ cells in samples from ICB responders and non-responders (n=5 samples from different patients per group). Scale bars, 10 μm and 10 μm (insets). Data are presented as mean ± SD in **B**, Statistics were acquired by one-way ANOVA followed by Tukey's multiple comparisons in **B**. ****, *P*<0.0001; ***, *P*<0.001; NS, not significant.

Supplemental Figure 16. TARBP2 attenuates NK cell effector function in the RCC patient-derived xenograft model. (**A**) Representative patient-derived xenograft (PDX) tumor sections from NCG mice treated with siRNA monotherapy stained with hematoxylin-eosin (HE), TARBP2, B7-H3, and Cleaved Caspase-3. Scale bar, 50 μm. (**B**) Quantification of total TARBP2, nuclear TARBP2, B7-H3, and Cleaved Caspase-3 expression in PDX tumor sections from NCG mice receiving indicated treatments (n=5 mice per group). (**C**) Proportions of TNF-α^+ and IFN-γ^+ cells in $CD3^-CD56^+$ NK cells from indicated PDX tumors in NCG mice (n=5 mice per group). All data with error bars are presented as mean ± SD. Statistics were acquired by one-way ANOVA followed by Tukey's multiple comparisons in **B**, by unpaired two-tailed Student's t-test in **C**. ****, *P*<0.0001; **, *P*<0.01; *, *P*<0.05; NS, not significant.

Supplemental Figure 17. HE staining of orthotopic tumor sections. Representative HE staining images of orthotopic tumor sections from BALB/c mice in the indicated groups. Scale bars, 1 mm and 50 μm (insets).

Supplemental Figure 18. Body weight monitoring and histological analysis of organ sections in the orthotopic tumor model. (**A**) Body weight curves of BALB/c mice from the indicated groups (n=5 mice per group). (**B**) Representative HE-stained sections of tumor-free organs from BALB/c mice in the indicated groups. Scale bars, 50 μm for each organ. All data with error bars are presented as mean ± SD. Statistics were acquired by two-way ANOVA followed by Bonferroni multiple comparisons in **A**. *, *P*<0.05; NS, not significant.

Supplemental Figure 19. Targeting TARBP2 combined with ICB therapy enhances NK cell effector function in the orthotopic tumor model. (**A**) Gating strategy used to profile intratumoral NK cells in orthotopic tumors from BALB/c mice. (**B**) Proportions of $GZMB^+$, TNF-α^+, and IFN-γ^+ cells among $CD3^-CD49b^+$ NK cells in orthotopic tumors from the indicated groups (n=5 mice per group). All data with error bars are presented as mean ± SD. Statistics were acquired by one-way ANOVA followed by Tukey's multiple comparisons in **B**. ****, *P*<0.0001; ***, *P*<0.001; **, *P*<0.01; *, *P*<0.05; NS, not significant.
