# Supplementary material for: TARBP2 facilitates renal cell carcinoma immune escape by suppressing 53BP1 phase separation to upregulate B7-H3
Source: J Clin Invest. 2026 Nov 2;136(21):e199525. doi: 10.1172/JCI199525 (PMC13722402; doi:10.1172/JCI199525)

# Full unedited blot for Figure 4I

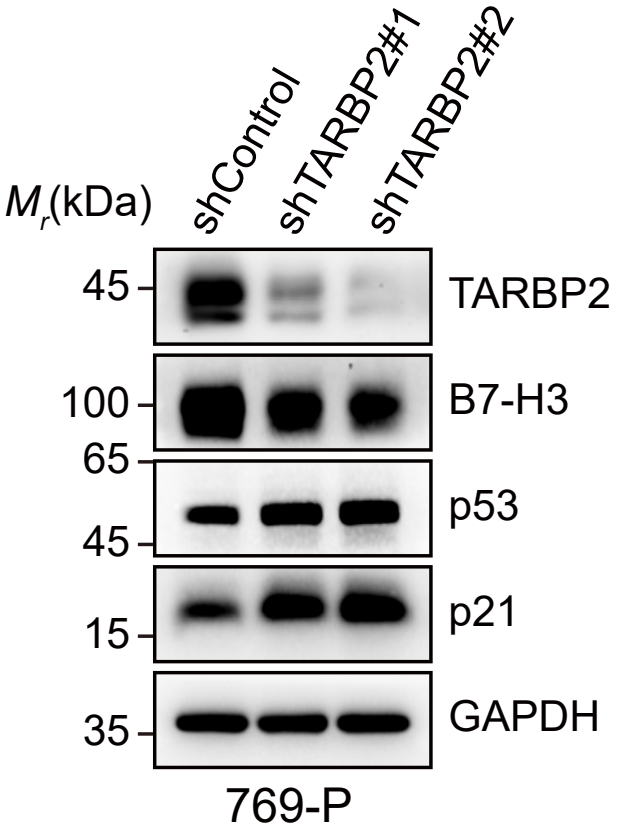


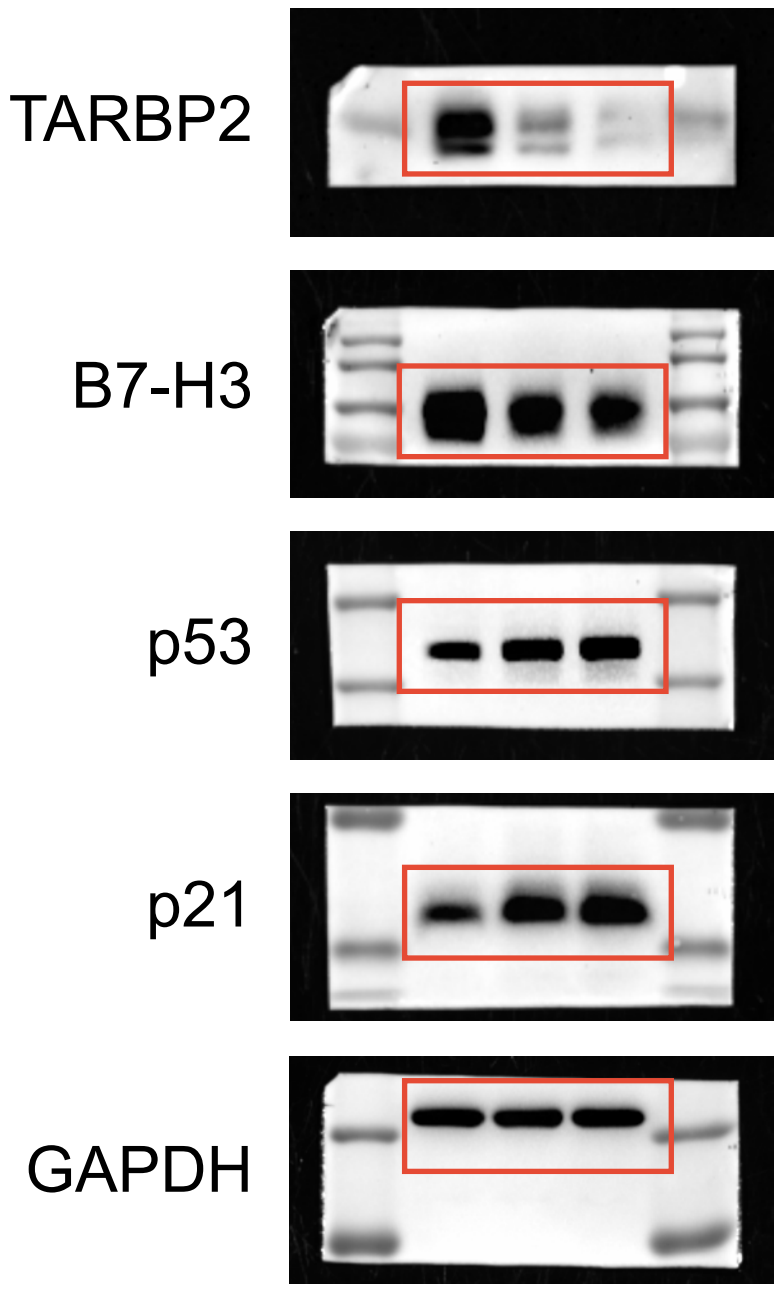

# Full unedited blot for Figure 4K

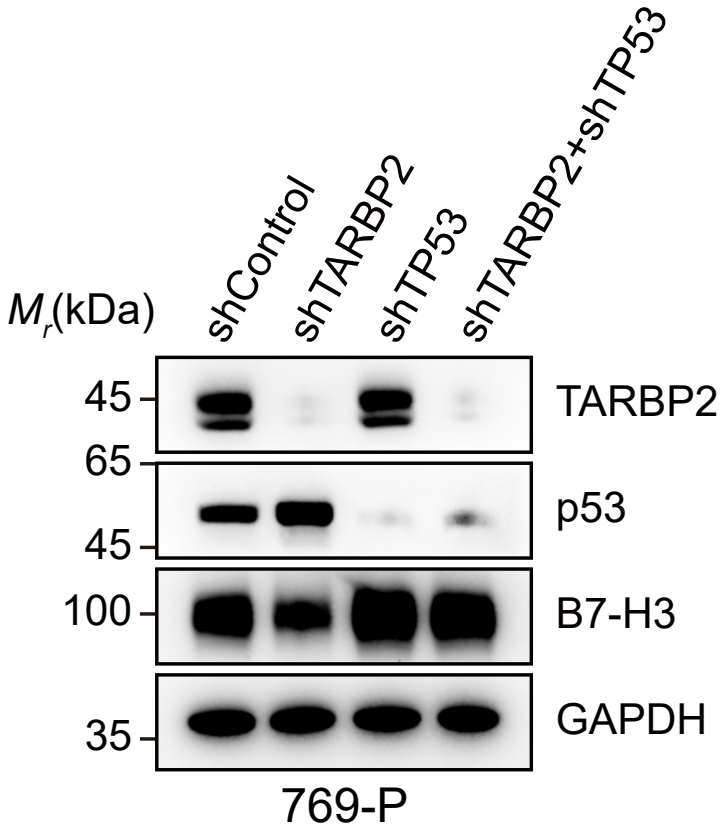


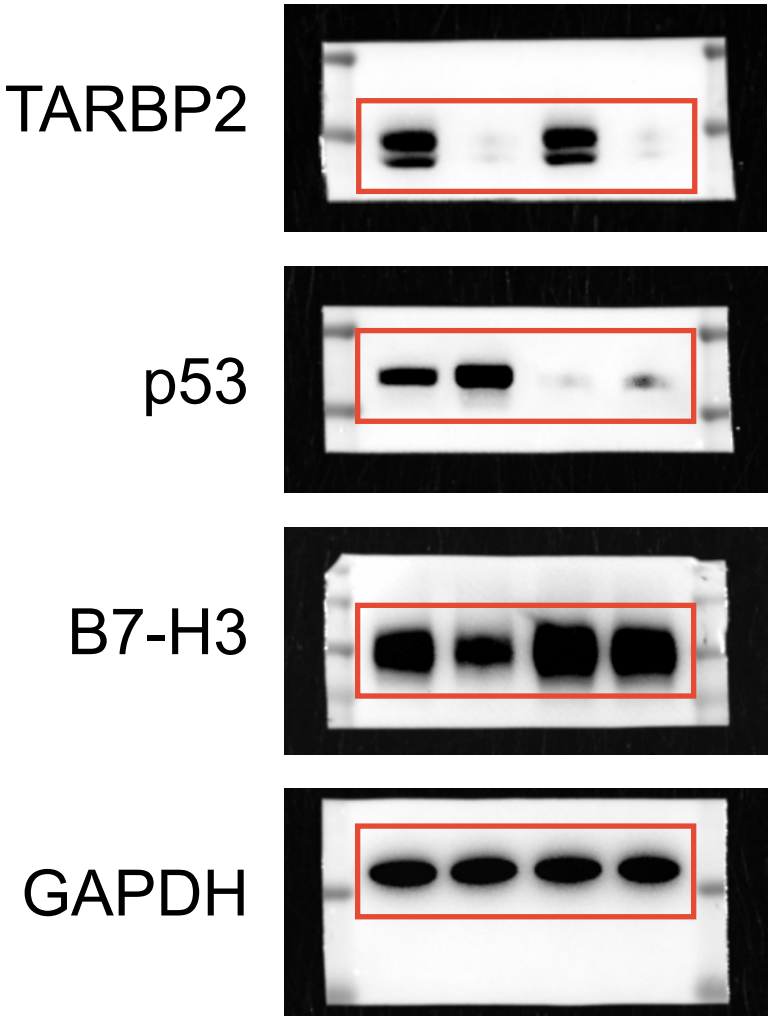

Full unedited blot for Figure 5B

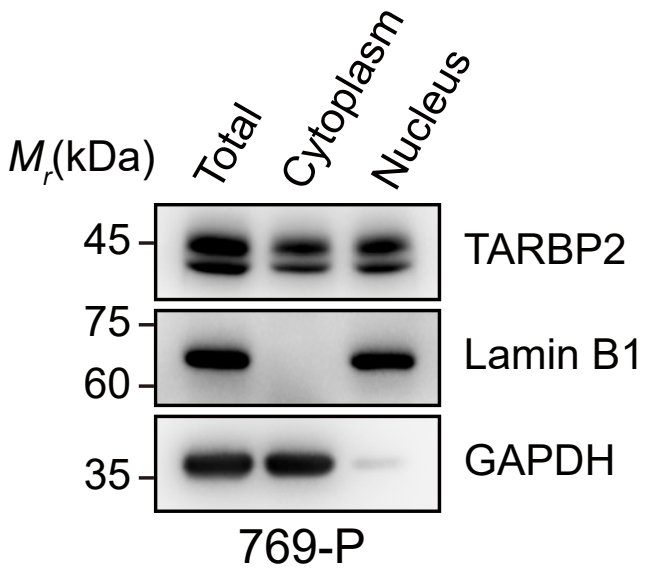


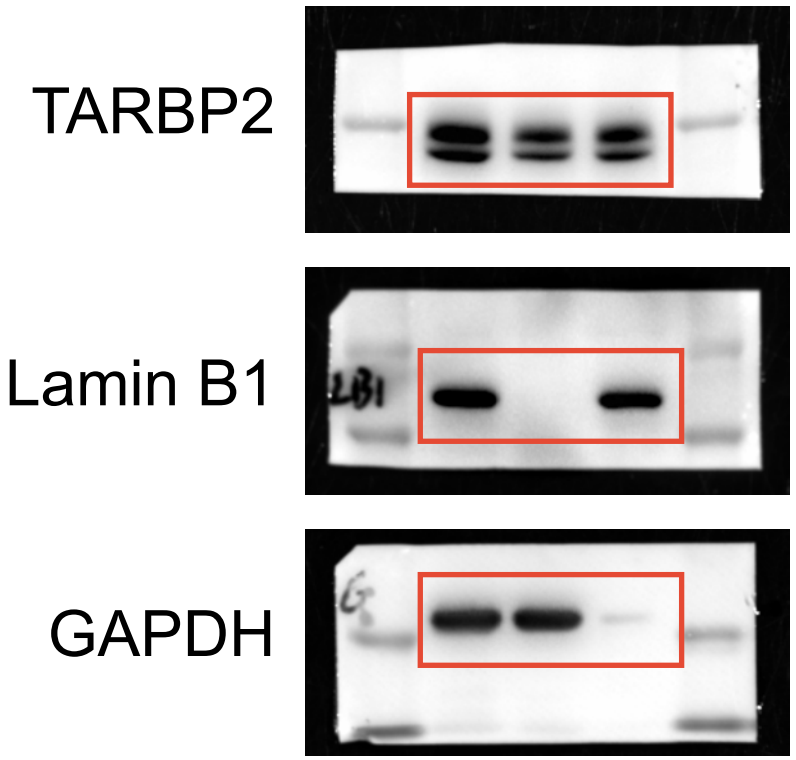

# Full unedited blot for Figure 5C

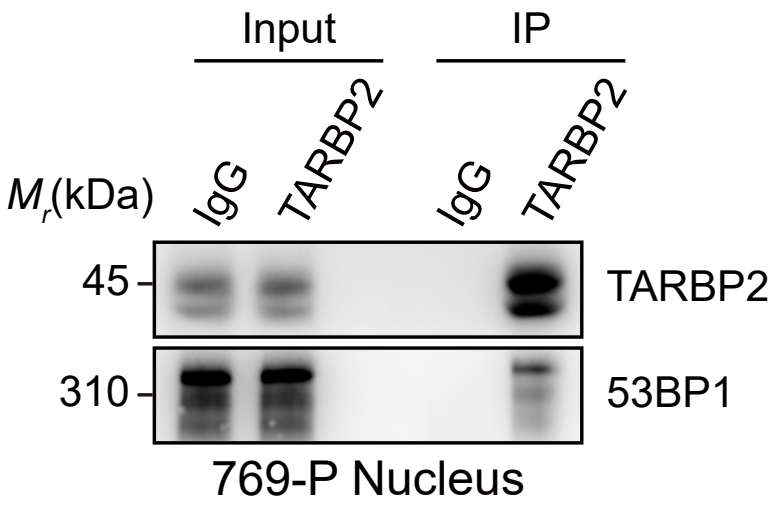


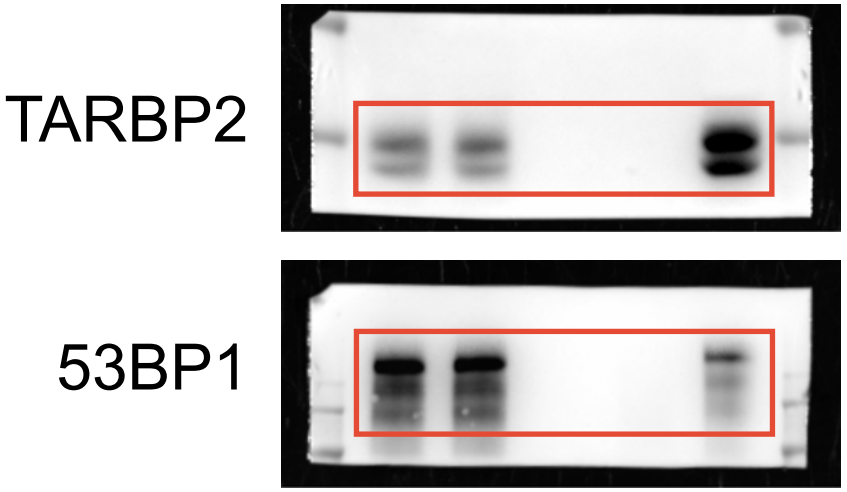

# Full unedited blot for Figure 5D

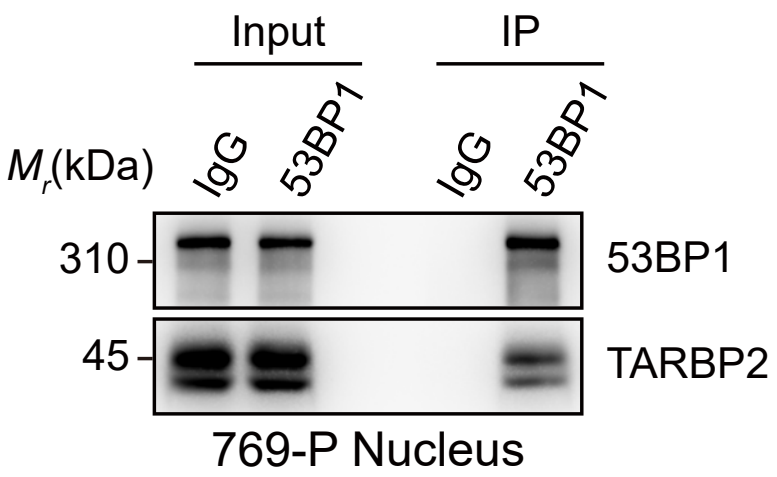


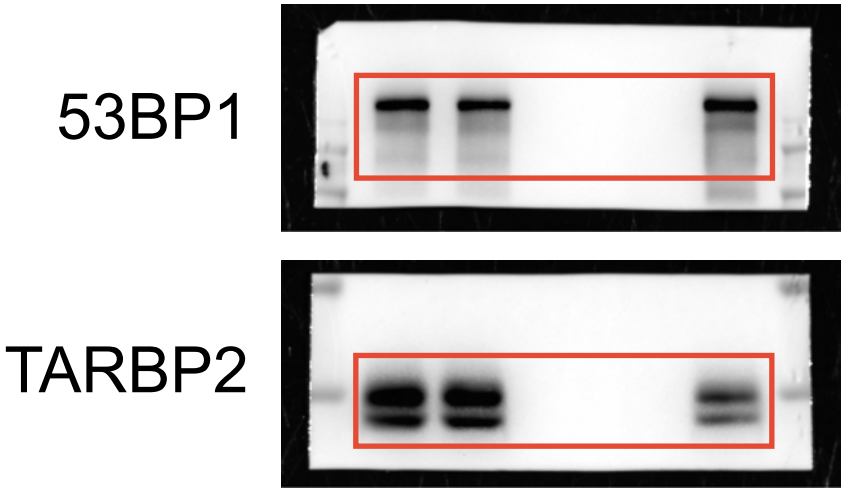

Full unedited blot for Figure 5E

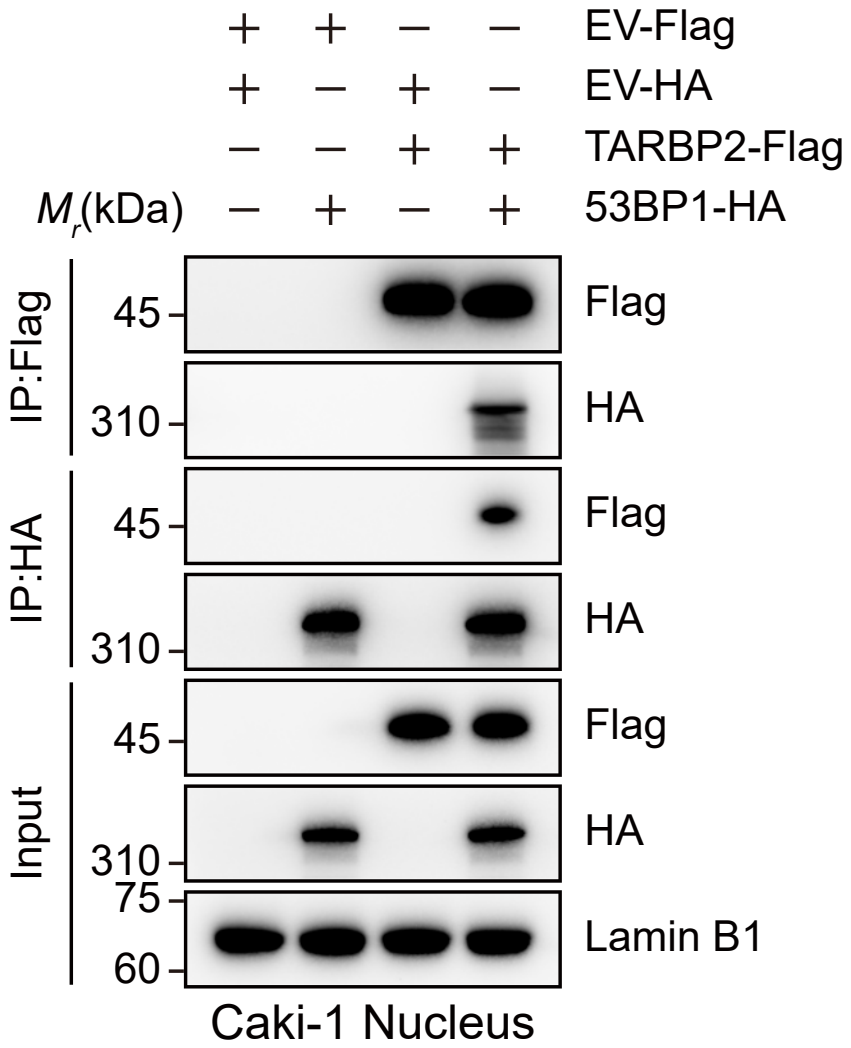


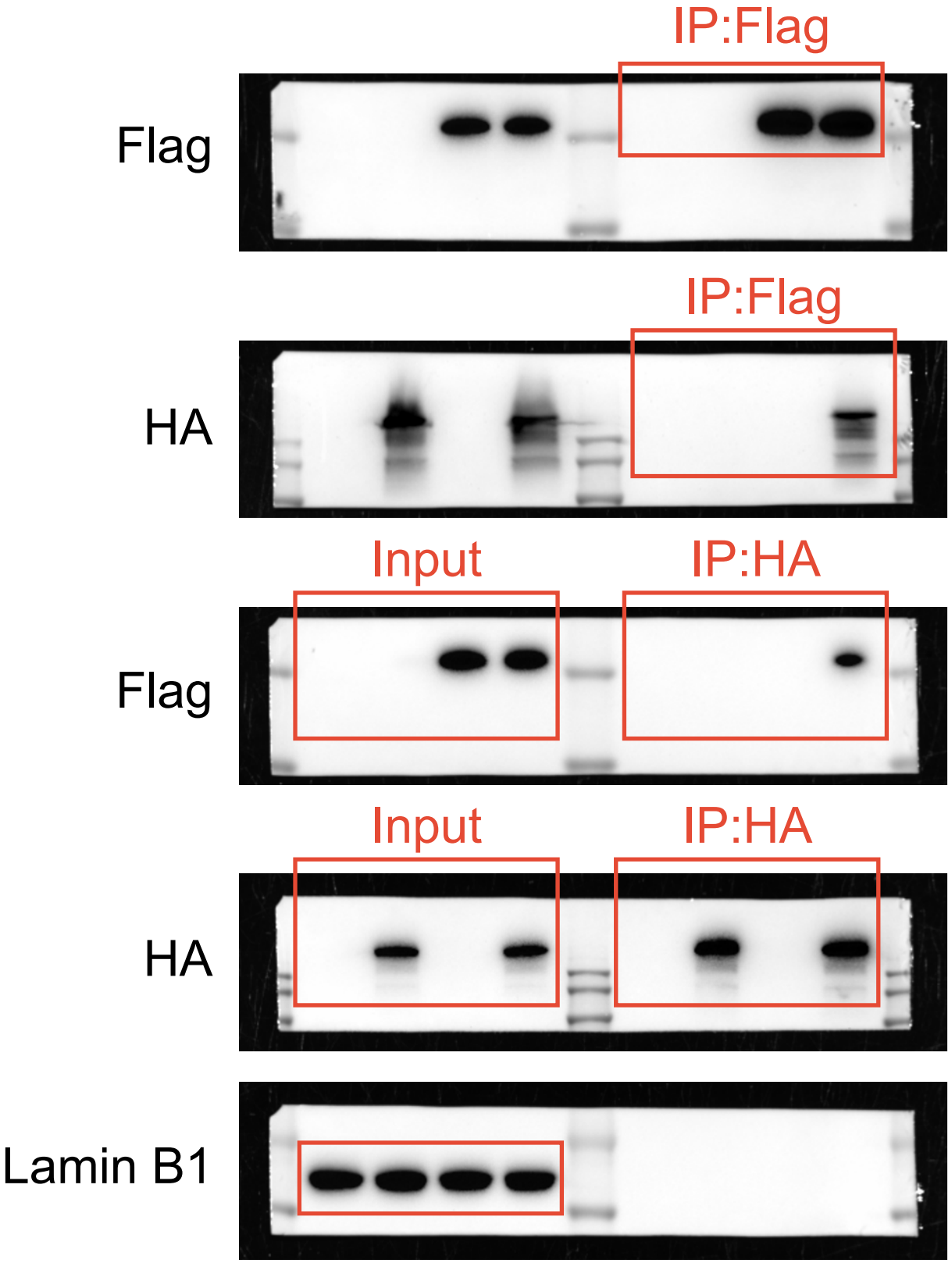

# Full unedited blot for Figure 5G

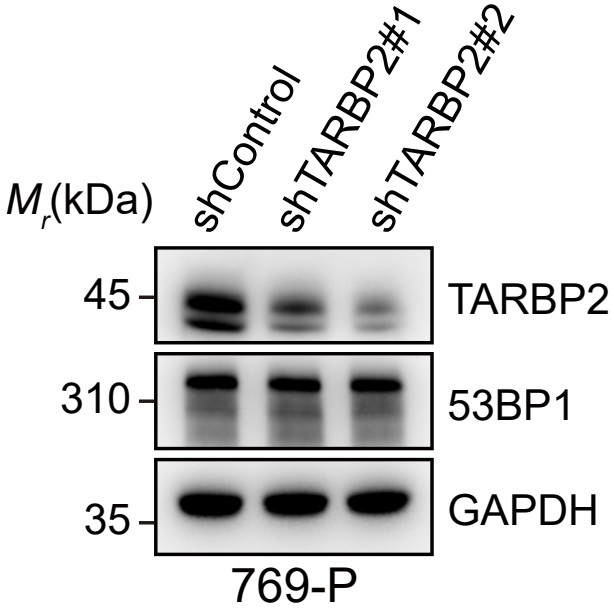


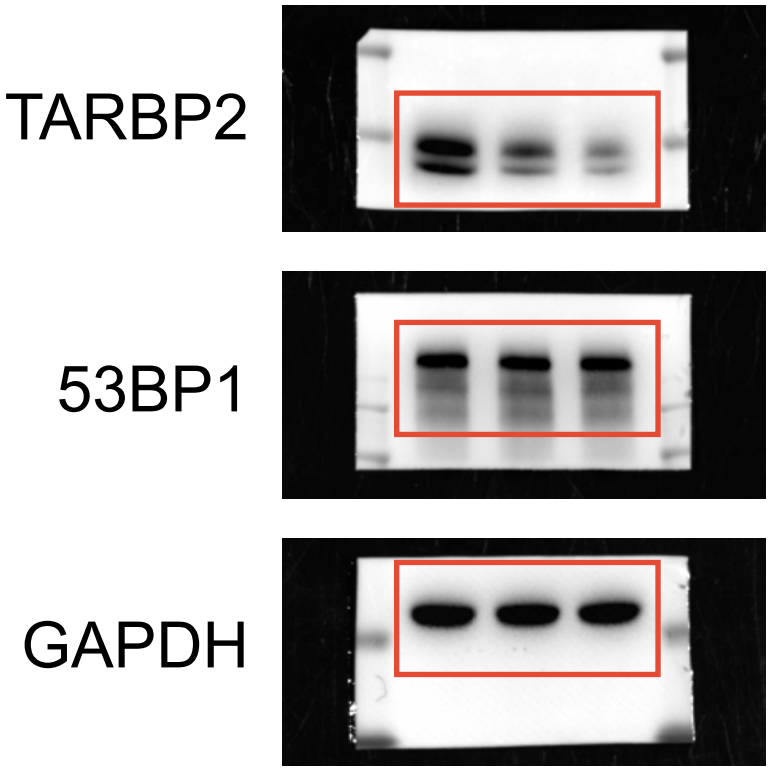

# Full unedited blot for Figure 5H

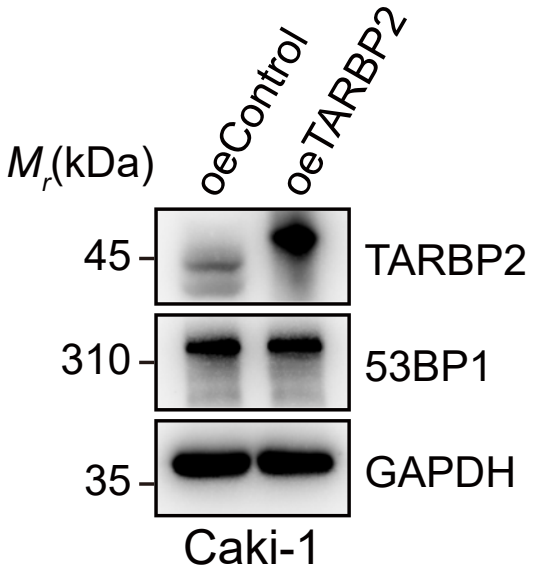


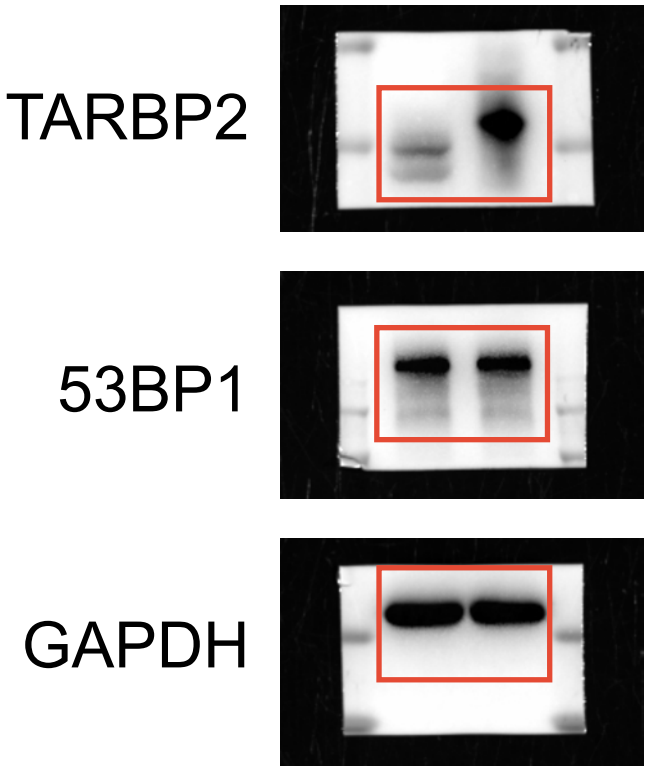

# Full unedited blot for Figure 5K

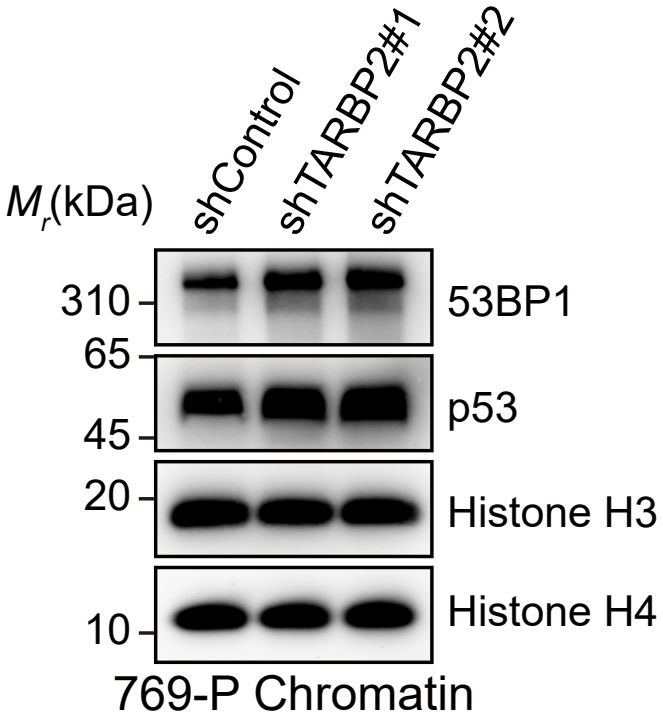


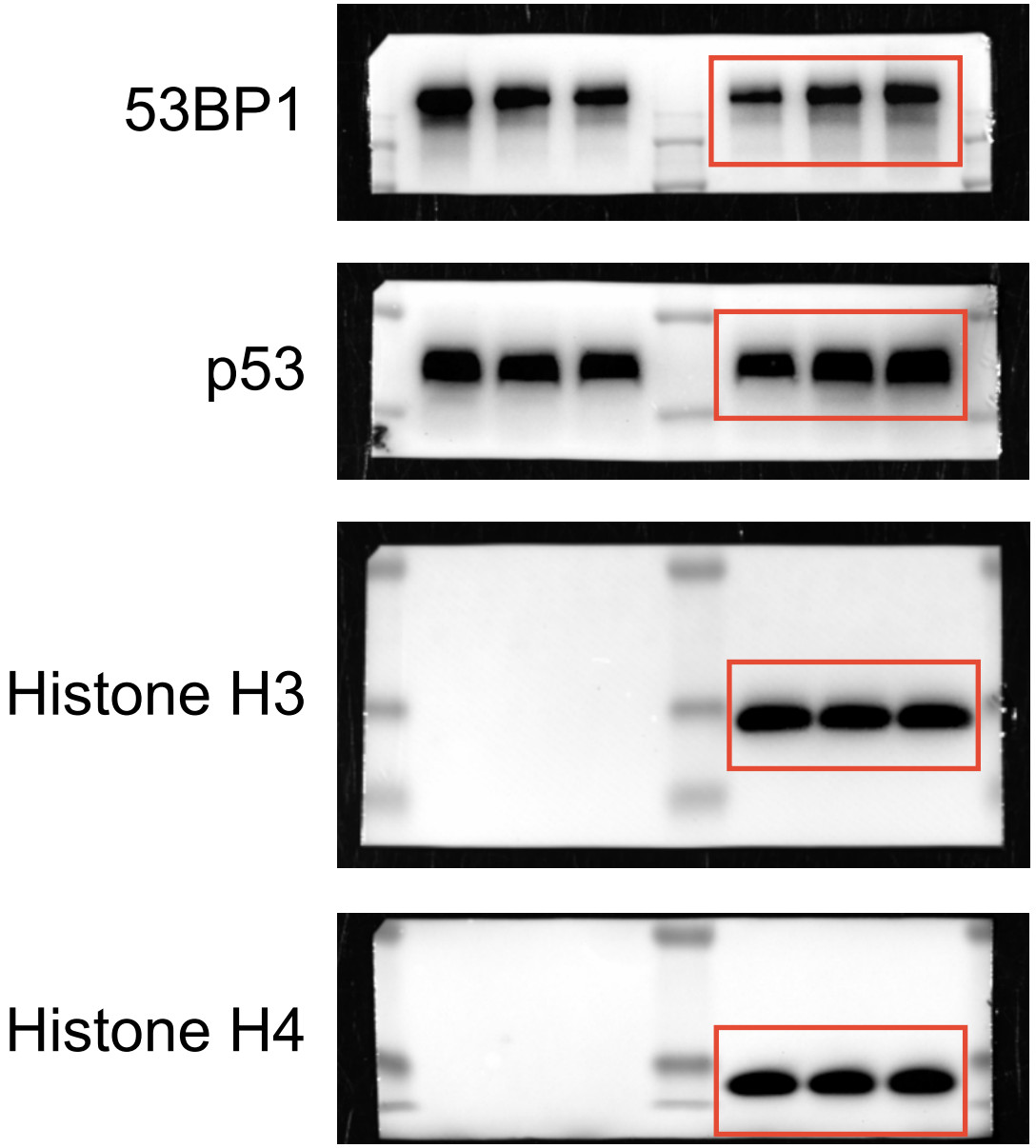

# Full unedited blot for Figure 5L

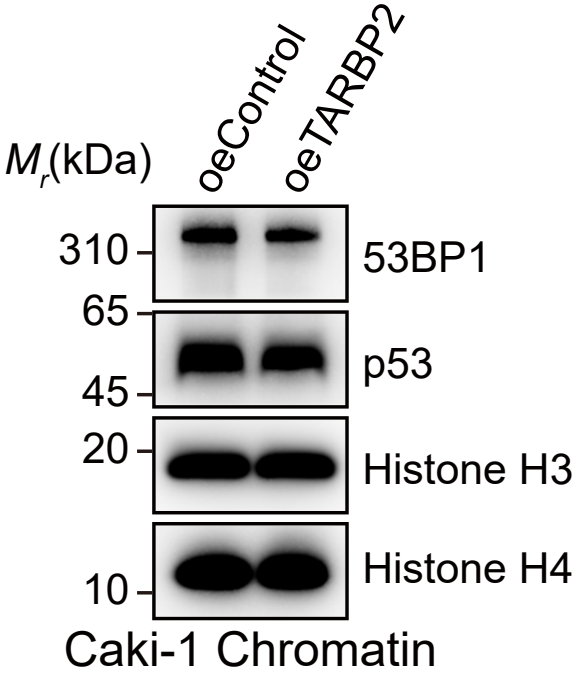


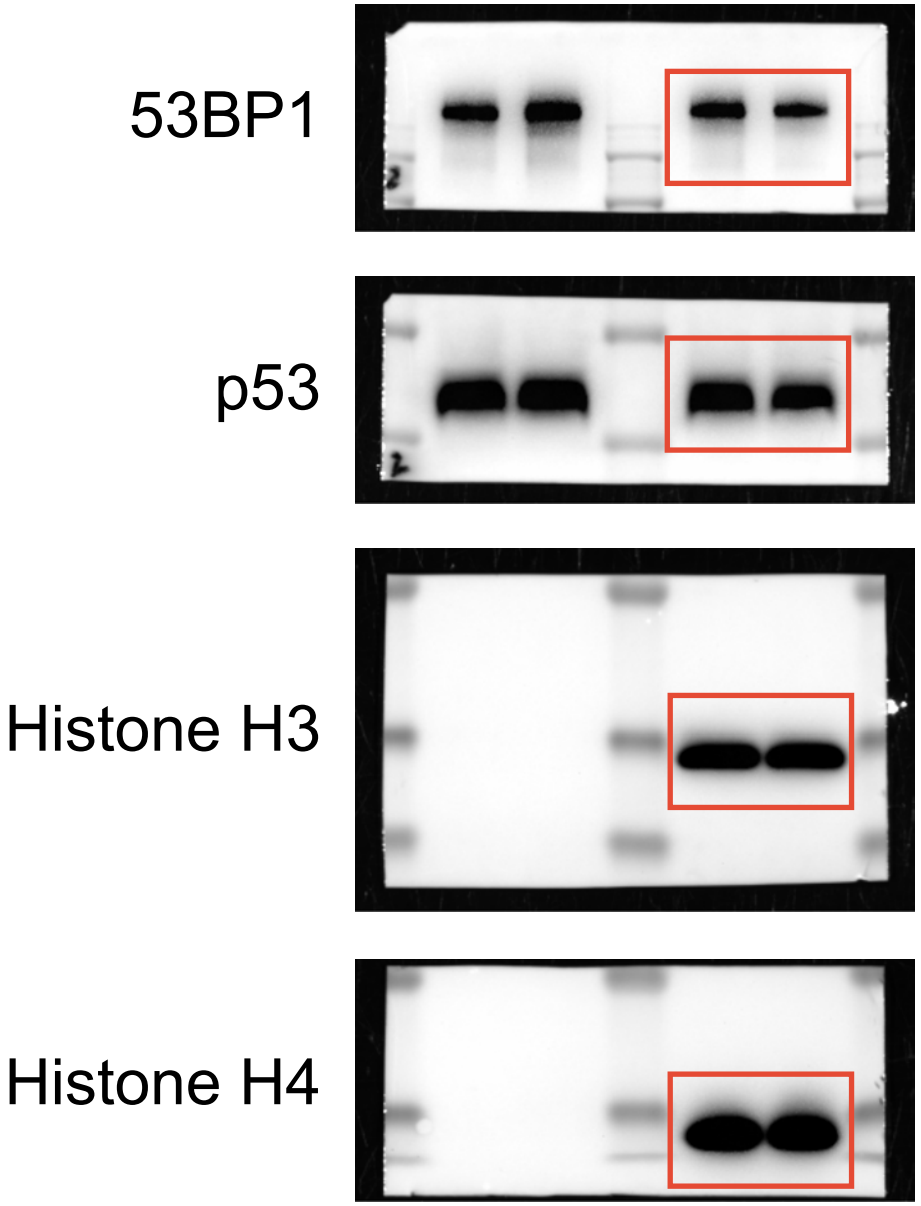

# Full unedited blot for Figure 5N

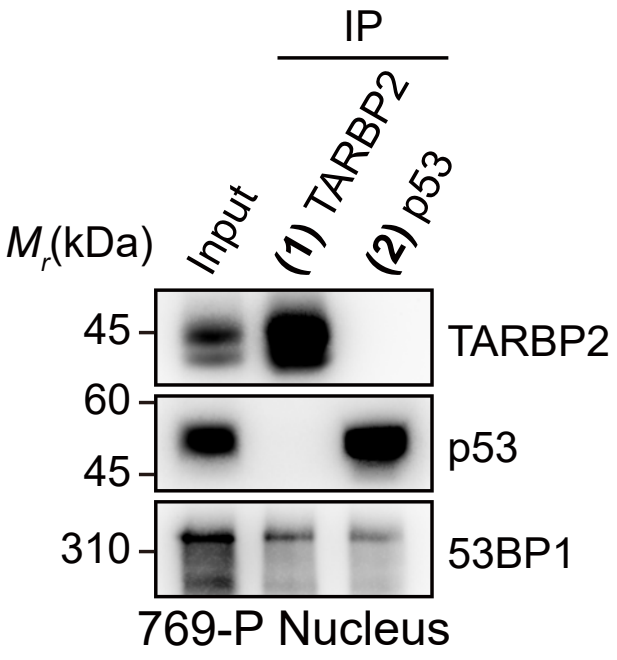


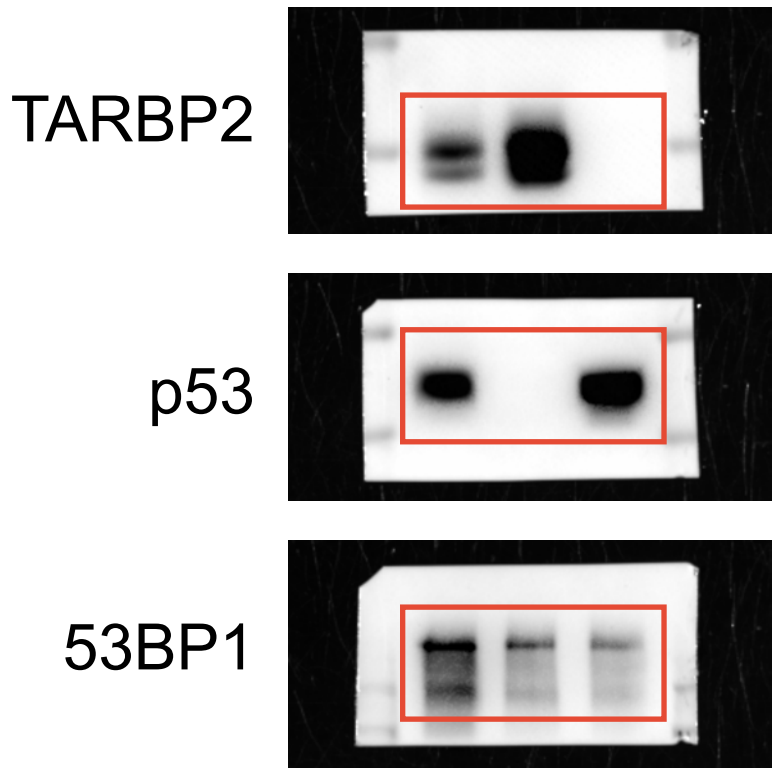

# Full unedited blot for Figure 6C

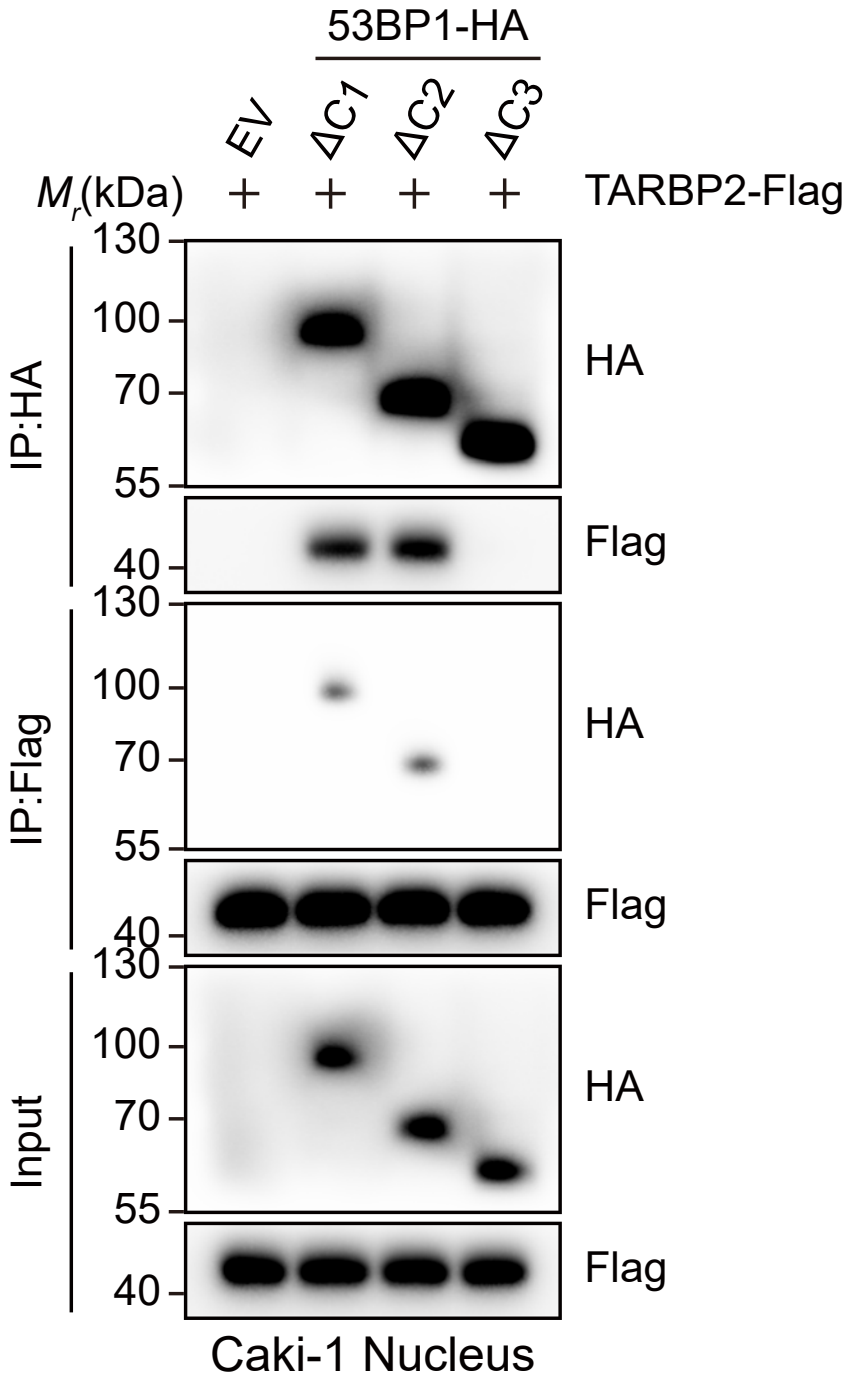


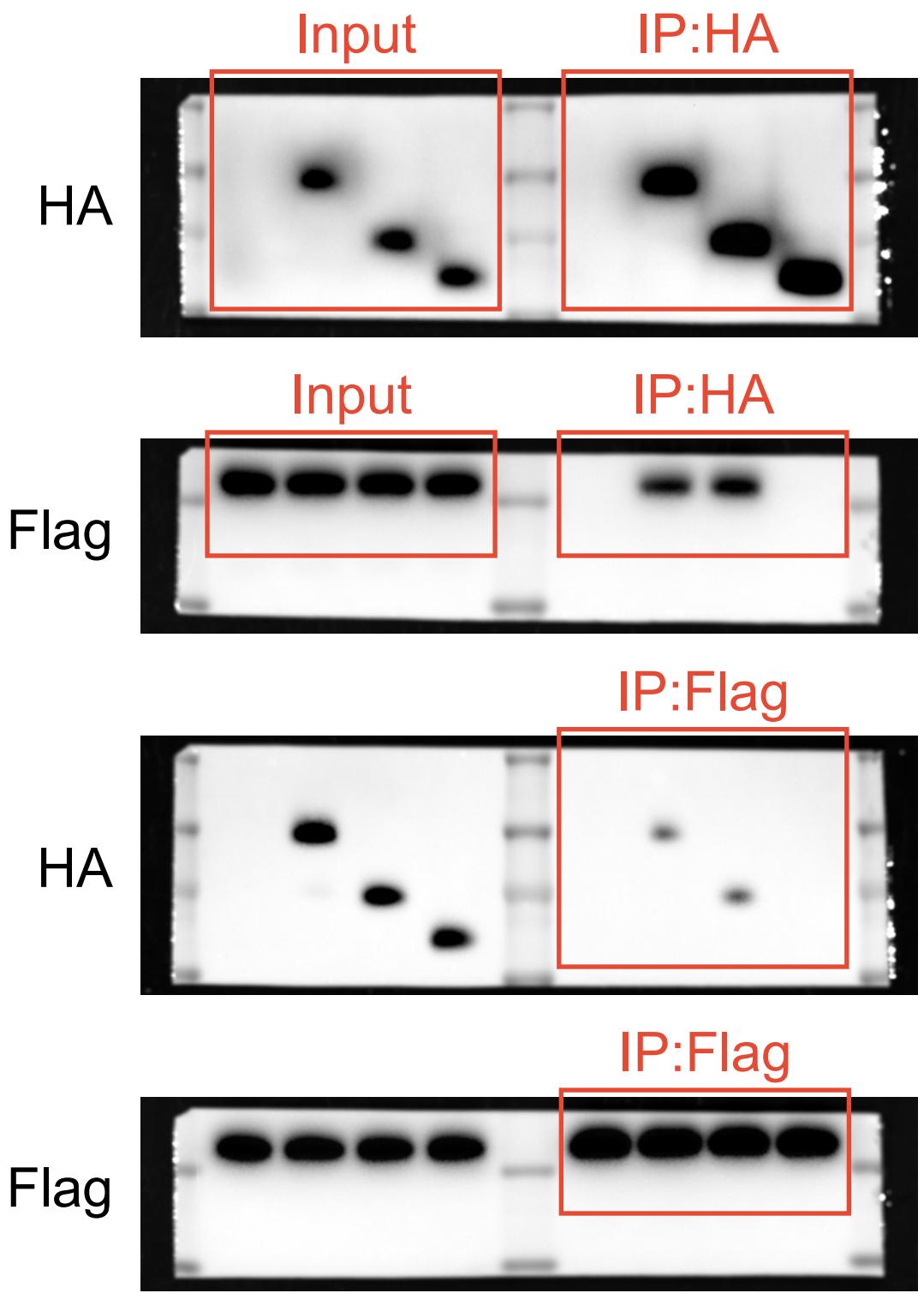

# Full unedited blot for Figure 6D

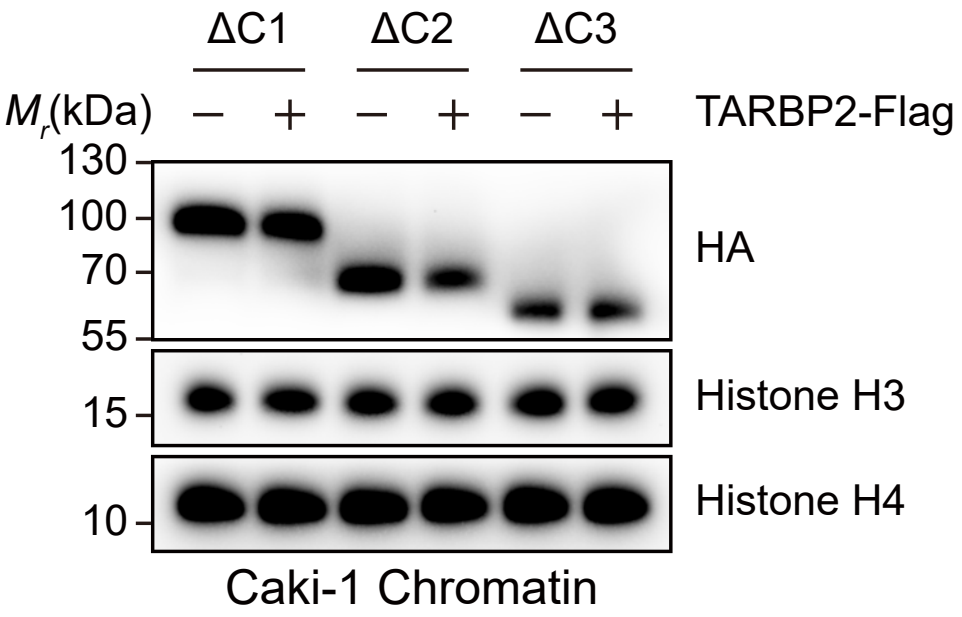


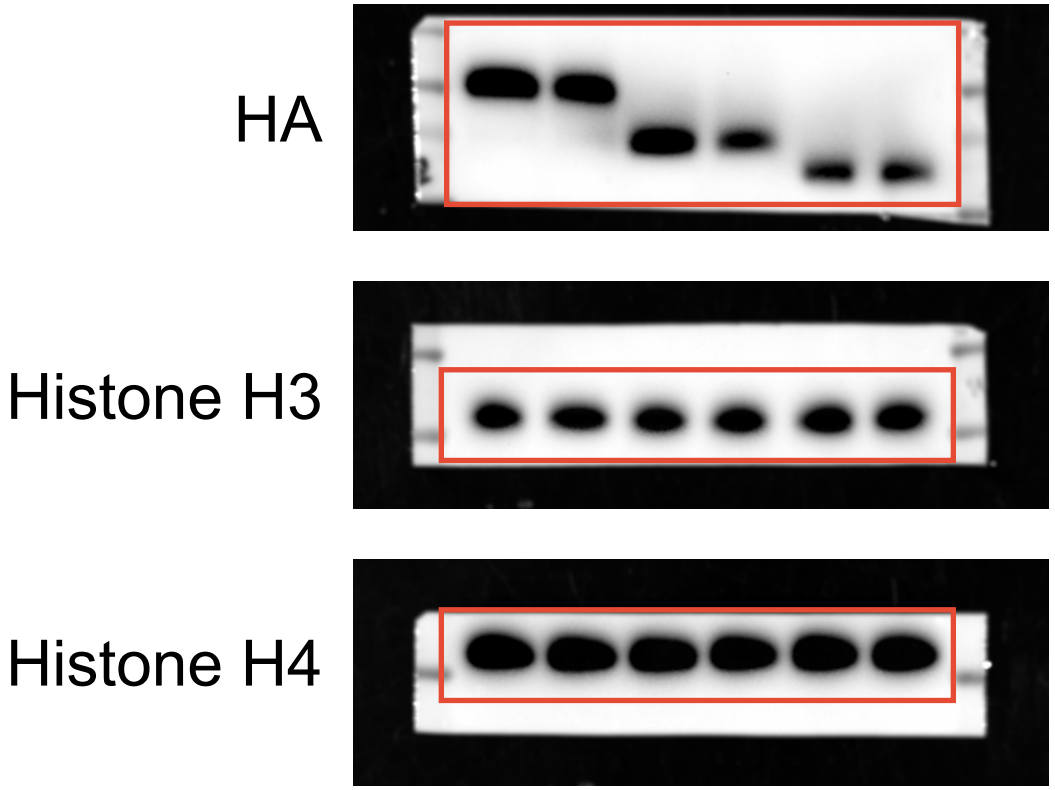

Full unedited blot for Figure 6E

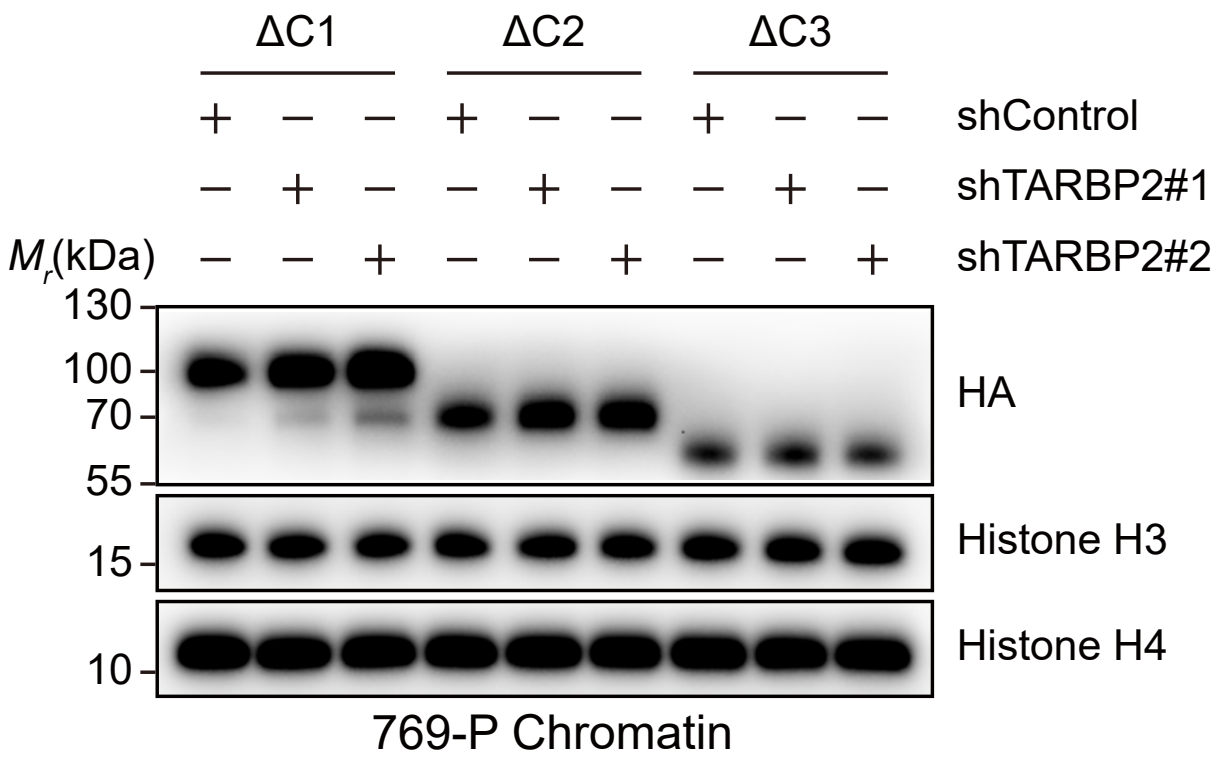


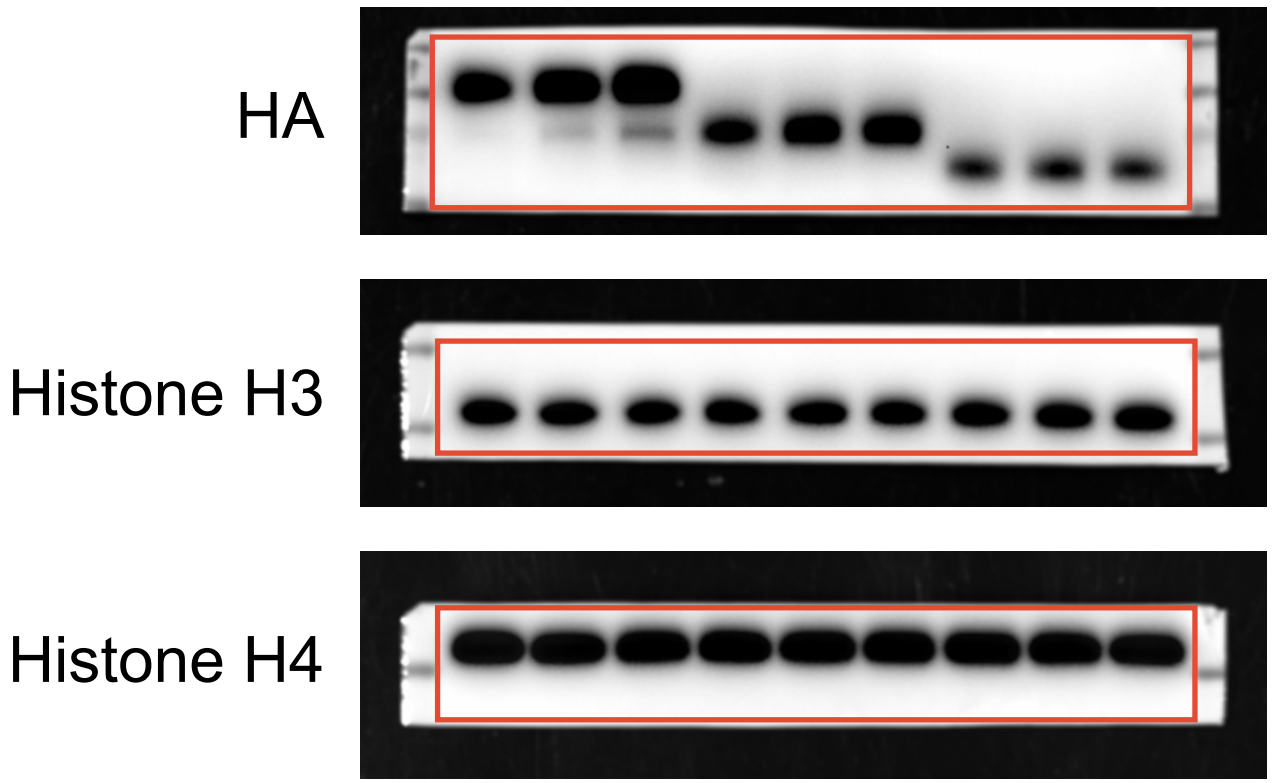

# Full unedited blot for Supplemental Figure 3A

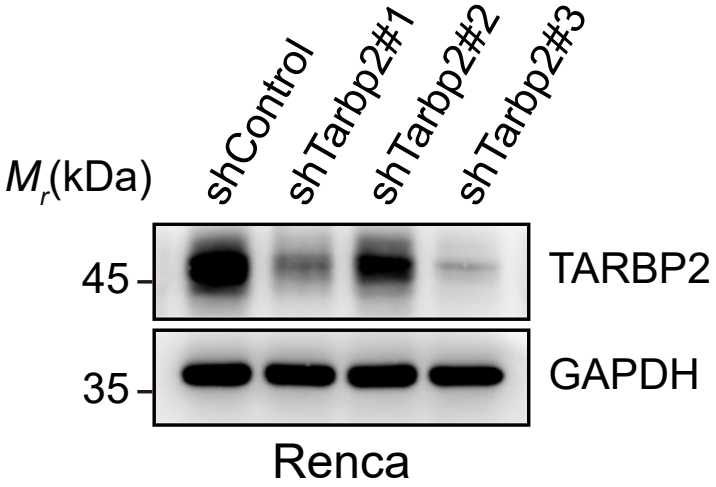


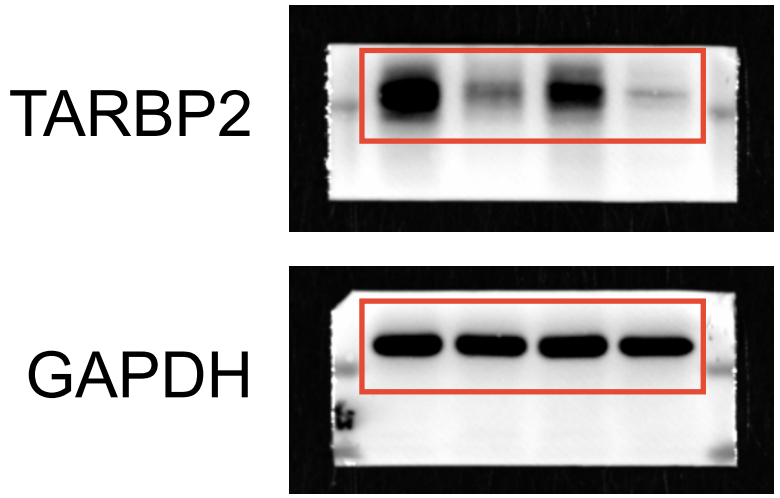

# Full unedited blot for Supplemental Figure 8G

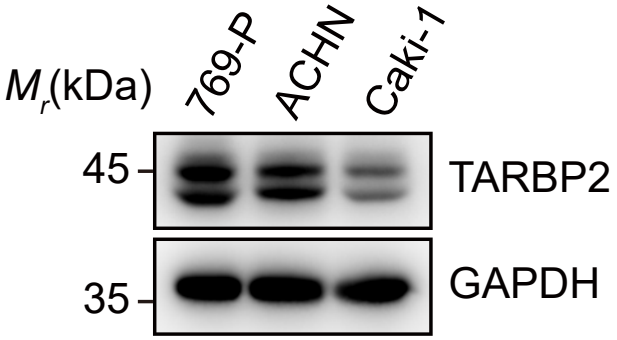


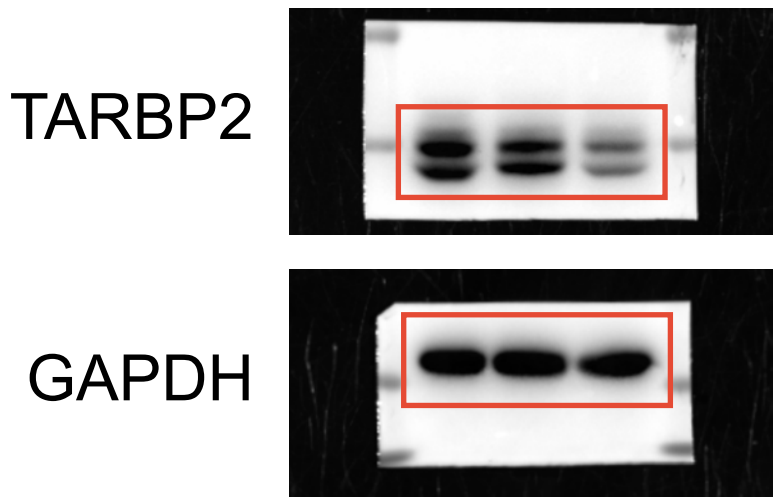

# Full unedited blot for Supplemental Figure 8J

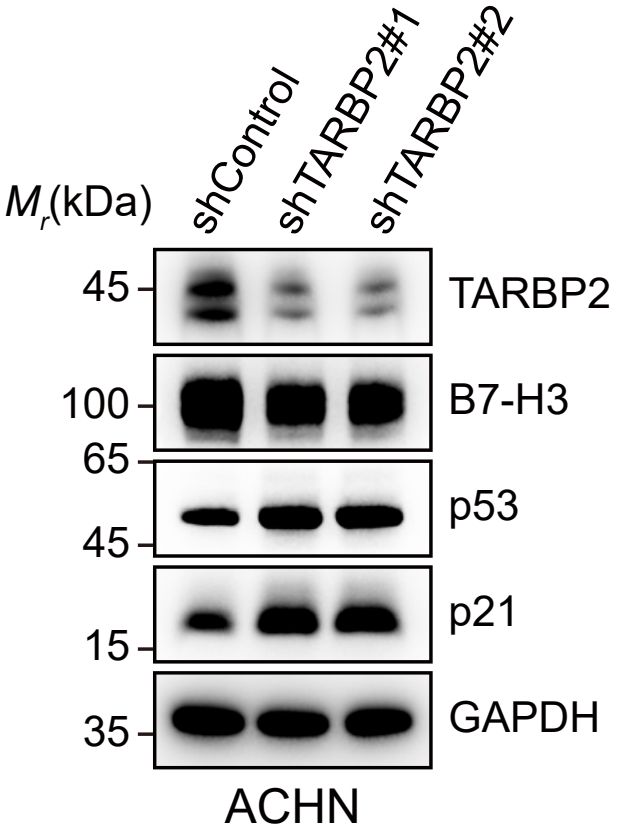


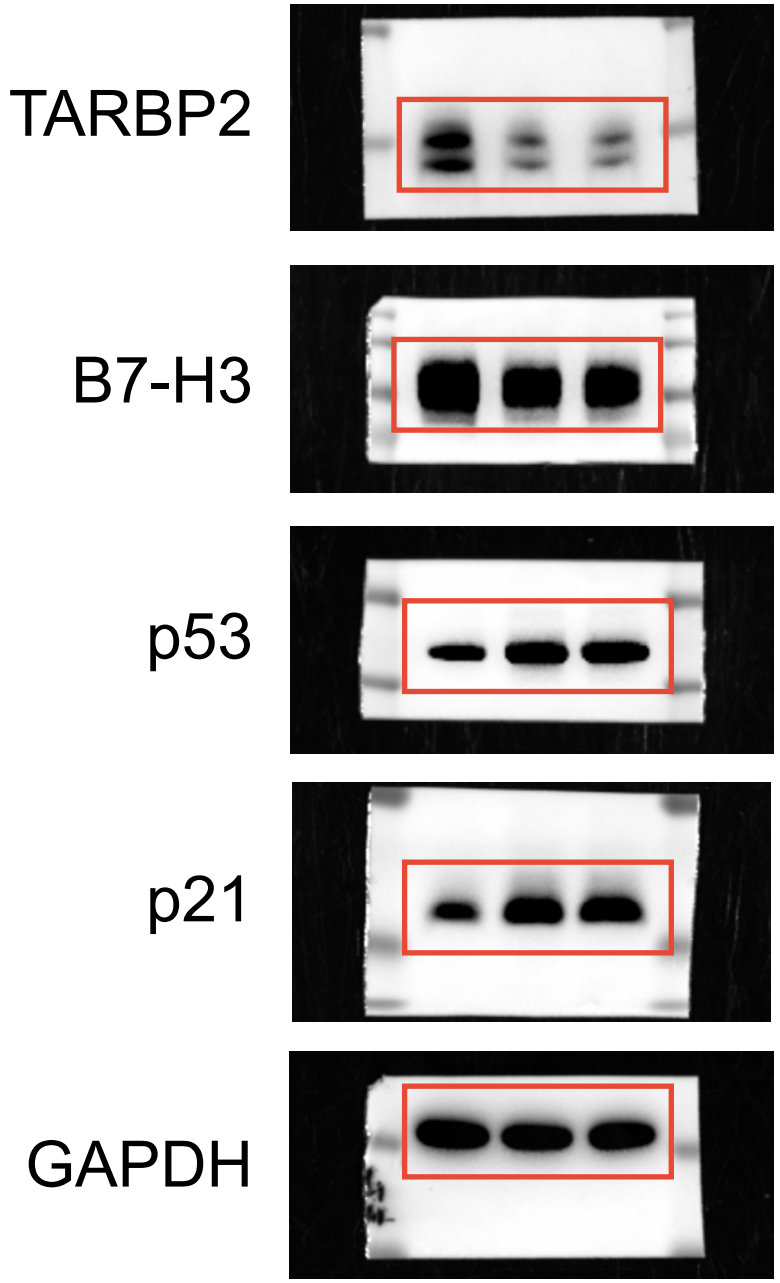

Full unedited blot for Supplemental Figure 8L

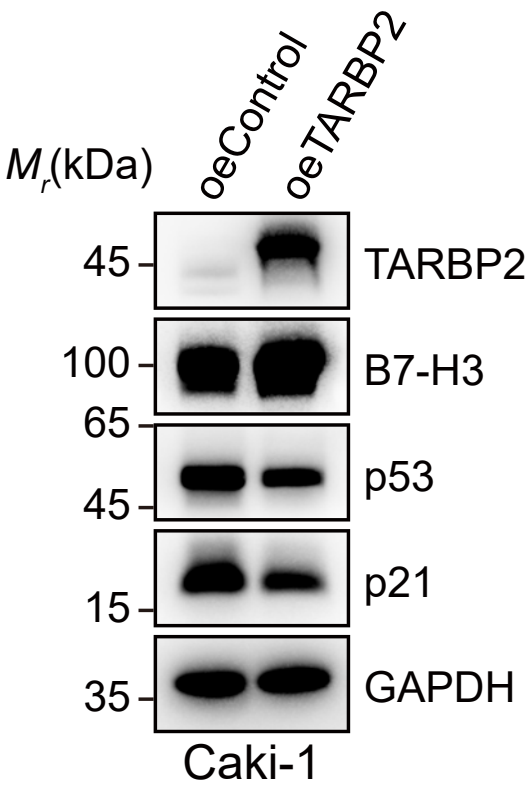


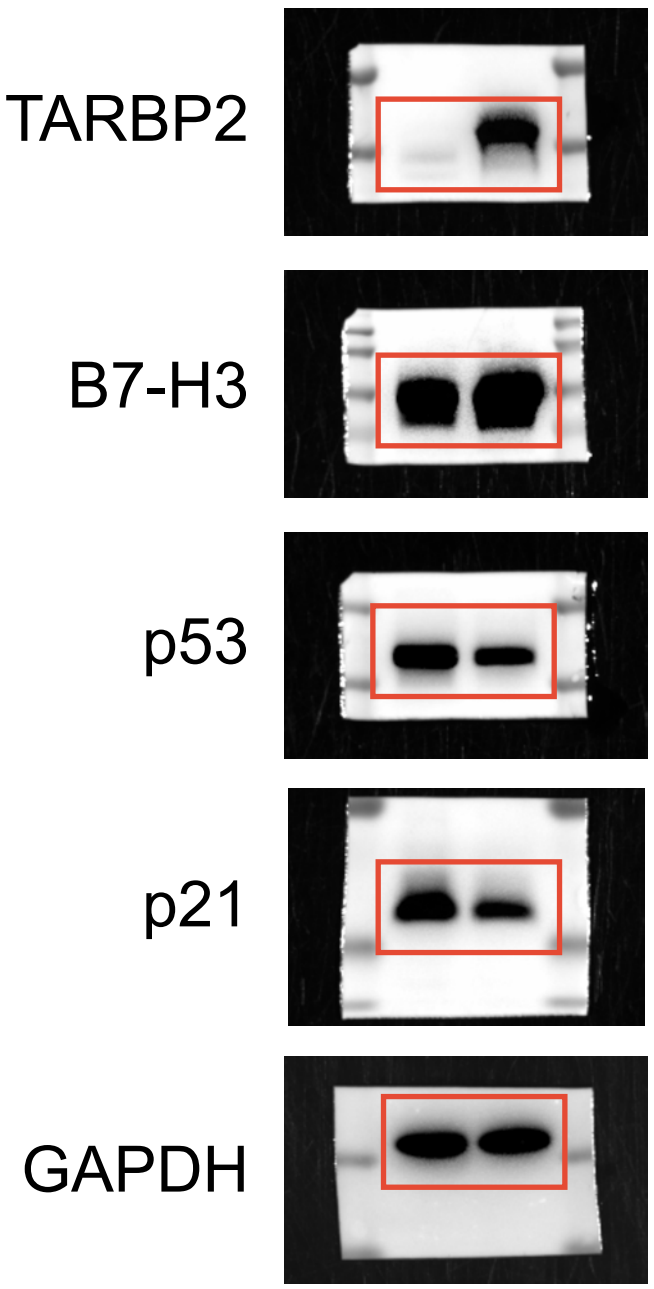

# Full unedited blot for Supplemental Figure 9B

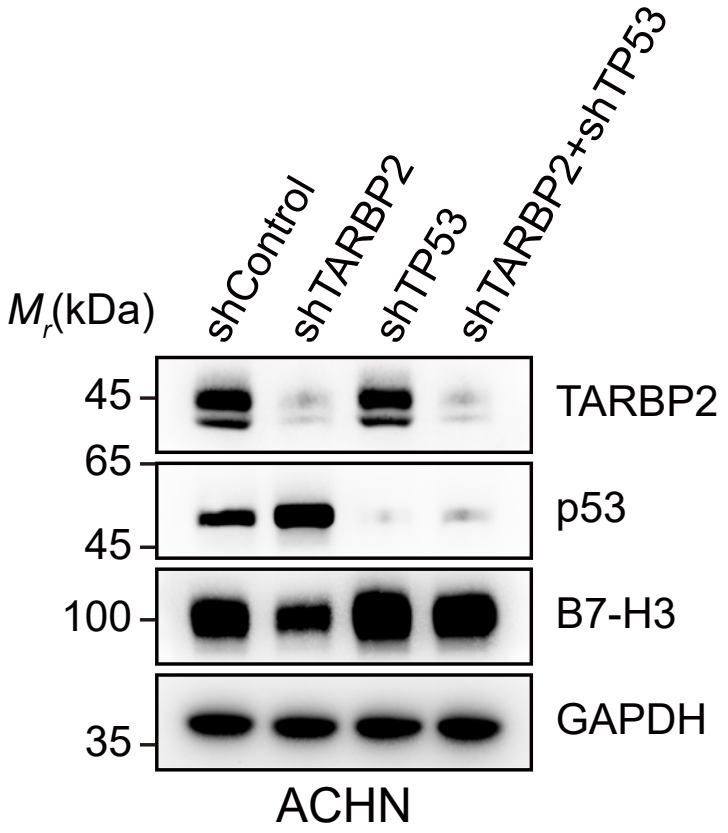


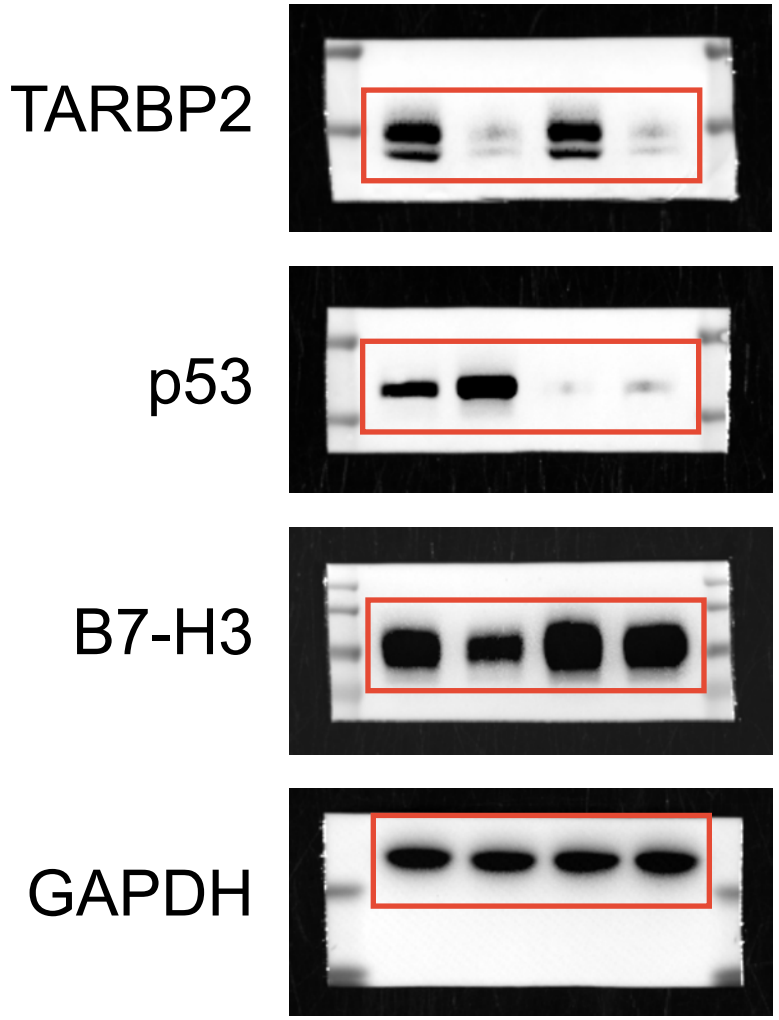

# Full unedited blot for Supplemental Figure 9D

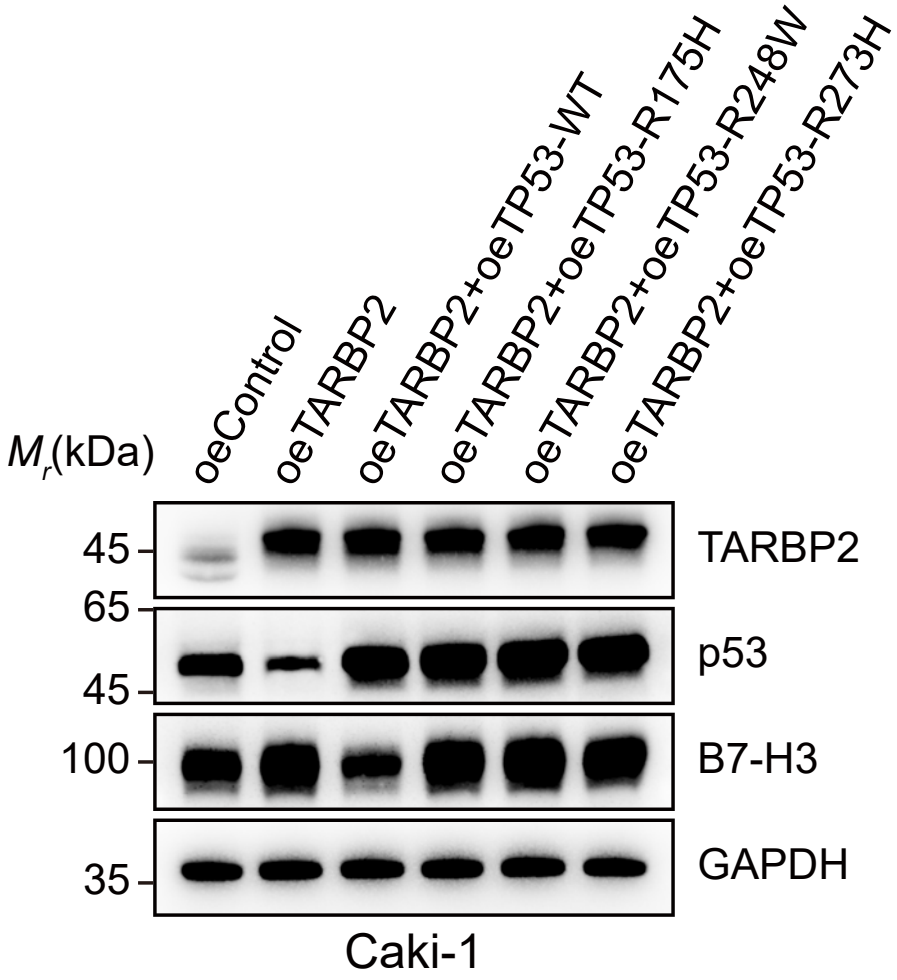


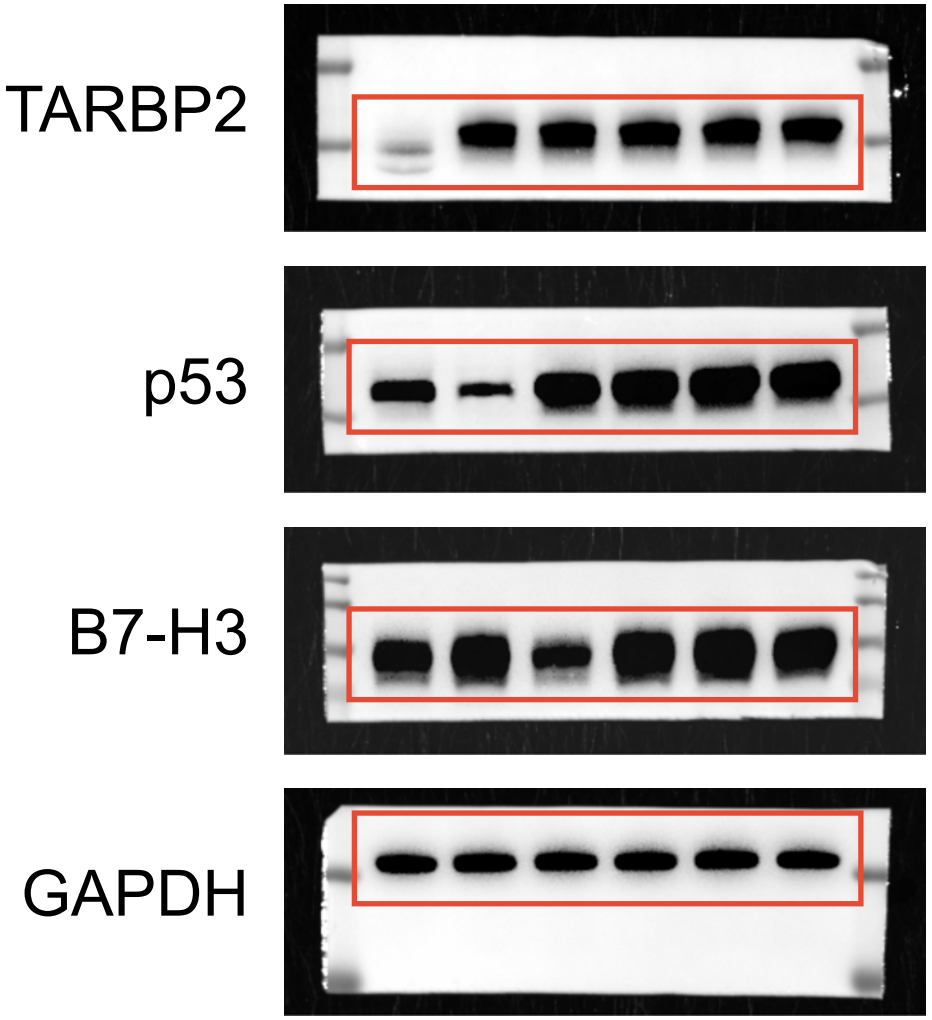

Full unedited blot for Supplemental Figure 11E

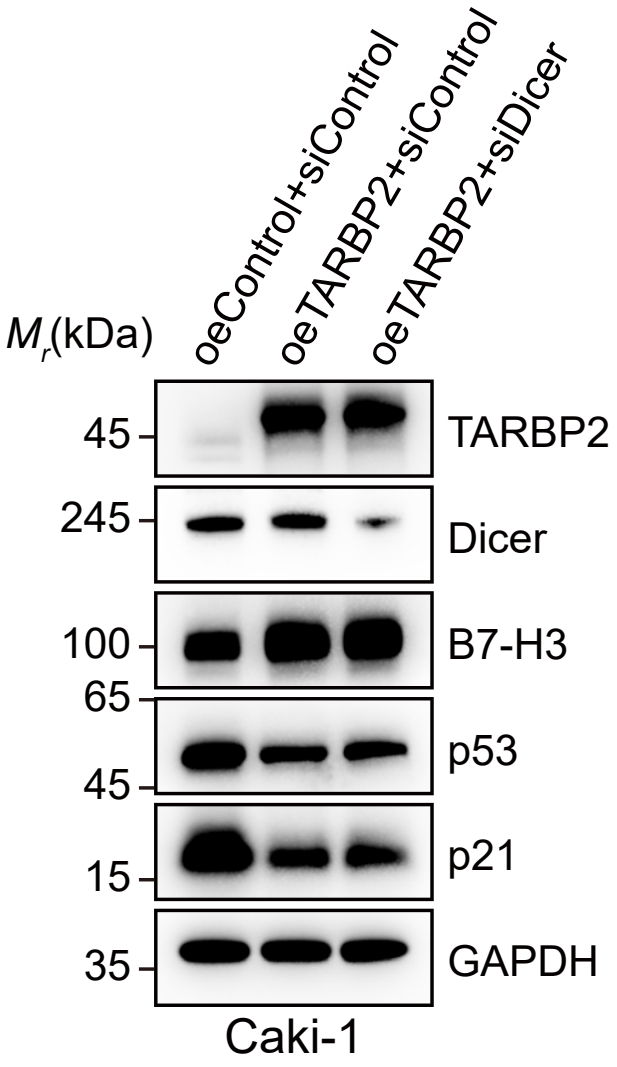


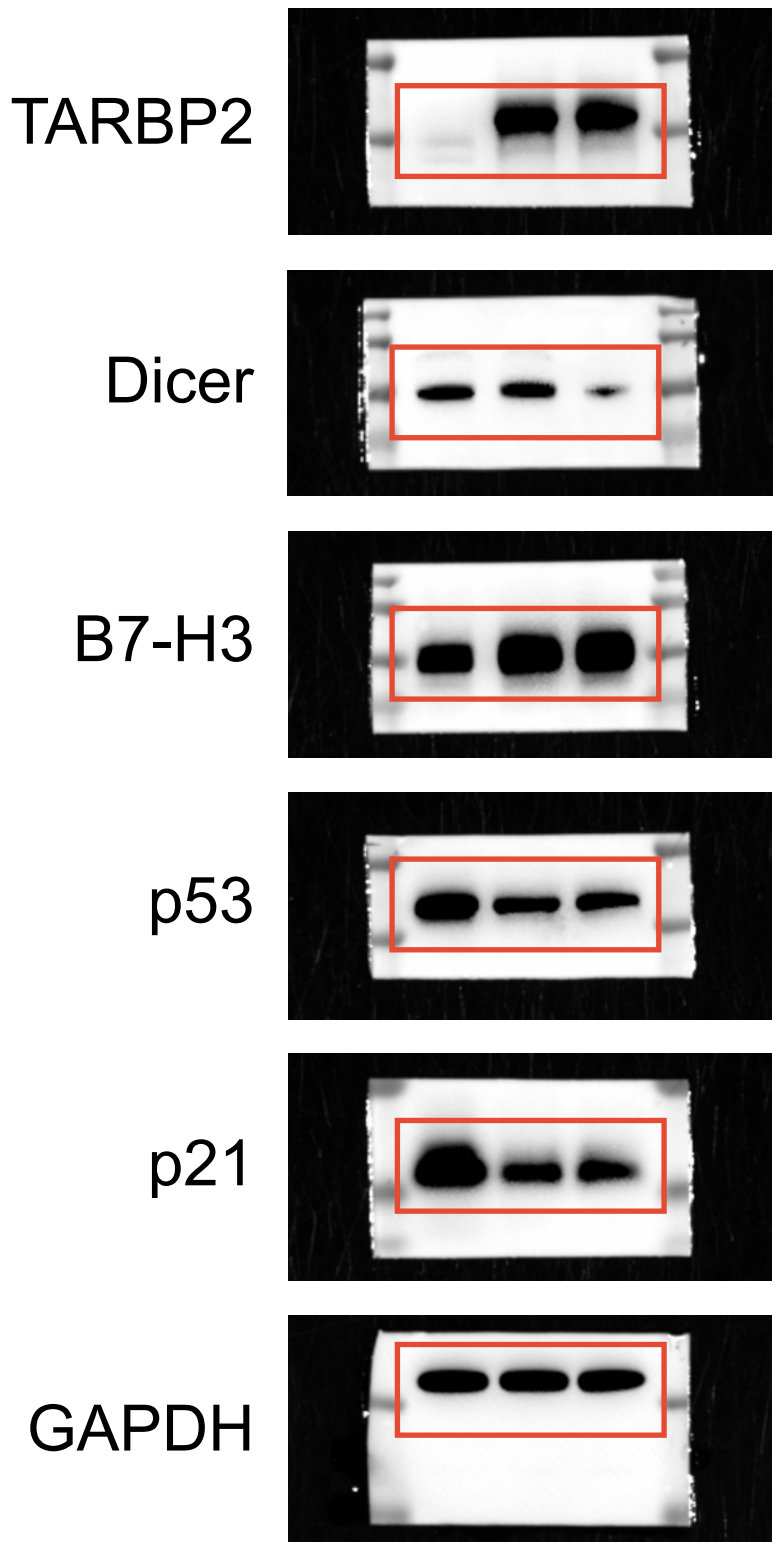

# Full unedited blot for Supplemental Figure 12D

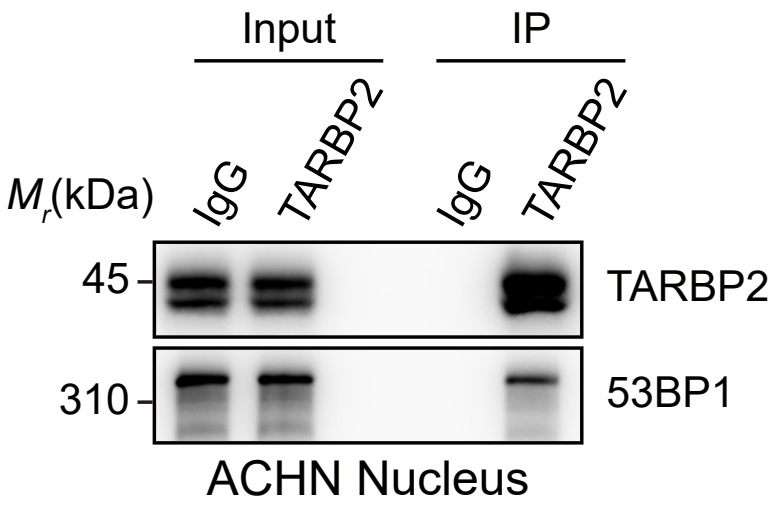


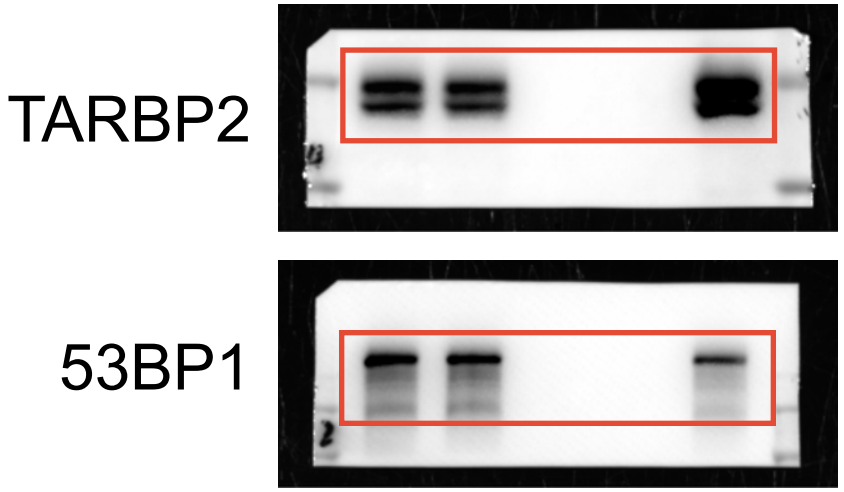

Full unedited blot for Supplemental Figure 12E

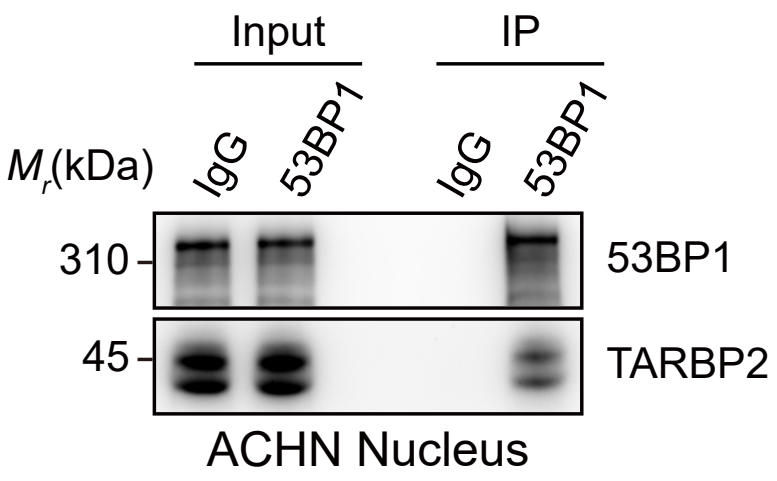


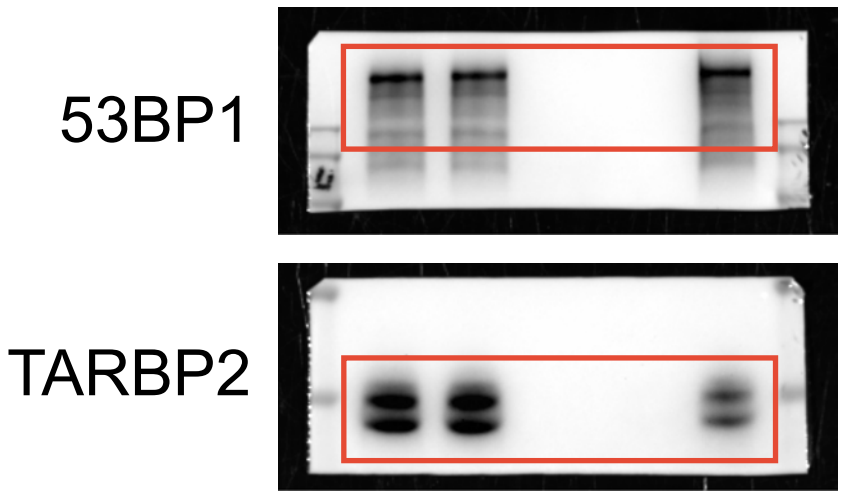

Full unedited blot for Supplemental Figure 12F

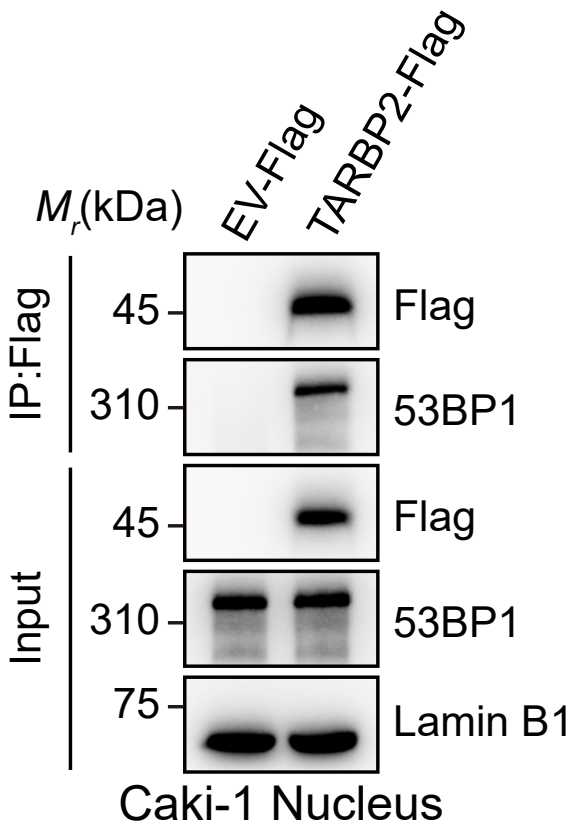


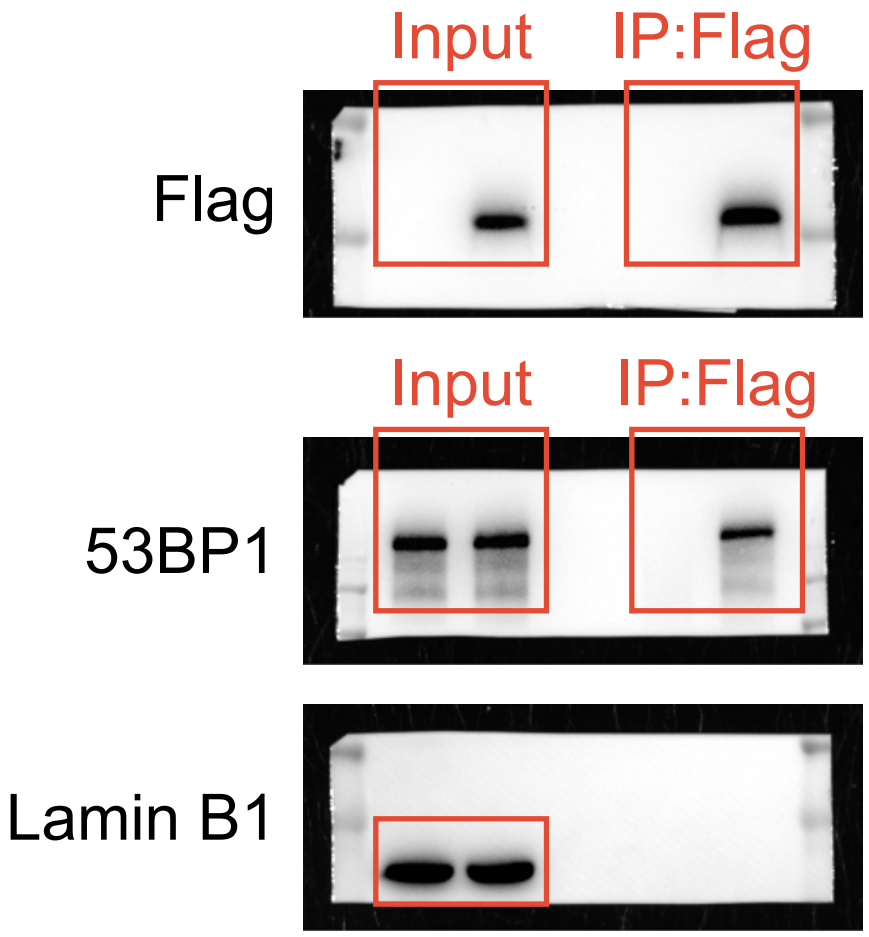

Full unedited blot for Supplemental Figure 12G

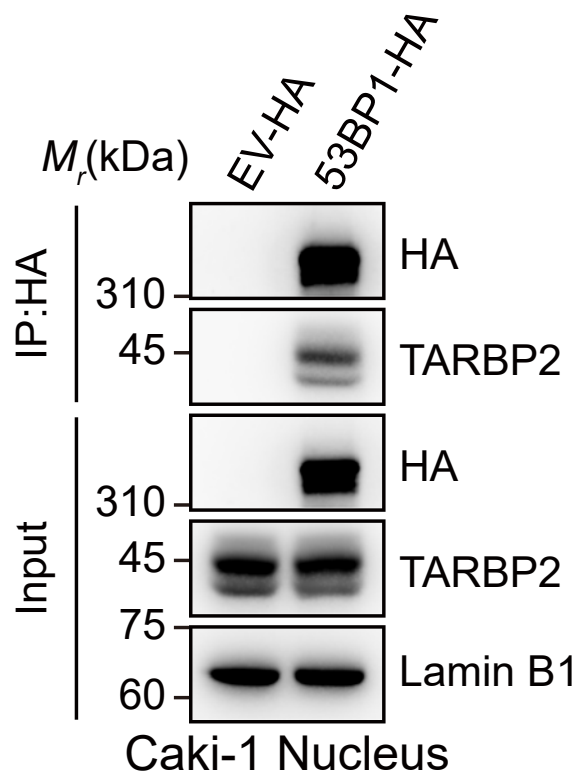


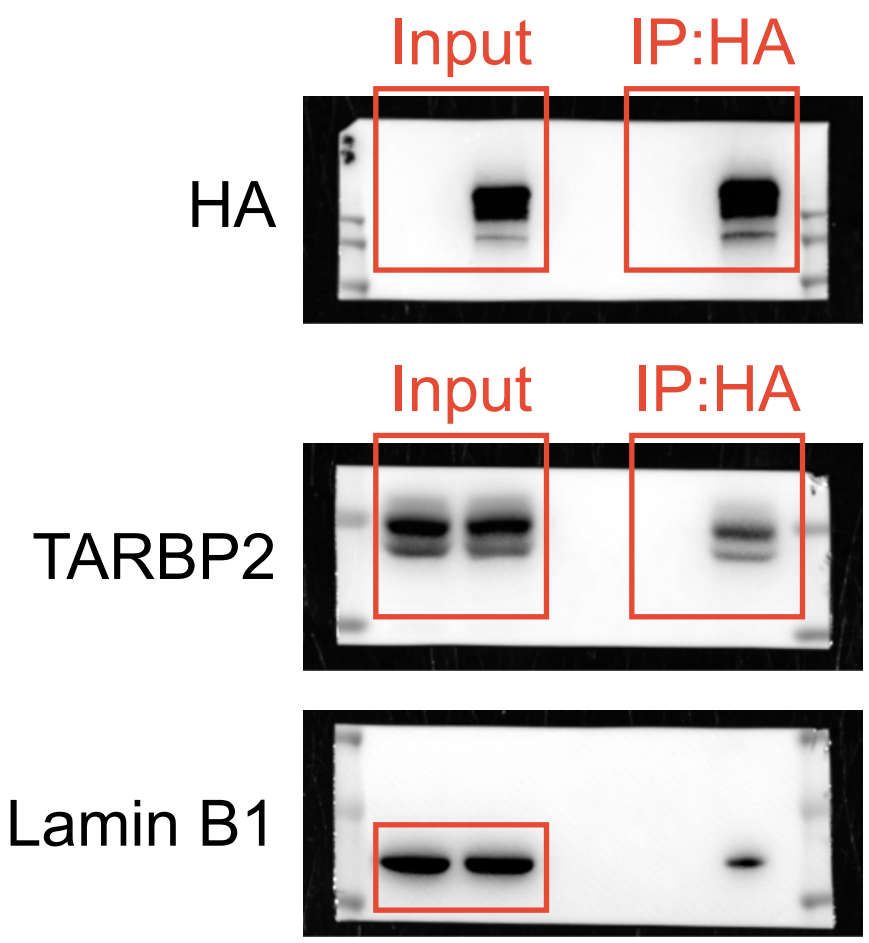

Full unedited blot for Supplemental Figure 12I

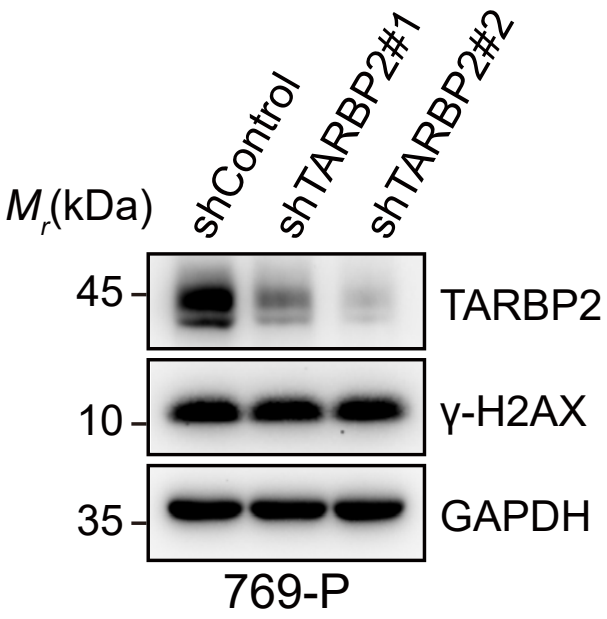


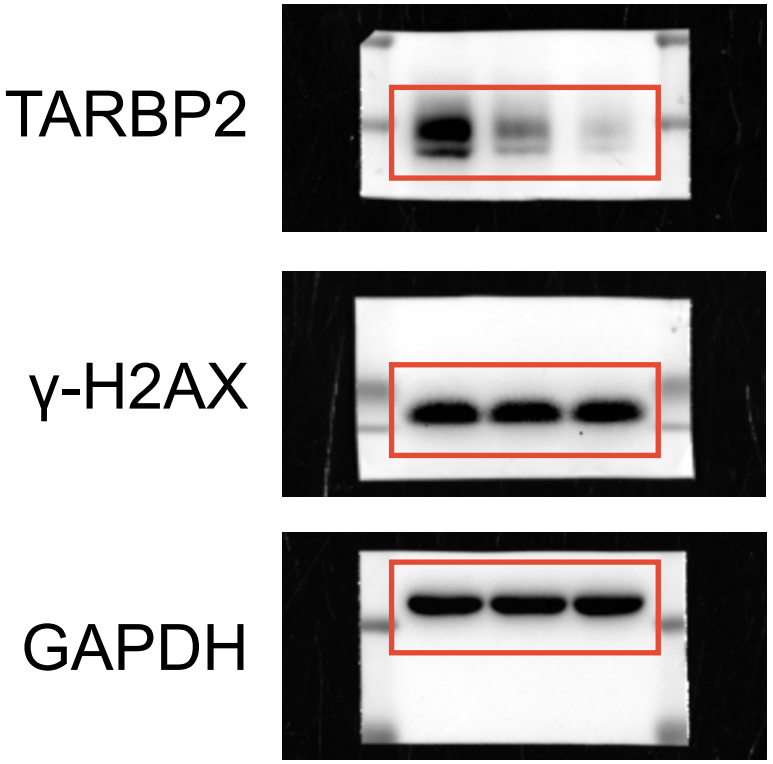

# Full unedited blot for Supplemental Figure 12J

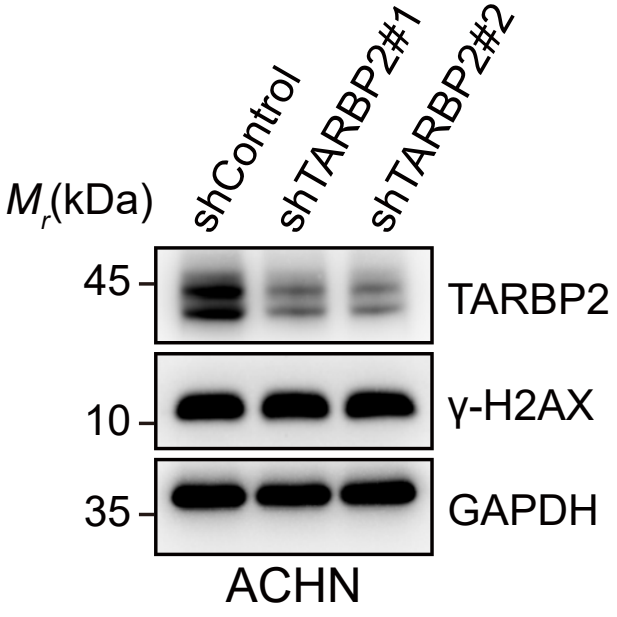


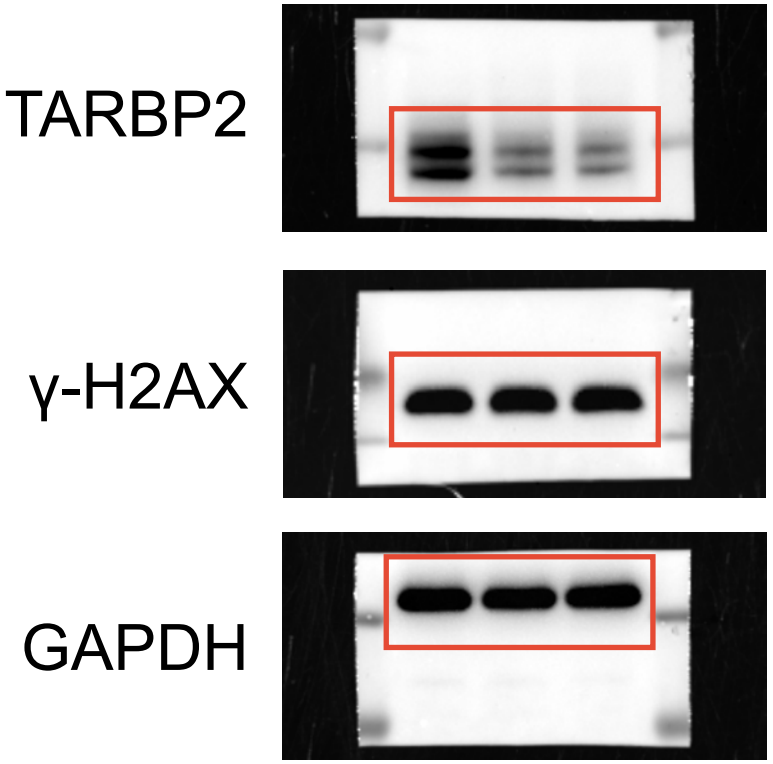

Full unedited blot for Supplemental Figure 12K

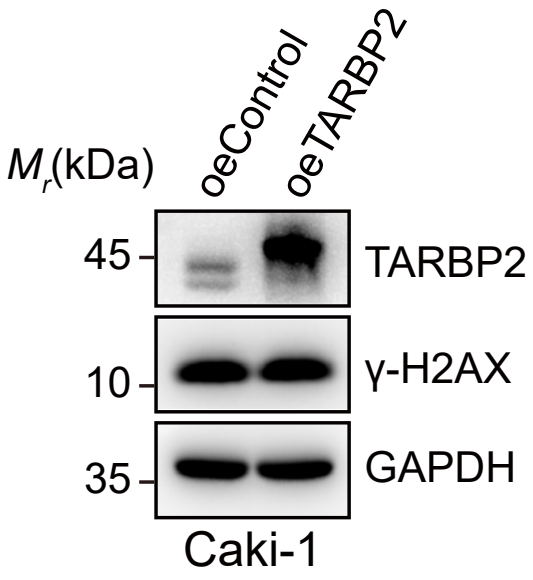


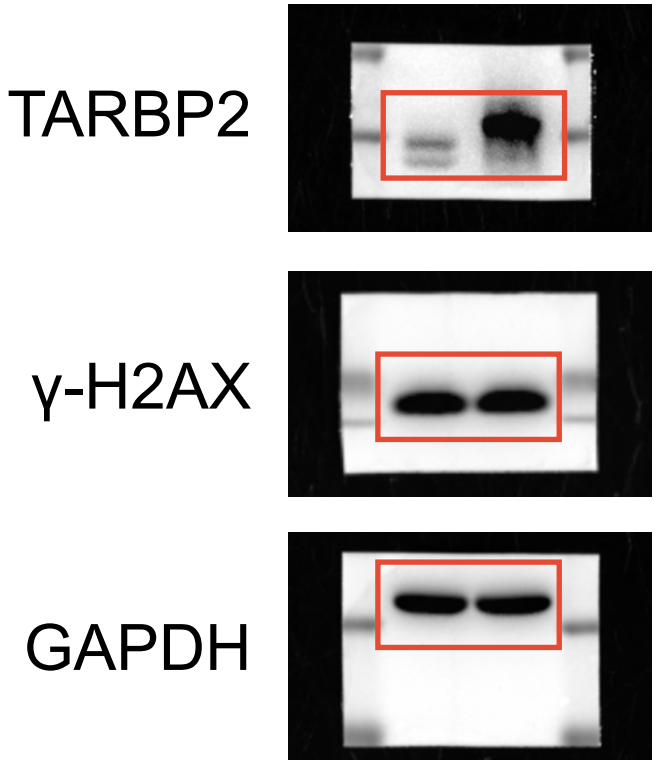

# Full unedited blot for Supplemental Figure 12L

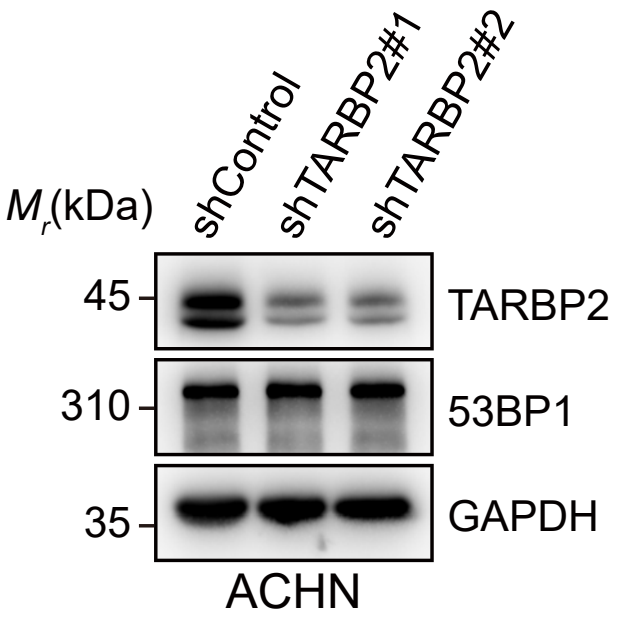


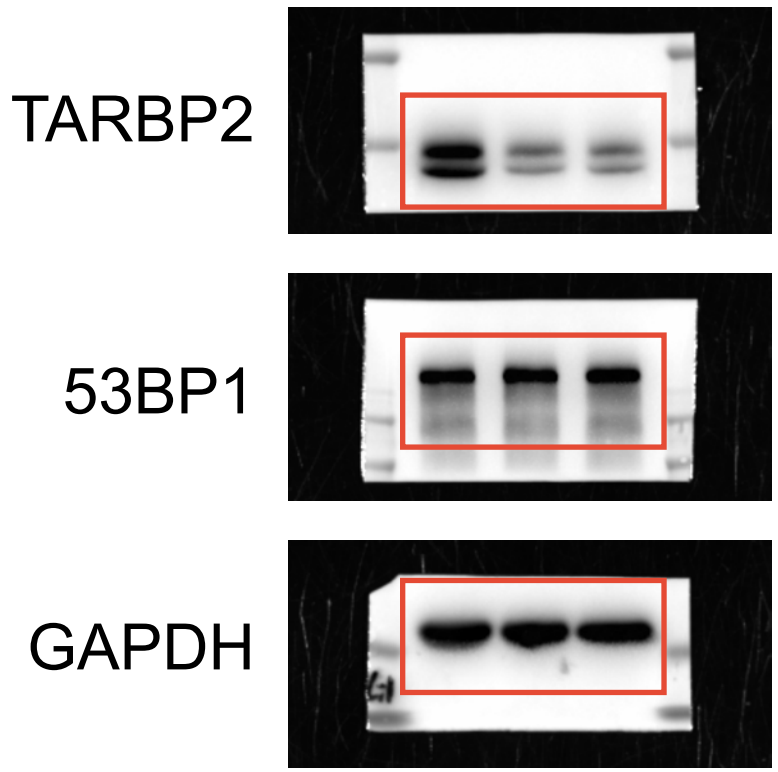

Full unedited blot for Supplemental Figure 12N

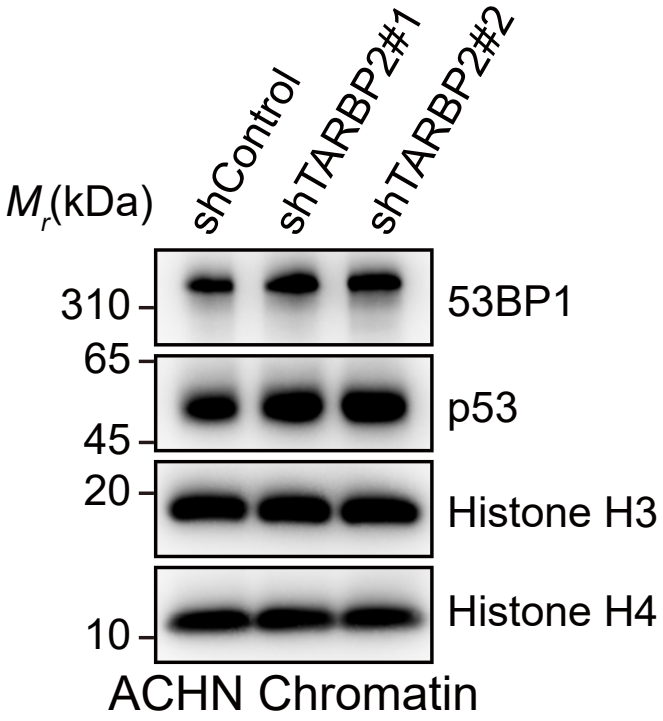


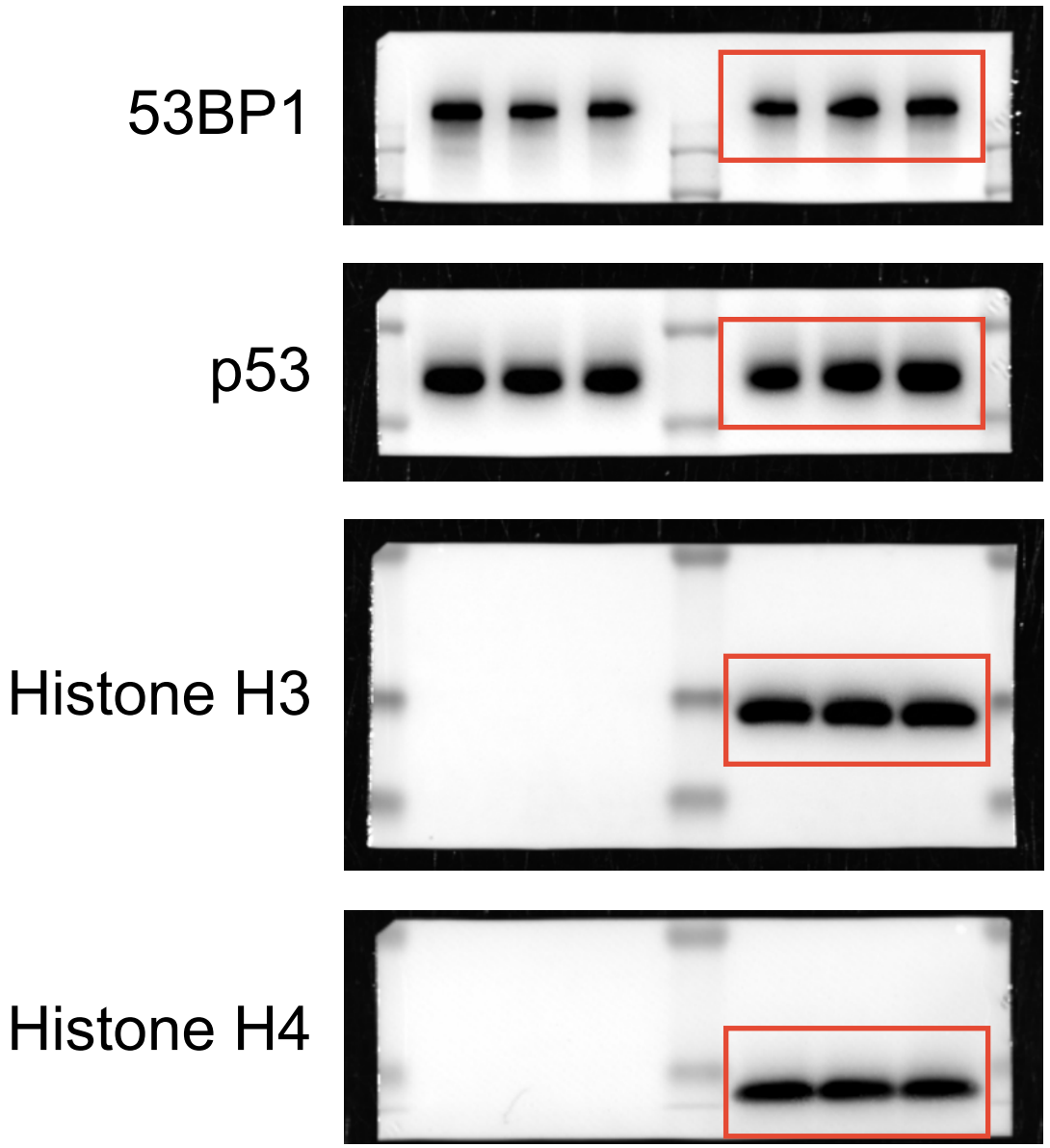

Full unedited blot for Supplemental Figure 13A

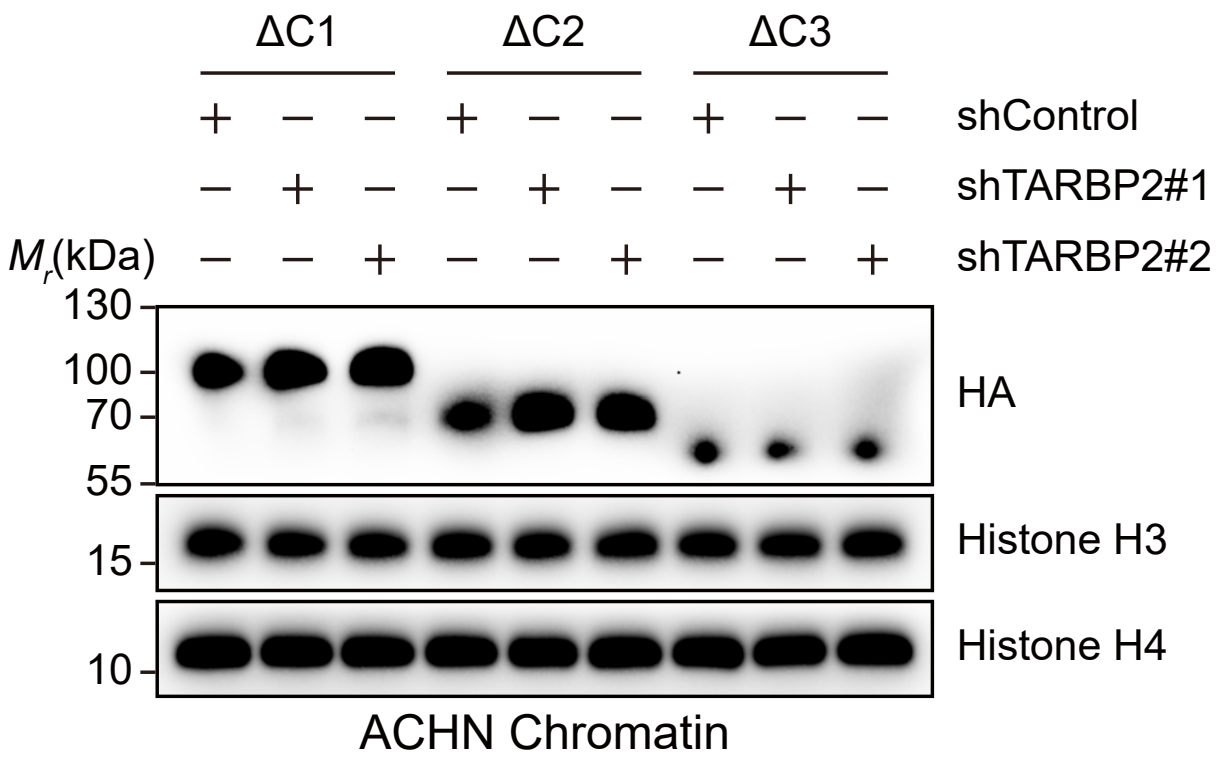


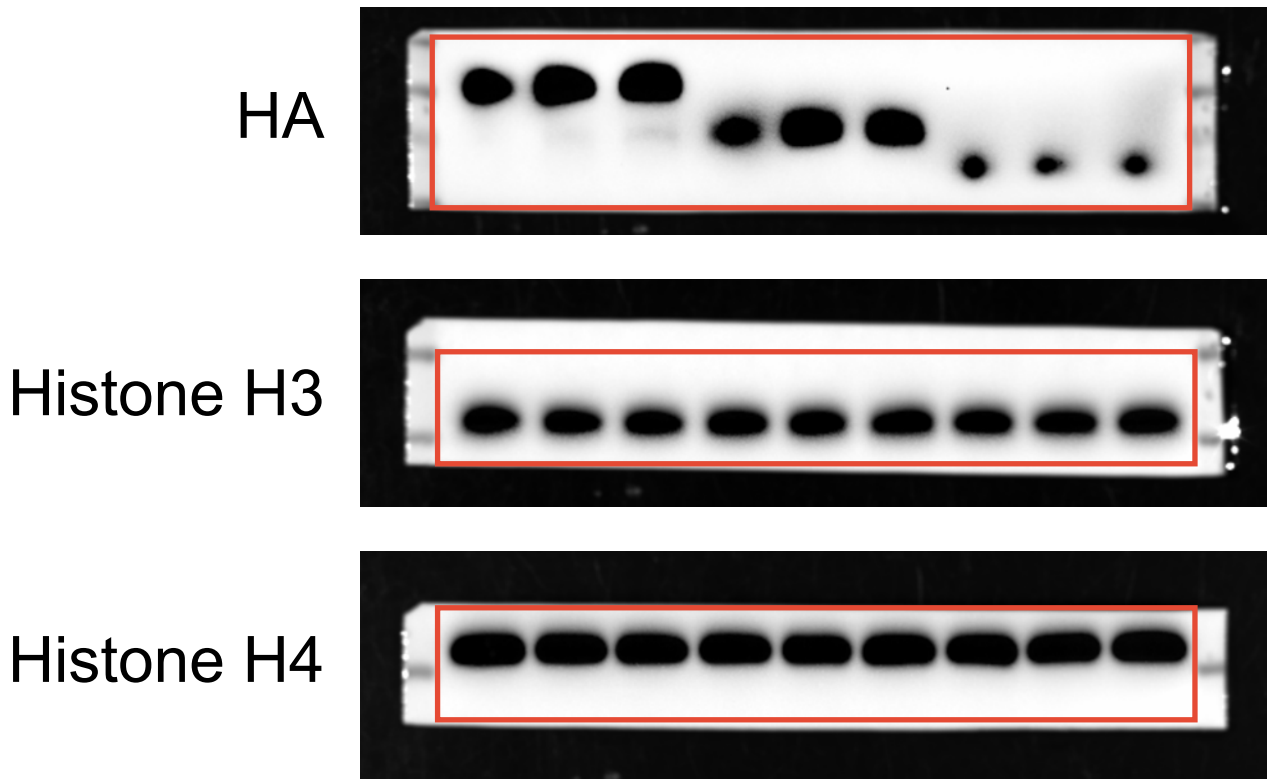

# Full unedited blot for Supplemental Figure 14B

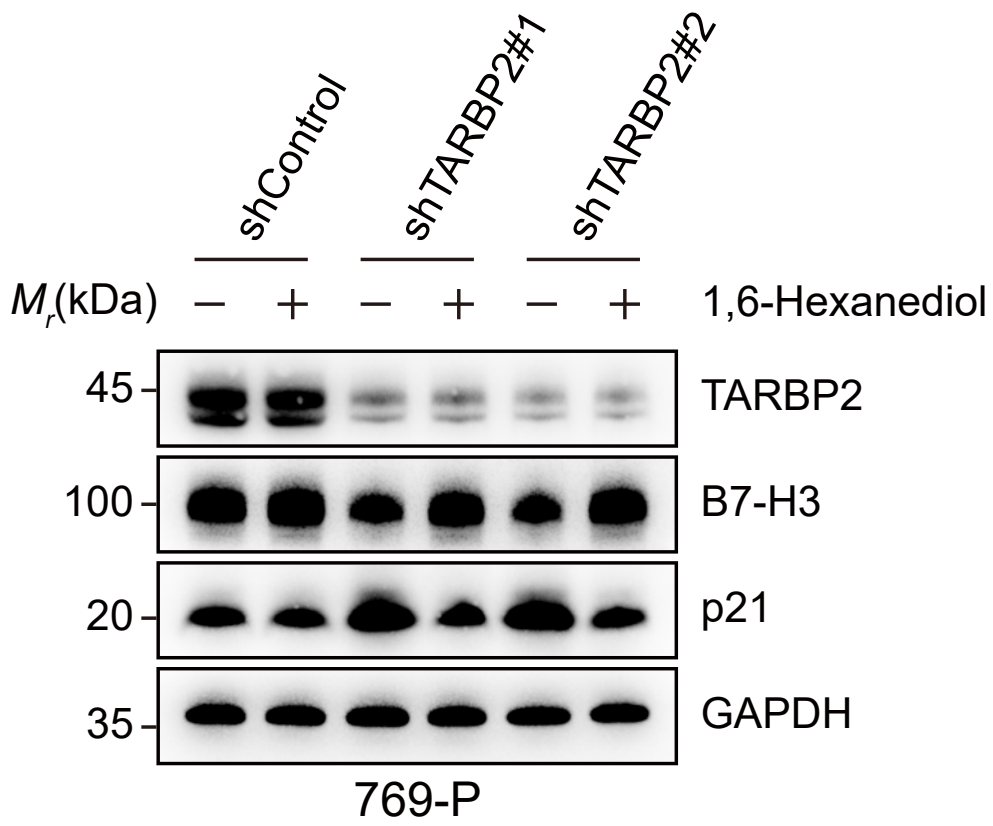


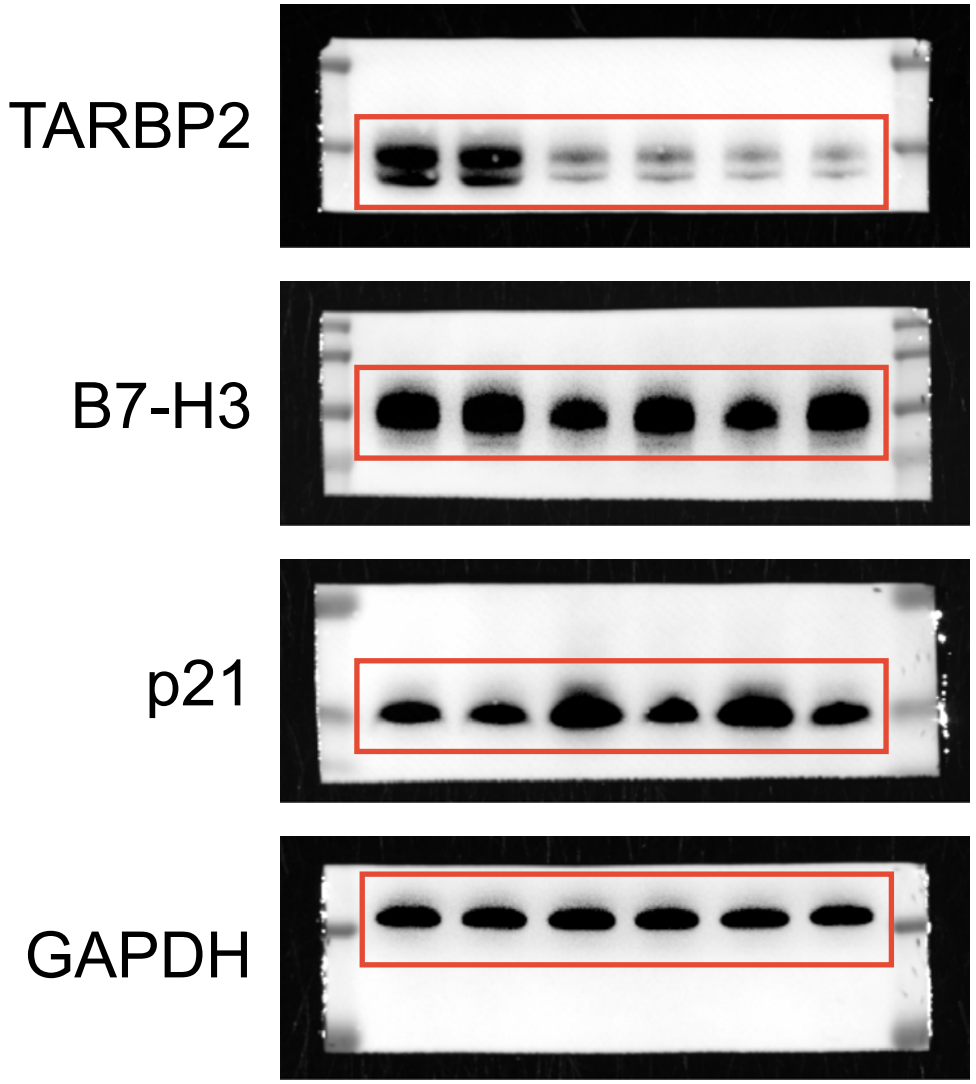

Full unedited blot for Supplemental Figure 14D

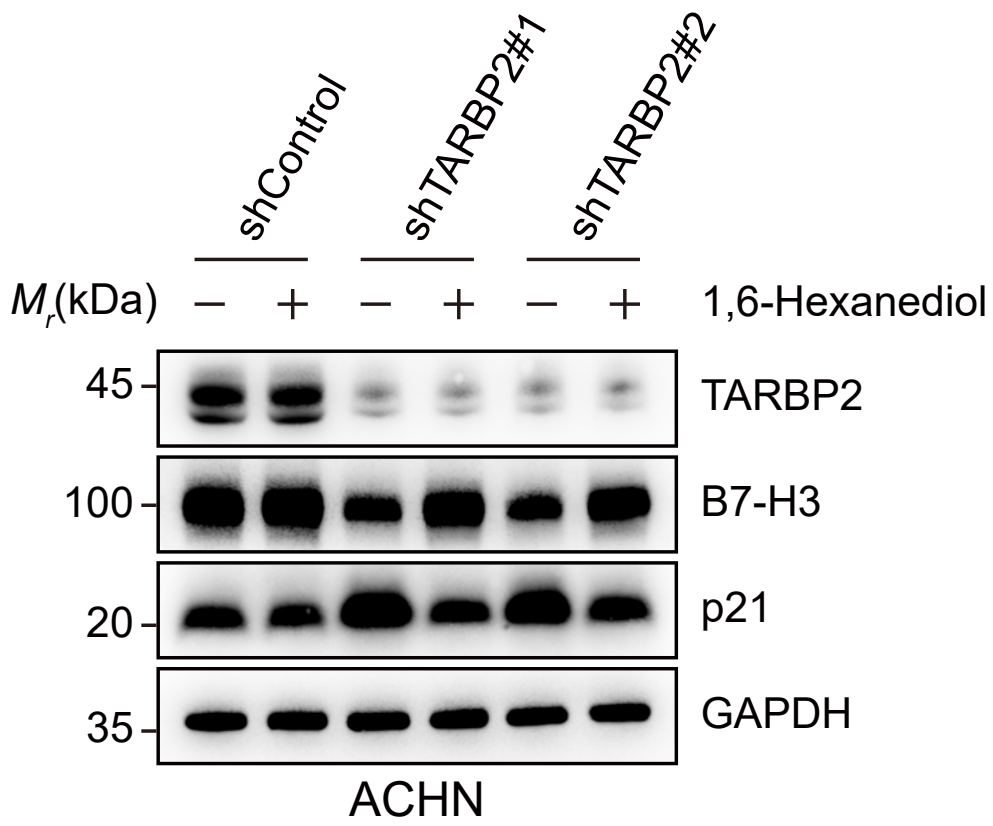


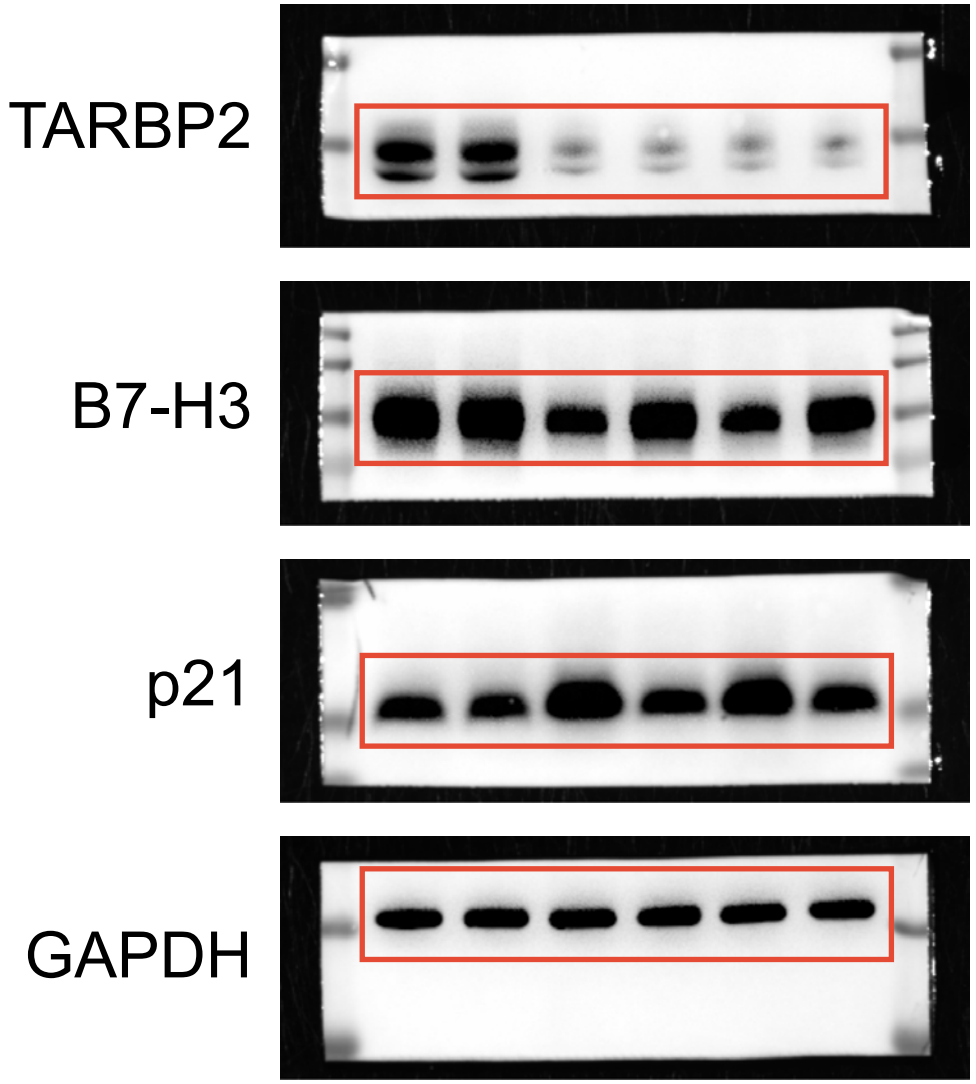

# Full unedited blot for Supplemental Figure 14F

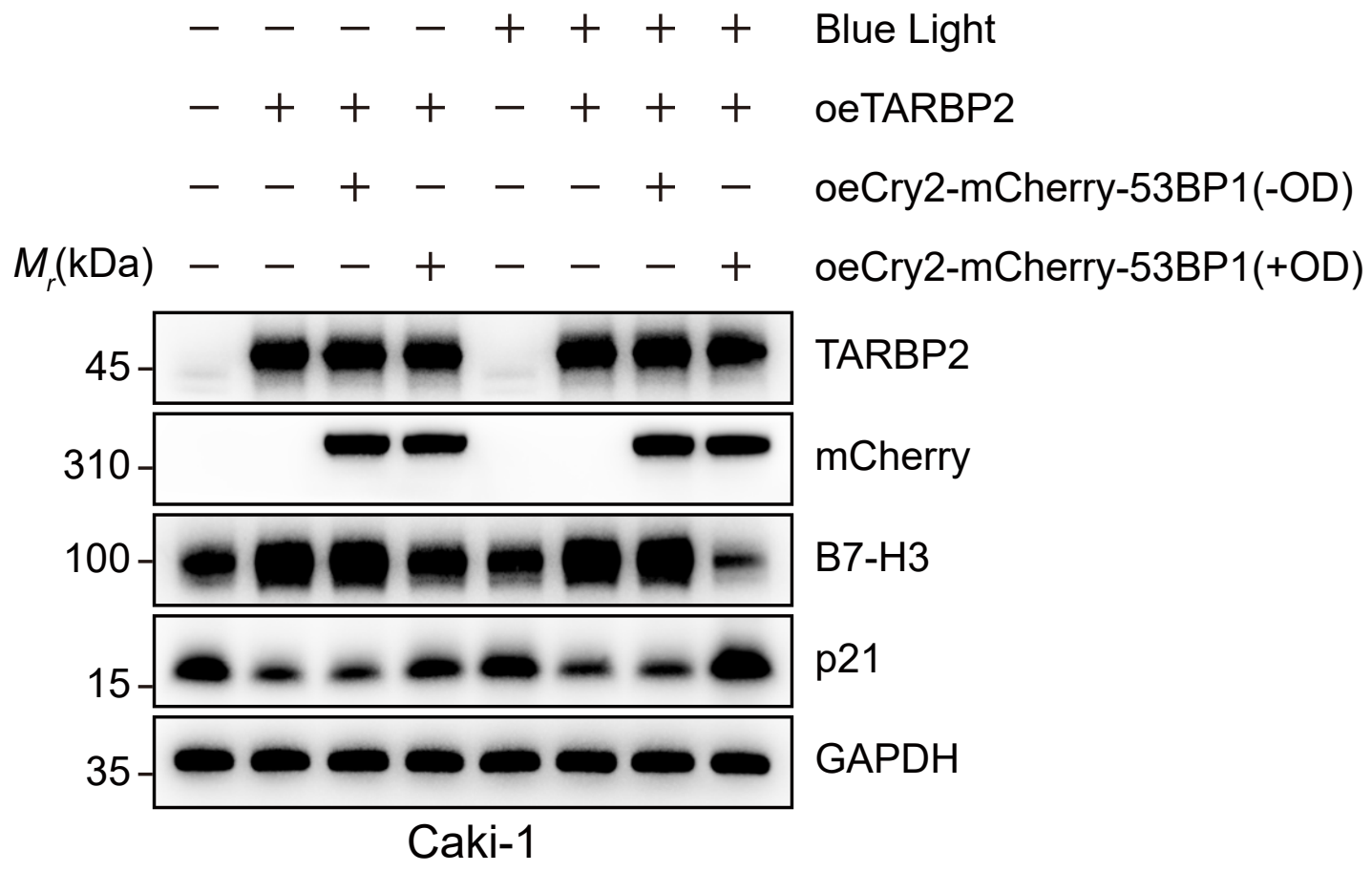


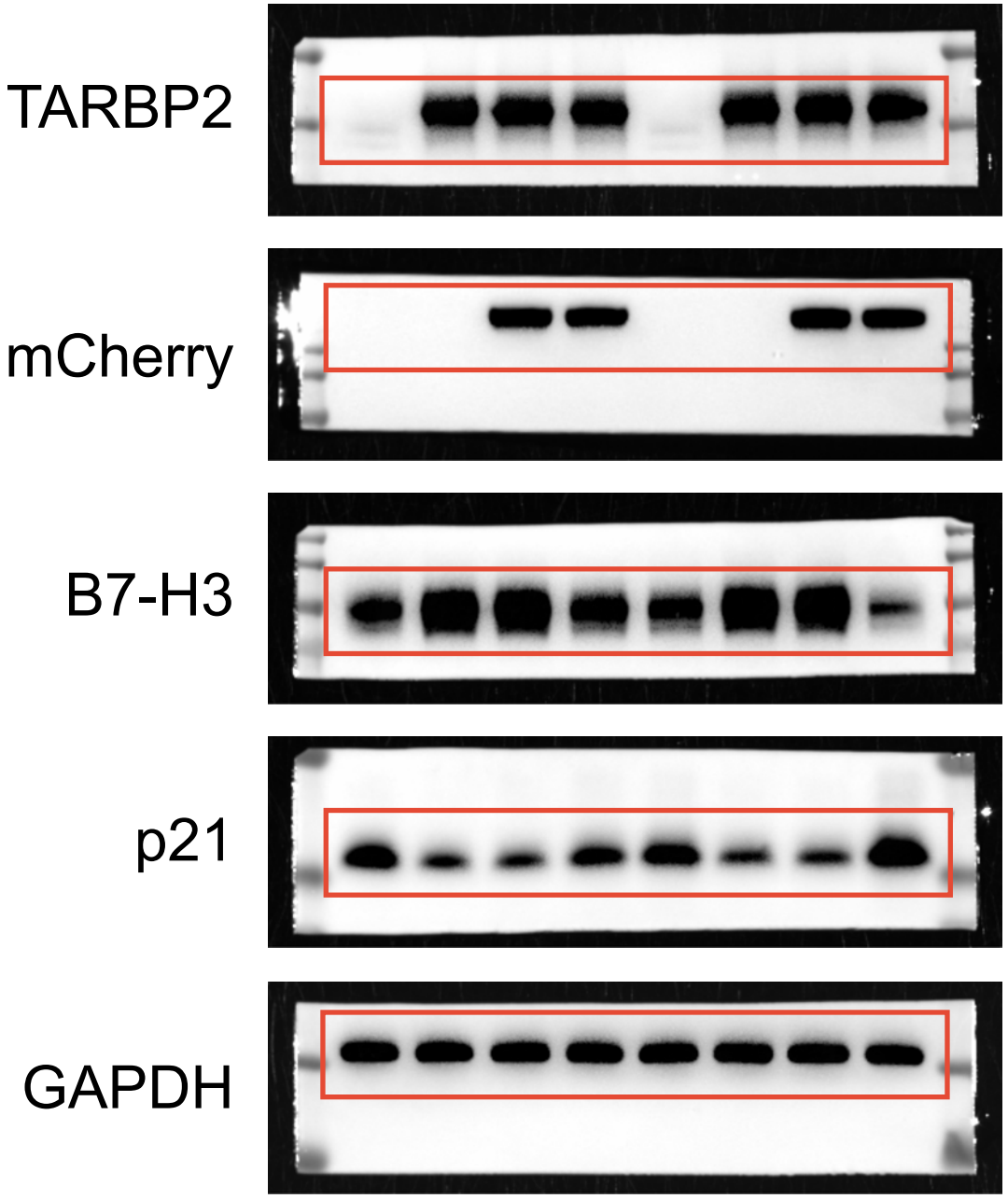

Supplement: Supplementary file 4 [file jci-136-199525-s009.pdf]
